# Supplementary material for: Diagnostic yield of nine user-friendly bioinformatics tools for predicting Mycobacterium tuberculosis drug resistance: A systematic review and network meta-analysis
Source: PLOS Glob Public Health. 2025 Apr 21;5(4):e0004465. doi: 10.1371/journal.pgph.0004465 (PMC12011222; doi:10.1371/journal.pgph.0004465)
Supplement: S1 Fig — (PDF) [file pgph.0004465.s001.pdf]

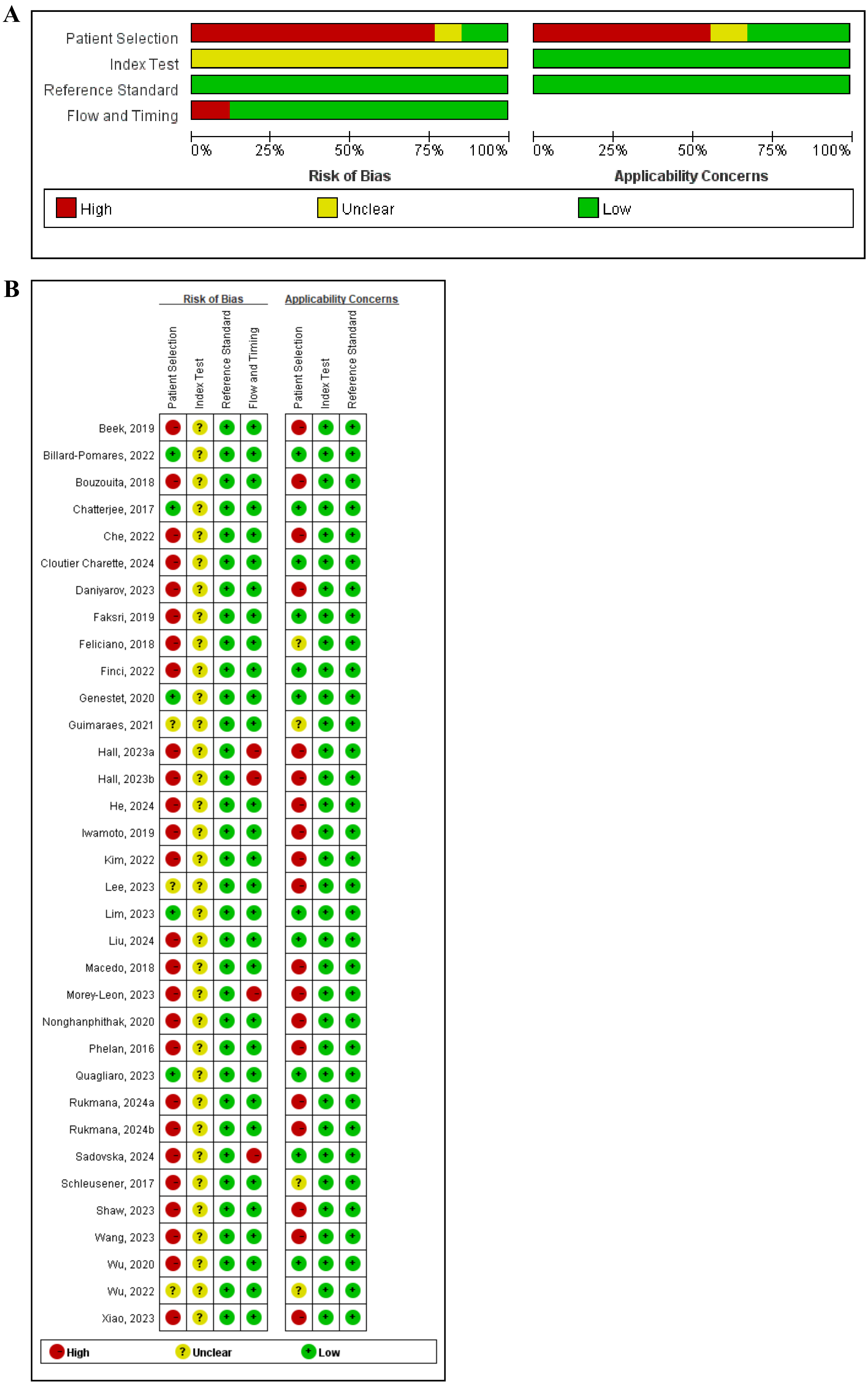

Figure 1. Risk of bias and applicability concerns of included studies assessed using QUADAS-2: (A) Methodological quality graph; (B) Methodological quality summary.
