# Supplementary material for: Diagnostic yield of nine user-friendly bioinformatics tools for predicting Mycobacterium tuberculosis drug resistance: A systematic review and network meta-analysis
Source: PLOS Glob Public Health. 2025 Apr 21;5(4):e0004465. doi: 10.1371/journal.pgph.0004465 (PMC12011222; doi:10.1371/journal.pgph.0004465)

# Figure 2. Forest plots of diagnostic yield for all bioinformatics tools across 14 anti-TB drugs.

## Isoniazid

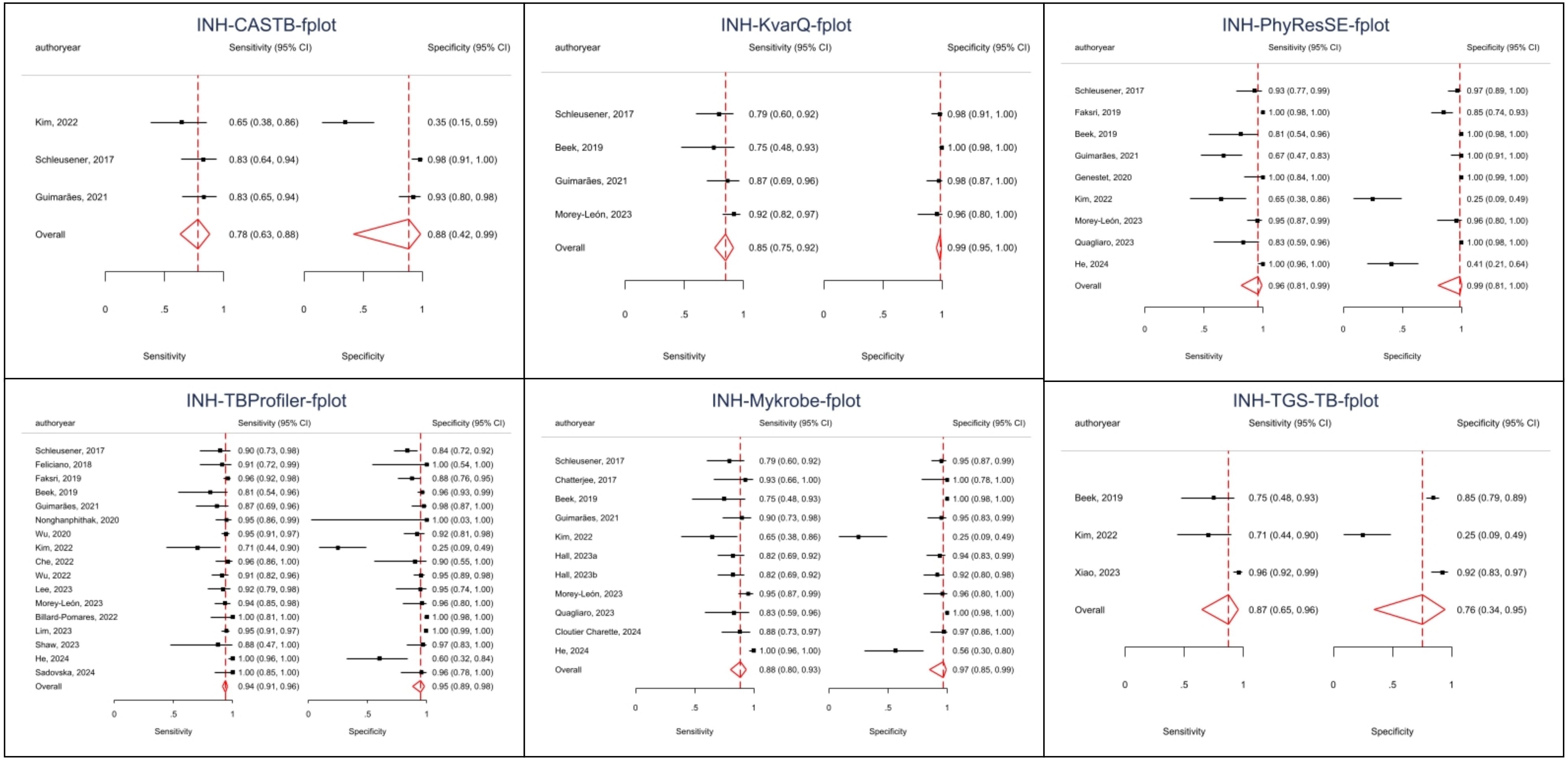

# Rifampicin

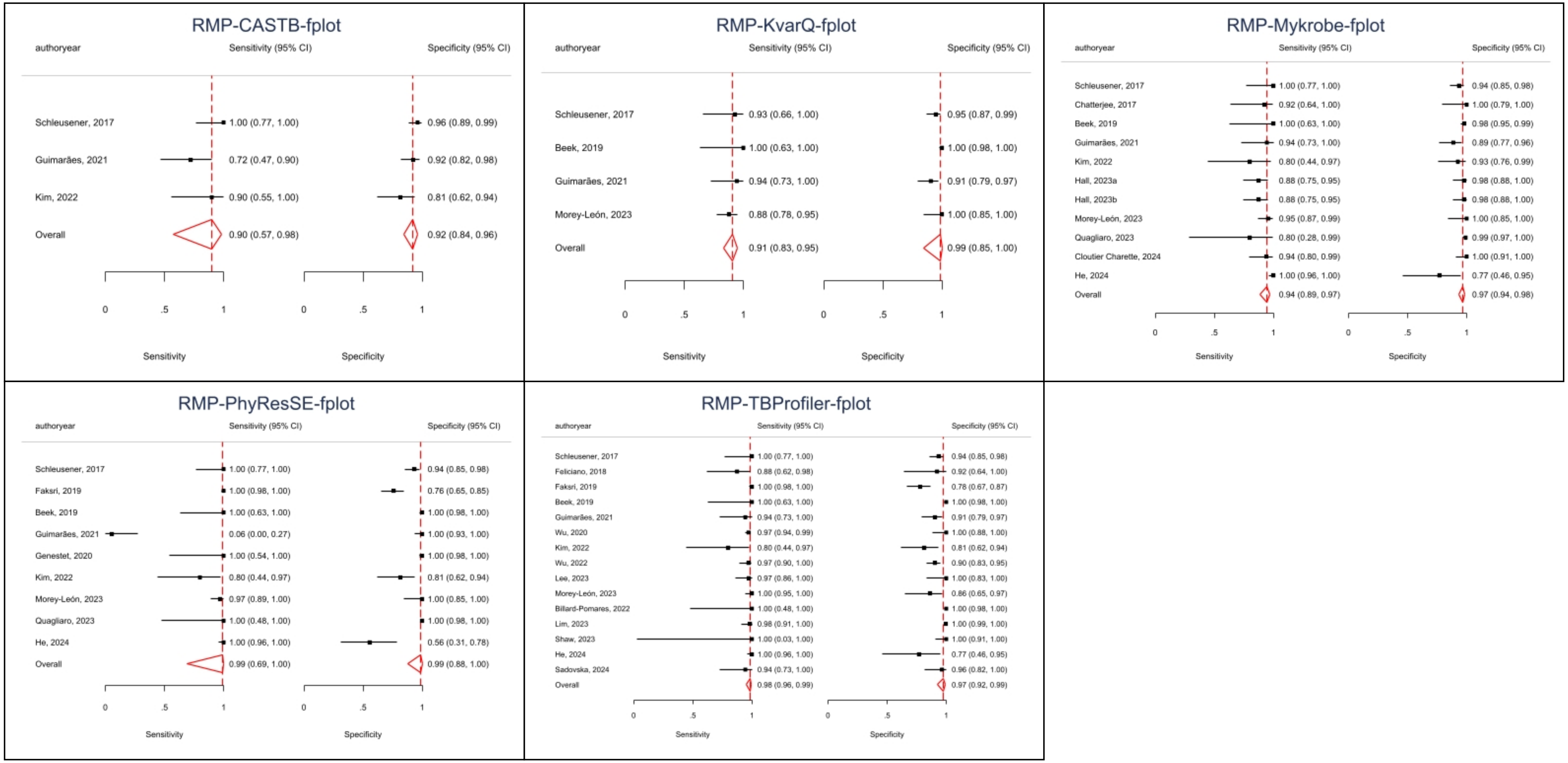
RMP-CASTB-fplot
authoryear
Sensitivity (95% CI)
Specificity (95% CI)
Schleusener, 2017 1.00 (0.77, 1.00) 0.96 (0.89, 0.99)
Guimarães, 2021 0.72 (0.47, 0.90) 0.92 (0.82, 0.98)
Kim, 2022 0.90 (0.55, 1.00) 0.81 (0.62, 0.94)
Overall 0.90 (0.57, 0.98) 0.92 (0.84, 0.96)
Sensitivity
Specificity
RMP-KvarQ-fplot
authoryear
Sensitivity (95% CI)
Specificity (95% CI)
Schleusener, 2017 0.93 (0.66, 1.00) 0.95 (0.87, 0.99)
Beek, 2019 1.00 (0.63, 1.00) 1.00 (0.98, 1.00)
Guimarães, 2021 0.94 (0.73, 1.00) 0.91 (0.79, 0.97)
Morey-León, 2023 0.88 (0.78, 0.95) 1.00 (0.85, 1.00)
Overall 0.91 (0.83, 0.95) 0.99 (0.85, 1.00)
Sensitivity
Specificity
RMP-Mykrobe-fplot
authoryear
Sensitivity (95% CI)
Specificity (95% CI)
Schleusener, 2017 1.00 (0.77, 1.00) 0.94 (0.85, 0.98)
Chatterjee, 2017 0.92 (0.64, 1.00) 1.00 (0.79, 1.00)
Beek, 2019 1.00 (0.63, 1.00) 0.98 (0.95, 0.99)
Guimarães, 2021 0.94 (0.73, 1.00) 0.89 (0.77, 0.96)
Kim, 2022 0.80 (0.44, 0.97) 0.93 (0.76, 0.99)
Hall, 2023a 0.88 (0.75, 0.95) 0.98 (0.88, 1.00)
Hall, 2023b 0.88 (0.75, 0.95) 0.98 (0.88, 1.00)
Morey-León, 2023 0.95 (0.87, 0.99) 1.00 (0.85, 1.00)
Quagliaro, 2023 0.80 (0.28, 0.99) 0.99 (0.97, 1.00)
Cloutier Charette, 2024 0.94 (0.80, 0.99) 1.00 (0.91, 1.00)
He, 2024 1.00 (0.96, 1.00) 0.77 (0.46, 0.95)
Overall 0.94 (0.89, 0.97) 0.97 (0.94, 0.98)
Sensitivity
Specificity
RMP-PhyResSE-fplot
authoryear
Sensitivity (95% CI)
Specificity (95% CI)
Schleusener, 2017 1.00 (0.77, 1.00) 0.94 (0.85, 0.98)
Faksri, 2019 1.00 (0.98, 1.00) 0.76 (0.65, 0.85)
Beek, 2019 1.00 (0.63, 1.00) 1.00 (0.98, 1.00)
Guimarães, 2021 0.06 (0.00, 0.27) 1.00 (0.93, 1.00)
Genestet, 2020 1.00 (0.54, 1.00) 1.00 (0.98, 1.00)
Kim, 2022 0.80 (0.44, 0.97) 0.81 (0.62, 0.94)
Morey-León, 2023 0.97 (0.89, 1.00) 1.00 (0.85, 1.00)
Quagliaro, 2023 1.00 (0.48, 1.00) 1.00 (0.98, 1.00)
He, 2024 1.00 (0.96, 1.00) 0.56 (0.31, 0.78)
Overall 0.99 (0.69, 1.00) 0.99 (0.88, 1.00)
Sensitivity
Specificity
RMP-TBProfiler-fplot
authoryear
Sensitivity (95% CI)
Specificity (95% CI)
Schleusener, 2017 1.00 (0.77, 1.00) 0.94 (0.85, 0.98)
Feliciano, 2018 0.88 (0.62, 0.98) 0.92 (0.64, 1.00)
Faksri, 2019 1.00 (0.98, 1.00) 0.78 (0.67, 0.87)
Beek, 2019 1.00 (0.63, 1.00) 1.00 (0.98, 1.00)
Guimarães, 2021 0.94 (0.73, 1.00) 0.91 (0.79, 0.97)
Wu, 2020 0.97 (0.94, 0.99) 1.00 (0.88, 1.00)
Kim, 2022 0.80 (0.44, 0.97) 0.81 (0.62, 0.94)
Wu, 2022 0.97 (0.90, 1.00) 0.90 (0.83, 0.95)
Lee, 2023 0.97 (0.86, 1.00) 1.00 (0.83, 1.00)
Morey-León, 2023 1.00 (0.95, 1.00) 0.86 (0.65, 0.97)
Billard-Pomares, 2022 1.00 (0.48, 1.00) 1.00 (0.98, 1.00)
Lim, 2023 0.98 (0.91, 1.00) 1.00 (0.99, 1.00)
Shaw, 2023 1.00 (0.03, 1.00) 1.00 (0.91, 1.00)
He, 2024 1.00 (0.96, 1.00) 0.77 (0.46, 0.95)
Sadovska, 2024 0.94 (0.73, 1.00) 0.96 (0.82, 1.00)
Overall 0.98 (0.96, 0.99) 0.97 (0.92, 0.99)
Sensitivity
Specificity

# Ethambutol

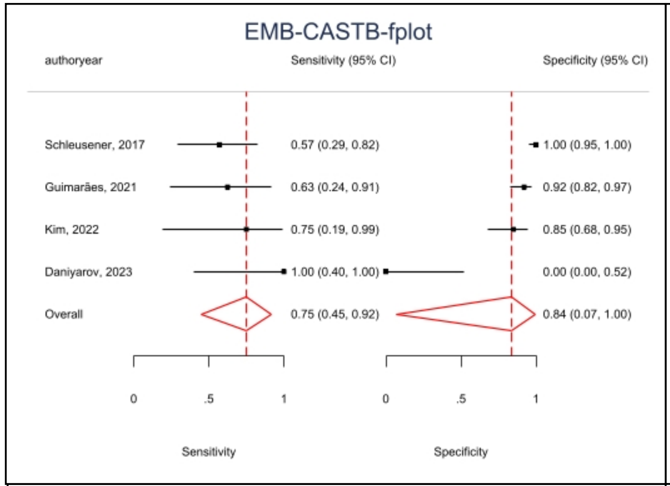
EMB-CASTB-fplot
authoryear
Sensitivity (95% CI)
Specificity (95% CI)
Schleusener, 2017 0.57 (0.29, 0.82) 1.00 (0.95, 1.00)
Guimarães, 2021 0.63 (0.24, 0.91) 0.92 (0.82, 0.97)
Kim, 2022 0.75 (0.19, 0.99) 0.85 (0.68, 0.95)
Daniyarov, 2023 1.00 (0.40, 1.00) 0.00 (0.00, 0.52)
Overall 0.75 (0.45, 0.92) 0.84 (0.07, 1.00)
0 .5 1
0 .5 1
Sensitivity
Specificity

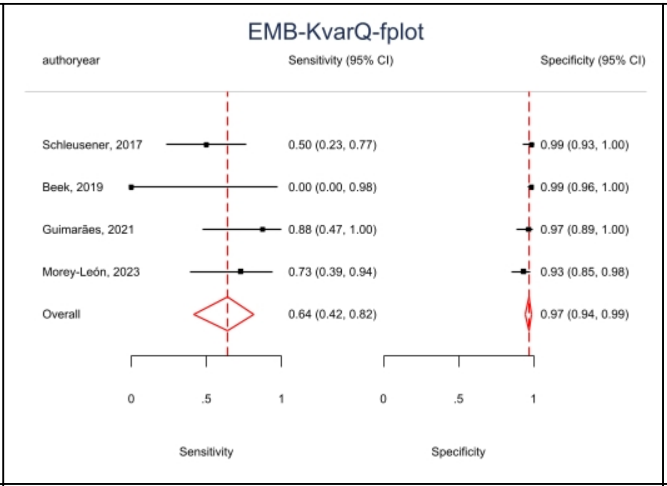
EMB-KvarQ-fplot
authoryear
Sensitivity (95% CI)
Specificity (95% CI)
Schleusener, 2017 0.50 (0.23, 0.77) 0.99 (0.93, 1.00)
Beek, 2019 0.00 (0.00, 0.98) 0.99 (0.96, 1.00)
Guimarães, 2021 0.88 (0.47, 1.00) 0.97 (0.89, 1.00)
Morey-León, 2023 0.73 (0.39, 0.94) 0.93 (0.85, 0.98)
Overall 0.64 (0.42, 0.82) 0.97 (0.94, 0.99)
0 .5 1
0 .5 1
Sensitivity
Specificity

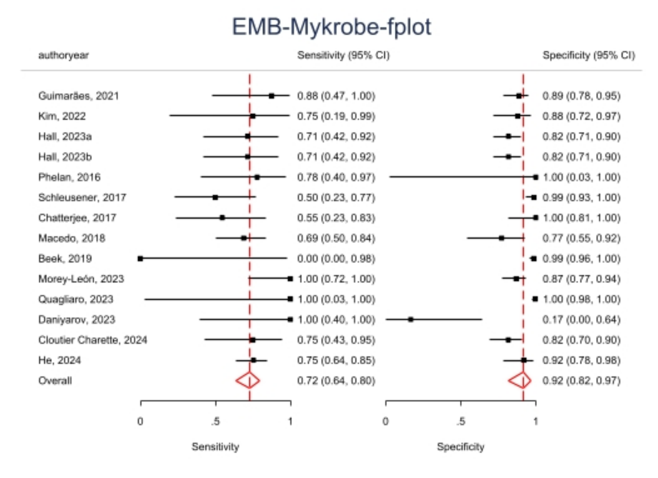
EMB-Mykrobe-fplot
authoryear
Sensitivity (95% CI)
Specificity (95% CI)
Guimarães, 2021 0.88 (0.47, 1.00) 0.89 (0.78, 0.95)
Kim, 2022 0.75 (0.19, 0.99) 0.88 (0.72, 0.97)
Hall, 2023a 0.71 (0.42, 0.92) 0.82 (0.71, 0.90)
Hall, 2023b 0.71 (0.42, 0.92) 0.82 (0.71, 0.90)
Phelan, 2016 0.78 (0.40, 0.97) 1.00 (0.03, 1.00)
Schleusener, 2017 0.50 (0.23, 0.77) 0.99 (0.93, 1.00)
Chatterjee, 2017 0.55 (0.23, 0.83) 1.00 (0.81, 1.00)
Macedo, 2018 0.69 (0.50, 0.84) 0.77 (0.55, 0.92)
Beek, 2019 0.00 (0.00, 0.98) 0.99 (0.96, 1.00)
Morey-León, 2023 1.00 (0.72, 1.00) 0.87 (0.77, 0.94)
Quagliaro, 2023 1.00 (0.03, 1.00) 1.00 (0.98, 1.00)
Daniyarov, 2023 1.00 (0.40, 1.00) 0.17 (0.00, 0.64)
Cloutier Charette, 2024 0.75 (0.43, 0.95) 0.82 (0.70, 0.90)
He, 2024 0.75 (0.64, 0.85) 0.92 (0.78, 0.98)
Overall 0.72 (0.64, 0.80) 0.92 (0.82, 0.97)
0 .5 1
0 .5 1
Sensitivity
Specificity

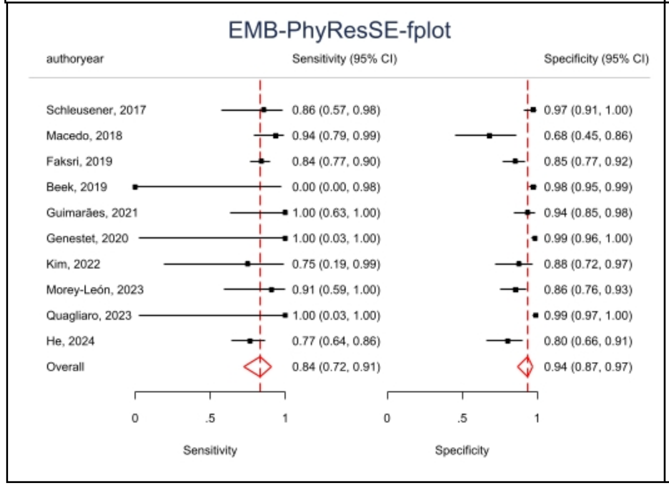
EMB-PhyResSE-fplot
authoryear
Sensitivity (95% CI)
Specificity (95% CI)
Schleusener, 2017 0.86 (0.57, 0.98) 0.97 (0.91, 1.00)
Macedo, 2018 0.94 (0.79, 0.99) 0.68 (0.45, 0.86)
Faksri, 2019 0.84 (0.77, 0.90) 0.85 (0.77, 0.92)
Beek, 2019 0.00 (0.00, 0.98) 0.98 (0.95, 0.99)
Guimarães, 2021 1.00 (0.63, 1.00) 0.94 (0.85, 0.98)
Genestet, 2020 1.00 (0.03, 1.00) 0.99 (0.96, 1.00)
Kim, 2022 0.75 (0.19, 0.99) 0.88 (0.72, 0.97)
Morey-León, 2023 0.91 (0.59, 1.00) 0.86 (0.76, 0.93)
Quagliaro, 2023 1.00 (0.03, 1.00) 0.99 (0.97, 1.00)
He, 2024 0.77 (0.64, 0.86) 0.80 (0.66, 0.91)
Overall 0.84 (0.72, 0.91) 0.94 (0.87, 0.97)
0 .5 1
0 .5 1
Sensitivity
Specificity

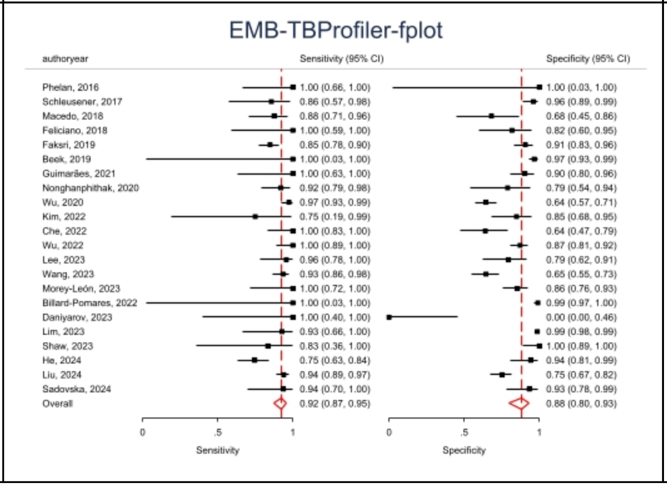
EMB-TBProfiler-fplot
authoryear
Sensitivity (95% CI)
Specificity (95% CI)
Phelan, 2016 1.00 (0.66, 1.00) 1.00 (0.03, 1.00)
Schleusener, 2017 0.86 (0.57, 0.98) 0.96 (0.89, 0.99)
Macedo, 2018 0.88 (0.71, 0.96) 0.68 (0.45, 0.86)
Feliciano, 2018 1.00 (0.59, 1.00) 0.82 (0.60, 0.95)
Faksri, 2019 0.85 (0.78, 0.90) 0.91 (0.83, 0.96)
Beek, 2019 1.00 (0.03, 1.00) 0.97 (0.93, 0.99)
Guimarães, 2021 1.00 (0.63, 1.00) 0.90 (0.80, 0.96)
Nonghanphithak, 2020 0.92 (0.79, 0.98) 0.79 (0.54, 0.94)
Wu, 2020 0.97 (0.93, 0.99) 0.64 (0.57, 0.71)
Kim, 2022 0.75 (0.19, 0.99) 0.85 (0.68, 0.95)
Che, 2022 1.00 (0.83, 1.00) 0.64 (0.47, 0.79)
Wu, 2022 1.00 (0.89, 1.00) 0.87 (0.81, 0.92)
Lee, 2023 0.96 (0.78, 1.00) 0.79 (0.62, 0.91)
Wang, 2023 0.93 (0.86, 0.98) 0.65 (0.55, 0.73)
Morey-León, 2023 1.00 (0.72, 1.00) 0.86 (0.76, 0.93)
Billard-Pomares, 2022 1.00 (0.03, 1.00) 0.99 (0.97, 1.00)
Daniyarov, 2023 1.00 (0.40, 1.00) 0.00 (0.00, 0.46)
Lim, 2023 0.93 (0.66, 1.00) 0.99 (0.98, 0.99)
Shaw, 2023 0.83 (0.36, 1.00) 1.00 (0.89, 1.00)
He, 2024 0.75 (0.63, 0.84) 0.94 (0.81, 0.99)
Liu, 2024 0.94 (0.89, 0.97) 0.75 (0.67, 0.82)
Sadovska, 2024 0.94 (0.70, 1.00) 0.93 (0.78, 0.99)
Overall 0.92 (0.87, 0.95) 0.88 (0.80, 0.93)
0 .5 1
0 .5 1
Sensitivity
Specificity

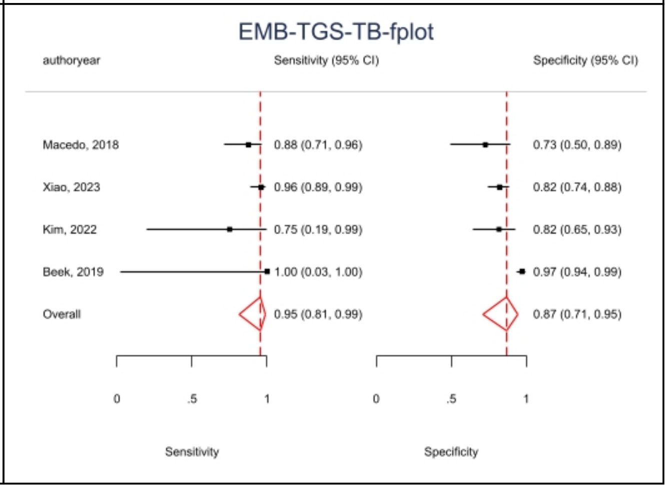
EMB-TGS-TB-fplot
authoryear
Sensitivity (95% CI)
Specificity (95% CI)
Macedo, 2018 0.88 (0.71, 0.96) 0.73 (0.50, 0.89)
Xiao, 2023 0.96 (0.89, 0.99) 0.82 (0.74, 0.88)
Kim, 2022 0.75 (0.19, 0.99) 0.82 (0.65, 0.93)
Beek, 2019 1.00 (0.03, 1.00) 0.97 (0.94, 0.99)
Overall 0.95 (0.81, 0.99) 0.87 (0.71, 0.95)
0 .5 1
0 .5 1
Sensitivity
Specificity

# Pyrazinamide

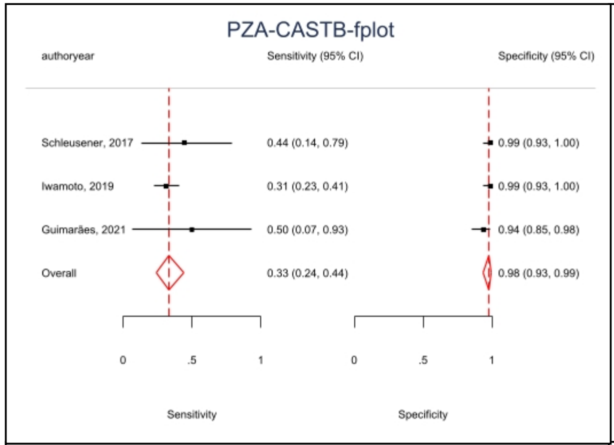


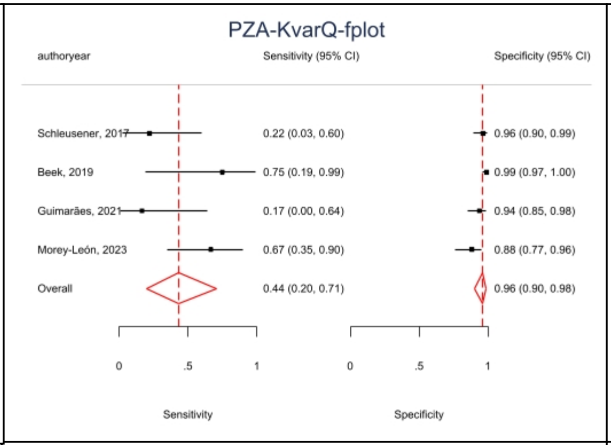


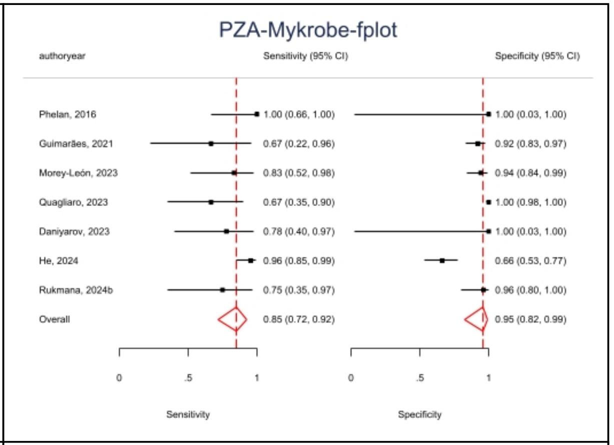


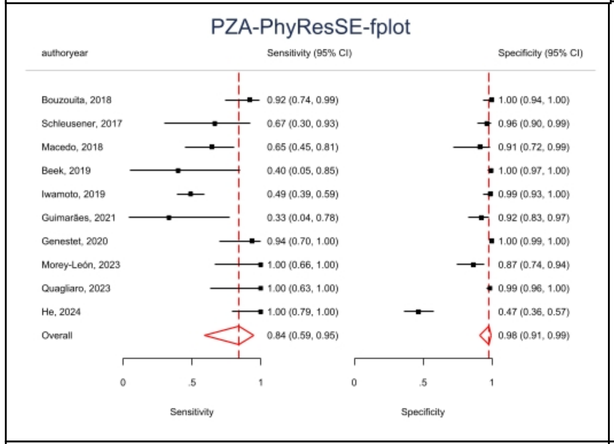


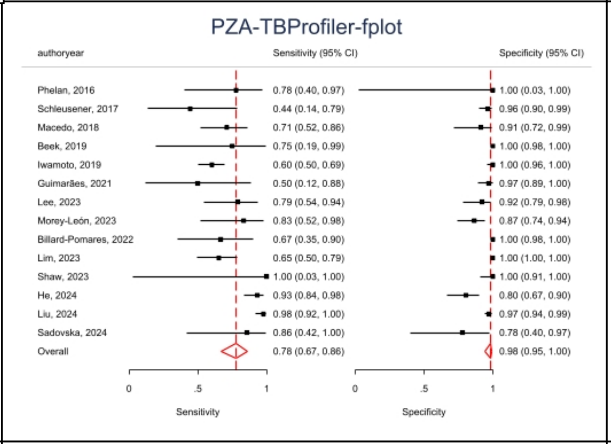


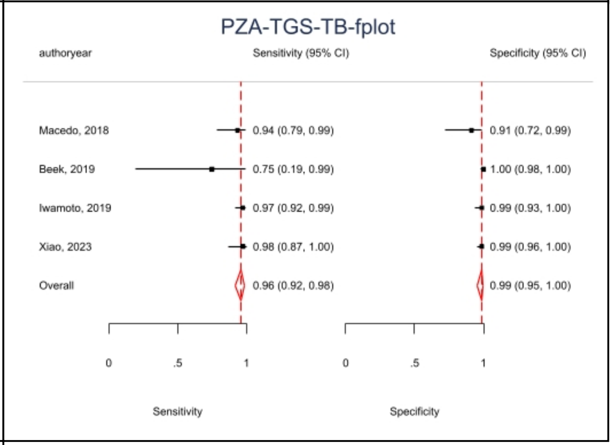


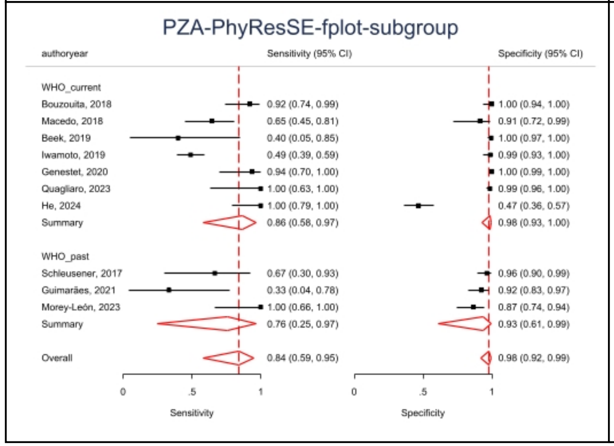


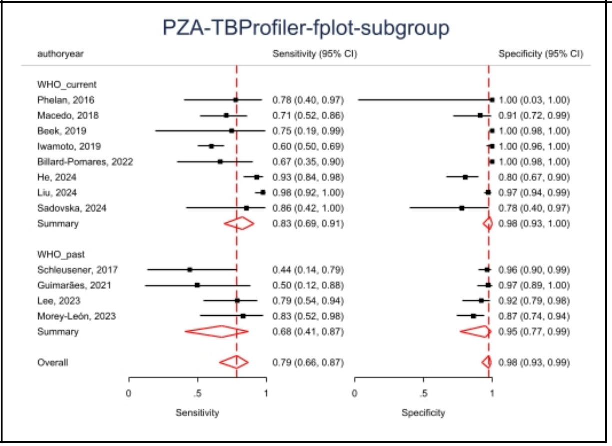

# Streptomycin

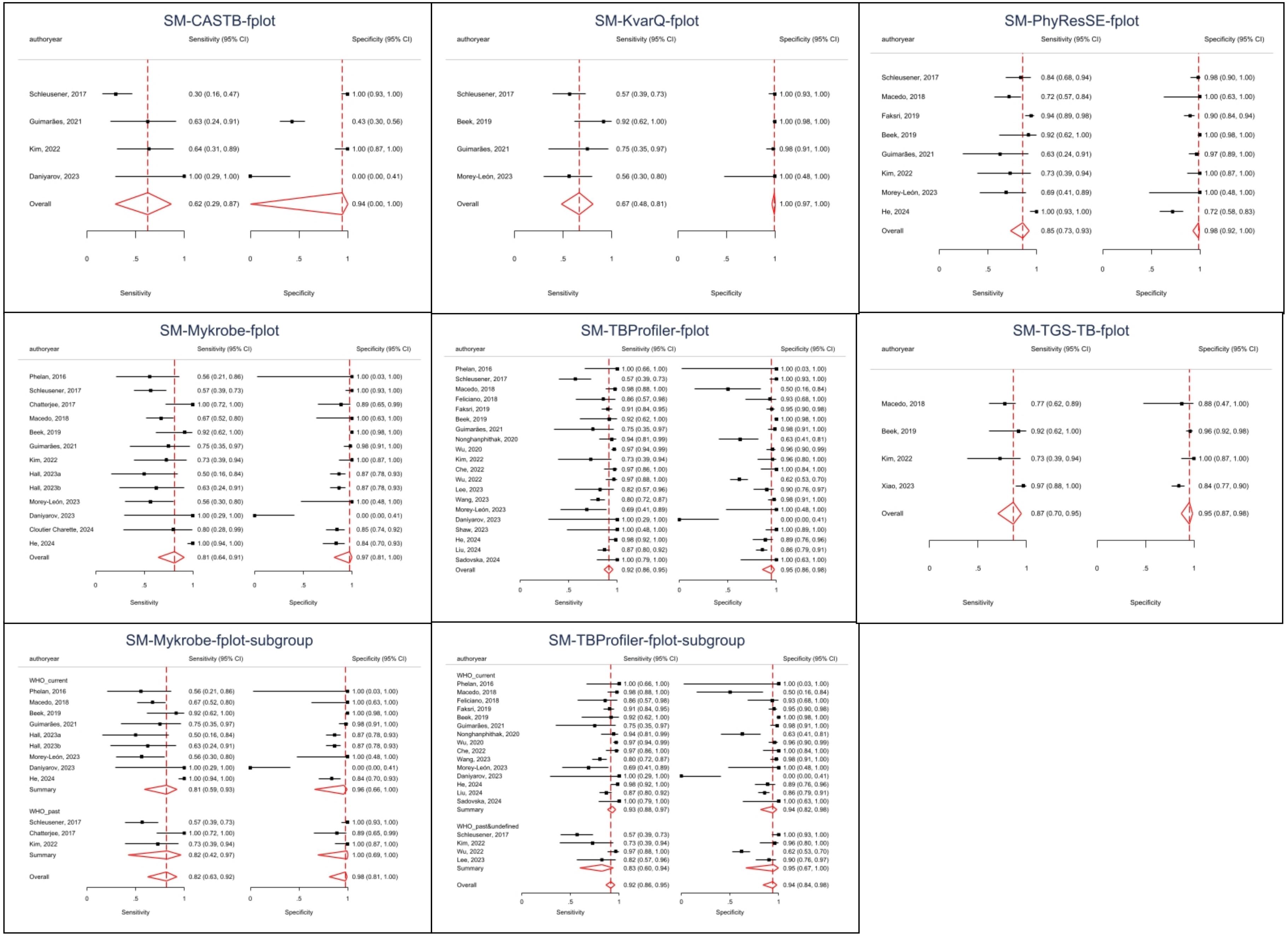

# Amikacin

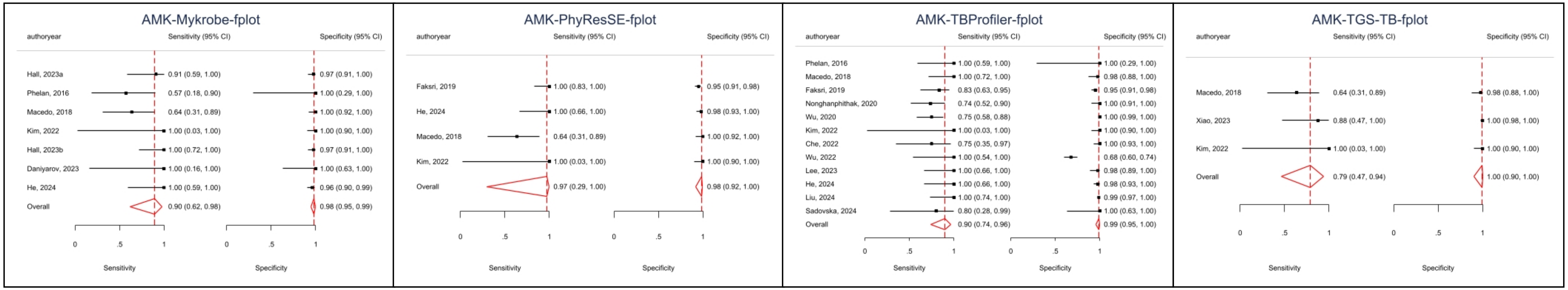


# Capreomycin

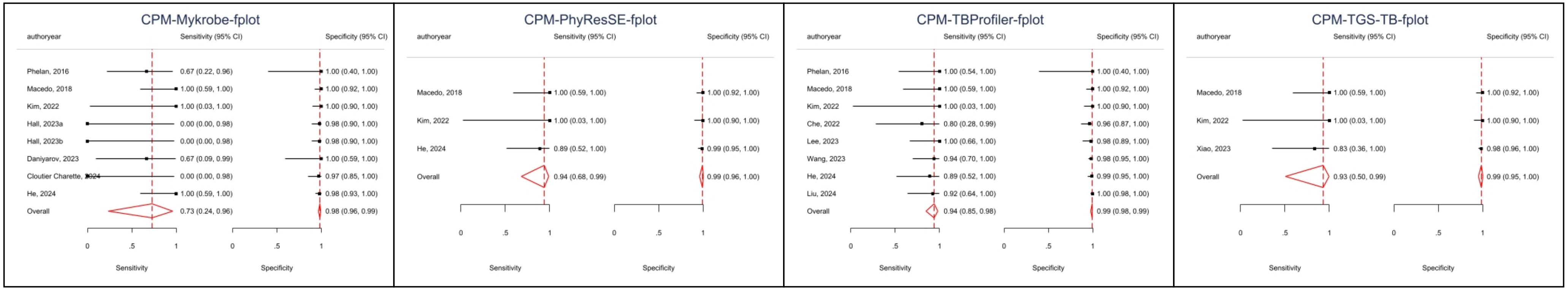


# Kanamycin

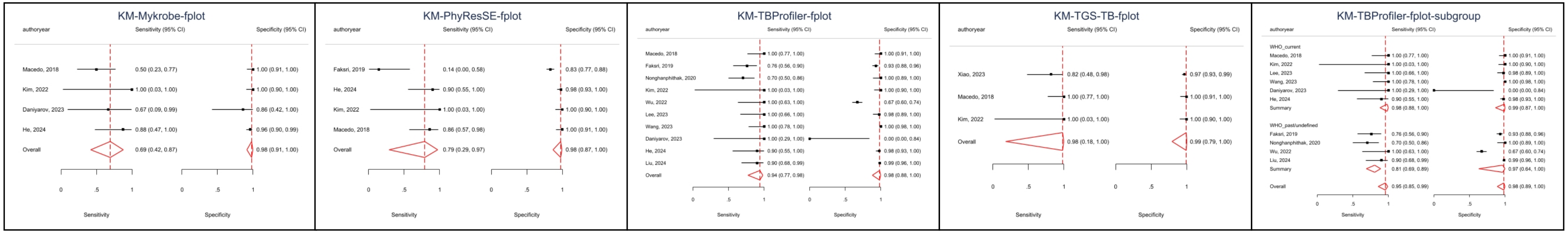

# Levofloxacin

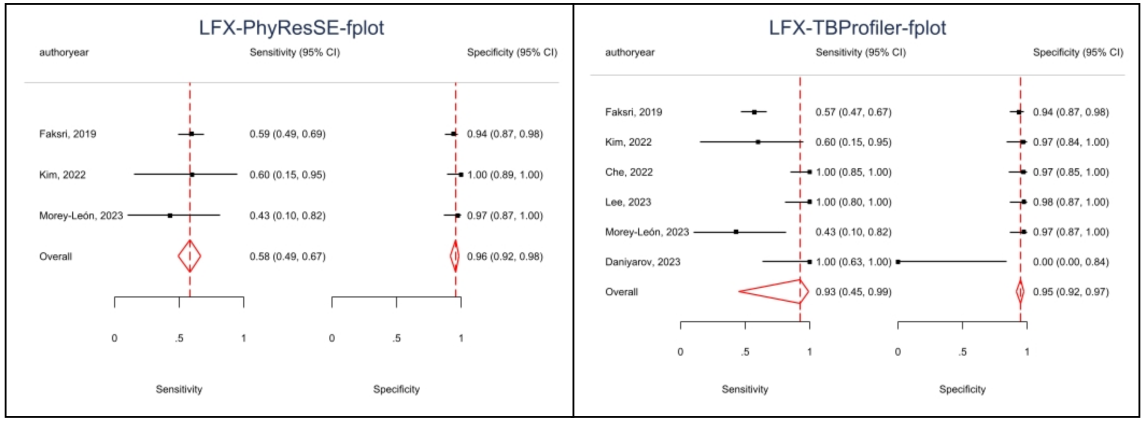


# Moxifloxacin

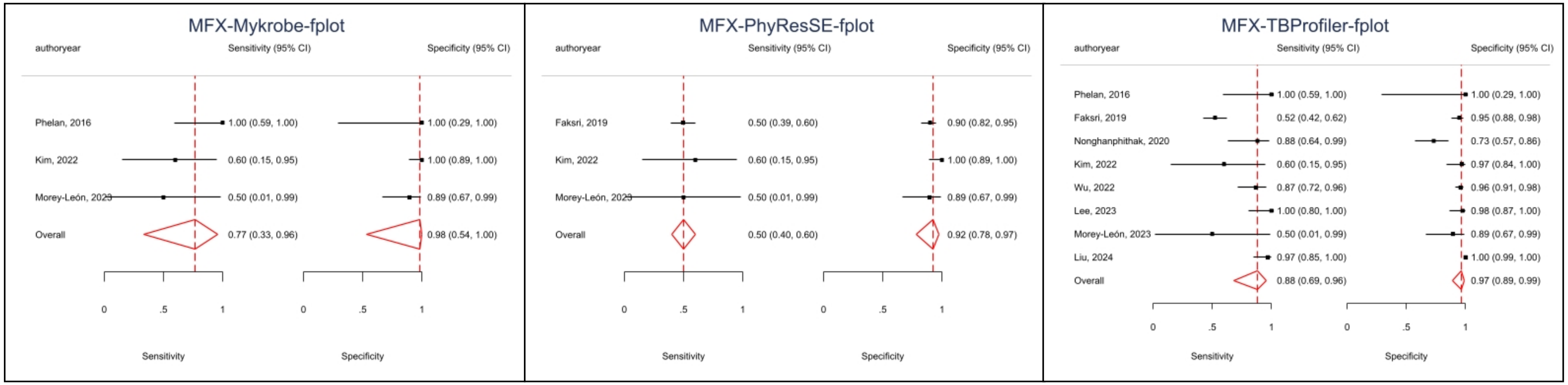


# Ofloxacin

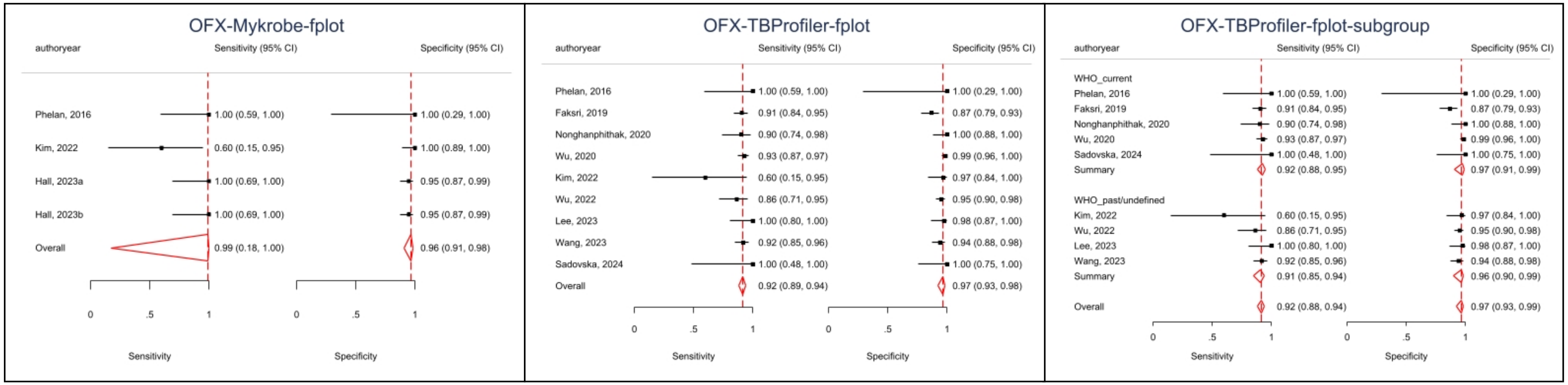

# Ethionamide

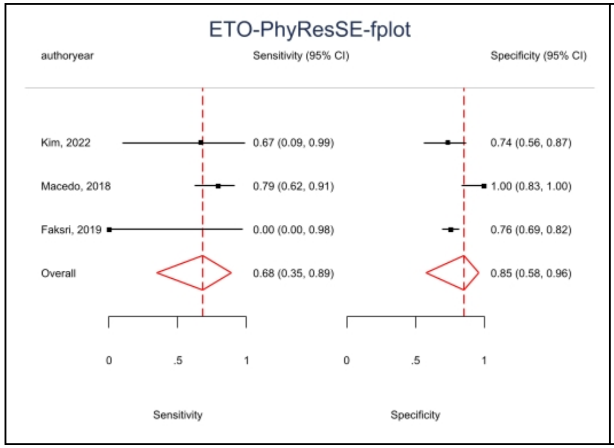


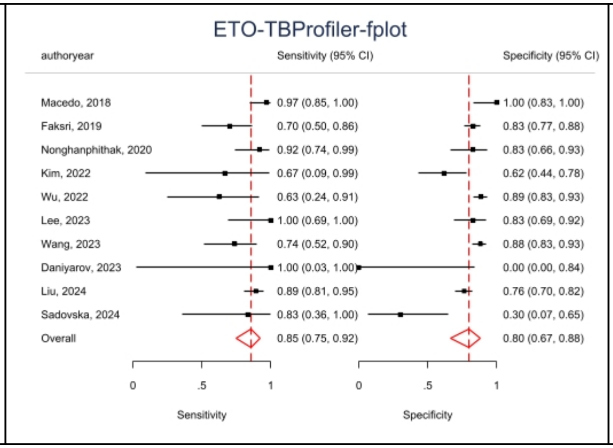


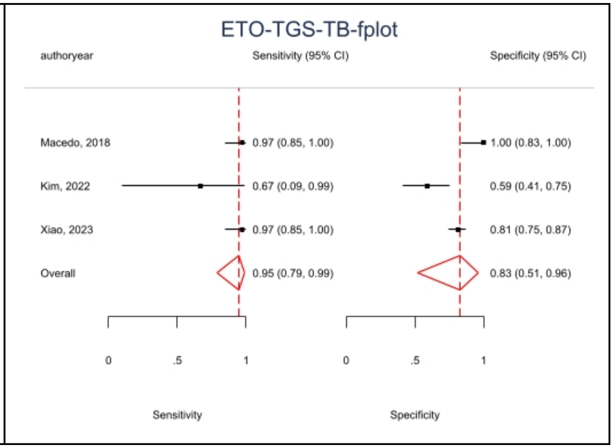


# Prothionamide

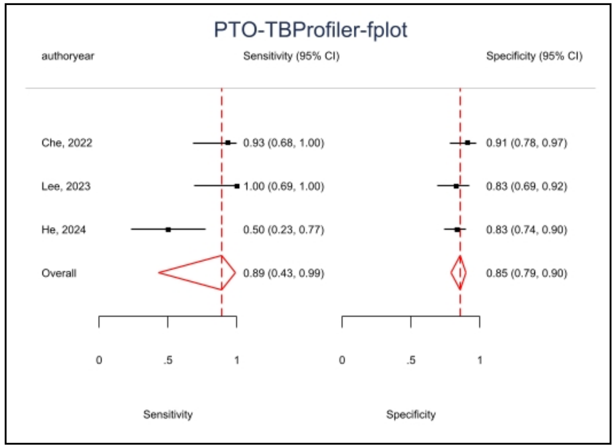


# Para-aminosalicylic acid

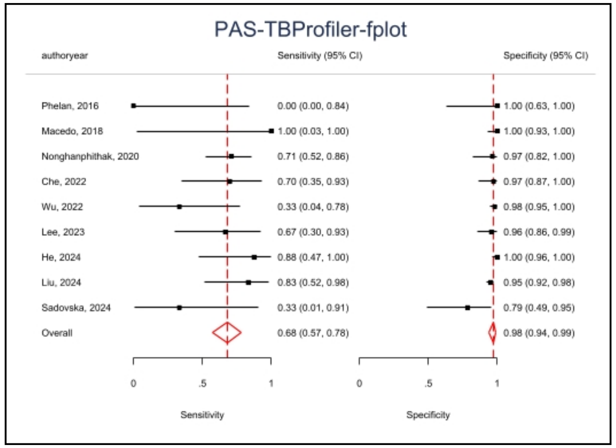

Supplement: S2 Fig — (PDF) [file pgph.0004465.s002.pdf]
