# Supplementary material for: Diagnostic yield of nine user-friendly bioinformatics tools for predicting Mycobacterium tuberculosis drug resistance: A systematic review and network meta-analysis
Source: PLOS Glob Public Health. 2025 Apr 21;5(4):e0004465. doi: 10.1371/journal.pgph.0004465 (PMC12011222; doi:10.1371/journal.pgph.0004465)

Figure 3. Summary receiver operating characteristic curves of all bioinformatics tools in predicting drug resistance to 14 anti-TB drugs.

Isoniazid

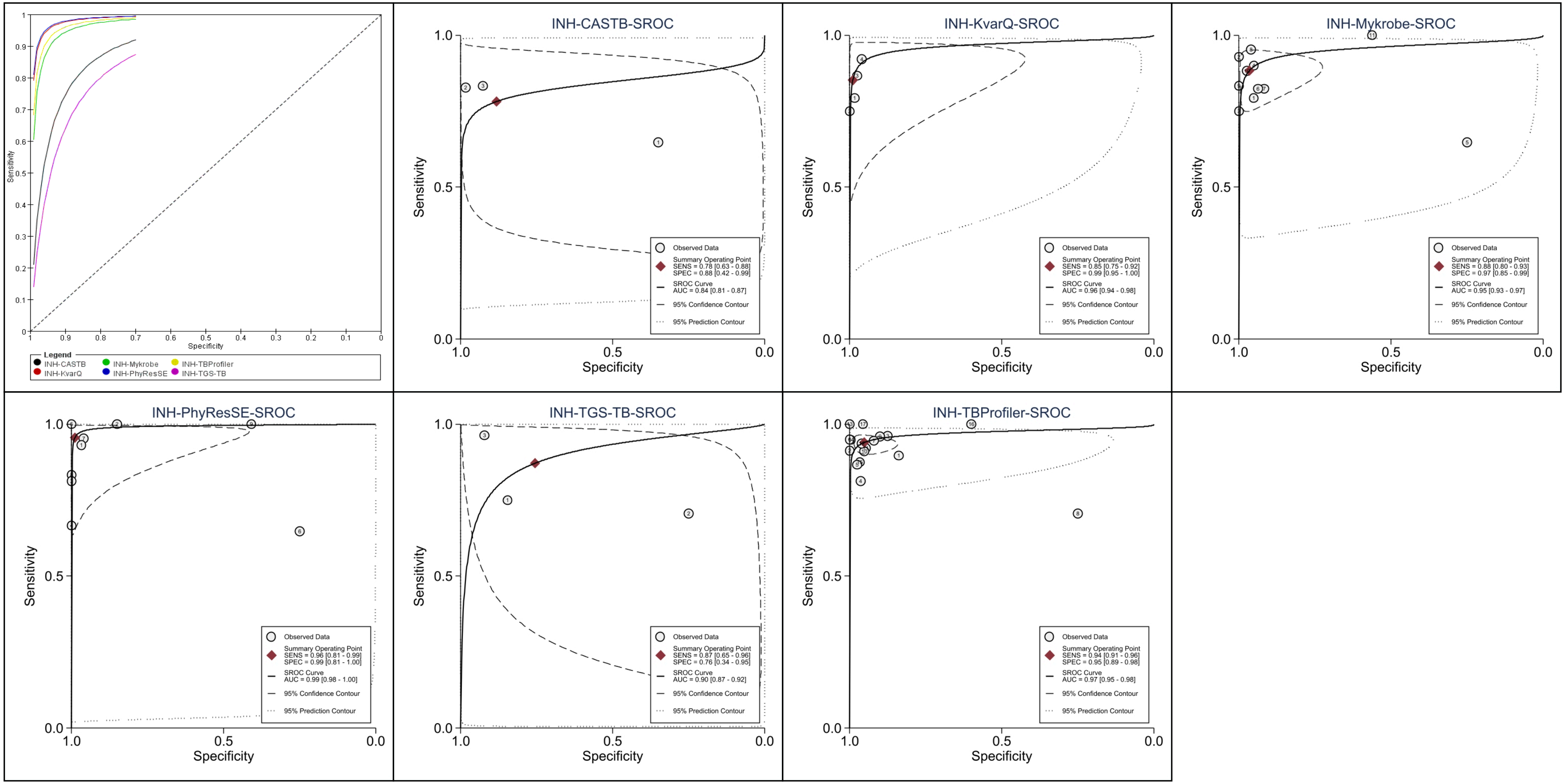

Rifampicin

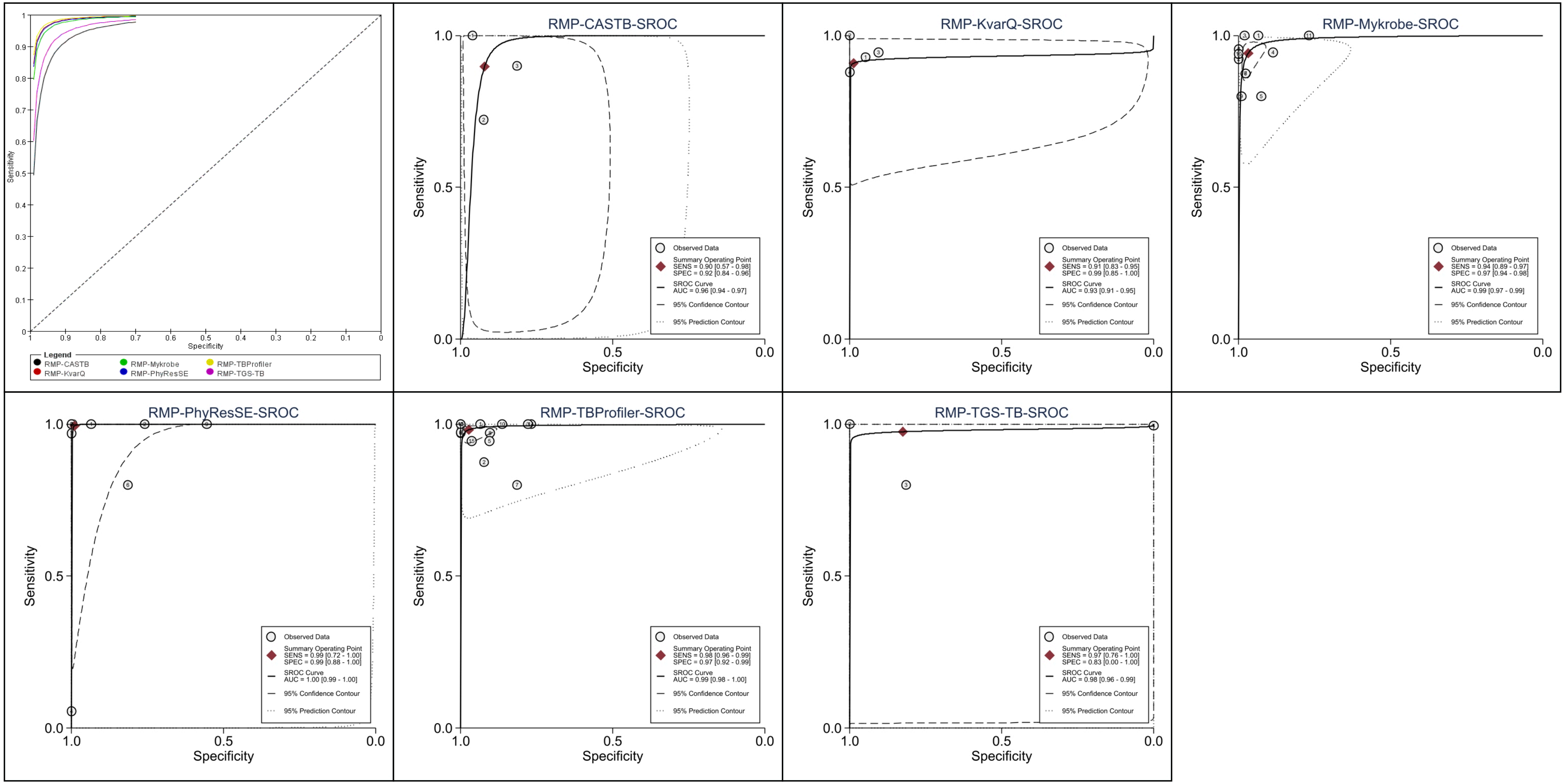

Sensitivity
Specificity
Legend
RMP-CASTB
RMP-KvarQ
RMP-Mykrobe
RMP-PhyResSE
RMP-TBProfiler
RMP-TGS-TB
RMP-CASTB-SROC
Observed Data
Summary Operating Point
SENS = 0.90 [0.57 - 0.98]
SPEC = 0.92 [0.84 - 0.96]
SROC Curve
AUC = 0.96 [0.94 - 0.97]
95% Confidence Contour
95% Prediction Contour
RMP-KvarQ-SROC
Observed Data
Summary Operating Point
SENS = 0.91 [0.83 - 0.95]
SPEC = 0.99 [0.85 - 1.00]
SROC Curve
AUC = 0.93 [0.91 - 0.95]
95% Confidence Contour
95% Prediction Contour
RMP-Mykrobe-SROC
Observed Data
Summary Operating Point
SENS = 0.94 [0.89 - 0.97]
SPEC = 0.97 [0.94 - 0.98]
SROC Curve
AUC = 0.99 [0.97 - 0.99]
95% Confidence Contour
95% Prediction Contour
RMP-PhyResSE-SROC
Observed Data
Summary Operating Point
SENS = 0.99 [0.72 - 1.00]
SPEC = 0.99 [0.88 - 1.00]
SROC Curve
AUC = 1.00 [0.99 - 1.00]
95% Confidence Contour
95% Prediction Contour
RMP-TBProfiler-SROC
Observed Data
Summary Operating Point
SENS = 0.98 [0.96 - 0.99]
SPEC = 0.97 [0.92 - 0.99]
SROC Curve
AUC = 0.99 [0.98 - 1.00]
95% Confidence Contour
95% Prediction Contour
RMP-TGS-TB-SROC
Observed Data
Summary Operating Point
SENS = 0.97 [0.76 - 1.00]
SPEC = 0.83 [0.00 - 1.00]
SROC Curve
AUC = 0.98 [0.96 - 0.99]
95% Confidence Contour
95% Prediction Contour

Ethambutol

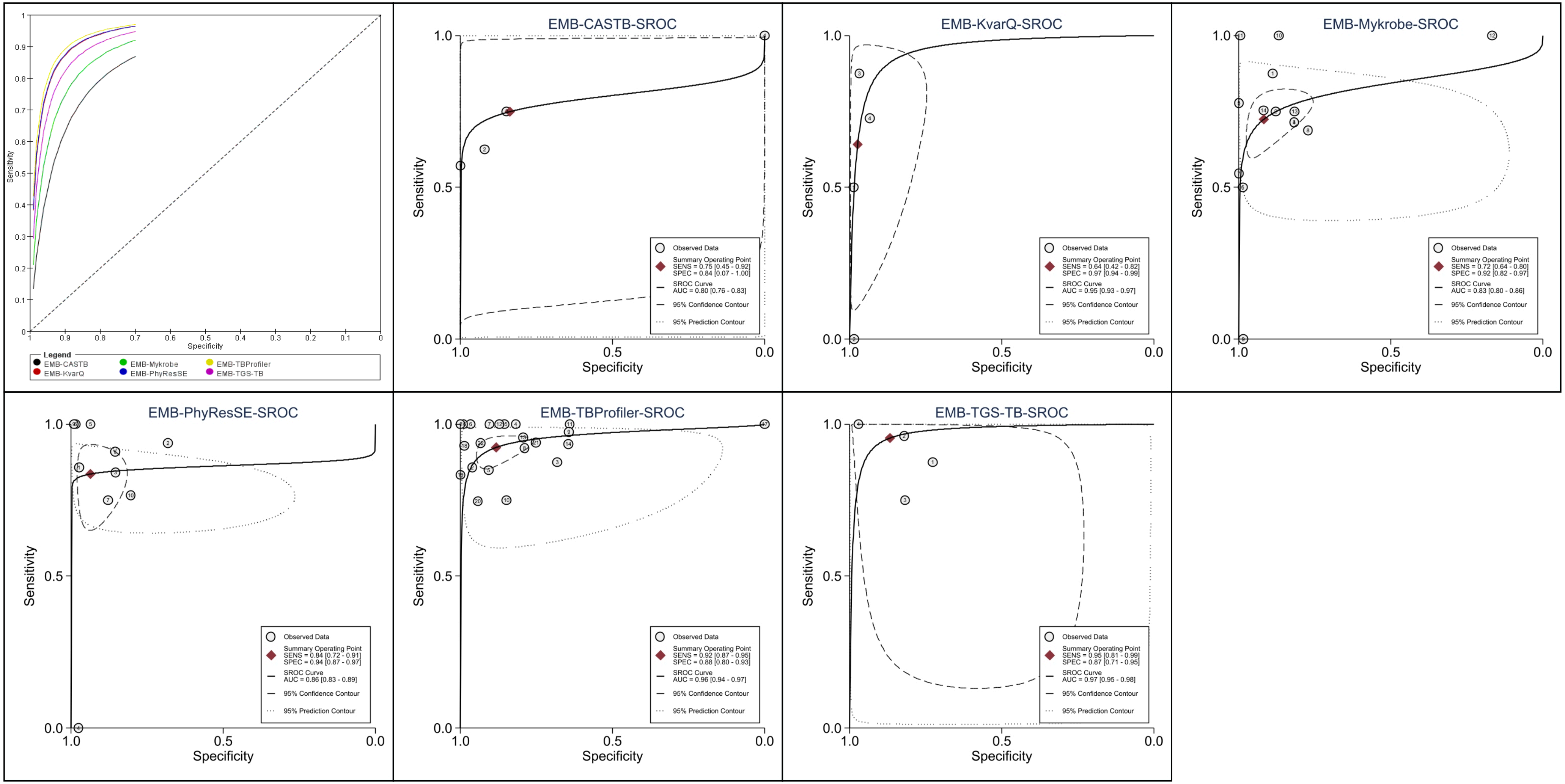

Sensitivity
Specificity
Legend
EMB-CASTB
EMB-KvarQ
EMB-Mykrobe
EMB-PhyResSE
EMB-TBProfiler
EMB-TGS-TB
EMB-CASTB-SROC
Observed Data
Summary Operating Point
SENS = 0.75 [0.45 - 0.92]
SPEC = 0.84 [0.07 - 1.00]
SROC Curve
AUC = 0.80 [0.76 - 0.83]
95% Confidence Contour
95% Prediction Contour
EMB-KvarQ-SROC
Observed Data
Summary Operating Point
SENS = 0.64 [0.42 - 0.82]
SPEC = 0.97 [0.94 - 0.99]
SROC Curve
AUC = 0.95 [0.93 - 0.97]
95% Confidence Contour
95% Prediction Contour
EMB-Mykrobe-SROC
Observed Data
Summary Operating Point
SENS = 0.72 [0.64 - 0.80]
SPEC = 0.92 [0.82 - 0.97]
SROC Curve
AUC = 0.83 [0.80 - 0.86]
95% Confidence Contour
95% Prediction Contour
EMB-PhyResSE-SROC
Observed Data
Summary Operating Point
SENS = 0.84 [0.72 - 0.91]
SPEC = 0.94 [0.87 - 0.97]
SROC Curve
AUC = 0.86 [0.83 - 0.89]
95% Confidence Contour
95% Prediction Contour
EMB-TBProfiler-SROC
Observed Data
Summary Operating Point
SENS = 0.92 [0.87 - 0.95]
SPEC = 0.88 [0.80 - 0.93]
SROC Curve
AUC = 0.96 [0.94 - 0.97]
95% Confidence Contour
95% Prediction Contour
EMB-TGS-TB-SROC
Observed Data
Summary Operating Point
SENS = 0.95 [0.81 - 0.99]
SPEC = 0.87 [0.71 - 0.95]
SROC Curve
AUC = 0.97 [0.95 - 0.98]
95% Confidence Contour
95% Prediction Contour

Pyrazinamide

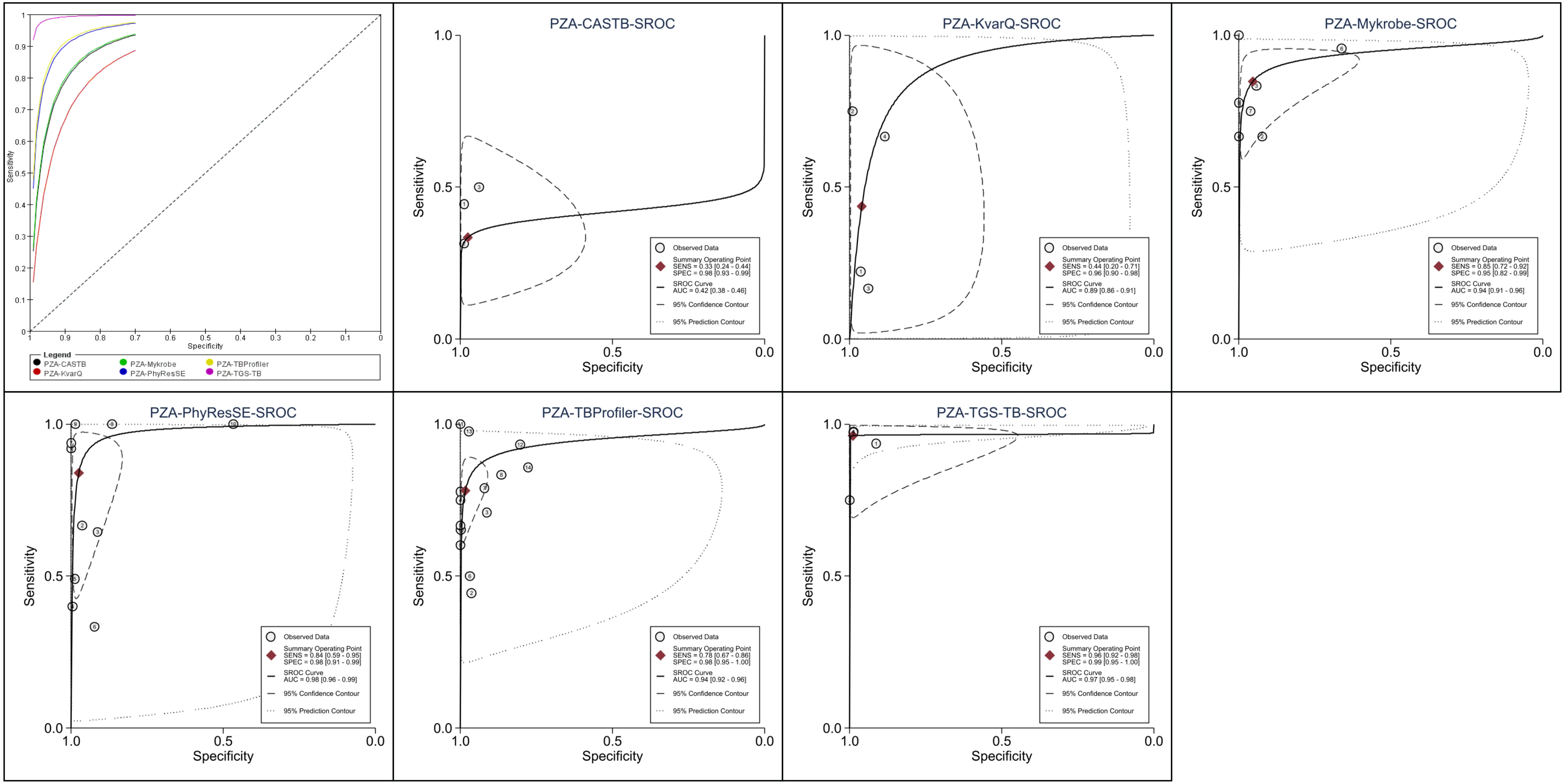

Streptomycin

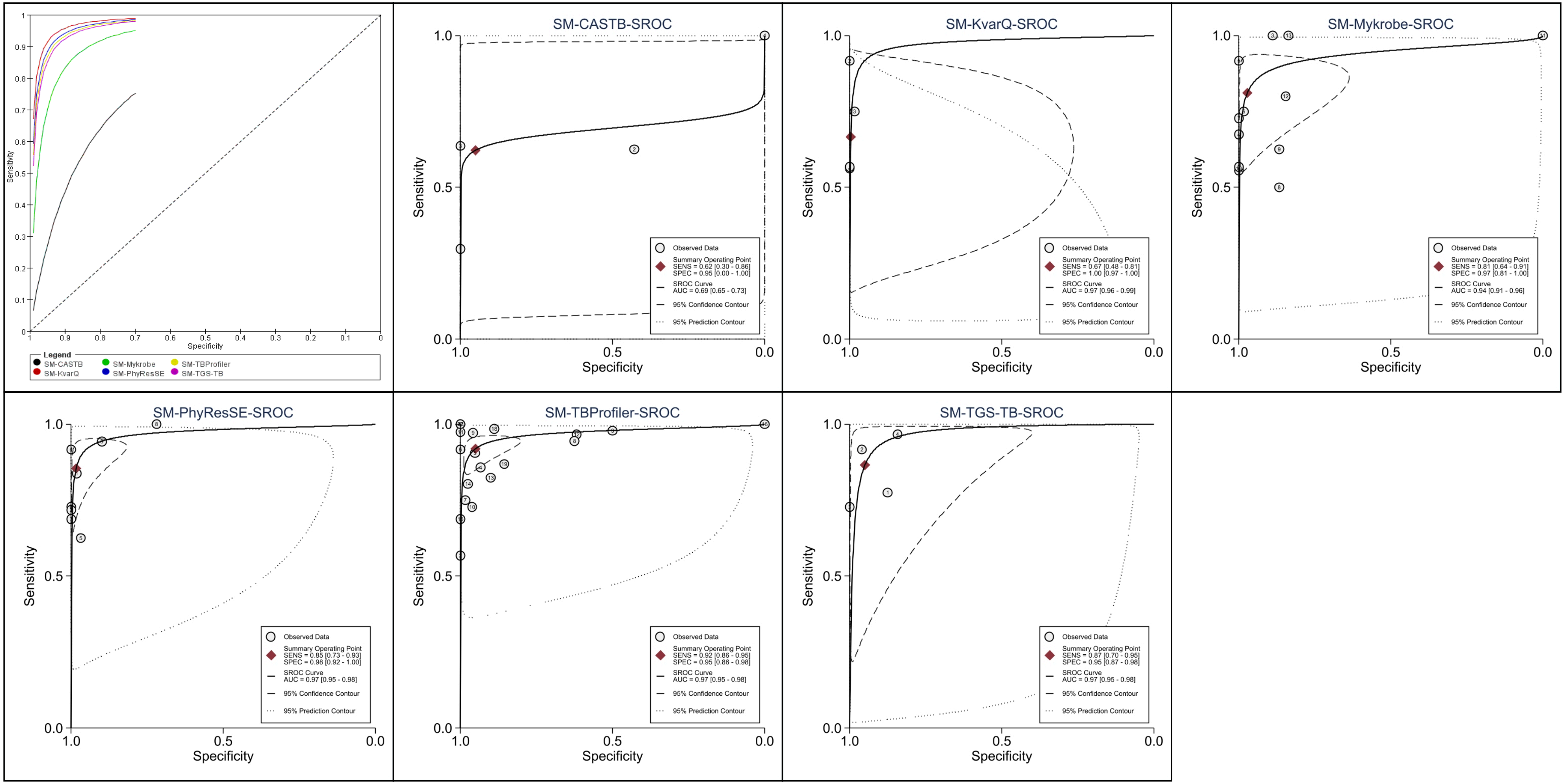

Amikacin

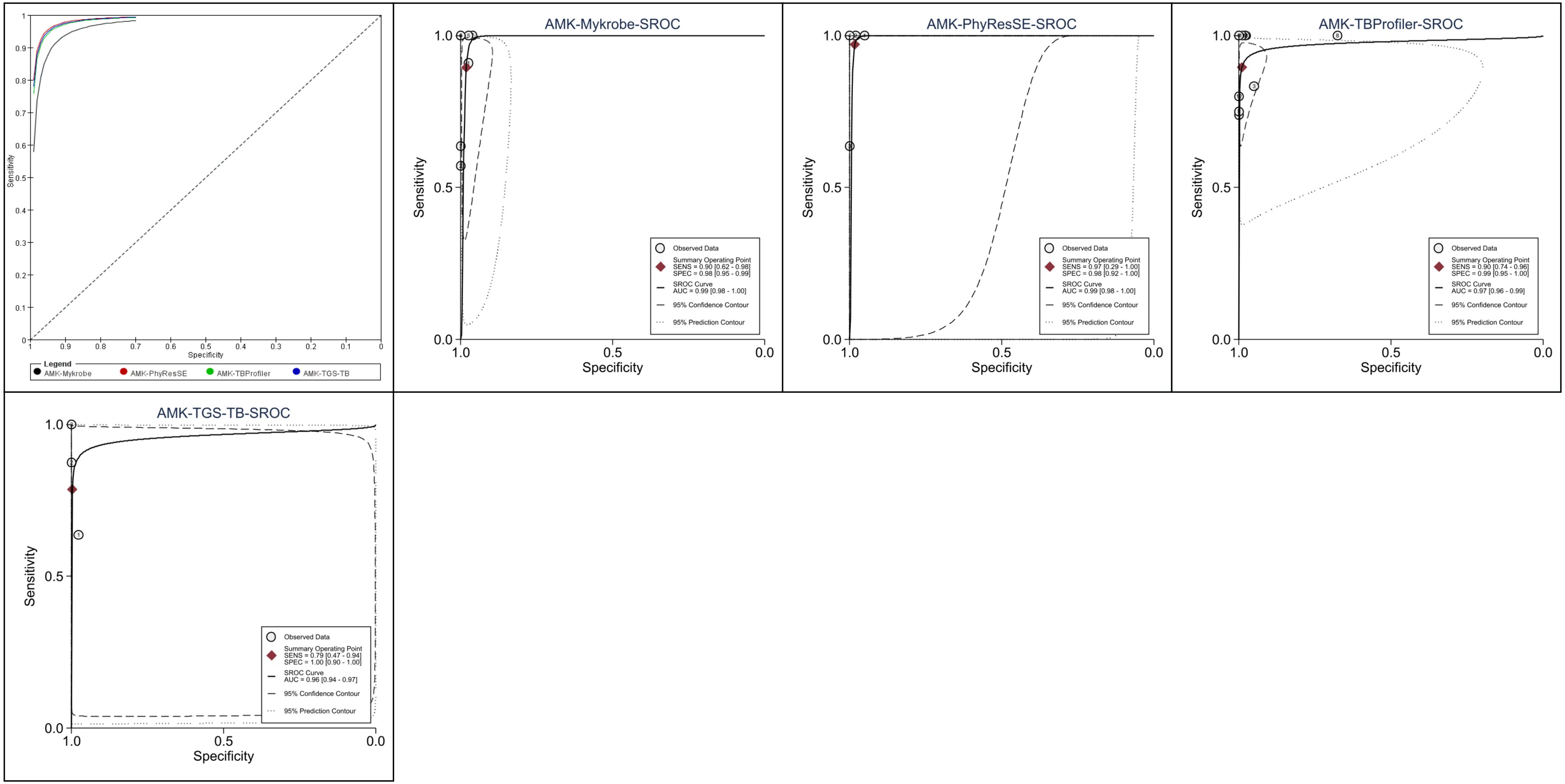
Sensitivity
Specificity
Legend
AMK-Mykrobe
AMK-PhyResSE
AMK-TBProfiler
AMK-TGS-TB
AMK-Mykrobe-SROC
Observed Data
Summary Operating Point
SENS = 0.90 [0.62 - 0.98]
SPEC = 0.98 [0.95 - 0.99]
SROC Curve
AUC = 0.99 [0.98 - 1.00]
95% Confidence Contour
95% Prediction Contour
AMK-PhyResSE-SROC
Observed Data
Summary Operating Point
SENS = 0.97 [0.29 - 1.00]
SPEC = 0.98 [0.92 - 1.00]
SROC Curve
AUC = 0.99 [0.98 - 1.00]
95% Confidence Contour
95% Prediction Contour
AMK-TBProfiler-SROC
Observed Data
Summary Operating Point
SENS = 0.90 [0.74 - 0.96]
SPEC = 0.99 [0.95 - 1.00]
SROC Curve
AUC = 0.97 [0.96 - 0.99]
95% Confidence Contour
95% Prediction Contour
AMK-TGS-TB-SROC
Observed Data
Summary Operating Point
SENS = 0.79 [0.47 - 0.94]
SPEC = 1.00 [0.90 - 1.00]
SROC Curve
AUC = 0.96 [0.94 - 0.97]
95% Confidence Contour
95% Prediction Contour

Capreomycin

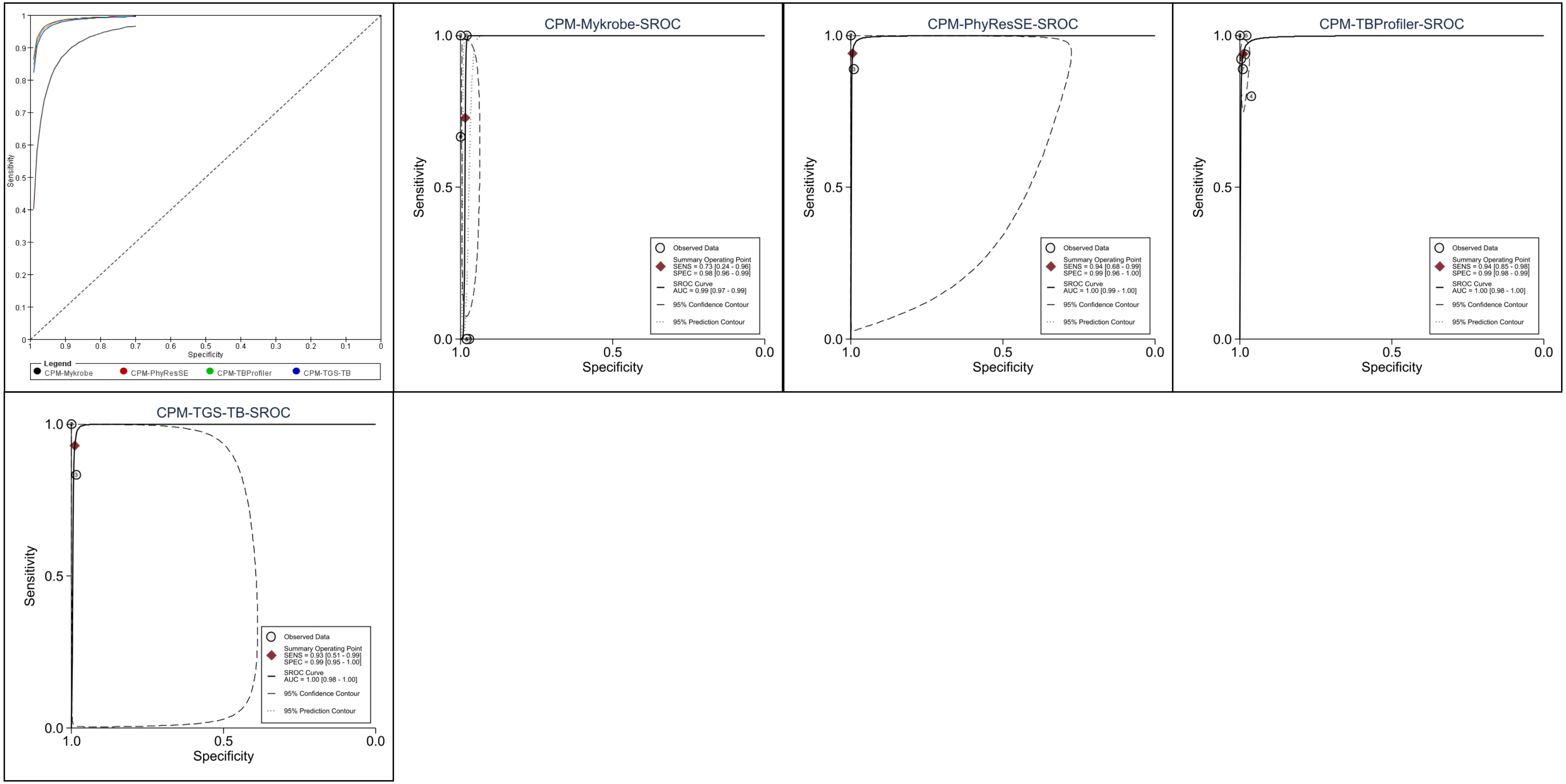

Kanamycin

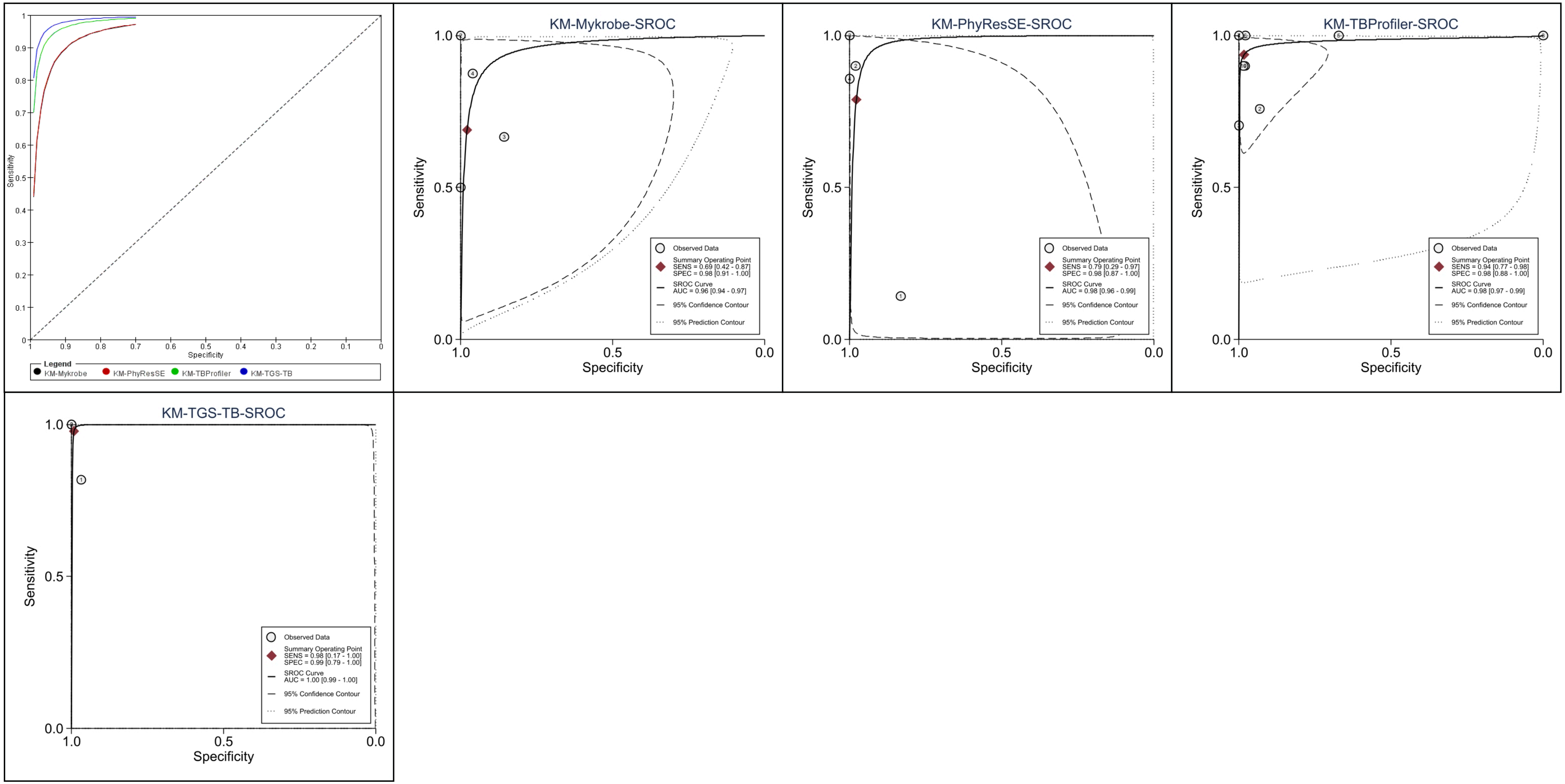

Sensitivity
Specificity
Legend
KM-Mykrobe
KM-PhyResSE
KM-TBProfiler
KM-TGS-TB
KM-Mykrobe-SROC
Observed Data
Summary Operating Point
SENS = 0.69 [0.42 - 0.87]
SPEC = 0.98 [0.91 - 1.00]
SROC Curve
AUC = 0.96 [0.94 - 0.97]
95% Confidence Contour
95% Prediction Contour
KM-PhyResSE-SROC
Observed Data
Summary Operating Point
SENS = 0.79 [0.29 - 0.97]
SPEC = 0.98 [0.87 - 1.00]
SROC Curve
AUC = 0.98 [0.96 - 0.99]
95% Confidence Contour
95% Prediction Contour
KM-TBProfiler-SROC
Observed Data
Summary Operating Point
SENS = 0.94 [0.77 - 0.98]
SPEC = 0.98 [0.88 - 1.00]
SROC Curve
AUC = 0.98 [0.97 - 0.99]
95% Confidence Contour
95% Prediction Contour
KM-TGS-TB-SROC
Observed Data
Summary Operating Point
SENS = 0.98 [0.17 - 1.00]
SPEC = 0.99 [0.79 - 1.00]
SROC Curve
AUC = 1.00 [0.99 - 1.00]
95% Confidence Contour
95% Prediction Contour

Levofloxacin

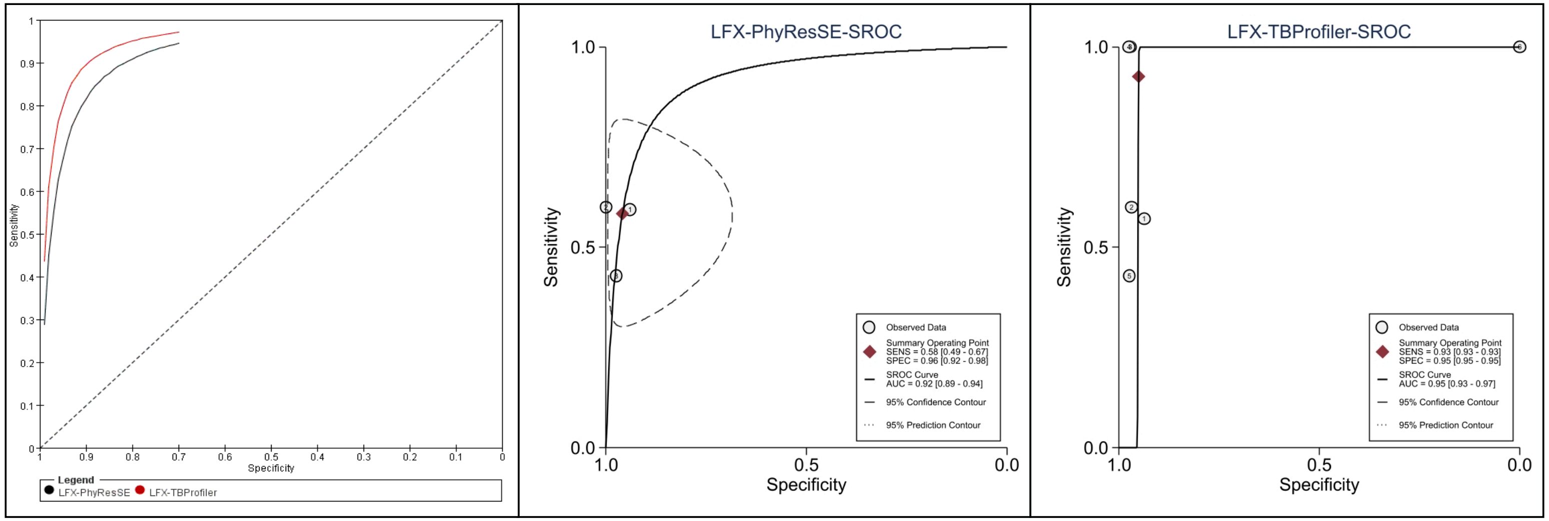

Moxifloxacin

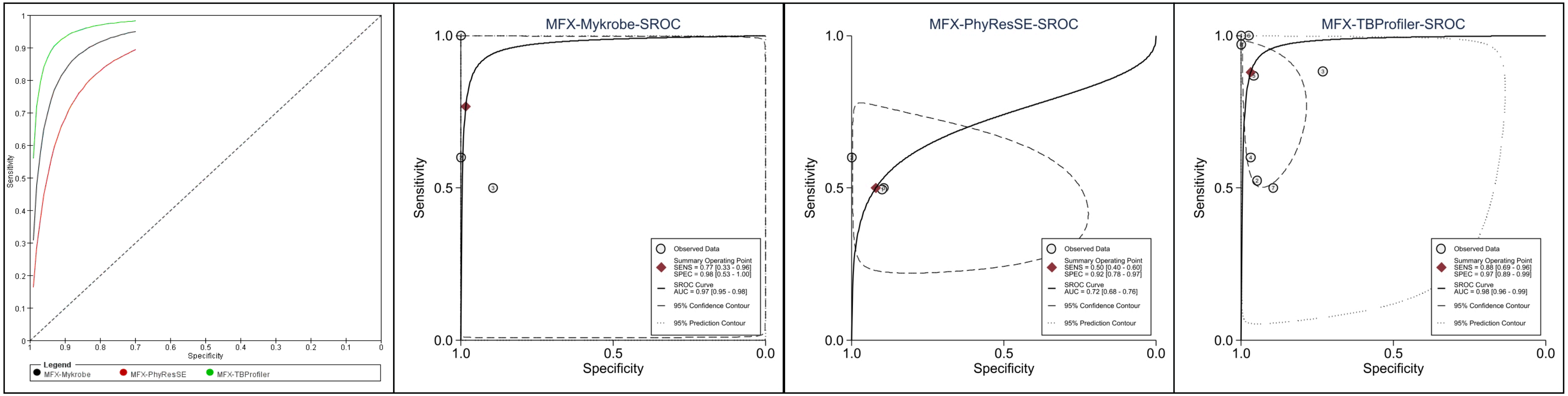

Ofloxacin

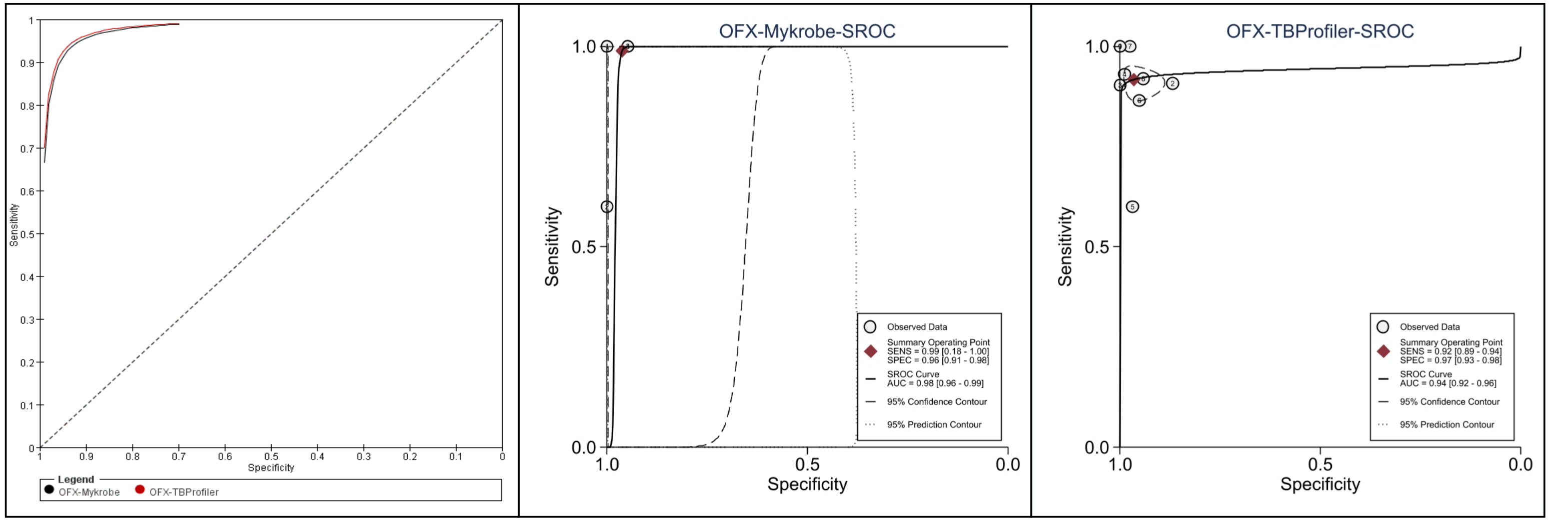

Ethionamide

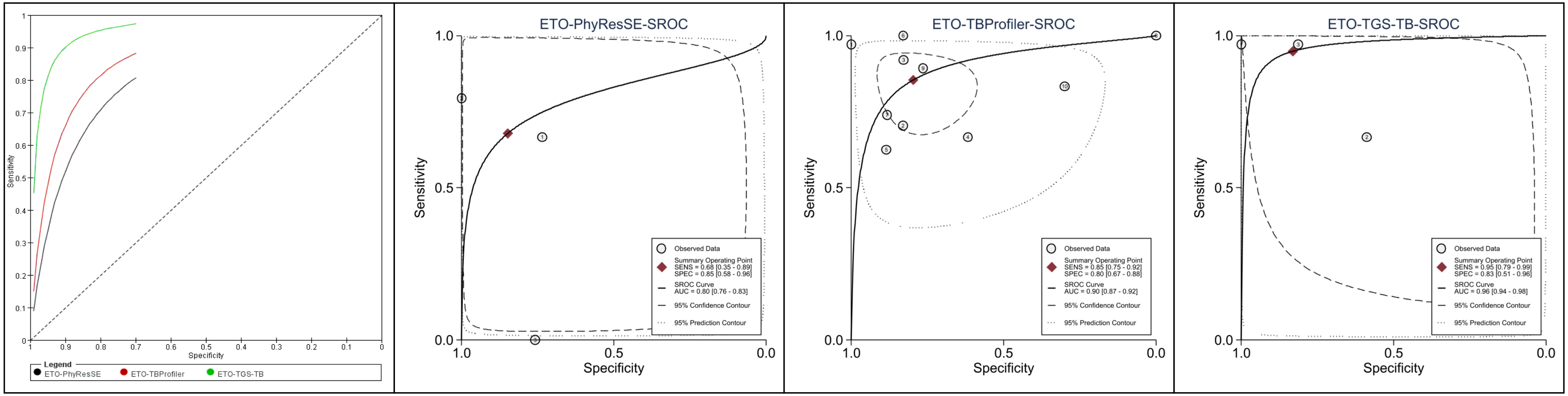

Prothionamide

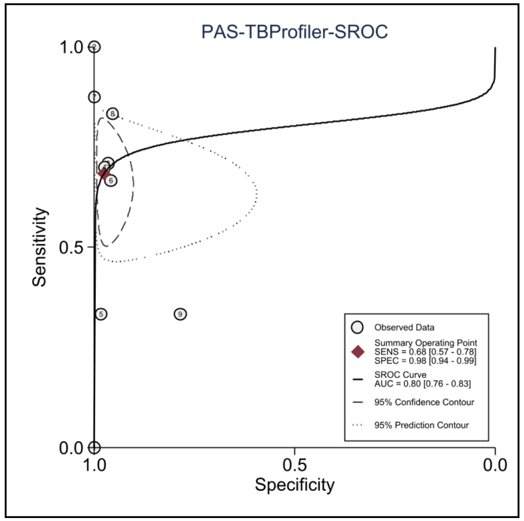

Para-aminosalicylic acid

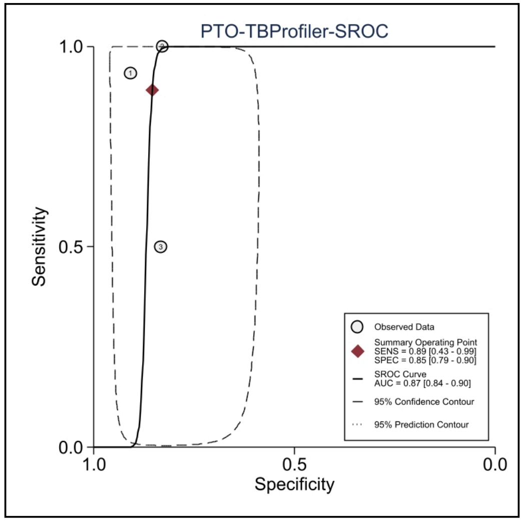

Supplement: S3 Fig — (PDF) [file pgph.0004465.s003.pdf]
