# Supplementary material for: Diagnostic yield of nine user-friendly bioinformatics tools for predicting Mycobacterium tuberculosis drug resistance: A systematic review and network meta-analysis
Source: PLOS Glob Public Health. 2025 Apr 21;5(4):e0004465. doi: 10.1371/journal.pgph.0004465 (PMC12011222; doi:10.1371/journal.pgph.0004465)

Figure 4. Publication bias of included studies assessed using Deek's funnel plot, , analyzed separately for each drug.

Isoniazid

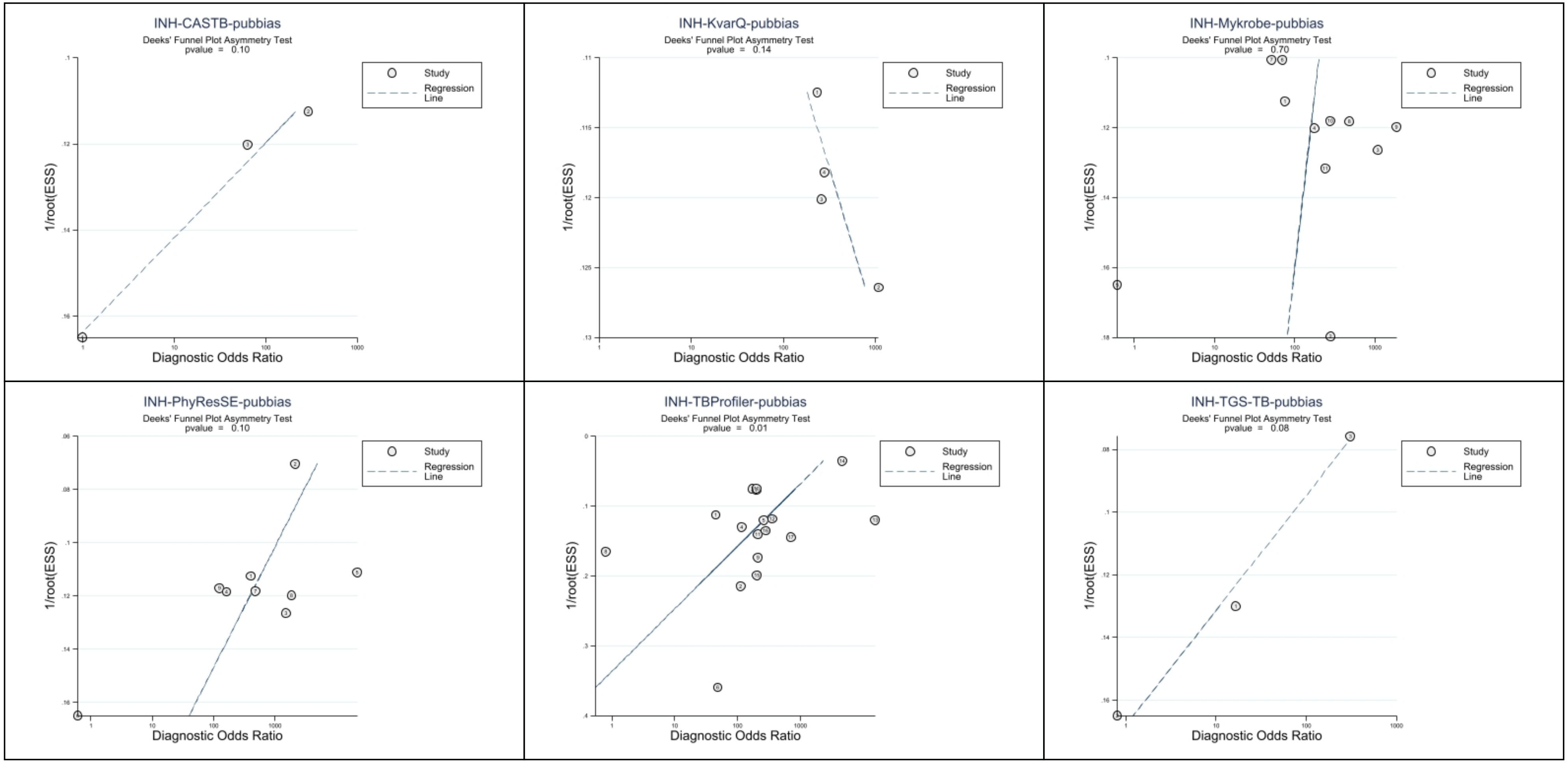

Rifampicin

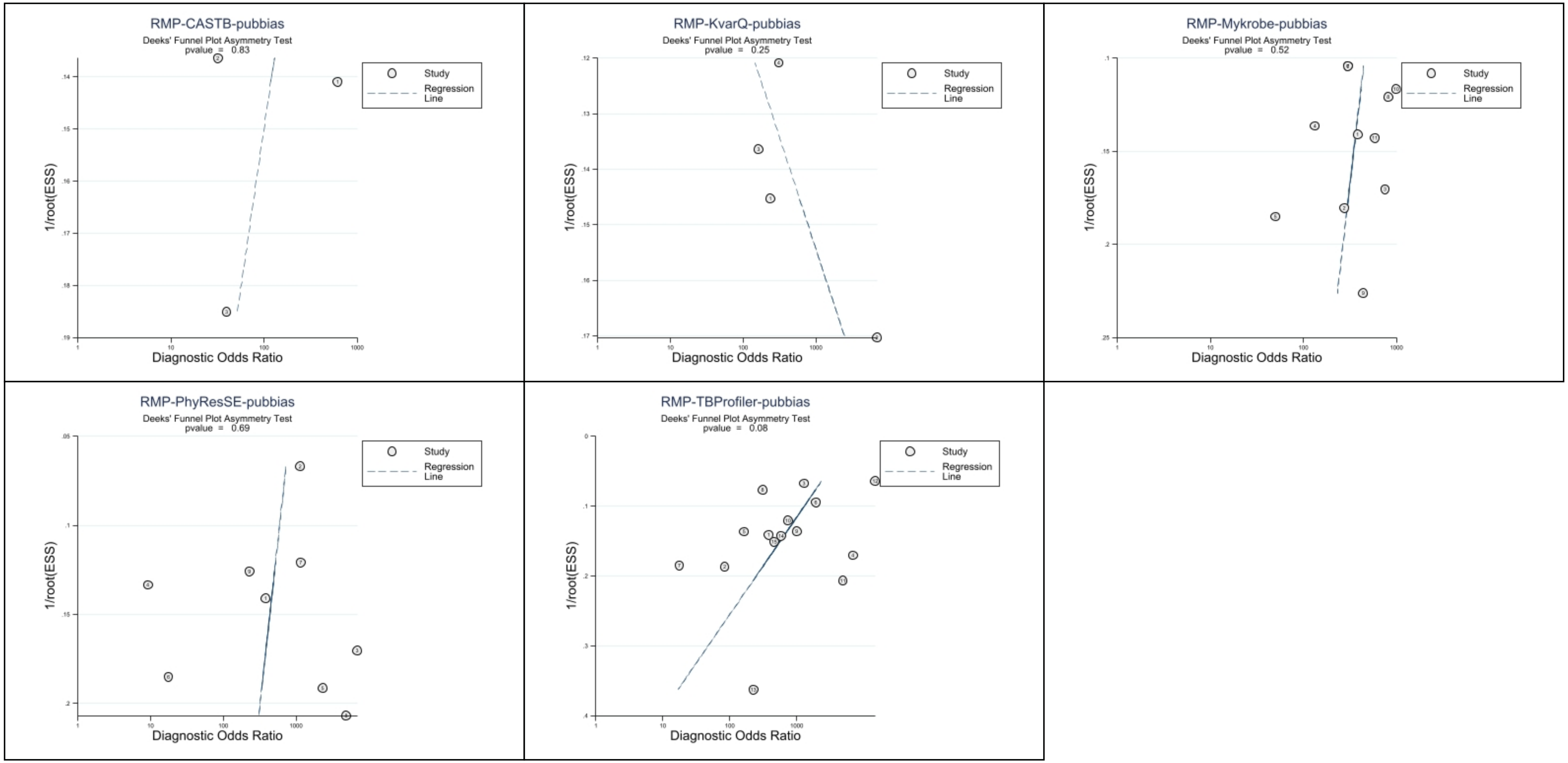
RMP-CASTB-pubbias
Deeks' Funnel Plot Asymmetry Test
pvalue = 0.83
1/root(ESS)
Diagnostic Odds Ratio
Study
Regression Line
RMP-KvarQ-pubbias
Deeks' Funnel Plot Asymmetry Test
pvalue = 0.25
1/root(ESS)
Diagnostic Odds Ratio
Study
Regression Line
RMP-Mykrobe-pubbias
Deeks' Funnel Plot Asymmetry Test
pvalue = 0.52
1/root(ESS)
Diagnostic Odds Ratio
Study
Regression Line
RMP-PhyResSE-pubbias
Deeks' Funnel Plot Asymmetry Test
pvalue = 0.69
1/root(ESS)
Diagnostic Odds Ratio
Study
Regression Line
RMP-TBProfiler-pubbias
Deeks' Funnel Plot Asymmetry Test
pvalue = 0.08
1/root(ESS)
Diagnostic Odds Ratio
Study
Regression Line

Ethambutol

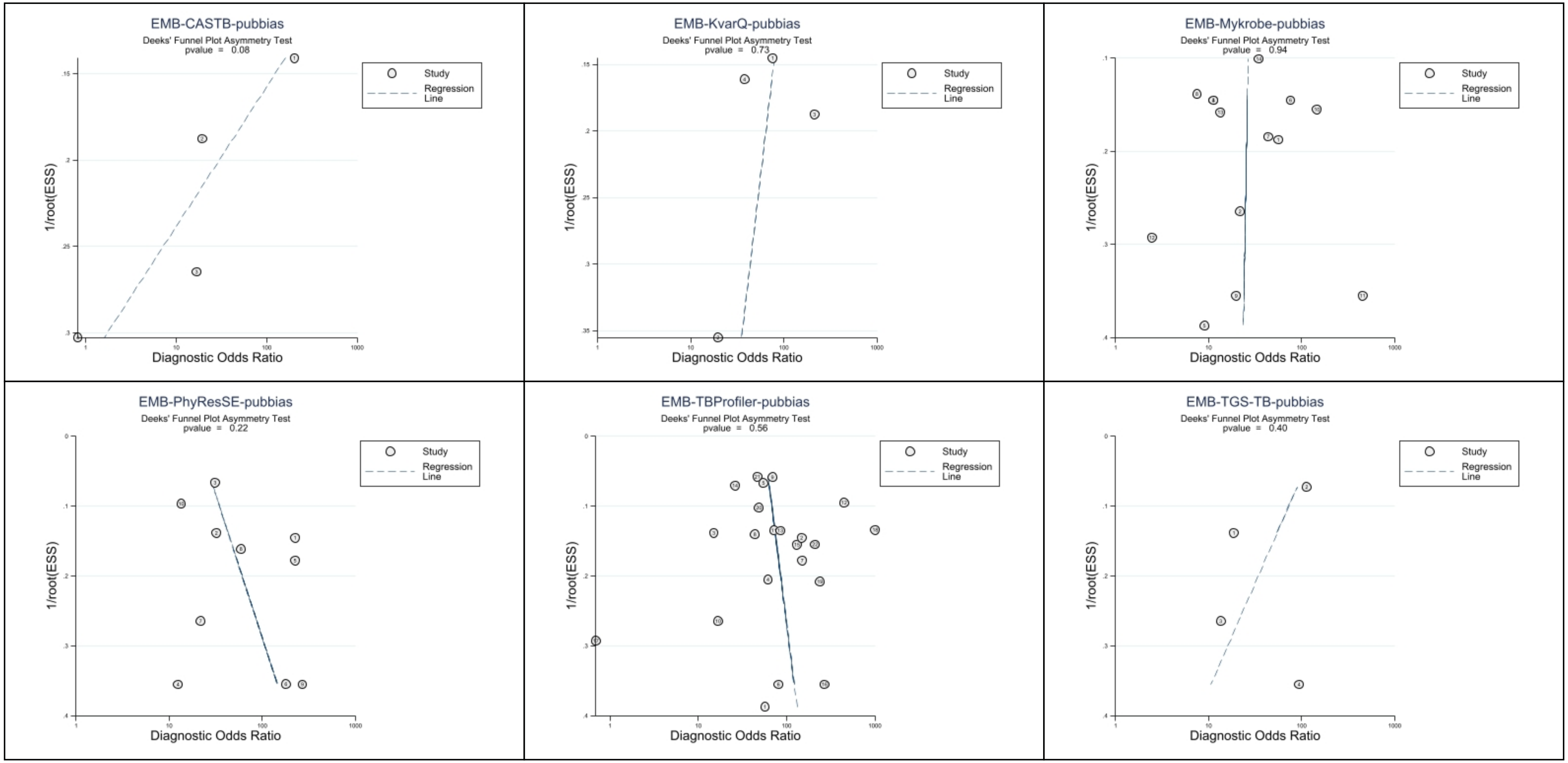
EMB-CASTB-pubbias
Deeks' Funnel Plot Asymmetry Test
pvalue = 0.08
EMB-KvarQ-pubbias
Deeks' Funnel Plot Asymmetry Test
pvalue = 0.73
EMB-Mykrobe-pubbias
Deeks' Funnel Plot Asymmetry Test
pvalue = 0.94
EMB-PhyResSE-pubbias
Deeks' Funnel Plot Asymmetry Test
pvalue = 0.22
EMB-TBProfiler-pubbias
Deeks' Funnel Plot Asymmetry Test
pvalue = 0.56
EMB-TGS-TB-pubbias
Deeks' Funnel Plot Asymmetry Test
pvalue = 0.40
Study
Regression Line
1/root(ESS)
Diagnostic Odds Ratio

Pyrazinamide

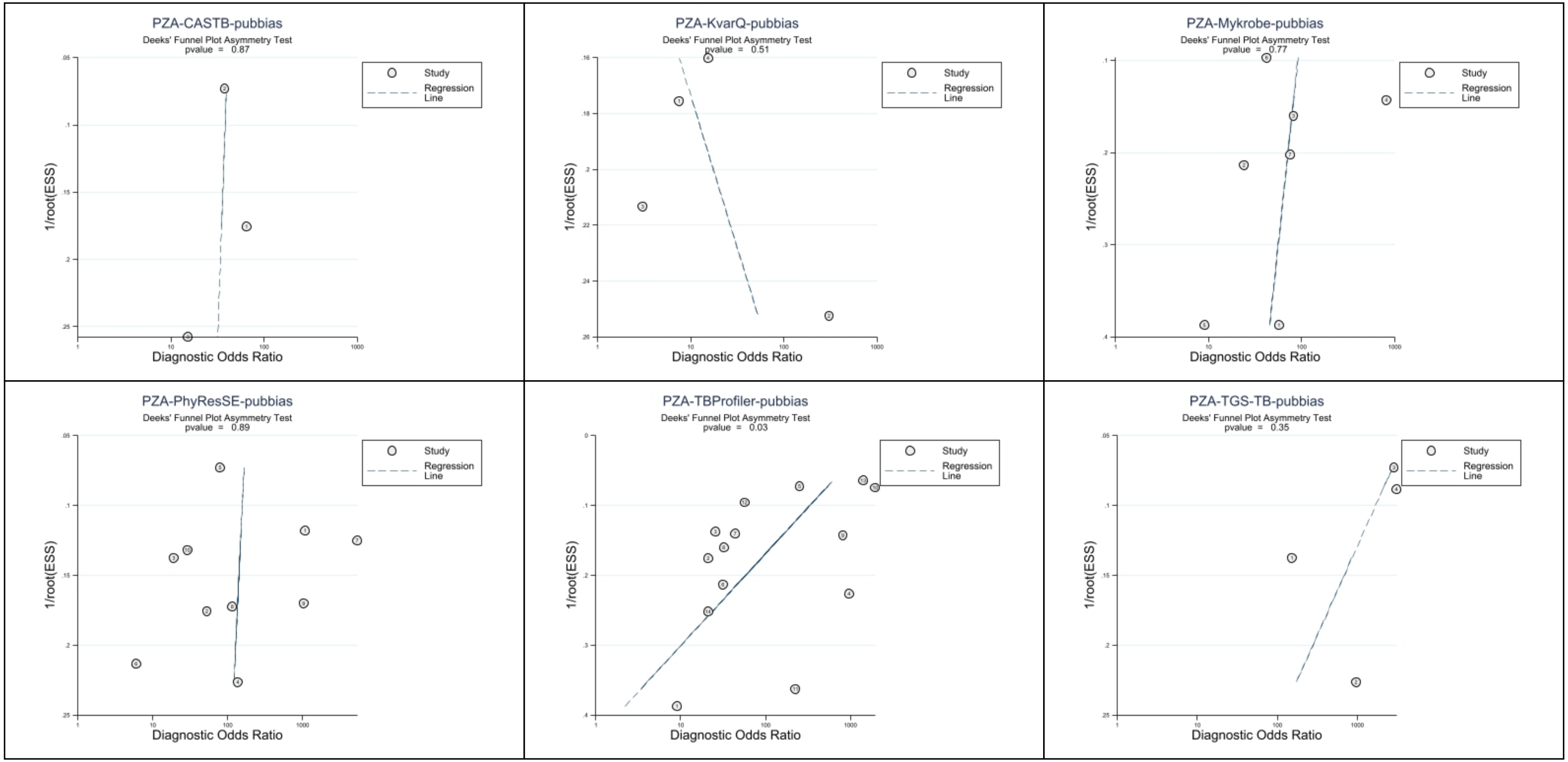

Streptomycin

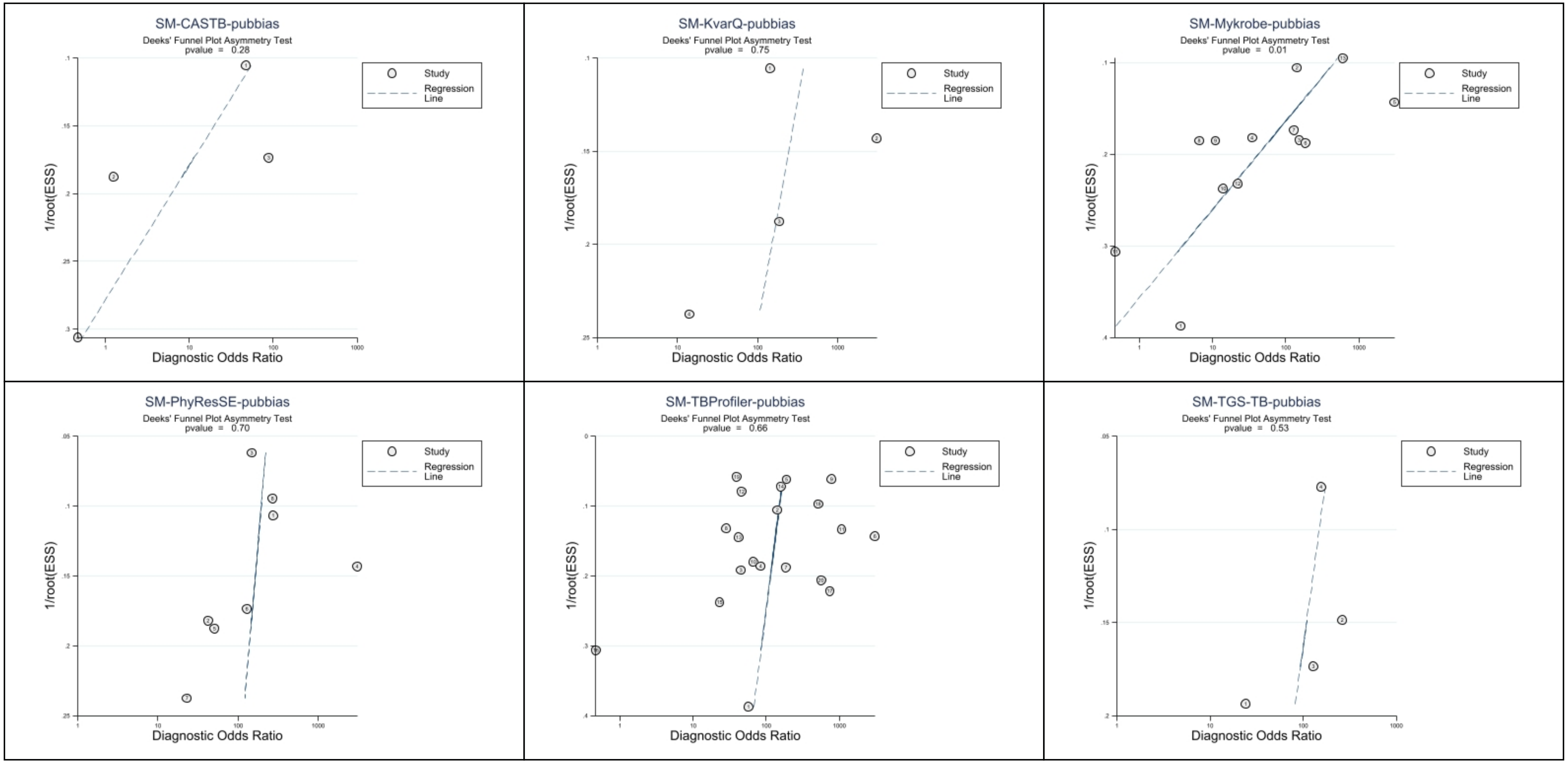

Amikacin

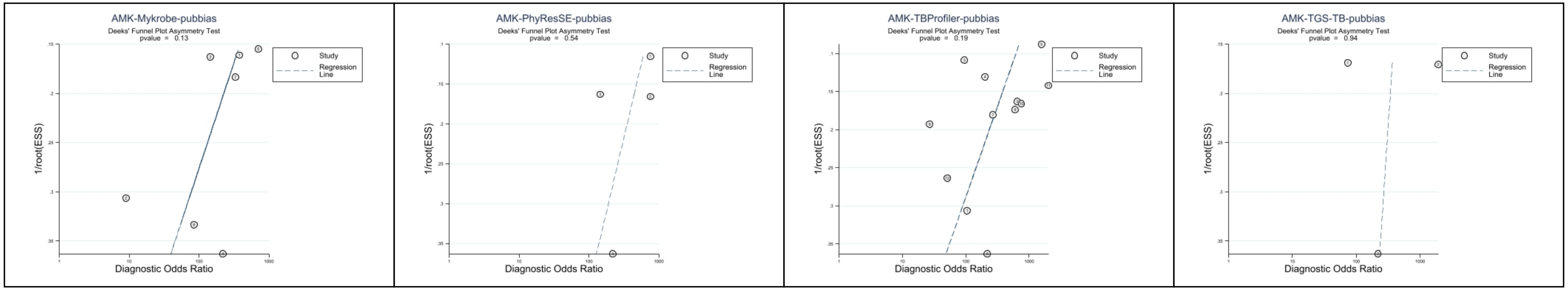

Capreomycin

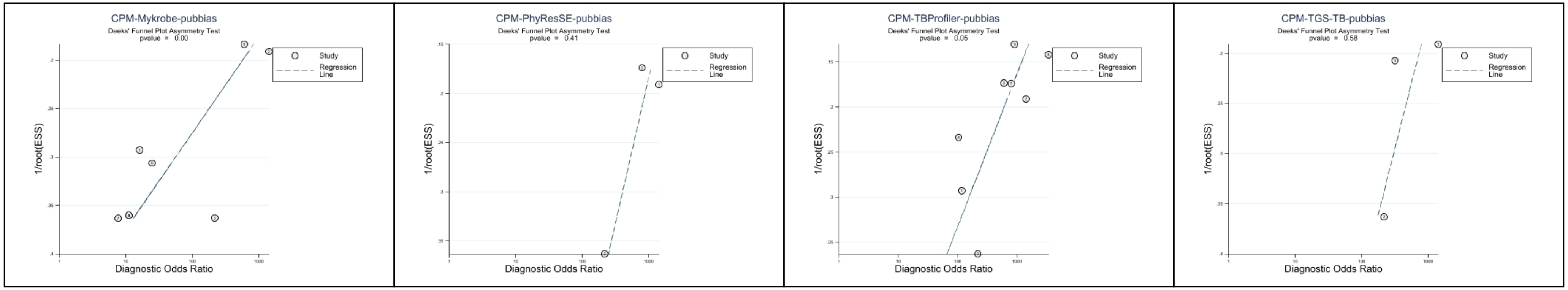

Kanamycin

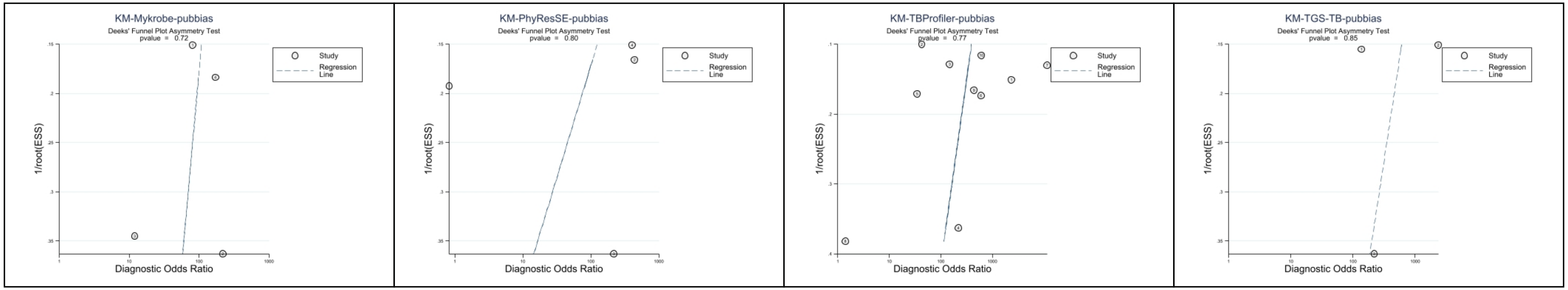

Levofloxacin

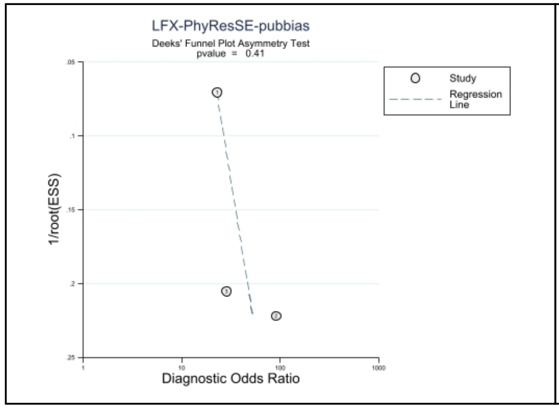

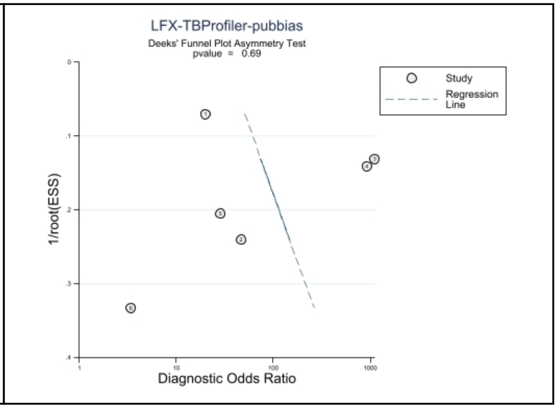

Moxifloxacin

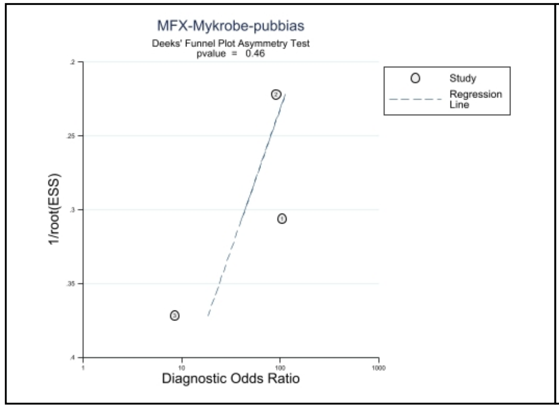

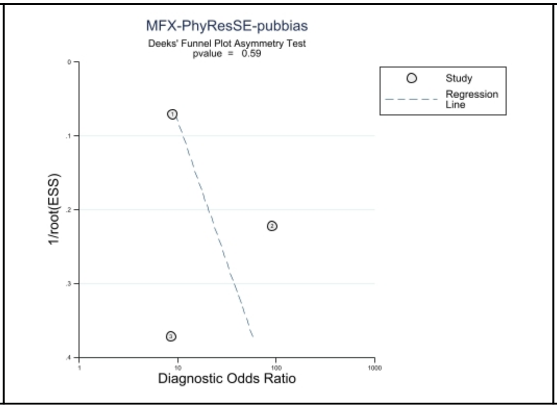

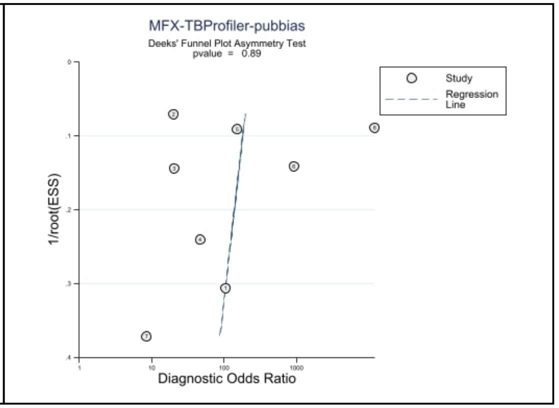

Ofloxacin

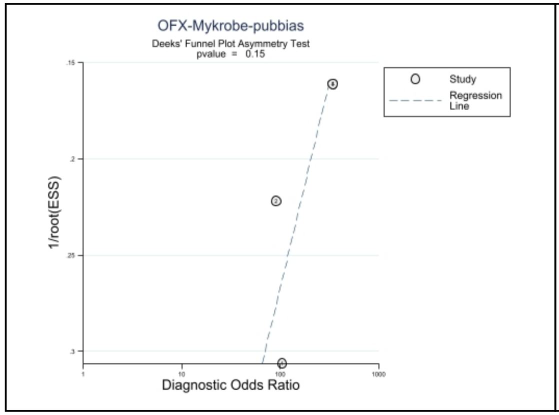

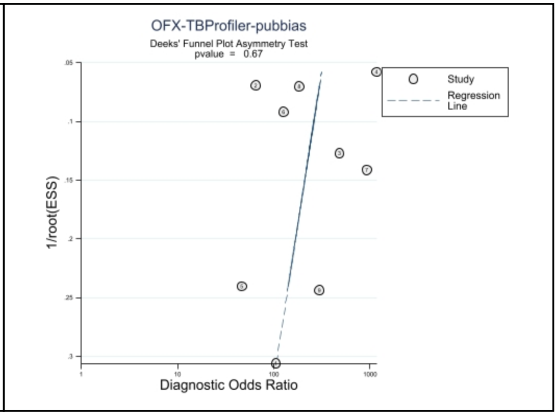

Ethionamide

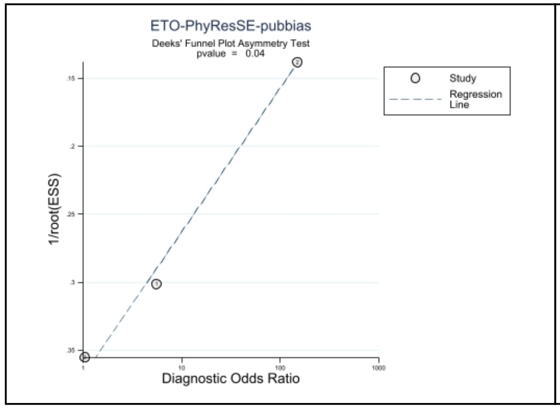

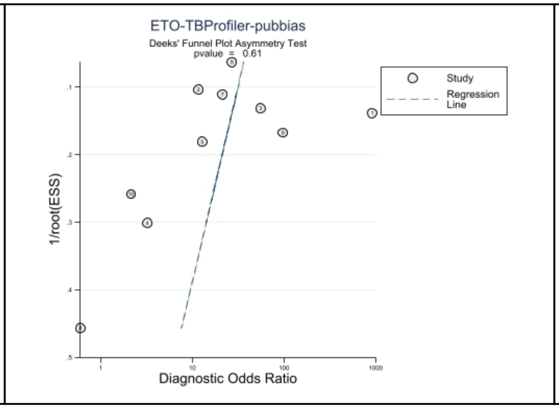

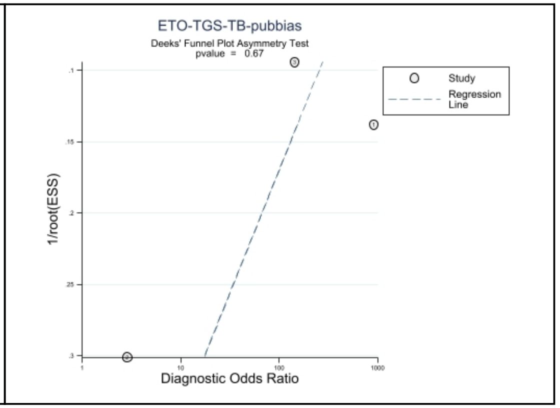

Prothionamide

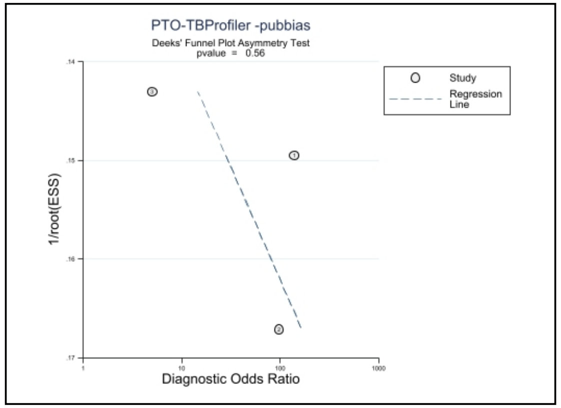

Para-aminosalicylic acid

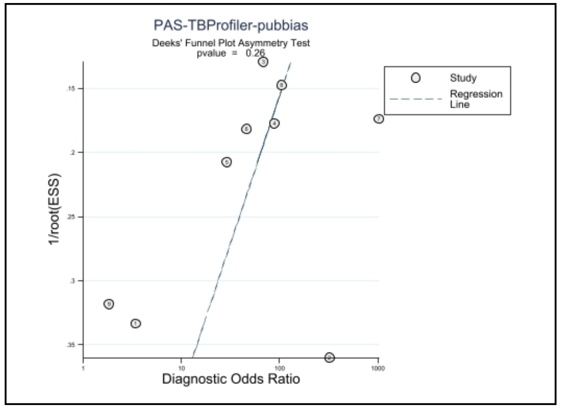

Supplement: S4 Fig — (PDF) [file pgph.0004465.s004.pdf]
