# Supplementary figures and images for: Diagnostic yield of nine user-friendly bioinformatics tools for predicting Mycobacterium tuberculosis drug resistance: A systematic review and network meta-analysis
Source: PLOS Glob Public Health. 2025 Apr 21;5(4):e0004465. doi: 10.1371/journal.pgph.0004465 (PMC12011222; doi:10.1371/journal.pgph.0004465)

**Figure 5. Network visualization of all included bioinformatics tools**

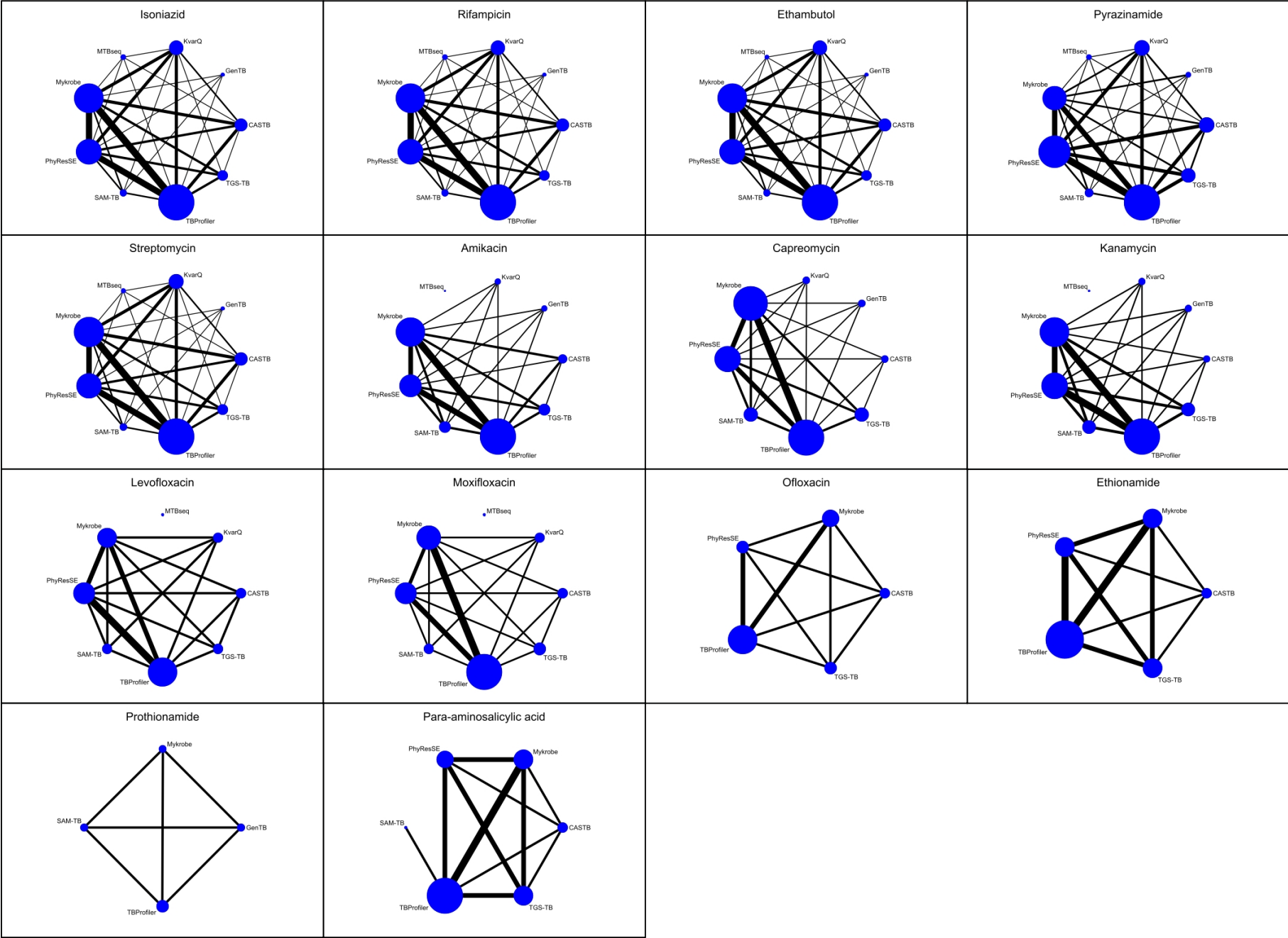

Supplement: S5 Fig — (PDF) [file pgph.0004465.s005.pdf]
