# Supplementary material for: Diagnostic yield of nine user-friendly bioinformatics tools for predicting Mycobacterium tuberculosis drug resistance: A systematic review and network meta-analysis
Source: PLOS Glob Public Health. 2025 Apr 21;5(4):e0004465. doi: 10.1371/journal.pgph.0004465 (PMC12011222; doi:10.1371/journal.pgph.0004465)
Supplement: S2 File — (DOCX) [file pgph.0004465.s007.docx]

File 2. Electronic search strategy

**1. Search Overview:**

- **Search Date:** October 25, 2024

**2. Databases Searched and Results:**

**PubMed:**

- **Search Terms:** CASTB OR KvarQ OR Mykrobe* OR PhyResSE* OR TBProfiler OR "TB Profiler" OR GenTB OR TGSTB OR TGS-TB OR SAMTB OR SAM-TB OR MTBseq OR ResistanceSniffer OR Resistance-Sniffer OR (ARIBA AND resistance)
- **Records Retrieved:** 123

**Embase:**

- **Search Terms:** CASTB OR KvarQ OR Mykrobe* OR PhyResSE* OR TBProfiler OR 'TB Profiler' OR GenTB OR TGSTB OR TGS-TB OR SAMTB OR SAM-TB OR MTBseq OR ResistanceSniffer OR Resistance-Sniffer OR (ARIBA AND resistance)
- **Records Retrieved:** 163

**Scopus:**

- **Search Terms:** CASTB OR KvarQ OR Mykrobe* OR PhyResSE* OR TBProfiler OR "TB Profiler" OR GenTB OR TGSTB OR TGS-TB OR SAMTB OR SAM-TB OR MTBseq OR ResistanceSniffer OR Resistance-Sniffer OR (ARIBA AND AMR)
- **Records Retrieved:** 754

**Web of Science:**

- **Search Terms:** CASTB OR KvarQ OR Mykrobe* OR PhyResSE* OR TBProfiler OR "TB Profiler" OR GenTB OR TGSTB OR TGS-TB OR SAMTB OR SAM-TB OR MTBseq OR ResistanceSniffer OR Resistance-Sniffer OR (ARIBA AND resistance)
- **Records Retrieved:** 146

**CINAHL:**

- **Search Terms:** CASTB OR KvarQ OR Mykrobe* OR PhyResSE* OR TBProfiler OR "TB Profiler" OR GenTB OR TGSTB OR TGS-TB OR SAMTB OR SAM-TB OR MTBseq OR ResistanceSniffer OR Resistance-Sniffer OR (ARIBA AND resistance)
- **Records Retrieved:** 139
