# Supplementary material for: Diagnostic yield of nine user-friendly bioinformatics tools for predicting Mycobacterium tuberculosis drug resistance: A systematic review and network meta-analysis
Source: PLOS Glob Public Health. 2025 Apr 21;5(4):e0004465. doi: 10.1371/journal.pgph.0004465 (PMC12011222; doi:10.1371/journal.pgph.0004465)
Supplement: S3 File — (PDF) [file pgph.0004465.s008.pdf]

File 3. Workflow of the analytical process using streptomycin as an example, with a detailed description of the statistical methods and corresponding results.

Original analysis data

Author, year	Size	MDR/XDR	Method	Drugs	Category	R	S	Tools	TP	FP	FN	TN
Phelan, 2016	10	10	MGIT 960	SM	WHO current	9	1	Mykrobe	5	0	4	1
Phelan, 2016	10	10	MGIT 960	SM	WHO current	9	1	TBProfiler	9	0	0	1
Schleusener, 2017	91	NA	BACTEC 460	SM	WHO past	37	54	CASTB	11	0	26	54
Schleusener, 2017	91	NA	BACTEC 460	SM	WHO past	37	54	KvarQ	21	0	16	54
Schleusener, 2017	91	NA	BACTEC 460	SM	WHO past	37	54	Mykrobe	21	0	16	54
Schleusener, 2017	91	NA	BACTEC 460	SM	WHO past	37	54	PhyResSE	31	1	6	53
Schleusener, 2017	91	NA	BACTEC 460	SM	WHO past	37	54	TBProfiler	21	0	16	54
Chatterjee, 2017	29	12	MGIT 960	SM	WHO past	11	18	Mykrobe	11	2	0	16
Macedo, 2018	54	54	MGIT 960	SM	WHO current	46	8	Mykrobe	31	0	15	8
Macedo, 2018	54	54	MGIT 960	SM	WHO current	46	8	PhyResSE	33	0	13	8
Macedo, 2018	54	54	MGIT 960	SM	WHO current	46	8	TBProfiler	45	4	1	4
Macedo, 2018	54	54	MGIT 960	SM	WHO current	46	8	TGS-TB	31	1	9	7
Feliciano, 2018	29	NA	MGIT 960	SM	WHO current	14	15	TBProfiler	12	1	2	14
Faksri, 2019	266	207	7H10	SM	WHO current	130	132	PhyResSE	116	14	7	125
Faksri, 2019	266	207	7H10	SM	WHO current	130	132	TBProfiler	124	6	13	119
Beek, 2019	211	8	MGIT 960	SM	WHO current	12	199	KvarQ	11	0	1	199
Beek, 2019	211	8	MGIT 960	SM	WHO current	12	199	Mykrobe	11	0	1	199
Beek, 2019	211	8	MGIT 960	SM	WHO current	12	199	PhyResSE	11	0	1	199
Beek, 2019	211	8	MGIT 960	SM	WHO current	12	199	TBProfiler	11	0	1	199
Beek, 2019	211	8	MGIT 960	SM	WHO current	12	199	TGS-TB	11	8	1	191
Guimarães, 2021	71	16	LJ	SM	WHO current	8	63	CASTB	5	36	3	27
Guimarães, 2021	71	16	LJ	SM	WHO current	8	63	KvarQ	6	1	2	62
Guimarães, 2021	71	16	LJ	SM	WHO current	8	63	MTBseq	6	2	2	61
Guimarães, 2021	71	16	LJ	SM	WHO current	8	63	Mykrobe	6	1	2	62
Guimarães, 2021	71	16	LJ	SM	WHO current	8	63	PhyResSE	5	2	3	61
Guimarães, 2021	71	16	LJ	SM	WHO current	8	63	TBProfiler	6	1	2	62
Nonghanphithak, 2020	60	59	7H10	SM	WHO current	36	24	TBProfiler	34	9	2	15
Wu, 2020	306	254	MGIT 960	SM	WHO current	210	96	TBProfiler	204	4	6	92
Kim, 2022	37	14	LJ	SM	WHO past	11	26	CASTB	7	0	4	26
Kim, 2022	37	14	LJ	SM	WHO past	11	26	Mykrobe	8	0	3	26
Kim, 2022	37	14	LJ	SM	WHO past	11	26	PhyResSE	8	0	3	26
Kim, 2022	37	14	LJ	SM	WHO past	11	26	TBProfiler	8	1	3	25
Kim, 2022	37	14	LJ	SM	WHO past	11	26	TGS-TB	8	0	3	26
Che, 2022	59	49	LJ	SM	WHO current	38	21	TBProfiler	37	0	1	21
Wu, 2022	182	59	MycoTB	SM	WHO undefined	59	123	TBProfiler	57	47	2	76
Hall, 2023a	132	NA	LJ or 7H	SM	WHO current	8	83	Mykrobe	4	11	4	72
Hall, 2023b	132	NA	LJ or 7H	SM	WHO current	8	83	Mykrobe	5	11	3	72
Lee, 2023	57	38	LJ	SM	WHO past	17	40	TBProfiler	14	4	3	36
Wang, 2023	202	202	LJ	SM	WHO current	122	80	TBProfiler	98	2	24	78
Xiao, 2023	200	136	7H10	SM	WHO current	60	140	TGS-TB	58	22	2	118
Morey-León, 2023	88	52	MGIT 960 and/or LJ	SM	WHO current	16	5	KvarQ	9	0	7	5
Morey-León, 2023	88	52	MGIT 960 and/or LJ	SM	WHO current	16	5	Mykrobe	9	0	7	5
Morey-León, 2023	88	52	MGIT 960 and/or LJ	SM	WHO current	16	5	PhyResSE	11	0	5	5
Morey-León, 2023	88	52	MGIT 960 and/or LJ	SM	WHO current	16	5	SAM-TB	12	0	4	5
Morey-León, 2023	88	52	MGIT 960 and/or LJ	SM	WHO current	16	5	TBProfiler	11	0	5	5
Daniyarov, 2023	10	10	MGIT 960	SM	WHO current	3	7	CASTB	3	7	0	0
Daniyarov, 2023	10	10	MGIT 960	SM	WHO current	3	7	Mykrobe	3	7	0	0
Daniyarov, 2023	10	10	MGIT 960	SM	WHO current	3	7	TBProfiler	3	7	0	0
Shaw, 2023	38	3	MGIT and solid agar	SM	NA	5	33	TBProfiler	5	0	0	33
Cloutier Charette, 2024	72	32	LJ	SM	NA	5	65	Mykrobe	4	10	1	55
He, 2024	110	100	LJ	SM	WHO current	61	49	GenTB	61	8	0	41
He, 2024	110	100	LJ	SM	WHO current	61	49	Mykrobe	61	8	0	41
He, 2024	110	100	LJ	SM	WHO current	53	57	PhyResSE	53	16	0	41
He, 2024	110	100	LJ	SM	WHO current	65	45	SAM-TB	64	5	1	40
He, 2024	110	100	LJ	SM	WHO current	65	45	TBProfiler	64	5	1	40
Liu, 2024	297	297	7H10	SM	WHO current	137	160	TBProfiler	119	23	18	137
Sadovska, 2024	46	NA	MGIT 960 and/or LJ	SM	WHO current	16	8	TBProfiler	16	0	0	8

Bayesian NMA-ANOVA model

Study	TP	TN	Dis	NDis	Test
1	5	1	9	1	2
1	9	1	9	1	1
2	11	54	37	54	6
2	21	54	37	54	5
2	21	54	37	54	2
2	31	53	37	54	3
2	21	54	37	54	1
3	11	16	11	18	2
5	31	8	46	8	2
5	33	8	46	8	3
5	45	4	46	8	1
5	31	7	46	8	4
6	12	14	14	15	1
7	116	125	130	132	3
7	124	119	130	132	1
8	11	199	12	199	5
8	11	199	12	199	2
8	11	199	12	199	3
8	11	199	12	199	1
8	11	191	12	199	4
10	5	27	8	63	6
10	6	62	8	63	5
10	6	61	8	63	7
10	6	62	8	63	2
10	5	61	8	63	3
10	6	62	8	63	1
11	34	15	36	24	1
12	204	92	210	96	1
14	7	26	11	26	6
14	8	26	11	26	2
14	8	26	11	26	3
14	8	25	11	26	1
14	8	26	11	26	4
15	37	21	38	21	1
16	57	76	59	123	1
18	4	72	8	83	2
18	5	72	8	83	2
19	14	36	17	40	1
20	98	78	122	80	1

TBProfiler	1
Mykrobe	2
PhyResSE	3
TGS-TB	4
KvarQ	5
CASTB	6
MTBseq	7
SAM-TB	8
GenTB	9

21	58	118	60	140	4
22	9	5	16	5	5
22	9	5	16	5	2
22	11	5	16	5	3
22	12	5	16	5	8
22	11	5	16	5	1
25	3	0	3	7	6
25	3	0	3	7	2
25	3	0	3	7	1
27	5	33	5	33	1
28	4	55	5	65	2
29	61	41	61	49	9
29	61	41	61	49	2
29	53	41	53	57	3
29	64	40	65	45	8
29	64	40	65	45	1
30	119	137	137	160	1
33	16	8	16	8	1

```
#========================DATA ===================================
library(rstan)
library(StanHeaders)
library(rstudioapi)
library(ggplot2)
library(plyr)
library(loo)

options(mc.cores = parallel::detectCores())
rstan_options(auto_write = TRUE)
data <- read.csv("data.csv",sep=",", header=T)
data


model = "
data{
  int N;
  int Nt;
  int Ns;
  int TP[N];
  int Dis[N];
  int TN[N];
  int NDis[N];
  int Study[N];
  int Test[N];
}
transformed data{
  vector[2] zero;
  zero[1] <- 0;
  zero[2] <- 0;
}
parameters{
  matrix[2,Nt] logitmu;
  matrix[2,Nt] Multi;
  matrix[Ns,2] nu;
  matrix[Ns,2] delta[Nt];
  vector<lower=0>[2] sigmaw;
  vector<lower=0>[2] sigmab;
  real etarho;
}

transformed parameters{
  matrix[2,Nt] MU;
  matrix[2,Nt] RR;
  matrix[2,Nt] OR;
  vector[Nt] DOR;
```

```
  vector[Nt] S;
  matrix[Nt,Nt] A;
  matrix[Nt,Nt] B;
  matrix[Nt,Nt] C;
  matrix[Ns,2] p_i[Nt];
  vector<lower=0>[2] sigmawsq;
  vector<lower=0>[2] sigmabsq;
  vector<lower=0>[2] sigmasq;
  real rho;
  vector[2] rhow;
  matrix[2, 2] Sigma;
  for (i in 1:Ns){
    for (j in 1:2){
      for (k in 1:Nt)
        p_i[k][i,j] <- inv_logit(logitmu[j,k] +nu[i,j] + delta[k][i,j]);
    }}
  for (j in 1:2){
    for (k in 1:Nt){
      MU[j,k] <- mean(col(p_i[k],j));
    }}
  for (j in 1:2){
    for (k in 1:Nt){
      RR[j,k] <- MU[j,k]/MU[j,Nt];
      OR[j,k] <- (MU[j,k]/(1 - MU[j,k]))/(MU[j,1]/(1 - MU[j,1]));
    }}
  for (l in 1:Nt){
    DOR[l] <- (MU[1,l]*MU[2,l])/((1 -MU[1,l])*(1 - MU[2,l]));
    for(m in 1:Nt){
      A[l,m] <- if_else((MU[1,l] > MU[1,m]) && (MU[2,l] > MU[2,m]),1,0);
      B[l,m] <- if_else((MU[1,l] < MU[1,m]) && (MU[2,l] < MU[2,m]),1,0);
      C[l,m] <- if_else((MU[1,l] == MU[1,m]) && (MU[2,l] == MU[2,m]),1,0);
    }
    S[l] <- (2*sum(row(A,l)) + sum(row(C,l)))/(2*sum(row(B,l)) + sum(row(C,l)));
  }
  rho <- tanh(etarho);
  sigmawsq <- (sigmaw).*(sigmaw);
  sigmabsq <- (sigmab).*(sigmab);
  for (j in 1:2){
    sigmasq[j] <- (sigmabsq[j] + sigmawsq[j]);
    rhow[j] <- sigmabsq[j]/(sigmasq[j]);
  }
  Sigma[1, 1] <- sigmabsq[1];
  Sigma[2, 2] <- sigmabsq[2];
  Sigma[1, 2] <- rho*sigmab[1]*sigmab[2];
  Sigma[2, 1] <- rho*sigmab[1]*sigmab[2];
}
model{
  //Priors
  for (i in 1:Ns){
    nu[i] ~ multi_normal(zero,Sigma);
  }
  for (j in 1:2){
    logitmu[j] ~ normal(0,5);
    Multi[j] ~ normal(0,5);
  }
  sigmaw ~ uniform(0,5);
  sigmab ~ uniform(0,5);
  etarho ~ normal(0,5);
  for (i in 1:Ns){
    for (j in 1:2){
      for (k in 1:Nt)
        delta[k][i,j] ~ normal(0,sigmaw[j]);
    }}
  for (n in 1:N){
  TP[n] ~ binomial(Dis[n] ,p_i[Test[n]][Study[n],1]);
  TN[n] ~ binomial (NDis[n] ,p_i[Test[n]][Study[n],2]);
  }}
generated quantities{
  vector[2*N] loglik;
  for (n in 1:N)
    loglik[n] <- binomial_log(TN[n],NDis[n],p_i[Test[n]][Study[n],1]);
  for (n in (N+1):(2*N))
    loglik[n] <- binomial_log(TN[n-N],NDis[n-N],p_i[Test[n-N]][Study[n-N],2]);
}"

N <- nrow(data)
Ns <- max(data$Study)
Nt <- max(data$Test)
datalist <- list(N = N, Ns = Ns, Nt = Nt, TP = data$TP, Dis = data$Dis, TN = data$TN, NDis = data$NDis, Test =
data$Test, Study = data$Study)
#===========================RESULTS============================
results <- stan(model_code = model, data=datalist, chains = 3, iter = 5000, warmup = 1000, thin = 5)
options(max.print = 10000)
print(results)
```

The results are shown in Table 3, Table 4 and Supplementary Table 6.

Stata-network plot-'networkplot'

study	t1	t2	r1	r2	n1	n2
1	Mykrobe	TBProfiler	5	9	10	10

2	CASTB	KvarQ	11	21	91	91
2	CASTB	Mykrobe	11	21	91	91
2	CASTB	PhyResSE	11	31	91	91
2	CASTB	TBProfiler	11	21	91	91
2	KvarQ	Mykrobe	21	21	91	91
2	KvarQ	PhyResSE	21	31	91	91
2	KvarQ	TBProfiler	21	21	91	91
2	Mykrobe	PhyResSE	21	31	91	91
2	Mykrobe	TBProfiler	21	21	91	91
2	PhyResSE	TBProfiler	31	21	91	91
3	Mykrobe		11		29	
5	Mykrobe	PhyResSE	31	33	54	54
5	Mykrobe	TBProfiler	31	45	54	54
5	Mykrobe	TGS-TB	31	31	54	54
5	PhyResSE	TBProfiler	33	45	54	54
5	PhyResSE	TGS-TB	33	31	54	54
5	TBProfiler	TGS-TB	45	31	54	54
6	TBProfiler		12		29	
7	PhyResSE	TBProfiler	116	124	262	262
8	KvarQ	Mykrobe	11	11	211	211
8	KvarQ	PhyResSE	11	11	211	211
8	KvarQ	TBProfiler	11	11	211	211
8	KvarQ	TGS-TB	11	11	211	211
8	Mykrobe	PhyResSE	11	11	211	211
8	Mykrobe	TBProfiler	11	11	211	211
8	Mykrobe	TGS-TB	11	11	211	211
8	PhyResSE	TBProfiler	11	11	211	211
8	PhyResSE	TGS-TB	11	11	211	211
8	TBProfiler	TGS-TB	11	11	211	211
10	CASTB	KvarQ	5	6	71	71
10	CASTB	MTBseq	5	6	71	71
10	CASTB	Mykrobe	5	6	71	71
10	CASTB	PhyResSE	5	5	71	71
10	CASTB	TBProfiler	5	6	71	71
10	KvarQ	MTBseq	6	6	71	71
10	KvarQ	Mykrobe	6	6	71	71
10	KvarQ	PhyResSE	6	5	71	71
10	KvarQ	TBProfiler	6	6	71	71
10	MTBseq	Mykrobe	6	6	71	71
10	MTBseq	PhyResSE	6	5	71	71
10	MTBseq	TBProfiler	6	6	71	71
10	Mykrobe	PhyResSE	6	5	71	71
10	Mykrobe	TBProfiler	6	6	71	71
10	PhyResSE	TBProfiler	5	6	71	71
11	TBProfiler		34		60	
12	TBProfiler		204		306	
14	CASTB	Mykrobe	7	8	37	37
14	CASTB	PhyResSE	7	8	37	37
14	CASTB	TBProfiler	7	8	37	37
14	CASTB	TGS-TB	7	8	37	37
14	Mykrobe	PhyResSE	8	8	37	37
14	Mykrobe	TBProfiler	8	8	37	37
14	Mykrobe	TGS-TB	8	8	37	37
14	PhyResSE	TBProfiler	8	8	37	37
14	PhyResSE	TGS-TB	8	8	37	37
14	TBProfiler	TGS-TB	8	8	37	37
15	TBProfiler		37		59	
16	TBProfiler		57		182	
18	Mykrobe		4		91	
18	Mykrobe		5		91	
19	TBProfiler		14		57	
20	TBProfiler		98		202	
21	TGS-TB		58		200	
22	KvarQ	Mykrobe	9	9	21	21
22	KvarQ	PhyResSE	9	11	21	21
22	KvarQ	SAM-TB	9	12	21	21
22	KvarQ	TBProfiler	9	11	21	21
22	Mykrobe	PhyResSE	9	11	21	21
22	Mykrobe	SAM-TB	9	12	21	21
22	Mykrobe	TBProfiler	9	11	21	21
22	PhyResSE	SAM-TB	11	12	21	21
22	PhyResSE	TBProfiler	11	11	21	21
22	SAM-TB	TBProfiler	12	11	21	21
25	CASTB	Mykrobe	3	3	10	10
25	CASTB	TBProfiler	3	3	10	10
25	Mykrobe	TBProfiler	3	3	10	10
27	TBProfiler		5		38	
28	Mykrobe		4		70	
29	GenTB	Mykrobe	61	61	110	110
29	GenTB	PhyResSE	61	53	110	110
29	GenTB	SAM-TB	61	64	110	110
29	GenTB	TBProfiler	61	64	110	110
29	Mykrobe	PhyResSE	61	53	110	110
29	Mykrobe	SAM-TB	61	64	110	110
29	Mykrobe	TBProfiler	61	64	110	110
29	PhyResSE	SAM-TB	53	64	110	110
29	PhyResSE	TBProfiler	53	64	110	110
29	SAM-TB	TBProfiler	64	64	110	110
30	TBProfiler		119		297	
33	TBProfiler		16		24	

networkplot t1 t2,edgecol(black)nodecolor(blue)

The results are shown inSupplementary Figure 5.

Stata-DTA-'metadta'+'midas', Revman 5.4

authoryear	Category	Tools	TP	FP	FN	TN	Size	MDR/XDR
Phelan, 2016	WHO_current	Mykrobe	5	0	4	1	10	10
Phelan, 2016	WHO_current	TBProfiler	9	0	0	1	10	10
Schleusener, 2017	WHO_past	CASTB	11	0	26	54	91	NA
Schleusener, 2017	WHO_past	KvarQ	21	0	16	54	91	NA
Schleusener, 2017	WHO_past	Mykrobe	21	0	16	54	91	NA
Schleusener, 2017	WHO_past	PhyResSE	31	1	6	53	91	NA
Schleusener, 2017	WHO_past	TBProfiler	21	0	16	54	91	NA
Chatterjee, 2017	WHO_past	Mykrobe	11	2	0	16	29	12
Macedo, 2018	WHO_current	Mykrobe	31	0	15	8	54	54
Macedo, 2018	WHO_current	PhyResSE	33	0	13	8	54	54

Macedo, 2018	WHO_current	TBProfiler	45	4	1	4	54	54
Macedo, 2018	WHO_current	TGS-TB	31	1	9	7	54	54
Feliciano, 2018	WHO_current	TBProfiler	12	1	2	14	29	NA
Faksri, 2019	WHO_current	PhyResSE	116	14	7	125	266	207
Faksri, 2019	WHO_current	TBProfiler	124	6	13	119	266	207
Beek, 2019	WHO_current	KvarQ	11	0	1	199	211	8
Beek, 2019	WHO_current	Mykrobe	11	0	1	199	211	8
Beek, 2019	WHO_current	PhyResSE	11	0	1	199	211	8
Beek, 2019	WHO_current	TBProfiler	11	0	1	199	211	8
Beek, 2019	WHO_current	TGS-TB	11	8	1	191	211	8
Guimarães, 2021	WHO_current	CASTB	5	36	3	27	71	16
Guimarães, 2021	WHO_current	KvarQ	6	1	2	62	71	16
Guimarães, 2021	WHO_current	MTBseq	6	2	2	61	71	16
Guimarães, 2021	WHO_current	Mykrobe	6	1	2	62	71	16
Guimarães, 2021	WHO_current	PhyResSE	5	2	3	61	71	16
Guimarães, 2021	WHO_current	TBProfiler	6	1	2	62	71	16
Nonghanphithak, 2020	WHO_current	TBProfiler	34	9	2	15	60	59
Wu, 2020	WHO_current	TBProfiler	204	4	6	92	306	254
Kim, 2022	WHO_past	CASTB	7	0	4	26	37	14
Kim, 2022	WHO_past	Mykrobe	8	0	3	26	37	14
Kim, 2022	WHO_past	PhyResSE	8	0	3	26	37	14
Kim, 2022	WHO_past	TBProfiler	8	1	3	25	37	14
Kim, 2022	WHO_past	TGS-TB	8	0	3	26	37	14
Che, 2022	WHO_current	TBProfiler	37	0	1	21	59	49
Wu, 2022	WHO_undefined	TBProfiler	57	47	2	76	182	59
Hall, 2023a	WHO_current	Mykrobe	4	11	4	72	132	NA
Hall, 2023b	WHO_current	Mykrobe	5	11	3	72	132	NA
Lee, 2023	WHO_past	TBProfiler	14	4	3	36	57	38
Wang, 2023	WHO_current	TBProfiler	98	2	24	78	202	202
Xiao, 2023	WHO_current	TGS-TB	58	22	2	118	200	136
Morey-León, 2023	WHO_current	KvarQ	9	0	7	5	88	52
Morey-León, 2023	WHO_current	Mykrobe	9	0	7	5	88	52
Morey-León, 2023	WHO_current	PhyResSE	11	0	5	5	88	52
Morey-León, 2023	WHO_current	SAM-TB	12	0	4	5	88	52
Morey-León, 2023	WHO_current	TBProfiler	11	0	5	5	88	52
Daniyarov, 2023	WHO_current	CASTB	3	7	0	0	10	10
Daniyarov, 2023	WHO_current	Mykrobe	3	7	0	0	10	10
Daniyarov, 2023	WHO_current	TBProfiler	3	7	0	0	10	10
Shaw, 2023	NA	TBProfiler	5	0	0	33	38	3
Cloutier Charette, 2024	NA	Mykrobe	4	10	1	55	72	32
He, 2024	WHO_current	GenTB	61	8	0	41	110	100
He, 2024	WHO_current	Mykrobe	61	8	0	41	110	100
He, 2024	WHO_current	PhyResSE	53	16	0	41	110	100
He, 2024	WHO_current	SAM-TB	64	5	1	40	110	100
He, 2024	WHO_current	TBProfiler	64	5	1	40	110	100
Liu, 2024	WHO_current	TBProfiler	119	23	18	137	297	297
Sadovska, 2024	WHO_current	TBProfiler	16	0	0	8	46	NA

midas tp fp fn tn,plot sroc(both)
midas tp fp fn tn, pubbias

metadta tp fp fn tn, studyid(authoryear) model(random) dp(2) sumtable(all)soptions(xtitle("False positive rate") xlabel(0(0.2)1) xscale(range(0 1)) ytitle("Sensitivity") yscale(range(0 1)) ylabel(0(0.2)1, nogrid) graphregion(color(white)) plotregion(margin(medium)) xsize(15) ysize(15)) foptions(graphregion(color(white)) texts(2) xlabel(0, 0.5, 1) diamopt(color(red)) pointopt(msymbol(s)msize(1)) olineopt(color(red) lpattern(dash)))

Revman v5.4, SROC

The results are shown in Table 1 and 2, S2, Supplementary Figure 2, 3 and 4, Supplementary Table 5.
