# Supplementary material for: Diagnostic yield of nine user-friendly bioinformatics tools for predicting Mycobacterium tuberculosis drug resistance: A systematic review and network meta-analysis
Source: PLOS Glob Public Health. 2025 Apr 21;5(4):e0004465. doi: 10.1371/journal.pgph.0004465 (PMC12011222; doi:10.1371/journal.pgph.0004465)
Supplement: S1 Table — (DOCX) [file pgph.0004465.s010.docx]

| Table 1. Characteristics of included studies | | | | | | | | | | | | | | | | | | | | |
| --- | --- | --- | --- | --- | --- | --- | --- | --- | --- | --- | --- | --- | --- | --- | --- | --- | --- | --- | --- | --- |
| Study characteristics | | | Strains characteristics | | | | Sequencing characteristics | | | Phenotypic DST characteristics | | | | | | Tools characteristics | | | | |
| No. | Author, year | Period | Source | Lineage | Size | MDR/XDR | Platform | Quality | BioProject | Method | Drugs | Critical concentrations | Category | R | S | Tools (version) | TP | FP | FN | TN |
| 1 | Phelan, 2016 | 2007-2013 | Portugal | Lineage 4, 100% | 10 | 10, 100% | Miseq | NA, 51X | PRJEB15555 | MGIT 960 | INH | 0.1 ug/mL | WHO_current | 10 | 0 | Mykrobe (v.0.1.3) | 9 | 0 | 1 | 0 |
|  |  |  |  |  |  |  |  |  |  |  | RMP | 1 ug/mL | WHO_current | 10 | 0 |  | 10 | 0 | 0 | 0 |
|  |  |  |  |  |  |  |  |  |  |  | SM | 1 ug/mL | WHO_current | 9 | 1 |  | 5 | 0 | 4 | 1 |
|  |  |  |  |  |  |  |  |  |  |  | EMB | 5 ug/mL | WHO_current | 9 | 1 |  | 7 | 0 | 2 | 1 |
|  |  |  |  |  |  |  |  |  |  |  | PZA | 100 ug/mL | WHO_current | 9 | 1 |  | 9 | 0 | 0 | 1 |
|  |  |  |  |  |  |  |  |  |  |  | RFB | 0.5 ug/mL | WHO_current | 10 | 0 |  | 10 | 0 | 0 | 0 |
|  |  |  |  |  |  |  |  |  |  |  | ETO | 5 ug/mL | WHO_current | 10 | 0 |  | 8 | 0 | 2 | 0 |
|  |  |  |  |  |  |  |  |  |  |  | AMK | 1 ug/mL | WHO_current | 7 | 3 |  | 4 | 0 | 3 | 3 |
|  |  |  |  |  |  |  |  |  |  |  | CPM | 2.5 ug/mL | WHO_current | 6 | 4 |  | 4 | 0 | 2 | 4 |
|  |  |  |  |  |  |  |  |  |  |  | OFX | 2 ug/mL | WHO_current | 7 | 3 |  | 7 | 0 | 0 | 3 |
|  |  |  |  |  |  |  |  |  |  |  | MFX | 0.25 ug/mL | WHO_current | 7 | 3 |  | 7 | 0 | 0 | 3 |
|  |  |  |  |  |  |  |  |  |  |  | PAS | 4μg/mL | WHO_past | 2 | 8 |  | 2 | 0 | 0 | 8 |
|  |  |  |  |  |  |  |  |  |  |  | LZD | 1 ug/mL | WHO_current | 0 | 10 |  | 0 | 0 | 0 | 10 |
|  |  |  |  |  |  |  |  |  |  |  | INH | 0.1 ug/mL | WHO_current | 10 | 0 | TBProfiler (v.1.0) | 10 | 0 | 0 | 0 |
|  |  |  |  |  |  |  |  |  |  |  | RMP | 1 ug/mL | WHO_current | 10 | 0 |  | 10 | 0 | 0 | 0 |
|  |  |  |  |  |  |  |  |  |  |  | SM | 1 ug/mL | WHO_current | 9 | 1 |  | 9 | 0 | 0 | 1 |
|  |  |  |  |  |  |  |  |  |  |  | EMB | 5 ug/mL | WHO_current | 9 | 1 |  | 9 | 0 | 0 | 1 |
|  |  |  |  |  |  |  |  |  |  |  | PZA | 100 ug/mL | WHO_current | 9 | 1 |  | 7 | 0 | 2 | 1 |
|  |  |  |  |  |  |  |  |  |  |  | RFB | 0.5 ug/mL | WHO_current | 10 | 0 |  | 10 | 0 | 0 | 0 |
|  |  |  |  |  |  |  |  |  |  |  | ETO | 5 ug/mL | WHO_current | 10 | 0 |  | 10 | 0 | 0 | 0 |
|  |  |  |  |  |  |  |  |  |  |  | AMK | 1 ug/mL | WHO_current | 7 | 3 |  | 7 | 0 | 0 | 3 |
|  |  |  |  |  |  |  |  |  |  |  | CPM | 2.5 ug/mL | WHO_current | 6 | 4 |  | 6 | 0 | 0 | 4 |
|  |  |  |  |  |  |  |  |  |  |  | OFX | 2 ug/mL | WHO_current | 7 | 3 |  | 7 | 0 | 0 | 3 |
|  |  |  |  |  |  |  |  |  |  |  | MFX | 0.25 ug/mL | WHO_current | 7 | 3 |  | 7 | 0 | 0 | 3 |
|  |  |  |  |  |  |  |  |  |  |  | PAS | 4μg/mL | WHO_past | 2 | 8 |  | 0 | 0 | 2 | 8 |
|  |  |  |  |  |  |  |  |  |  |  | LZD | 1 ug/mL | WHO_current | 0 | 10 |  | 0 | 0 | 0 | 10 |
| 2 | Schleusener, 2017 | 2003.3-2004.6 | Sierra Leone | Lineage 4, 69.2% | 91 | NA | Miseq | NA | PRJEB7727 | BACTEC 460 | INH | 0.1 ug/mL | WHO_past | 29 | 62 | CASTB (v.1.1); | 24 | 1 | 5 | 61 |
|  |  |  |  |  |  |  |  |  |  |  | RMP | 2 ug/mL | WHO_past | 14 | 77 |  | 14 | 3 | 0 | 74 |
|  |  |  |  |  |  |  |  |  |  |  | SM | 2 ug/mL | WHO_past | 37 | 54 |  | 11 | 0 | 26 | 54 |
|  |  |  |  |  |  |  |  |  |  |  | EMB | 2.5 ug/mL | WHO_past | 14 | 77 |  | 8 | 0 | 6 | 77 |
|  |  |  |  |  |  |  |  |  |  |  | PZA | 100 ug/mL | WHO_past | 9 | 82 |  | 4 | 1 | 5 | 81 |
|  |  |  |  |  |  |  |  |  |  |  | INH | 0.1 ug/mL | WHO_past | 29 | 62 | KvarQ (v.0.12.2); | 23 | 1 | 6 | 61 |
|  |  |  |  |  |  |  |  |  |  |  | RMP | 2 ug/mL | WHO_past | 14 | 77 |  | 13 | 4 | 1 | 73 |
|  |  |  |  |  |  |  |  |  |  |  | SM | 2 ug/mL | WHO_past | 37 | 54 |  | 21 | 0 | 16 | 54 |
|  |  |  |  |  |  |  |  |  |  |  | EMB | 2.5 ug/mL | WHO_past | 14 | 77 |  | 7 | 1 | 7 | 76 |
|  |  |  |  |  |  |  |  |  |  |  | PZA | 100 ug/mL | WHO_past | 9 | 82 |  | 2 | 3 | 7 | 79 |
|  |  |  |  |  |  |  |  |  |  |  | INH | 0.1 ug/mL | WHO_past | 29 | 62 | Mykrobe (v.0.1.3); | 23 | 3 | 6 | 59 |
|  |  |  |  |  |  |  |  |  |  |  | RMP | 2 ug/mL | WHO_past | 14 | 77 |  | 14 | 5 | 0 | 72 |
|  |  |  |  |  |  |  |  |  |  |  | SM | 2 ug/mL | WHO_past | 37 | 54 |  | 21 | 0 | 16 | 54 |
|  |  |  |  |  |  |  |  |  |  |  | EMB | 2.5 ug/mL | WHO_past | 14 | 77 |  | 7 | 1 | 7 | 76 |
|  |  |  |  |  |  |  |  |  |  |  | PZA | 100 ug/mL | WHO_past | 9 | 82 |  | - | - | - | - |
|  |  |  |  |  |  |  |  |  |  |  | INH | 0.1 ug/mL | WHO_past | 29 | 62 | PhyResSE (v.1.0); | 27 | 2 | 2 | 60 |
|  |  |  |  |  |  |  |  |  |  |  | RMP | 2 ug/mL | WHO_past | 14 | 77 |  | 14 | 5 | 0 | 72 |
|  |  |  |  |  |  |  |  |  |  |  | SM | 2 ug/mL | WHO_past | 37 | 54 |  | 31 | 1 | 6 | 53 |
|  |  |  |  |  |  |  |  |  |  |  | EMB | 2.5 ug/mL | WHO_past | 14 | 77 |  | 12 | 2 | 2 | 75 |
|  |  |  |  |  |  |  |  |  |  |  | PZA | 100 ug/mL | WHO_past | 9 | 82 |  | 6 | 3 | 3 | 79 |
|  |  |  |  |  |  |  |  |  |  |  | INH | 0.1 ug/mL | WHO_past | 29 | 62 | TBProfiler | 26 | 10 | 3 | 52 |
|  |  |  |  |  |  |  |  |  |  |  | RMP | 2 ug/mL | WHO_past | 14 | 77 |  | 14 | 5 | 0 | 72 |
|  |  |  |  |  |  |  |  |  |  |  | SM | 2 ug/mL | WHO_past | 37 | 54 |  | 21 | 0 | 16 | 54 |
|  |  |  |  |  |  |  |  |  |  |  | EMB | 2.5 ug/mL | WHO_past | 14 | 77 |  | 12 | 3 | 2 | 74 |
|  |  |  |  |  |  |  |  |  |  |  | PZA | 100 ug/mL | WHO_past | 9 | 82 |  | 4 | 3 | 5 | 79 |
| 3 | Chatterjee, 2017 | 2014.7-2014.9 | Mumbai | Lineage 2, 37%; Lineage 3, 34%; Lineage 4, 23% | 29 | 12, 41.38% | Miseq | NA | PRJNA376471/SRP101835 | MGIT 960 | INH | 0.1 ug/mL | WHO_current | 14 | 15 | Mykrobe (v 0.4.2) | 13 | 0 | 1 | 15 |
|  |  |  |  |  |  |  |  |  |  |  | RMP | 1 ug/mL | WHO_current | 13 | 16 |  | 12 | 0 | 1 | 16 |
|  |  |  |  |  |  |  |  |  |  |  | EMB | 3.5 ug/mL | WHO_past | 11 | 18 |  | 6 | 0 | 5 | 18 |
|  |  |  |  |  |  |  |  |  |  |  | SM | 0.8 ug/mL | WHO_past | 11 | 18 |  | 11 | 2 | 0 | 16 |
| 4 | Bouzouita, 2018 | 2013.1-2015.8 | Tunisia | NA | 82 | 61, 74.4% | Hiseq | NA | U59967 | MGIT 960 | PZA | 100 ug/mL | WHO_current | 25 | 57 | PhyResSE | 23 | 0 | 2 | 57 |
| 5 | Macedo, 2018 | 2013-2017 | Portugal | NA | 54 | 54, 100% | Miseq | 96%, 88.6X | PRJNA431049 | MGIT 960 | SM | 1 ug/mL | WHO_current | 46 | 8 | Mykrobe (v.0.1.3) | 31 | 0 | 15 | 8 |
|  |  |  |  |  |  |  |  |  |  |  | INH | 0.1 ug/mL | WHO_current | 54 | 0 |  | 52 | 0 | 2 | 0 |
|  |  |  |  |  |  |  |  |  |  |  | RMP | 1 ug/mL | WHO_current | 54 | 0 |  | 54 | 0 | 0 | 0 |
|  |  |  |  |  |  |  |  |  |  |  | EMB | 5 ug/mL | WHO_current | 32 | 22 |  | 22 | 5 | 10 | 17 |
|  |  |  |  |  |  |  |  |  |  |  | PZA | 100 ug/mL | WHO_current | 31 | 23 |  | 0 | 0 | 31 | 23 |
|  |  |  |  |  |  |  |  |  |  |  | ETO | 5 ug/mL | WHO_current | 34 | 20 |  | 0 | 0 | 34 | 20 |
|  |  |  |  |  |  |  |  |  |  |  | FLQ | NA | WHO_undefined | 8 | 46 |  | 8 | 0 | 0 | 46 |
|  |  |  |  |  |  |  |  |  |  |  | CPM | 2.5 ug/mL | WHO_current | 7 | 47 |  | 7 | 0 | 0 | 47 |
|  |  |  |  |  |  |  |  |  |  |  | AMK | 1 ug/mL | WHO_current | 11 | 43 |  | 7 | 0 | 4 | 43 |
|  |  |  |  |  |  |  |  |  |  |  | KM | 2.5 ug/mL | WHO_current | 14 | 40 |  | 7 | 0 | 7 | 40 |
|  |  |  |  |  |  |  |  |  |  |  | PAS | 1 ug/mL (LJ) | WHO_past | 1 | 53 |  | 1 | 0 | 0 | 53 |
|  |  |  |  |  |  |  |  |  |  |  | LZD | 1 ug/mL | WHO_current | 0 | 54 |  | 0 | 0 | 0 | 54 |
|  |  |  |  |  |  |  |  |  |  |  | SM | 1 ug/mL | WHO_current | 46 | 8 | PhyResSE (v.1.0) | 33 | 0 | 13 | 8 |
|  |  |  |  |  |  |  |  |  |  |  | INH | 0.1 ug/mL | WHO_current | 54 | 0 |  | 52 | 0 | 2 | 0 |
|  |  |  |  |  |  |  |  |  |  |  | RMP | 1 ug/mL | WHO_current | 54 | 0 |  | 54 | 0 | 0 | 0 |
|  |  |  |  |  |  |  |  |  |  |  | EMB | 5 ug/mL | WHO_current | 32 | 22 |  | 30 | 7 | 2 | 15 |
|  |  |  |  |  |  |  |  |  |  |  | PZA | 100 ug/mL | WHO_current | 31 | 23 |  | 20 | 2 | 11 | 21 |
|  |  |  |  |  |  |  |  |  |  |  | ETO | 5 ug/mL | WHO_current | 34 | 20 |  | 27 | 0 | 7 | 20 |
|  |  |  |  |  |  |  |  |  |  |  | FLQ | NA | WHO_undefined | 8 | 46 |  | 8 | 0 | 0 | 46 |
|  |  |  |  |  |  |  |  |  |  |  | CPM | 2.5 ug/mL | WHO_current | 7 | 47 |  | 7 | 0 | 0 | 47 |
|  |  |  |  |  |  |  |  |  |  |  | AMK | 1 ug/mL | WHO_current | 11 | 43 |  | 7 | 0 | 4 | 43 |
|  |  |  |  |  |  |  |  |  |  |  | KM | 2.5 ug/mL | WHO_current | 14 | 40 |  | 12 | 0 | 2 | 40 |
|  |  |  |  |  |  |  |  |  |  |  | PAS | 1 ug/mL (LJ) | WHO_past | 1 | 53 |  | 1 | 0 | 0 | 53 |
|  |  |  |  |  |  |  |  |  |  |  | LZD | 1 ug/mL | WHO_current | 0 | 54 |  | 0 | 0 | 0 | 54 |
|  |  |  |  |  |  |  |  |  |  |  | SM | 1 ug/mL | WHO_current | 46 | 8 | TBProfiler (v.1.0) | 45 | 4 | 1 | 4 |
|  |  |  |  |  |  |  |  |  |  |  | INH | 0.1 ug/mL | WHO_current | 54 | 0 |  | 54 | 0 | 0 | 0 |
|  |  |  |  |  |  |  |  |  |  |  | RMP | 1 ug/mL | WHO_current | 54 | 0 |  | 53 | 0 | 1 | 0 |
|  |  |  |  |  |  |  |  |  |  |  | EMB | 5 ug/mL | WHO_current | 32 | 22 |  | 28 | 7 | 4 | 15 |
|  |  |  |  |  |  |  |  |  |  |  | PZA | 100 ug/mL | WHO_current | 31 | 23 |  | 22 | 2 | 9 | 21 |
|  |  |  |  |  |  |  |  |  |  |  | ETO | 5 ug/mL | WHO_current | 34 | 20 |  | 33 | 0 | 1 | 20 |
|  |  |  |  |  |  |  |  |  |  |  | FLQ | NA | WHO_undefined | 8 | 46 |  | 8 | 0 | 0 | 46 |
|  |  |  |  |  |  |  |  |  |  |  | CPM | 2.5 ug/mL | WHO_current | 7 | 47 |  | 7 | 0 | 0 | 47 |
|  |  |  |  |  |  |  |  |  |  |  | AMK | 1 ug/mL | WHO_current | 11 | 43 |  | 11 | 1 | 0 | 42 |
|  |  |  |  |  |  |  |  |  |  |  | KM | 2.5 ug/mL | WHO_current | 14 | 40 |  | 14 | 0 | 0 | 40 |
|  |  |  |  |  |  |  |  |  |  |  | PAS | 1 ug/mL (LJ) | WHO_past | 1 | 53 |  | 1 | 0 | 0 | 53 |
|  |  |  |  |  |  |  |  |  |  |  | LZD | 1 ug/mL | WHO_current | 0 | 54 |  | 0 | 0 | 0 | 54 |
|  |  |  |  |  |  |  |  |  |  |  | SM | 1 ug/mL | WHO_current | 46 | 8 | TGS-TB (v.2.0) | 31 | 1 | 9 | 7 |
|  |  |  |  |  |  |  |  |  |  |  | INH | 0.1 ug/mL | WHO_current | 54 | 0 |  | 53 | 0 | 1 | 0 |
|  |  |  |  |  |  |  |  |  |  |  | RMP | 1 ug/mL | WHO_current | 54 | 0 |  | 52 | 0 | 2 | 0 |
|  |  |  |  |  |  |  |  |  |  |  | EMB | 5 ug/mL | WHO_current | 32 | 22 |  | 28 | 6 | 4 | 16 |
|  |  |  |  |  |  |  |  |  |  |  | PZA | 100 ug/mL | WHO_current | 31 | 23 |  | 29 | 2 | 2 | 21 |
|  |  |  |  |  |  |  |  |  |  |  | ETO | 5 ug/mL | WHO_current | 34 | 20 |  | 33 | 0 | 1 | 20 |
|  |  |  |  |  |  |  |  |  |  |  | FLQ | NA | WHO_undefined | 8 | 46 |  | 8 | 0 | 0 | 46 |
|  |  |  |  |  |  |  |  |  |  |  | CPM | 2.5 ug/mL | WHO_current | 7 | 47 |  | 7 | 0 | 0 | 47 |
|  |  |  |  |  |  |  |  |  |  |  | AMK | 1 ug/mL | WHO_current | 11 | 43 |  | 7 | 1 | 4 | 42 |
|  |  |  |  |  |  |  |  |  |  |  | KM | 2.5 ug/mL | WHO_current | 14 | 40 |  | 14 | 0 | 0 | 40 |
|  |  |  |  |  |  |  |  |  |  |  | PAS | 1 ug/mL (LJ) | WHO_past | 1 | 53 |  | 1 | 0 | 0 | 53 |
|  |  |  |  |  |  |  |  |  |  |  | LZD | 1 ug/mL | WHO_current | 0 | 54 |  | 0 | 0 | 0 | 54 |
| 6 | Feliciano, 2018 | NA | Brazil, Mozambique | Lineage 4, 69.2% | 29 | NA | Miseq | NA, 44X | PRJEB23648 | MGIT 960 | INH | 0.1 ug/mL | WHO_current | 23 | 6 | TBProfiler (v.1.0) | 21 | 0 | 2 | 6 |
|  |  |  |  |  |  |  |  |  |  |  | RMP | 1 ug/mL | WHO_current | 16 | 13 |  | 14 | 1 | 2 | 12 |
|  |  |  |  |  |  |  |  |  |  |  | EMB | 5 g/mL | WHO_current | 7 | 22 |  | 7 | 4 | 0 | 18 |
|  |  |  |  |  |  |  |  |  |  |  | SM | 1 ug/mL | WHO_current | 14 | 15 |  | 12 | 1 | 2 | 14 |
| 7 | Faksri, 2019 | 1998-2013 | Thailand | NA | 266 | 207, 77.82% | MiSeq, Hiseq | 97.79 ± 1.65%, 118.88 ± 69.62X | PRJNA390471 | 7H10 | INH | 0.2 ug/mL | WHO_current | 204 | 57 | PhyResSE | 194 | 10 | 0 | 57 |
|  |  |  |  |  |  |  |  |  |  |  | RMP | 1 ug/mL | WHO_current | 202 | 60 |  | 183 | 19 | 0 | 60 |
|  |  |  |  |  |  |  |  |  |  |  | SM | 2 ug/mL | WHO_current | 130 | 132 |  | 116 | 14 | 7 | 125 |
|  |  |  |  |  |  |  |  |  |  |  | EMB | 5 ug/mL | WHO_current | 121 | 108 |  | 106 | 15 | 20 | 88 |
|  |  |  |  |  |  |  |  |  |  |  | AMK | 6 ug/mL | WHO_undefined | 29 | 175 |  | 20 | 9 | 0 | 175 |
|  |  |  |  |  |  |  |  |  |  |  | KM | 6 ug/mL | WHO_past | 34 | 170 |  | 1 | 33 | 6 | 164 |
|  |  |  |  |  |  |  |  |  |  |  | ETO | 5 ug/mL | WHO_current | 49 | 155 |  | 0 | 49 | 1 | 154 |
|  |  |  |  |  |  |  |  |  |  |  | OFX | 2 ug/mL | WHO_current | 111 | 96 |  | 99 | 12 | 5 | 91 |
|  |  |  |  |  |  |  |  |  |  |  | GFX | 2 ug/mL | WHO_past | 20 | 184 |  | 19 | 1 | 86 | 98 |
|  |  |  |  |  |  |  |  |  |  |  | LFX | 2 ug/mL | WHO_current | 66 | 135 |  | 60 | 6 | 41 | 94 |
|  |  |  |  |  |  |  |  |  |  |  | MFX | 2 ug/mL | WHO_past | 60 | 141 |  | 50 | 10 | 51 | 90 |
|  |  |  |  |  |  |  |  |  |  |  | INH | 0.2 ug/mL | WHO_current | 204 | 57 | TBProfiler | 197 | 7 | 8 | 49 |
|  |  |  |  |  |  |  |  |  |  |  | RMP | 1 ug/mL | WHO_current | 202 | 60 |  | 185 | 17 | 0 | 60 |
|  |  |  |  |  |  |  |  |  |  |  | SM | 2 ug/mL | WHO_current | 130 | 132 |  | 124 | 6 | 13 | 119 |
|  |  |  |  |  |  |  |  |  |  |  | EMB | 5 ug/mL | WHO_current | 121 | 108 |  | 112 | 9 | 20 | 88 |
|  |  |  |  |  |  |  |  |  |  |  | AMK | 6 ug/mL | WHO_undefined | 29 | 175 |  | 20 | 9 | 4 | 171 |
|  |  |  |  |  |  |  |  |  |  |  | KM | 6 ug/mL | WHO_past | 34 | 170 |  | 22 | 12 | 7 | 163 |
|  |  |  |  |  |  |  |  |  |  |  | ETO | 5 ug/mL | WHO_current | 49 | 155 |  | 19 | 30 | 8 | 147 |
|  |  |  |  |  |  |  |  |  |  |  | OFX | 2 ug/mL | WHO_current | 111 | 96 |  | 98 | 13 | 10 | 86 |
|  |  |  |  |  |  |  |  |  |  |  | GFX | 2 ug/mL | WHO_past | 20 | 184 |  | 19 | 1 | 89 | 95 |
|  |  |  |  |  |  |  |  |  |  |  | LFX | 2 ug/mL | WHO_current | 66 | 135 |  | 60 | 6 | 45 | 90 |
|  |  |  |  |  |  |  |  |  |  |  | MFX | 2 ug/mL | WHO_past | 60 | 141 |  | 55 | 5 | 50 | 91 |
| 8 | Beek, 2019 | 2014.1-2014.12 | Finland | NA | 211 | 8, 3.8% | Miseq | NA, 71X | PRJEB25543 | MGIT 960 | INH | 0.1 ug/mL | WHO_current | 16 | 195 | KvarQ | 12 | 0 | 4 | 195 |
|  |  |  |  |  |  |  |  |  |  |  | RMP | 1 ug/mL | WHO_current | 8 | 203 |  | 8 | 0 | 0 | 203 |
|  |  |  |  |  |  |  |  |  |  |  | EMB | 5 ug/mL | WHO_current | 1 | 210 |  | 0 | 3 | 1 | 206 |
|  |  |  |  |  |  |  |  |  |  |  | PZA | 100 ug/mL | WHO_current | 5 | 206 |  | 3 | 2 | 1 | 204 |
|  |  |  |  |  |  |  |  |  |  |  | SM | 1 ug/mL | WHO_current | 12 | 199 |  | 11 | 0 | 1 | 199 |
|  |  |  |  |  |  |  |  |  |  |  | INH | 0.1 ug/mL | WHO_current | 16 | 195 | Mykrobe (v.0.1.3) | 12 | 0 | 4 | 195 |
|  |  |  |  |  |  |  |  |  |  |  | RMP | 1 ug/mL | WHO_current | 8 | 203 |  | 8 | 4 | 0 | 199 |
|  |  |  |  |  |  |  |  |  |  |  | EMB | 5 ug/mL | WHO_current | 1 | 210 |  | 0 | 3 | 1 | 207 |
|  |  |  |  |  |  |  |  |  |  |  | PZA | 100 ug/mL | WHO_current | 5 | 206 |  | - | - | - | - |
|  |  |  |  |  |  |  |  |  |  |  | SM | 1 ug/mL | WHO_current | 12 | 199 |  | 11 | 0 | 1 | 199 |
|  |  |  |  |  |  |  |  |  |  |  | INH | 0.1 ug/mL | WHO_current | 16 | 195 | PhyResSE (v.1.0) | 13 | 0 | 3 | 195 |
|  |  |  |  |  |  |  |  |  |  |  | RMP | 1 ug/mL | WHO_current | 8 | 203 |  | 8 | 0 | 0 | 203 |
|  |  |  |  |  |  |  |  |  |  |  | EMB | 5 ug/mL | WHO_current | 1 | 210 |  | 0 | 5 | 1 | 205 |
|  |  |  |  |  |  |  |  |  |  |  | PZA | 100 ug/mL | WHO_current | 5 | 206 |  | 2 | 1 | 3 | 205 |
|  |  |  |  |  |  |  |  |  |  |  | SM | 1 ug/mL | WHO_current | 12 | 199 |  | 11 | 0 | 1 | 199 |
|  |  |  |  |  |  |  |  |  |  |  | INH | 0.1 ug/mL | WHO_current | 16 | 195 | TBProfiler (v.0.1) | 13 | 7 | 3 | 188 |
|  |  |  |  |  |  |  |  |  |  |  | RMP | 1 ug/mL | WHO_current | 8 | 203 |  | 8 | 0 | 0 | 203 |
|  |  |  |  |  |  |  |  |  |  |  | EMB | 5 ug/mL | WHO_current | 1 | 210 |  | 1 | 7 | 0 | 202 |
|  |  |  |  |  |  |  |  |  |  |  | PZA | 100 ug/mL | WHO_current | 5 | 206 |  | 3 | 0 | 1 | 206 |
|  |  |  |  |  |  |  |  |  |  |  | SM | 1 ug/mL | WHO_current | 12 | 199 |  | 11 | 0 | 1 | 199 |
|  |  |  |  |  |  |  |  |  |  |  | INH | 0.1 ug/mL | WHO_current | 16 | 195 | TGS-TB (v.2) | 12 | 30 | 4 | 165 |
|  |  |  |  |  |  |  |  |  |  |  | RMP | 1 ug/mL | WHO_current | 8 | 203 |  | 8 | 0 | 0 | 203 |
|  |  |  |  |  |  |  |  |  |  |  | EMB | 5 ug/mL | WHO_current | 1 | 210 |  | 1 | 6 | 0 | 203 |
|  |  |  |  |  |  |  |  |  |  |  | PZA | 100 ug/mL | WHO_current | 5 | 206 |  | 3 | 0 | 1 | 206 |
|  |  |  |  |  |  |  |  |  |  |  | SM | 1 ug/mL | WHO_current | 12 | 199 |  | 11 | 8 | 1 | 191 |
| 9 | Iwamoto, 2019 | 2001-2015 | Japan | NA | 191 | 165, 86.39% | Miseq | NA | PRJDB7006/DRA006844 | MGIT 960 | PZA | 100 ug/mL | WHO_current | 108 | 83 | CASTB (v.1.5) | 34 | 1 | 74 | 82 |
|  |  |  |  |  |  |  |  |  |  |  | PZA | 100 ug/mL | WHO_current | 108 | 83 | PhyResSE (v.1.0) | 53 | 1 | 55 | 82 |
|  |  |  |  |  |  |  |  |  |  |  | PZA | 100 ug/mL | WHO_current | 108 | 83 | TBProfiler (v.0.2.1) | 65 | 0 | 43 | 83 |
|  |  |  |  |  |  |  |  |  |  |  | PZA | 100 ug/mL | WHO_current | 108 | 83 | TGS-TB (v.2.0) | 105 | 1 | 3 | 82 |
| 10 | Guimarães, 2021 | NA | Brazil | Lineage 1, 100% | 71 | 16, 22.53% | Hiseq | 250X | PRJNA49493, PRJNA630228 | LJ | INH | 0.2 ug/mL | WHO_current | 30 | 41 | CASTB | 25 | 3 | 5 | 38 |
|  |  |  |  |  |  |  |  |  |  |  | RMP | 40 ug/mL | WHO_current | 18 | 53 |  | 13 | 4 | 5 | 49 |
|  |  |  |  |  |  |  |  |  |  |  | PZA | NA | WHO_past | 4 | 65 |  | 2 | 4 | 2 | 61 |
|  |  |  |  |  |  |  |  |  |  |  | EMB | 2 ug/mL | WHO_current | 8 | 63 |  | 5 | 5 | 3 | 58 |
|  |  |  |  |  |  |  |  |  |  |  | SM | 4 ug/mL | WHO_current | 8 | 63 |  | 5 | 36 | 3 | 27 |
|  |  |  |  |  |  |  |  |  |  |  | INH | 0.2 ug/mL | WHO_current | 30 | 41 | KvarQ (v.0.12.2) | 26 | 1 | 4 | 40 |
|  |  |  |  |  |  |  |  |  |  |  | RMP | 40 ug/mL | WHO_current | 18 | 53 |  | 17 | 5 | 1 | 48 |
|  |  |  |  |  |  |  |  |  |  |  | PZA | NA | WHO_past | 4 | 65 |  | 1 | 4 | 5 | 61 |
|  |  |  |  |  |  |  |  |  |  |  | EMB | 2 ug/mL | WHO_current | 8 | 63 |  | 7 | 2 | 1 | 61 |
|  |  |  |  |  |  |  |  |  |  |  | SM | 4 ug/mL | WHO_current | 8 | 63 |  | 6 | 1 | 2 | 62 |
|  |  |  |  |  |  |  |  |  |  |  | INH | 0.2 ug/mL | WHO_current | 30 | 41 | Mykrobe (v.0.8.1) | 27 | 2 | 3 | 39 |
|  |  |  |  |  |  |  |  |  |  |  | RMP | 40 ug/mL | WHO_current | 18 | 53 |  | 17 | 6 | 1 | 47 |
|  |  |  |  |  |  |  |  |  |  |  | PZA | NA | WHO_past | 4 | 65 |  | 4 | 5 | 2 | 60 |
|  |  |  |  |  |  |  |  |  |  |  | EMB | 2 ug/mL | WHO_current | 8 | 63 |  | 7 | 7 | 1 | 56 |
|  |  |  |  |  |  |  |  |  |  |  | SM | 4 ug/mL | WHO_current | 8 | 63 |  | 6 | 1 | 2 | 62 |
|  |  |  |  |  |  |  |  |  |  |  | INH | 0.2 ug/mL | WHO_current | 30 | 41 | PhyResSE | 20 | 0 | 10 | 41 |
|  |  |  |  |  |  |  |  |  |  |  | RMP | 40 ug/mL | WHO_current | 18 | 53 |  | 1 | 0 | 17 | 53 |
|  |  |  |  |  |  |  |  |  |  |  | PZA | NA | WHO_past | 4 | 65 |  | 2 | 5 | 4 | 60 |
|  |  |  |  |  |  |  |  |  |  |  | EMB | 2 ug/mL | WHO_current | 8 | 63 |  | 8 | 4 | 0 | 59 |
|  |  |  |  |  |  |  |  |  |  |  | SM | 4 ug/mL | WHO_current | 8 | 63 |  | 5 | 2 | 3 | 61 |
|  |  |  |  |  |  |  |  |  |  |  | INH | 0.2 ug/mL | WHO_current | 30 | 41 | TBProfiler (v.2.8.6) | 26 | 1 | 4 | 40 |
|  |  |  |  |  |  |  |  |  |  |  | RMP | 40 ug/mL | WHO_current | 18 | 53 |  | 17 | 5 | 1 | 48 |
|  |  |  |  |  |  |  |  |  |  |  | PZA | NA | WHO_past | 4 | 65 |  | 3 | 2 | 3 | 63 |
|  |  |  |  |  |  |  |  |  |  |  | EMB | 2 ug/mL | WHO_current | 8 | 63 |  | 8 | 6 | 0 | 57 |
|  |  |  |  |  |  |  |  |  |  |  | SM | 4 ug/mL | WHO_current | 8 | 63 |  | 6 | 1 | 2 | 62 |
|  |  |  |  |  |  |  |  |  |  |  | INH | 0.2 ug/mL | WHO_current | 30 | 41 | MTBseq (v.1.0.4) | 28 | 10 | 2 | 31 |
|  |  |  |  |  |  |  |  |  |  |  | RMP | 40 ug/mL | WHO_current | 18 | 53 |  | 15 | 5 | 3 | 48 |
|  |  |  |  |  |  |  |  |  |  |  | PZA | NA | WHO_past | 4 | 65 |  | 2 | 5 | 4 | 60 |
|  |  |  |  |  |  |  |  |  |  |  | EMB | 2 ug/mL | WHO_current | 8 | 63 |  | 7 | 5 | 1 | 58 |
|  |  |  |  |  |  |  |  |  |  |  | SM | 4 ug/mL | WHO_current | 8 | 63 |  | 6 | 2 | 2 | 61 |
| 11.1 | Nonghanphithak, 2020 | 2003-2017 | Thailand | Lineage 2, 88.3% | 60 | 59, 98.3% | Miseq | 97.9%, 224.5 ± 152.4X | PRJNA598981, PRJNA598949 | 7H10 | INH | 0.2 ug/mL | WHO_current | 59 | 1 | TBProfiler (v.2.8.6) | 56 | 0 | 3 | 1 |
|  |  |  |  |  |  |  |  |  |  |  | RMP | 1 ug/mL | WHO_current | 60 | 0 |  | 57 | 0 | 3 | 0 |
|  |  |  |  |  |  |  |  |  |  |  | EMB | 5 ug/mL | WHO_current | 38 | 19 |  | 35 | 4 | 3 | 15 |
|  |  |  |  |  |  |  |  |  |  |  | SM | 2 ug/mL | WHO_current | 36 | 24 |  | 34 | 9 | 2 | 15 |
|  |  |  |  |  |  |  |  |  |  |  | KM | 6 ug/mL | WHO_past | 27 | 32 |  | 19 | 0 | 8 | 32 |
|  |  |  |  |  |  |  |  |  |  |  | AMK | 6 ug/mL | WHO_undefined | 23 | 37 |  | 17 | 0 | 6 | 37 |
|  |  |  |  |  |  |  |  |  |  |  | OFX | 2 ug/mL | WHO_current | 31 | 29 |  | 28 | 0 | 3 | 29 |
|  |  |  |  |  |  |  |  |  |  |  | MFX | 2 ug/mL | WHO_past | 17 | 41 |  | 15 | 11 | 2 | 30 |
|  |  |  |  |  |  |  |  |  |  |  | ETO | 5 ug/mL | WHO_current | 25 | 35 |  | 23 | 6 | 2 | 29 |
|  |  |  |  |  |  |  |  |  |  |  | PAS | 2 ug/mL | WHO_past | 31 | 29 |  | 22 | 1 | 9 | 28 |
| 11.2 |  |  |  |  |  |  |  |  |  | MycoTB | INH | 0.25 ug/mL | WHO_undefined | 56 | 4 |  | 54 | 2 | 2 | 2 |
|  |  |  |  |  |  |  |  |  |  |  | RMP | 1 ug/mL | WHO_undefined | 51 | 9 |  | 51 | 6 | 0 | 3 |
|  |  |  |  |  |  |  |  |  |  |  | EMB | 4 ug/mL | WHO_undefined | 21 | 39 |  | 21 | 21 | 0 | 18 |
|  |  |  |  |  |  |  |  |  |  |  | SM | 2 ug/mL | WHO_undefined | 40 | 20 |  | 39 | 4 | 1 | 16 |
|  |  |  |  |  |  |  |  |  |  |  | KM | 5 ug/mL | WHO_undefined | 20 | 40 |  | 19 | 0 | 1 | 40 |
|  |  |  |  |  |  |  |  |  |  |  | AMK | 4 ug/mL | WHO_undefined | 18 | 42 |  | 17 | 0 | 1 | 42 |
|  |  |  |  |  |  |  |  |  |  |  | OFX | 2 ug/mL | WHO_undefined | 29 | 31 |  | 28 | 0 | 1 | 31 |
|  |  |  |  |  |  |  |  |  |  |  | MFX | 1 ug/mL | WHO_undefined | 25 | 35 |  | 24 | 4 | 1 | 31 |
|  |  |  |  |  |  |  |  |  |  |  | ETO | 5 ug/mL | WHO_undefined | 6 | 54 |  | 6 | 23 | 0 | 31 |
|  |  |  |  |  |  |  |  |  |  |  | PAS | 1 ug/mL | WHO_undefined | 25 | 35 |  | 20 | 3 | 5 | 32 |
| 12 | Wu, 2020 | 2013.1.1-2019.6.30 | China | Lineage 2, 95% | 306 | 254, 83% | Hiseq | 99.38%, 166.29X | NA | MGIT 960 | INH | 0.1 ug/mL | WHO_current | 256 | 50 | TBProfiler (v.2.8.5) | 242 | 4 | 14 | 46 |
|  |  |  |  |  |  |  |  |  |  |  | RMP | 1 ug/mL | WHO_current | 276 | 30 |  | 268 | 0 | 8 | 30 |
|  |  |  |  |  |  |  |  |  |  |  | EMB | 5 ug/mL | WHO_current | 118 | 188 |  | 115 | 67 | 3 | 121 |
|  |  |  |  |  |  |  |  |  |  |  | SM | 1 ug/mL | WHO_current | 210 | 96 |  | 204 | 4 | 6 | 92 |
|  |  |  |  |  |  |  |  |  |  |  | OFX | 2 ug/mL | WHO_current | 129 | 177 |  | 120 | 2 | 9 | 175 |
|  |  |  |  |  |  |  |  |  |  |  | AMK | 1 ug/mL | WHO_current | 36 | 270 |  | 27 | 0 | 9 | 270 |
| 13 | Genestet, 2020 | 2016.11-2019.7 | France | NA | 274 | 4, 1.46% | NextSeq or MiSeq | NA | NA | MGIT 960 | RMP | 1 ug/mL | WHO_current | 6 | 268 | PhyResSE (v.1.0) | 6 | 1 | 0 | 267 |
|  |  |  |  |  |  |  |  |  |  |  | INH | 0.1 ug/mL | WHO_current | 21 | 253 |  | 21 | 0 | 0 | 253 |
|  |  |  |  |  |  |  |  |  |  |  | EMB | 5 ug/mL | WHO_current | 1 | 273 |  | 1 | 4 | 0 | 269 |
|  |  |  |  |  |  |  |  |  |  |  | PZA | 100 ug/mL | WHO_current | 16 | 258 |  | 15 | 0 | 1 | 258 |
| 14 | Kim, 2022 | 2010-2018 | Korea | Lineage 2, 81.1% | 37 | 14, 37.84% | Miseq | NA, 112.06 ± 33.13X | NA | LJ | INH | 0.2 µg/mL | WHO_current | 17 | 20 | CASTB | 11 | 13 | 6 | 7 |
|  |  |  |  |  |  |  |  |  |  |  | RMP | 40 ug/mL | WHO_current | 10 | 27 |  | 9 | 5 | 1 | 22 |
|  |  |  |  |  |  |  |  |  |  |  | EMB | 2 ug/mL | WHO_current | 4 | 33 |  | 3 | 5 | 1 | 28 |
|  |  |  |  |  |  |  |  |  |  |  | FLQ | LFX: 2 ug/mL MFX: 1 ug/mL OFX: 4 ug/mL | WHO_past | 5 | 32 |  | 2 | 0 | 3 | 32 |
|  |  |  |  |  |  |  |  |  |  |  | CS | 30 ug/mL | WHO_past | 0 | 37 |  | 0 | 0 | 0 | 37 |
|  |  |  |  |  |  |  |  |  |  |  | SM | 10 ug/mL | WHO_past | 11 | 26 |  | 7 | 0 | 4 | 26 |
|  |  |  |  |  |  |  |  |  |  |  | AMG | AMK: 30 ug/mL KM: 30 ug/mL CPM: 40 ug/mL | WHO_current | 1 | 36 |  | 1 | 0 | 0 | 36 |
|  |  |  |  |  |  |  |  |  |  |  | ETO | 40 ug/mL | WHO_current | 3 | 34 |  | 0 | 0 | 3 | 34 |
|  |  |  |  |  |  |  |  |  |  |  | PAS | 1 ug/mL | WHO_past | 0 | 37 |  | 0 | 0 | 0 | 37 |
|  |  |  |  |  |  |  |  |  |  | Pyrazinamidase test | PZA | NA | WHO_past | 0 | 37 |  | 0 | 4 | 0 | 33 |
|  |  |  |  |  |  |  |  |  |  | LJ | INH | 0.2 µg/mL | WHO_current | 17 | 20 | Mykrobe | 11 | 15 | 6 | 5 |
|  |  |  |  |  |  |  |  |  |  |  | RMP | 40 ug/mL | WHO_current | 10 | 27 |  | 8 | 2 | 2 | 25 |
|  |  |  |  |  |  |  |  |  |  |  | EMB | 2 ug/mL | WHO_current | 4 | 33 |  | 3 | 4 | 1 | 29 |
|  |  |  |  |  |  |  |  |  |  |  | FLQ | LFX: 2 ug/mL MFX: 1 ug/mL OFX: 4 ug/mL | WHO_past | 5 | 32 |  | 3 | 0 | 2 | 32 |
|  |  |  |  |  |  |  |  |  |  |  | CS | 30 ug/mL | WHO_past | 0 | 37 |  | 0 | 0 | 0 | 37 |
|  |  |  |  |  |  |  |  |  |  |  | SM | 10 ug/mL | WHO_past | 11 | 26 |  | 8 | 0 | 3 | 26 |
|  |  |  |  |  |  |  |  |  |  |  | AMG | AMK: 30 ug/mL KM: 30 ug/mL CPM: 40 ug/mL | WHO_current | 1 | 36 |  | 1 | 0 | 0 | 36 |
|  |  |  |  |  |  |  |  |  |  |  | ETO | 40 ug/mL | WHO_current | 3 | 34 |  | 0 | 0 | 3 | 34 |
|  |  |  |  |  |  |  |  |  |  |  | PAS | 1 ug/mL | WHO_past | 0 | 37 |  | 0 | 0 | 0 | 37 |
|  |  |  |  |  |  |  |  |  |  | Pyrazinamidase test | PZA | NA | WHO_past | 0 | 37 |  | 0 | 5 | 0 | 32 |
|  |  |  |  |  |  |  |  |  |  | LJ | INH | 0.2 µg/mL | WHO_current | 17 | 20 | PhyResSE | 11 | 15 | 6 | 5 |
|  |  |  |  |  |  |  |  |  |  |  | RMP | 40 ug/mL | WHO_current | 10 | 27 |  | 8 | 5 | 2 | 22 |
|  |  |  |  |  |  |  |  |  |  |  | EMB | 2 ug/mL | WHO_current | 4 | 33 |  | 3 | 4 | 1 | 29 |
|  |  |  |  |  |  |  |  |  |  |  | FLQ | LFX: 2 ug/mL MFX: 1 ug/mL OFX: 4 ug/mL | WHO_past | 5 | 32 |  | 3 | 0 | 2 | 32 |
|  |  |  |  |  |  |  |  |  |  |  | CS | 30 ug/mL | WHO_past | 0 | 37 |  | 0 | 0 | 0 | 37 |
|  |  |  |  |  |  |  |  |  |  |  | SM | 10 ug/mL | WHO_past | 11 | 26 |  | 8 | 0 | 3 | 26 |
|  |  |  |  |  |  |  |  |  |  |  | AMG | AMK: 30 ug/mL KM: 30 ug/mL CPM: 40 ug/mL | WHO_current | 1 | 36 |  | 1 | 0 | 0 | 36 |
|  |  |  |  |  |  |  |  |  |  |  | ETO | 40 ug/mL | WHO_current | 3 | 34 |  | 2 | 9 | 1 | 25 |
|  |  |  |  |  |  |  |  |  |  |  | PAS | 1 ug/mL | WHO_past | 0 | 37 |  | 0 | 0 | 0 | 37 |
|  |  |  |  |  |  |  |  |  |  | Pyrazinamidase test | PZA | NA | WHO_past | 0 | 37 |  | 0 | 11 | 0 | 26 |
|  |  |  |  |  |  |  |  |  |  | LJ | INH | 0.2 µg/mL | WHO_current | 17 | 20 | TBProfiler | 12 | 15 | 5 | 5 |
|  |  |  |  |  |  |  |  |  |  |  | RMP | 40 ug/mL | WHO_current | 10 | 27 |  | 8 | 5 | 2 | 22 |
|  |  |  |  |  |  |  |  |  |  |  | EMB | 2 ug/mL | WHO_current | 4 | 33 |  | 3 | 5 | 1 | 28 |
|  |  |  |  |  |  |  |  |  |  |  | FLQ | LFX: 2 ug/mL MFX: 1 ug/mL OFX: 4 ug/mL | WHO_past | 5 | 32 |  | 3 | 1 | 2 | 31 |
|  |  |  |  |  |  |  |  |  |  |  | CS | 30 ug/mL | WHO_past | 0 | 37 |  | 0 | 0 | 0 | 37 |
|  |  |  |  |  |  |  |  |  |  |  | SM | 10 ug/mL | WHO_past | 11 | 26 |  | 8 | 1 | 3 | 25 |
|  |  |  |  |  |  |  |  |  |  |  | AMG | AMK: 30 ug/mL KM: 30 ug/mL CPM: 40 ug/mL | WHO_current | 1 | 36 |  | 1 | 0 | 0 | 36 |
|  |  |  |  |  |  |  |  |  |  |  | ETO | 40 ug/mL | WHO_current | 3 | 34 |  | 2 | 13 | 1 | 21 |
|  |  |  |  |  |  |  |  |  |  |  | PAS | 1 ug/mL | WHO_past | 0 | 37 |  | 0 | 2 | 0 | 35 |
|  |  |  |  |  |  |  |  |  |  | Pyrazinamidase test | PZA | NA | WHO_past | 0 | 37 |  | 0 | 6 | 0 | 31 |
|  |  |  |  |  |  |  |  |  |  | LJ | INH | 0.2 µg/mL | WHO_current | 17 | 20 | TGS-TB | 12 | 15 | 5 | 5 |
|  |  |  |  |  |  |  |  |  |  |  | RMP | 40 ug/mL | WHO_current | 10 | 27 |  | 8 | 5 | 2 | 22 |
|  |  |  |  |  |  |  |  |  |  |  | EMB | 2 ug/mL | WHO_current | 4 | 33 |  | 3 | 6 | 1 | 27 |
|  |  |  |  |  |  |  |  |  |  |  | FLQ | LFX: 2 ug/mL MFX: 1 ug/mL OFX: 4 ug/mL | WHO_past | 5 | 32 |  | 4 | 2 | 1 | 30 |
|  |  |  |  |  |  |  |  |  |  |  | CS | 30 ug/mL | WHO_past | 0 | 37 |  | 0 | 0 | 0 | 37 |
|  |  |  |  |  |  |  |  |  |  |  | SM | 10 ug/mL | WHO_past | 11 | 26 |  | 8 | 0 | 3 | 26 |
|  |  |  |  |  |  |  |  |  |  |  | AMG | AMK: 30 ug/mL KM: 30 ug/mL CPM: 40 ug/mL | WHO_current | 1 | 36 |  | 1 | 0 | 0 | 36 |
|  |  |  |  |  |  |  |  |  |  |  | ETO | 40 ug/mL | WHO_current | 3 | 34 |  | 2 | 14 | 1 | 20 |
|  |  |  |  |  |  |  |  |  |  |  | PAS | 1 ug/mL | WHO_past | 0 | 37 |  | 0 | 0 | 0 | 37 |
|  |  |  |  |  |  |  |  |  |  | Pyrazinamidase test | PZA | NA | WHO_past | 0 | 37 |  | 0 | 3 | 0 | 34 |
| 15 | Che, 2022 | 2018.1.1-2019.12.30 | China | Lineage 2, 89.83% | 59 | 49, 83.05% | Hiseq | 99.38%, 166.29X | PRJNA862152 | LJ | INH | 0.2 ug/mL | WHO_current | 49 | 10 | TBProfiler | 47 | 1 | 2 | 9 |
|  |  |  |  |  |  |  |  |  |  |  | RMP | 40 ug/mL | WHO_current | 59 | 0 |  | 59 | 0 | 0 | 0 |
|  |  |  |  |  |  |  |  |  |  |  | EMB | 2 ug/mL | WHO_current | 20 | 39 |  | 20 | 14 | 0 | 25 |
|  |  |  |  |  |  |  |  |  |  |  | SM | 4 ug/mL | WHO_current | 38 | 21 |  | 37 | 0 | 1 | 21 |
|  |  |  |  |  |  |  |  |  |  |  | AMK | 30 ug/mL | WHO_current | 8 | 51 |  | 6 | 0 | 2 | 51 |
|  |  |  |  |  |  |  |  |  |  |  | CPM | 40 ug/mL | WHO_current | 5 | 54 |  | 4 | 2 | 1 | 52 |
|  |  |  |  |  |  |  |  |  |  |  | LFX | 2 ug/mL | WHO_past | 23 | 36 |  | 23 | 1 | 0 | 35 |
|  |  |  |  |  |  |  |  |  |  |  | PTO | 40 ug/mL | WHO_current | 15 | 44 |  | 14 | 4 | 1 | 40 |
|  |  |  |  |  |  |  |  |  |  |  | PAS | 1 ug/mL | WHO_past | 10 | 39 |  | 7 | 1 | 3 | 38 |
| 16 | Wu, 2022 | 2019.8.1-2020.10.30 | China | Lineage 2, 90.1% | 182 | 59, 32.4% | Hiseq | NA | PRJNA806507 | MycoTB | RMP | 1 ug/mL | WHO_undefined | 68 | 114 | TBProfiler | 66 | 11 | 2 | 103 |
|  |  |  |  |  |  |  |  |  |  |  | INH | 0.2 ug/mL | WHO_undefined | 78 | 104 |  | 71 | 5 | 7 | 99 |
|  |  |  |  |  |  |  |  |  |  |  | EMB | 5 ug/mL | WHO_undefined | 33 | 149 |  | 33 | 19 | 0 | 130 |
|  |  |  |  |  |  |  |  |  |  |  | SM | 2 ug/mL | WHO_undefined | 59 | 123 |  | 57 | 47 | 2 | 76 |
|  |  |  |  |  |  |  |  |  |  |  | MFX | 0.5 ug/mL | WHO_undefined | 38 | 144 |  | 33 | 6 | 5 | 138 |
|  |  |  |  |  |  |  |  |  |  |  | OFX | 2 ug/mL | WHO_undefined | 37 | 145 |  | 32 | 7 | 5 | 138 |
|  |  |  |  |  |  |  |  |  |  |  | AMK | 4 ug/mL | WHO_undefined | 6 | 176 |  | 6 | 57 | 0 | 119 |
|  |  |  |  |  |  |  |  |  |  |  | KM | 5 ug/mL | WHO_undefined | 8 | 174 |  | 8 | 57 | 0 | 117 |
|  |  |  |  |  |  |  |  |  |  |  | ETO | 5 ug/mL | WHO_undefined | 8 | 174 |  | 5 | 20 | 3 | 154 |
|  |  |  |  |  |  |  |  |  |  |  | PAS | 2 ug/mL | WHO_undefined | 6 | 177 |  | 2 | 3 | 4 | 174 |
|  |  |  |  |  |  |  |  |  |  |  | CS | 25 ug/mL | WHO_undefined | 28 | 154 |  | 0 | 0 | 28 | 154 |
| 17.1 | Finci, 2022 | 2014.12.5-2017.12.12 | Peru, Vietnam, South Africa, Moldova, Georgia | Lineage 2, 56%; Lineage 4, 42% | 677 | 605, 89.36% | NextSeq or MiSeq | NA | PRJEB48275 | MGIT 960 | INH | 0.1 ug/mL | WHO_current | 635 | 39 | MTBseq | 626 | 3 | 9 | 36 |
|  |  |  |  |  |  |  |  |  |  |  | RMP | 1 ug/mL | WHO_current | 614 | 57 |  | 610 | 36 | 4 | 21 |
|  |  |  |  |  |  |  |  |  |  |  | KM | 2.5 ug/mL | WHO_current | 186 | 486 |  | 176 | 23 | 10 | 463 |
|  |  |  |  |  |  |  |  |  |  |  | AMK | 1 ug/mL | WHO_current | 36 | 636 |  | 34 | 10 | 2 | 626 |
|  |  |  |  |  |  |  |  |  |  |  | MFX | 0.5 ug/mL | WHO_past | 70 | 604 |  | 59 | 7 | 11 | 597 |
|  |  |  |  |  |  |  |  |  |  |  | LFX | 1.5 ug/mL | WHO_past | 42 | 613 |  | 41 | 9 | 1 | 604 |
| 17.2 |  |  |  |  |  |  |  |  |  | MycoTB | INH | 0.1 ug/mL | WHO_undefined | 173 | 49 |  | 172 | 0 | 1 | 49 |
|  |  |  |  |  |  |  |  |  |  |  | RMP | 1 ug/mL | WHO_undefined | 166 | 57 |  | 166 | 5 | 0 | 52 |
|  |  |  |  |  |  |  |  |  |  |  | KM | 2.5 ug/mL | WHO_undefined | 145 | 78 |  | 142 | 5 | 3 | 73 |
|  |  |  |  |  |  |  |  |  |  |  | AMK | 1 ug/mL | WHO_undefined | 145 | 78 |  | 142 | 0 | 3 | 78 |
|  |  |  |  |  |  |  |  |  |  |  | MFX | 0.5 ug/mL | WHO_undefined | 146 | 77 |  | 133 | 12 | 13 | 65 |
|  |  |  |  |  |  |  |  |  |  |  | LFX | 1 ug/mL | WHO_undefined | 54 | 15 |  | 50 | 8 | 4 | 7 |
| 18.1 | Hall, 2023 | 2011-2018 | Madagascar, South Africa | NA | 132 | NA | MiSeq, HiSeq 2500, or NextSeq500 | NA | PRJEB4909 | LJ or 7H | INH | LJ/7H: 0.2 ug/mL | WHO_current | 51 | 48 | Mykrobe (v.0.10.0) | 42 | 3 | 9 | 45 |
|  |  |  |  |  |  |  |  |  |  |  | RMP | 40 ug/mL | WHO_current | 48 | 44 |  | 42 | 1 | 6 | 43 |
|  |  |  |  |  |  |  |  |  |  |  | EMB | LJ: 2 ug/mL  7H: 7.5 ug/mL | WHO_current | 14 | 77 |  | 10 | 14 | 4 | 63 |
|  |  |  |  |  |  |  |  |  |  |  | SM | 4 ug/mL | WHO_current | 8 | 83 |  | 4 | 11 | 4 | 72 |
|  |  |  |  |  |  |  |  |  |  |  | AMK | 30 ug/mL | WHO_current | 11 | 78 |  | 10 | 2 | 1 | 76 |
|  |  |  |  |  |  |  |  |  |  |  | CPM | 40 ug/mL | WHO_current | 1 | 51 |  | 0 | 1 | 1 | 50 |
|  |  |  |  |  |  |  |  |  |  |  | KM | 30 ug/mL | WHO_current | 0 | 52 |  | 0 | 1 | 0 | 51 |
|  |  |  |  |  |  |  |  |  |  |  | OFX | 2 ug/mL | WHO_current | 10 | 77 |  | 10 | 4 | 0 | 73 |
| 18.2 |  |  |  |  |  |  | MinION or GridION |  |  |  | INH | LJ/7H: 0.2 ug/mL | WHO_current | 51 | 48 | Mykrobe (v.0.10.0) | 42 | 4 | 9 | 44 |
|  |  |  |  |  |  |  |  |  |  |  | RMP | 40 ug/mL | WHO_current | 48 | 44 |  | 42 | 1 | 6 | 43 |
|  |  |  |  |  |  |  |  |  |  |  | EMB | LJ: 2 ug/mL  7H: 7.5 ug/mL | WHO_current | 14 | 77 |  | 10 | 14 | 4 | 63 |
|  |  |  |  |  |  |  |  |  |  |  | SM | 4 ug/mL | WHO_current | 8 | 83 |  | 5 | 11 | 3 | 72 |
|  |  |  |  |  |  |  |  |  |  |  | AMK | 30 ug/mL | WHO_current | 11 | 78 |  | 11 | 2 | 0 | 76 |
|  |  |  |  |  |  |  |  |  |  |  | CPM | 40 ug/mL | WHO_current | 1 | 51 |  | 0 | 1 | 1 | 50 |
|  |  |  |  |  |  |  |  |  |  |  | KM | 30 ug/mL | WHO_current | 0 | 52 |  | 0 | 1 | 0 | 51 |
|  |  |  |  |  |  |  |  |  |  |  | OFX | 2 ug/mL | WHO_current | 10 | 77 |  | 10 | 4 | 0 | 73 |
| 19 | Lee, 2023 | 2015-2017 | Korea | Lineage 2, 86% | 57 | 38, 66.67% | Hiseq | NA | PRJNA219826 | LJ | RMP | 40 ug/mL | WHO_current | 37 | 20 | TBProfiler (v.2.8.5) | 36 | 0 | 1 | 20 |
|  |  |  |  |  |  |  |  |  |  |  | INH | 0.2 ug/mL | WHO_current | 38 | 19 |  | 35 | 1 | 3 | 18 |
|  |  |  |  |  |  |  |  |  |  |  | EMB | 2 ug/mL | WHO_current | 23 | 34 |  | 22 | 7 | 1 | 27 |
|  |  |  |  |  |  |  |  |  |  |  | SM | 10 ug/mL | WHO_past | 17 | 40 |  | 14 | 4 | 3 | 36 |
|  |  |  |  |  |  |  |  |  |  |  | FLQ | LFX: 2 ug/mL MFX: 1 ug/mL OFX: 4 ug/mL | WHO_past | 17 | 40 |  | 17 | 1 | 0 | 39 |
|  |  |  |  |  |  |  |  |  |  |  | AMG | KM: 30 ug/mL CPM: 40 ug/mL | WHO_current | 9 | 48 |  | 9 | 1 | 0 | 47 |
|  |  |  |  |  |  |  |  |  |  |  | ETO/PTO | 40 ug/mL | WHO_current | 10 | 47 |  | 10 | 8 | 0 | 39 |
|  |  |  |  |  |  |  |  |  |  |  | PAS | 1 ug/mL | WHO_past | 9 | 48 |  | 6 | 2 | 3 | 46 |
|  |  |  |  |  |  |  |  |  |  |  | CS | 30 ug/Ml | WHO_past | 2 | 55 |  | 0 | 1 | 2 | 54 |
|  |  |  |  |  |  |  |  |  |  |  | LZD | 2.0 ug/mL | WHO_undefined | 1 | 56 |  | 1 | 0 | 0 | 56 |
|  |  |  |  |  |  |  |  |  |  | Pyrazinamidase test | PZA | NA | WHO_past | 19 | 38 |  | 15 | 3 | 4 | 35 |
| 20 | Wang, 2023 | 2019.7-2021.6 | China | Lineage 2, 83.7% | 202 | 202, 100% | NovaSeq 6000 | 99.3%, 193.5X | PRJNA1000054 | LJ | RMP | 40 ug/mL | WHO_current | 202 | 0 | TBProfiler (v.2.8.12) | 200 | 0 | 2 | 0 |
|  |  |  |  |  |  |  |  |  |  |  | INH | 0.2 ug/mL | WHO_current | 202 | 0 |  | 191 | 0 | 11 | 0 |
|  |  |  |  |  |  |  |  |  |  |  | EMB | 2 ug/mL | WHO_current | 92 | 110 |  | 86 | 39 | 6 | 71 |
|  |  |  |  |  |  |  |  |  |  |  | SM | 4 ug/mL | WHO_current | 122 | 80 |  | 98 | 2 | 24 | 78 |
|  |  |  |  |  |  |  |  |  |  |  | OFX | 2 ug/mL | WHO_past | 99 | 103 |  | 91 | 6 | 8 | 97 |
|  |  |  |  |  |  |  |  |  |  |  | KM | 30 ug/mL | WHO_current | 15 | 187 |  | 15 | 0 | 0 | 187 |
|  |  |  |  |  |  |  |  |  |  |  | CPM | 40 ug/mL | WHO_current | 16 | 186 |  | 15 | 3 | 1 | 183 |
|  |  |  |  |  |  |  |  |  |  |  | ETO | 40 ug/mL | WHO_current | 23 | 179 |  | 17 | 21 | 6 | 158 |
| 21 | Xiao, 2023 | 2013-2016 | China | Lineage 2, 66% | 200 | 136, 69% | Miseq | NA | PRJNA879962 | 7H10 | RMP | 1 ug/mL | WHO_current | 197 | 3 | TGS-TB (v.2) | 196 | 3 | 1 | 0 |
|  |  |  |  |  |  |  |  |  |  |  | INH | 0.2 ug/mL | WHO_current | 136 | 64 |  | 131 | 5 | 5 | 59 |
|  |  |  |  |  |  |  |  |  |  |  | EMB | 5 ug/mL | WHO_current | 77 | 123 |  | 74 | 22 | 3 | 101 |
|  |  |  |  |  |  |  |  |  |  |  | SM | 2 ug/mL | WHO_current | 60 | 140 |  | 58 | 22 | 2 | 118 |
|  |  |  |  |  |  |  |  |  |  |  | FLQ | MFX, 0.5 ug/mL OFX, 2 ug/mL | WHO_past | 22 | 178 |  | 22 | 1 | 0 | 177 |
|  |  |  |  |  |  |  |  |  |  | 7H11 | KM | 6 ug/mL | WHO_past | 11 | 189 |  | 9 | 6 | 2 | 183 |
|  |  |  |  |  |  |  |  |  |  |  | AMK | 6 ug/mL | WHO_undefined | 8 | 192 |  | 7 | 0 | 1 | 192 |
|  |  |  |  |  |  |  |  |  |  |  | CPM | 10 ug/mL | WHO_undefined | 6 | 194 |  | 5 | 3 | 1 | 191 |
|  |  |  |  |  |  |  |  |  |  |  | ETO | 10 ug/mL | WHO_current | 34 | 166 |  | 33 | 31 | 1 | 135 |
|  |  |  |  |  |  |  |  |  |  |  | PAS | 8 ug/mL | WHO_past | 6 | 194 |  | 6 | 1 | 0 | 193 |
|  |  |  |  |  |  |  |  |  |  | MGIT 960 | PZA | 100 ug/mL | WHO_current | 40 | 160 |  | 39 | 2 | 1 | 158 |
| 22 | Morey-León, 2023 | 2019-2021 | Ecuador | Lineage 4, 100% | 88 | 52, 59.1% | MiniSeq | NA | PRJNA827129 | MGIT 960 and/or LJ | INH | MGIT: 0.1 ug/mL LJ: 0.2 ug/mL | WHO_current | 63 | 25 | KvarQ (v.0.12.2) | 58 | 1 | 5 | 24 |
|  |  |  |  |  |  |  |  |  |  |  | RMP | MGIT: 1 ug/mL LJ: 40 ug/mL | WHO_current | 66 | 22 |  | 58 | 0 | 8 | 22 |
|  |  |  |  |  |  |  |  |  |  |  | EMB | MGIT: 5 ug/mL LJ: 0.4 ug/mL | WHO_current | 11 | 76 |  | 8 | 5 | 3 | 71 |
|  |  |  |  |  |  |  |  |  |  |  | SM | LJ: 4 ug/mL | WHO_current | 16 | 5 |  | 9 | 0 | 7 | 5 |
|  |  |  |  |  |  |  |  |  |  |  | KM | MGIT: 2.5 ug/mL | WHO_current | 0 | 45 |  | 0 | 0 | 0 | 45 |
|  |  |  |  |  |  |  |  |  |  |  | AMK | MGIT: 1 ug/mL | WHO_current | 0 | 21 |  | 0 | 0 | 0 | 21 |
|  |  |  |  |  |  |  |  |  |  |  | CPM | MGIT: 1 ug/mL | WHO_past | 0 | 21 |  | 0 | 0 | 0 | 21 |
|  |  |  |  |  |  |  |  |  |  |  | LFX | MGIT: 1 ug/mL | WHO_past | 7 | 39 |  | 3 | 0 | 4 | 39 |
|  |  |  |  |  |  |  |  |  |  |  | MFX | MGIT: 1 ug/mL | WHO_past | 2 | 19 |  | 1 | 1 | 1 | 18 |
|  |  |  |  |  |  |  |  |  |  | Pyrazinamidase test | PZA | 200 ug/mL | WHO_past | 12 | 52 |  | 8 | 6 | 4 | 46 |
|  |  |  |  |  |  |  |  |  |  | MGIT 960 and/or LJ | INH | MGIT: 0.1 ug/mL LJ: 0.2 ug/mL | WHO_current | 63 | 25 | Mykrobe (v.0.12.2) | 60 | 1 | 3 | 24 |
|  |  |  |  |  |  |  |  |  |  |  | RMP | MGIT: 1 ug/mL LJ: 40 ug/mL | WHO_current | 66 | 22 |  | 63 | 0 | 3 | 22 |
|  |  |  |  |  |  |  |  |  |  |  | EMB | MGIT: 5 ug/mL LJ: 0.4 ug/mL | WHO_current | 11 | 76 |  | 11 | 10 | 0 | 66 |
|  |  |  |  |  |  |  |  |  |  |  | SM | LJ: 4 ug/mL | WHO_current | 16 | 5 |  | 9 | 0 | 7 | 5 |
|  |  |  |  |  |  |  |  |  |  |  | KM | MGIT: 2.5 ug/mL | WHO_current | 0 | 45 |  | 0 | 2 | 0 | 43 |
|  |  |  |  |  |  |  |  |  |  |  | AMK | MGIT: 1 ug/mL | WHO_current | 0 | 21 |  | 0 | 1 | 0 | 20 |
|  |  |  |  |  |  |  |  |  |  |  | CPM | MGIT: 1 ug/mL | WHO_past | 0 | 21 |  | 0 | 1 | 0 | 20 |
|  |  |  |  |  |  |  |  |  |  |  | LFX | MGIT: 1 ug/mL | WHO_past | 7 | 39 |  | 3 | 1 | 4 | 38 |
|  |  |  |  |  |  |  |  |  |  |  | MFX | MGIT: 1 ug/mL | WHO_past | 2 | 19 |  | 1 | 2 | 1 | 17 |
|  |  |  |  |  |  |  |  |  |  | Pyrazinamidase test | PZA | 200 ug/mL | WHO_past | 12 | 52 |  | 10 | 3 | 2 | 49 |
|  |  |  |  |  |  |  |  |  |  | MGIT 960 and/or LJ | INH | MGIT: 0.1 ug/mL LJ: 0.2 ug/mL | WHO_current | 63 | 25 | PhyResSE (v.1.0) | 60 | 1 | 3 | 24 |
|  |  |  |  |  |  |  |  |  |  |  | RMP | MGIT: 1 ug/mL LJ: 40 ug/mL | WHO_current | 66 | 22 |  | 64 | 0 | 2 | 22 |
|  |  |  |  |  |  |  |  |  |  |  | EMB | MGIT: 5 ug/mL LJ: 0.4 ug/mL | WHO_current | 11 | 76 |  | 10 | 11 | 1 | 65 |
|  |  |  |  |  |  |  |  |  |  |  | SM | LJ: 4 ug/mL | WHO_current | 16 | 5 |  | 11 | 0 | 5 | 5 |
|  |  |  |  |  |  |  |  |  |  |  | KM | MGIT: 2.5 ug/mL | WHO_current | 0 | 45 |  | 0 | 2 | 0 | 43 |
|  |  |  |  |  |  |  |  |  |  |  | AMK | MGIT: 1 ug/mL | WHO_current | 0 | 21 |  | 0 | 1 | 0 | 20 |
|  |  |  |  |  |  |  |  |  |  |  | CPM | MGIT: 1 ug/mL | WHO_past | 0 | 21 |  | 0 | 1 | 0 | 20 |
|  |  |  |  |  |  |  |  |  |  |  | LFX | MGIT: 1 ug/mL | WHO_past | 7 | 39 |  | 3 | 1 | 4 | 38 |
|  |  |  |  |  |  |  |  |  |  |  | MFX | MGIT: 1 ug/mL | WHO_past | 2 | 19 |  | 1 | 2 | 1 | 17 |
|  |  |  |  |  |  |  |  |  |  | Pyrazinamidase test | PZA | 200 ug/mL | WHO_past | 12 | 52 |  | 9 | 7 | 3 | 45 |
|  |  |  |  |  |  |  |  |  |  | MGIT 960 and/or LJ | INH | MGIT: 0.1 ug/mL LJ: 0.2 ug/mL | WHO_current | 63 | 25 | TBProfiler (v.5) | 59 | 1 | 4 | 24 |
|  |  |  |  |  |  |  |  |  |  |  | RMP | MGIT: 1 ug/mL LJ: 40 ug/mL | WHO_current | 66 | 22 |  | 66 | 3 | 0 | 19 |
|  |  |  |  |  |  |  |  |  |  |  | EMB | MGIT: 5 ug/mL LJ: 0.4 ug/mL | WHO_current | 11 | 76 |  | 11 | 11 | 0 | 65 |
|  |  |  |  |  |  |  |  |  |  |  | SM | LJ: 4 ug/mL | WHO_current | 16 | 5 |  | 11 | 0 | 5 | 5 |
|  |  |  |  |  |  |  |  |  |  |  | KM | MGIT: 2.5 ug/mL | WHO_current | 0 | 45 |  | 0 | 2 | 0 | 43 |
|  |  |  |  |  |  |  |  |  |  |  | AMK | MGIT: 1 ug/mL | WHO_current | 0 | 21 |  | 0 | 1 | 0 | 20 |
|  |  |  |  |  |  |  |  |  |  |  | CPM | MGIT: 1 ug/mL | WHO_past | 0 | 21 |  | 0 | 1 | 0 | 20 |
|  |  |  |  |  |  |  |  |  |  |  | LFX | MGIT: 1 ug/mL | WHO_past | 7 | 39 |  | 3 | 1 | 4 | 38 |
|  |  |  |  |  |  |  |  |  |  |  | MFX | MGIT: 1 ug/mL | WHO_past | 2 | 19 |  | 1 | 2 | 1 | 17 |
|  |  |  |  |  |  |  |  |  |  | Pyrazinamidase test | PZA | 200 ug/mL | WHO_past | 12 | 52 |  | 10 | 7 | 2 | 45 |
|  |  |  |  |  |  |  |  |  |  | MGIT 960 and/or LJ | INH | MGIT: 0.1 ug/mL LJ: 0.2 ug/mL | WHO_current | 63 | 25 | SAM-TB | 60 | 1 | 3 | 24 |
|  |  |  |  |  |  |  |  |  |  |  | RMP | MGIT: 1 ug/mL LJ: 40 ug/mL | WHO_current | 66 | 22 |  | 64 | 0 | 2 | 22 |
|  |  |  |  |  |  |  |  |  |  |  | EMB | MGIT: 5 ug/mL LJ: 0.4 ug/mL | WHO_current | 11 | 76 |  | 10 | 11 | 1 | 65 |
|  |  |  |  |  |  |  |  |  |  |  | SM | LJ: 4 ug/mL | WHO_current | 16 | 5 |  | 12 | 0 | 4 | 5 |
|  |  |  |  |  |  |  |  |  |  |  | KM | MGIT: 2.5 ug/mL | WHO_current | 0 | 45 |  | 0 | 0 | 0 | 45 |
|  |  |  |  |  |  |  |  |  |  |  | AMK | MGIT: 1 ug/mL | WHO_current | 0 | 21 |  | 0 | 1 | 0 | 20 |
|  |  |  |  |  |  |  |  |  |  |  | CPM | MGIT: 1 ug/mL | WHO_past | 0 | 21 |  | 0 | 1 | 0 | 20 |
|  |  |  |  |  |  |  |  |  |  |  | LFX | MGIT: 1 ug/mL | WHO_past | 7 | 39 |  | 5 | 1 | 2 | 38 |
|  |  |  |  |  |  |  |  |  |  |  | MFX | MGIT: 1 ug/mL | WHO_past | 2 | 19 |  | 1 | 4 | 1 | 15 |
|  |  |  |  |  |  |  |  |  |  | Pyrazinamidase test | PZA | 200 ug/mL | WHO_past | 12 | 52 |  | 12 | 8 | 0 | 44 |
| 23 | Billard-Pomares, 2022 | 2015-2021 | France | Lineage 4, 68% | 227 | 4, 1.76% | Miseq | 98%, 119X | PRJEB52390 | MGIT 960 | RMP | 1 ug/mL | WHO_current | 5 | 222 | TBProﬁler (v.2.8.12) | 5 | 0 | 0 | 222 |
|  |  |  |  |  |  |  |  |  |  |  | INH | 0.1 ug/mL | WHO_current | 18 | 209 |  | 18 | 0 | 0 | 209 |
|  |  |  |  |  |  |  |  |  |  |  | EMB | 5 ug/mL | WHO_current | 1 | 226 |  | 1 | 2 | 0 | 224 |
|  |  |  |  |  |  |  |  |  |  |  | PZA | 100 ug/mL | WHO_current | 12 | 215 |  | 8 | 0 | 4 | 215 |
| 24 | Quagliaro, 2023 |  |  |  |  |  |  |  |  |  | RMP | 1 ug/mL | WHO_current | 5 | 222 | Mykrobe | 4 | 2 | 1 | 220 |
|  |  |  |  |  |  |  |  |  |  |  | INH | 0.1 ug/mL | WHO_current | 18 | 209 |  | 15 | 0 | 3 | 209 |
|  |  |  |  |  |  |  |  |  |  |  | EMB | 5 ug/mL | WHO_current | 1 | 226 |  | 1 | 1 | 0 | 225 |
|  |  |  |  |  |  |  |  |  |  |  | PZA | 100 ug/mL | WHO_current | 12 | 215 |  | 8 | 0 | 4 | 215 |
|  |  |  |  |  |  |  |  |  |  |  | RMP | 1 ug/mL | WHO_current | 5 | 222 | PhyResSE | 5 | 0 | 0 | 222 |
|  |  |  |  |  |  |  |  |  |  |  | INH | 0.1 ug/mL | WHO_current | 18 | 209 |  | 15 | 0 | 3 | 209 |
|  |  |  |  |  |  |  |  |  |  |  | EMB | 5 ug/mL | WHO_current | 1 | 226 |  | 1 | 2 | 0 | 224 |
|  |  |  |  |  |  |  |  |  |  |  | PZA | 100 ug/mL | WHO_current | 12 | 215 |  | 8 | 3 | 4 | 212 |
| 25 | Daniyarov, 2023 | 2022.1-2022.12 | Kazakhstan | Lineage 2, 100% | 10 | 10, 100% | MiSeq | 98.95%, 160X | PRJNA481625 | MGIT 960 | INH | 0.1 ug/mL | WHO_current | 10 | 0 | CASTB | 10 | 0 | 0 | 0 |
|  |  |  |  |  |  |  |  |  |  |  | RMP | 1 ug/mL | WHO_current | 10 | 0 |  | 9 | 0 | 0 | 0 |
|  |  |  |  |  |  |  |  |  |  |  | EMB | 5 ug/mL | WHO_current | 4 | 6 |  | 4 | 5 | 0 | 0 |
|  |  |  |  |  |  |  |  |  |  |  | SM | 1 ug/mL | WHO_current | 3 | 7 |  | 3 | 7 | 0 | 0 |
|  |  |  |  |  |  |  |  |  |  |  | PZA | 100 ug/mL | WHO_current | 9 | 1 |  | - | - | - | - |
|  |  |  |  |  |  |  |  |  |  |  | ETO | 5 ug/mL | WHO_current | 4 | 6 |  | - | - | - | - |
|  |  |  |  |  |  |  |  |  |  |  | MFX | 0.25 ug/mL | WHO_current | 10 | 0 |  | - | - | - | - |
|  |  |  |  |  |  |  |  |  |  |  | LFX | 1 ug/mL | WHO_past | 8 | 2 |  | - | - | - | - |
|  |  |  |  |  |  |  |  |  |  |  | AMK | 1 ug/mL | WHO_current | 2 | 8 |  | 2 | 0 | 0 | 0 |
|  |  |  |  |  |  |  |  |  |  |  | CPM | 2.5 ug/mL | WHO_current | 3 | 7 |  | - | - | - | - |
|  |  |  |  |  |  |  |  |  |  |  | KM | 2.5 ug/mL | WHO_current | 3 | 7 |  | - | - | - | - |
|  |  |  |  |  |  |  |  |  |  |  | INH | 0.1 ug/mL | WHO_current | 10 | 0 | Mykrobe | 10 | 0 | 0 | 0 |
|  |  |  |  |  |  |  |  |  |  |  | RMP | 1 ug/mL | WHO_current | 10 | 0 |  | 10 | 0 | 0 | 0 |
|  |  |  |  |  |  |  |  |  |  |  | EMB | 5 ug/mL | WHO_current | 4 | 6 |  | 4 | 5 | 0 | 1 |
|  |  |  |  |  |  |  |  |  |  |  | SM | 1 ug/mL | WHO_current | 3 | 7 |  | 3 | 7 | 0 | 0 |
|  |  |  |  |  |  |  |  |  |  |  | PZA | 100 ug/mL | WHO_current | 9 | 1 |  | 7 | 0 | 2 | 1 |
|  |  |  |  |  |  |  |  |  |  |  | ETO | 5 ug/mL | WHO_current | 4 | 6 |  | - | - | - | - |
|  |  |  |  |  |  |  |  |  |  |  | MFX | 0.25 ug/mL | WHO_current | 10 | 0 |  | 10 | 0 | 0 | 0 |
|  |  |  |  |  |  |  |  |  |  |  | LFX | 1 ug/mL | WHO_past | 8 | 2 |  | - | - | - | - |
|  |  |  |  |  |  |  |  |  |  |  | AMK | 1 ug/mL | WHO_current | 2 | 8 |  | 2 | 0 | 0 | 8 |
|  |  |  |  |  |  |  |  |  |  |  | CPM | 2.5 ug/mL | WHO_current | 3 | 7 |  | 2 | 0 | 1 | 7 |
|  |  |  |  |  |  |  |  |  |  |  | KM | 2.5 ug/mL | WHO_current | 3 | 7 |  | 2 | 1 | 1 | 6 |
|  |  |  |  |  |  |  |  |  |  |  | INH | 0.1 ug/mL | WHO_current | 10 | 0 | TBProﬁle | 10 | 0 | 0 | 0 |
|  |  |  |  |  |  |  |  |  |  |  | RMP | 1 ug/mL | WHO_current | 10 | 0 |  | 10 | 0 | 0 | 0 |
|  |  |  |  |  |  |  |  |  |  |  | EMB | 5 ug/mL | WHO_current | 4 | 6 |  | 4 | 6 | 0 | 0 |
|  |  |  |  |  |  |  |  |  |  |  | SM | 1 ug/mL | WHO_current | 3 | 7 |  | 3 | 7 | 0 | 0 |
|  |  |  |  |  |  |  |  |  |  |  | PZA | 100 ug/mL | WHO_current | 9 | 1 |  | - | - | - | - |
|  |  |  |  |  |  |  |  |  |  |  | ETO | 5 ug/mL | WHO_current | 4 | 6 |  | 1 | 2 | 0 | 0 |
|  |  |  |  |  |  |  |  |  |  |  | MFX | 0.25 ug/mL | WHO_current | 10 | 0 |  | 10 | 0 | 0 | 0 |
|  |  |  |  |  |  |  |  |  |  |  | LFX | 1 ug/mL | WHO_past | 8 | 2 |  | 8 | 2 | 0 | 0 |
|  |  |  |  |  |  |  |  |  |  |  | AMK | 1 ug/mL | WHO_current | 2 | 8 |  | 2 | 0 | 0 | 0 |
|  |  |  |  |  |  |  |  |  |  |  | CPM | 2.5 ug/mL | WHO_current | 3 | 7 |  | 2 | 0 | 0 | 0 |
|  |  |  |  |  |  |  |  |  |  |  | KM | 2.5 ug/mL | WHO_current | 3 | 7 |  | 3 | 2 | 0 | 0 |
| 26 | Lim, 2023 | 2019-2022 | Singapore | Lineage 1, 35%; Lineage 2, 48%; Lineage 4, 14% | 3808 | 58, 1.5% | MiSeq | NA | NA | MGIT | RIF | NA | NA | 62 | 2934 | TBProfiler | 61 | 12 | 1 | 2922 |
|  |  |  |  |  |  |  |  |  |  |  | INH | NA | NA | 213 | 2781 |  | 202 | 11 | 11 | 2770 |
|  |  |  |  |  |  |  |  |  |  |  | EMB | NA | NA | 14 | 2984 |  | 13 | 38 | 1 | 2946 |
|  |  |  |  |  |  |  |  |  |  |  | PZA | NA | NA | 46 | 2092 |  | 30 | 2 | 16 | 2090 |
| 27 | Shaw, 2023 | NA | USA | Lineage 2, 26%; Lineage 4, 47% | 38 | 3, 7.9% | MiSeq | 95.6%, 15X | NA | MGIT and solid agar | RIF | NA | NA | 1 | 37 | TBProfiler | 1 | 0 | 0 | 37 |
|  |  |  |  |  |  |  |  |  |  |  | INH | NA | NA | 8 | 30 |  | 7 | 1 | 1 | 29 |
|  |  |  |  |  |  |  |  |  |  |  | EMB | NA | NA | 6 | 32 |  | 5 | 0 | 1 | 32 |
|  |  |  |  |  |  |  |  |  |  |  | PZA | NA | NA | 1 | 37 |  | 1 | 0 | 0 | 37 |
|  |  |  |  |  |  |  |  |  |  |  | SM | NA | NA | 5 | 33 |  | 5 | 0 | 0 | 33 |
| 28 | Cloutier Charette, 2024 | 2012-2017 | Madagascar | NA | 72 | 32, 44.44% | HiSeq | NA, 30X | NA | LJ | RMP | NA |  | 33 | 39 | Mykrobe (v.0.12.1) | 31 | 0 | 2 | 39 |
|  |  |  |  |  |  |  |  |  |  |  | INH | NA | NA | 34 | 38 |  | 30 | 1 | 4 | 37 |
|  |  |  |  |  |  |  |  |  |  |  | EMB | NA | NA | 12 | 60 |  | 9 | 11 | 3 | 49 |
|  |  |  |  |  |  |  |  |  |  |  | SM | NA | NA | 5 | 65 |  | 4 | 10 | 1 | 55 |
|  |  |  |  |  |  |  |  |  |  |  | FLQ | NA | NA | 0 | 36 |  | 0 | 0 | 0 | 36 |
|  |  |  |  |  |  |  |  |  |  |  | AMK | NA | NA | 0 | 36 |  | 0 | 1 | 0 | 35 |
|  |  |  |  |  |  |  |  |  |  |  | CPM | NA | NA | 1 | 35 |  | 0 | 1 | 1 | 34 |
|  |  |  |  |  |  |  |  |  |  |  | KM | NA | NA | 0 | 36 |  | 0 | 1 | 0 | 35 |
| 29 | He, 2024 | 2014.1.1-2017.6.30 | China | NA | 110 | 100, 90.91% | Sanger | NA | NA | LJ | INH | 0.2 ug/mL | WHO_current | 95 | 15 | TBProfiler (v.3.0.7) | 95 | 6 | 0 | 9 |
|  |  |  |  |  |  |  |  |  |  |  | RMP | 40 ug/mL | WHO_current | 97 | 13 |  | 97 | 3 | 0 | 10 |
|  |  |  |  |  |  |  |  |  |  |  | EMB | 2 ug/mL | WHO_current | 75 | 35 |  | 56 | 2 | 19 | 33 |
|  |  |  |  |  |  |  |  |  |  |  | SM | 4 ug/mL | WHO_current | 65 | 45 |  | 64 | 5 | 1 | 40 |
|  |  |  |  |  |  |  |  |  |  |  | PAS | 1 ug/mL | WHO_past | 8 | 102 |  | 7 | 0 | 1 | 102 |
|  |  |  |  |  |  |  |  |  |  |  | AMK | 30 ug/mL | WHO_current | 9 | 101 |  | 9 | 2 | 0 | 99 |
|  |  |  |  |  |  |  |  |  |  |  | KM | 30 ug/mL | WHO_current | 10 | 100 |  | 9 | 2 | 1 | 98 |
|  |  |  |  |  |  |  |  |  |  |  | CPM | 40 ug/mL | WHO_current | 9 | 101 |  | 8 | 1 | 1 | 100 |
|  |  |  |  |  |  |  |  |  |  | MGIT 960 | PZA | 100 ug/mL | WHO_current | 59 | 51 |  | 55 | 10 | 4 | 41 |
|  |  |  |  |  |  |  |  |  |  |  | PTO | 2.5 ug/mL | WHO_current | 14 | 96 |  | 7 | 16 | 7 | 80 |
|  |  |  |  |  |  |  |  |  |  | LJ | INH | 0.2 ug/mL | WHO_current | 90 | 20 | SAM-TB | 90 | 11 | 0 | 9 |
|  |  |  |  |  |  |  |  |  |  |  | RMP | 40 ug/mL | WHO_current | 97 | 13 |  | 97 | 3 | 0 | 10 |
|  |  |  |  |  |  |  |  |  |  |  | EMB | 2 ug/mL | WHO_current | 71 | 39 |  | 53 | 5 | 18 | 34 |
|  |  |  |  |  |  |  |  |  |  |  | SM | 4 ug/mL | WHO_current | 65 | 45 |  | 64 | 5 | 1 | 40 |
|  |  |  |  |  |  |  |  |  |  |  | PAS | 1 ug/mL | WHO_past | 6 | 104 |  | 5 | 2 | 1 | 102 |
|  |  |  |  |  |  |  |  |  |  |  | AMK | 30 ug/mL | WHO_current | 9 | 101 |  | 9 | 2 | 0 | 99 |
|  |  |  |  |  |  |  |  |  |  |  | KM | 30 ug/mL | WHO_current | 9 | 101 |  | 9 | 2 | 0 | 99 |
|  |  |  |  |  |  |  |  |  |  |  | CPM | 40 ug/mL | WHO_current | 9 | 101 |  | 8 | 1 | 1 | 100 |
|  |  |  |  |  |  |  |  |  |  | MGIT 960 | PZA | 100 ug/mL | WHO_current | 46 | 64 |  | 42 | 23 | 4 | 41 |
|  |  |  |  |  |  |  |  |  |  |  | PTO | 2.5 ug/mL | WHO_current | 1 | 109 |  | 1 | 22 | 0 | 87 |
|  |  |  |  |  |  |  |  |  |  | LJ | INH | 0.2 ug/mL | WHO_current | 82 | 28 | GenTB (v.7.24.0) | 82 | 19 | 0 | 9 |
|  |  |  |  |  |  |  |  |  |  |  | RMP | 40 ug/mL | WHO_current | 85 | 25 |  | 85 | 15 | 0 | 10 |
|  |  |  |  |  |  |  |  |  |  |  | EMB | 2 ug/mL | WHO_current | 49 | 61 |  | 39 | 19 | 10 | 42 |
|  |  |  |  |  |  |  |  |  |  |  | SM | 4 ug/mL | WHO_current | 61 | 49 |  | 61 | 8 | 0 | 41 |
|  |  |  |  |  |  |  |  |  |  |  | PAS | 1 ug/mL | WHO_past | / | / |  | / | / | / | / |
|  |  |  |  |  |  |  |  |  |  |  | AMK | 30 ug/mL | WHO_current | 9 | 101 |  | 9 | 2 | 0 | 99 |
|  |  |  |  |  |  |  |  |  |  |  | KM | 30 ug/mL | WHO_current | 11 | 99 |  | 9 | 2 | 2 | 97 |
|  |  |  |  |  |  |  |  |  |  |  | CPM | 40 ug/mL | WHO_current | 11 | 99 |  | 8 | 1 | 3 | 98 |
|  |  |  |  |  |  |  |  |  |  | MGIT 960 | PZA | 100 ug/mL | WHO_current | 22 | 88 |  | 21 | 44 | 1 | 44 |
|  |  |  |  |  |  |  |  |  |  |  | PTO | 2.5 ug/mL | WHO_current | 1 | 109 |  | 0 | 23 | 1 | 86 |
|  |  |  |  |  |  |  |  |  |  | LJ | INH | 0.2 ug/mL | WHO_current | 88 | 22 | PhyResSE (v.1.0); | 88 | 13 | 0 | 9 |
|  |  |  |  |  |  |  |  |  |  |  | RMP | 40 ug/mL | WHO_current | 92 | 18 |  | 92 | 8 | 0 | 10 |
|  |  |  |  |  |  |  |  |  |  |  | EMB | 2 ug/mL | WHO_current | 64 | 46 |  | 49 | 9 | 15 | 37 |
|  |  |  |  |  |  |  |  |  |  |  | SM | 4 ug/mL | WHO_current | 53 | 57 |  | 53 | 16 | 0 | 41 |
|  |  |  |  |  |  |  |  |  |  |  | PAS | 1 ug/mL | WHO_past | / | / |  | / | / | / | / |
|  |  |  |  |  |  |  |  |  |  |  | AMK | 30 ug/mL | WHO_current | 9 | 101 |  | 9 | 2 | 0 | 99 |
|  |  |  |  |  |  |  |  |  |  |  | KM | 30 ug/mL | WHO_current | 10 | 100 |  | 9 | 2 | 1 | 98 |
|  |  |  |  |  |  |  |  |  |  |  | CPM | 40 ug/mL | WHO_current | 9 | 101 |  | 8 | 1 | 1 | 100 |
|  |  |  |  |  |  |  |  |  |  | MGIT 960 | PZA | 100 ug/mL | WHO_current | 18 | 92 |  | 16 | 49 | 2 | 43 |
|  |  |  |  |  |  |  |  |  |  |  | PTO | 2.5 ug/mL | WHO_current | / | / |  | / | / | / | / |
|  |  |  |  |  |  |  |  |  |  | LJ | INH | 0.2 ug/mL | WHO_current | 94 | 16 | Mykrobe (v 0.12.1) | 94 | 7 | 0 | 9 |
|  |  |  |  |  |  |  |  |  |  |  | RMP | 40 ug/mL | WHO_current | 97 | 13 |  | 97 | 3 | 0 | 10 |
|  |  |  |  |  |  |  |  |  |  |  | EMB | 2 ug/mL | WHO_current | 73 | 37 |  | 55 | 3 | 18 | 34 |
|  |  |  |  |  |  |  |  |  |  |  | SM | 4 ug/mL | WHO_current | 61 | 49 |  | 61 | 8 | 0 | 41 |
|  |  |  |  |  |  |  |  |  |  |  | PAS | 1 ug/mL | WHO_past | / | / |  | / | / | / | / |
|  |  |  |  |  |  |  |  |  |  |  | AMK | 30 ug/mL | WHO_current | 7 | 103 |  | 7 | 4 | 0 | 99 |
|  |  |  |  |  |  |  |  |  |  |  | KM | 30 ug/mL | WHO_current | 8 | 102 |  | 7 | 4 | 1 | 98 |
|  |  |  |  |  |  |  |  |  |  |  | CPM | 40 ug/mL | WHO_current | 7 | 103 |  | 7 | 2 | 0 | 101 |
|  |  |  |  |  |  |  |  |  |  | MGIT 960 | PZA | 100 ug/mL | WHO_current | 45 | 65 |  | 43 | 22 | 2 | 43 |
|  |  |  |  |  |  |  |  |  |  |  | PTO | 2.5 ug/mL | WHO_current | 30 | 80 |  | 14 | 9 | 16 | 71 |
| 30 | Liu, 2024 | 2019-2022 | Taiwan,China | Lineage 1, 9.7%; Lineage 2, 63.3%; Lineage 4, 27% | 297 | 297, 100% | MiSeq | NA, 100X | PRJNA1141184 | 7H10 | INH | 0.2 ug/mL | WHO_current | 297 | 0 | TBProfiler (v.4.1.1) | 295 | 0 | 2 | 0 |
|  |  |  |  |  |  |  |  |  |  |  | RMP | 1 ug/mL | WHO_current | 297 | 0 |  | 297 | 0 | 0 | 0 |
|  |  |  |  |  |  |  |  |  |  |  | EMB | 5 ug/mL | WHO_current | 148 | 149 |  | 139 | 37 | 9 | 112 |
|  |  |  |  |  |  |  |  |  |  |  | SM | 2 ug/mL | WHO_current | 137 | 160 |  | 119 | 23 | 18 | 137 |
|  |  |  |  |  |  |  |  |  |  |  | ETO | 10 ug/mL | WHO_current | 93 | 204 |  | 83 | 48 | 10 | 156 |
|  |  |  |  |  |  |  |  |  |  |  | FLQ | LFX: 1 ug/mL; MFX, 0.5ug/mL | WHO_current | 35 | 262 |  | 34 | 0 | 1 | 262 |
|  |  |  |  |  |  |  |  |  |  |  | AMK | 6 ug/mL | WHO_past | 12 | 285 |  | 12 | 3 | 0 | 282 |
|  |  |  |  |  |  |  |  |  |  |  | CPM | 6 ug/mL | WHO_past | 13 | 284 |  | 12 | 1 | 1 | 283 |
|  |  |  |  |  |  |  |  |  |  |  | KM | 10 ug/mL | WHO_past | 20 | 277 |  | 18 | 4 | 2 | 273 |
|  |  |  |  |  |  |  |  |  |  |  | PAS | 8 ug/mL | WHO_past | 12 | 285 |  | 10 | 13 | 2 | 272 |
|  |  |  |  |  |  |  |  |  |  | MGIT 960 | PZA | 100 ug/mL | WHO_current | 84 | 213 |  | 82 | 6 | 2 | 207 |
| 31 | Rukmana, 2024 | NA | Indonesia | Lineage 1, 5%; Lineage 2, 46.67%; Lineage 4, 48.33% | 60 | 7, 11.7% | NovaSeq 6000 | NA | NA | MGIT 960 | BDQ | NA | NA | 3 | 57 | Mykrobe (v.0.10) | 3 | 0 | 0 | 57 |
| 32 | Rukmana, 2024 | NA | Indonesia | Lineage 1, 5.9%; Lineage 2, 61.7%; Lineage 4, 32.4% | 34 | NA | NovaSeq 6000 | NA | NA | MGIT 960 | PZA | 100 ug/mL | WHO_current | 8 | 26 | GenTB | 7 | 1 | 1 | 25 |
|  |  |  |  |  |  |  |  |  |  |  |  |  |  | 8 | 26 | Mykrobe | 6 | 1 | 2 | 25 |
| 33 | Sadovska, 2024 | 2002-2019 | Latvia | Lineage 2, 41.3%; Lineage 4, 58.7% | 46 | NA | Ion Proton system | NA | PRJEB53131 | MGIT 960 and/or LJ | RIF | MGIT: 1 ug/mL LJ: 40 ug/mL | WHO_current | 18 | 28 | TBProfiler | 17 | 1 | 1 | 27 |
|  |  |  |  |  |  |  |  |  |  |  | INH | MGIT: 0.1 ug/mL LJ: 0.2 ug/mL | WHO_current | 23 | 23 |  | 23 | 1 | 0 | 22 |
|  |  |  |  |  |  |  |  |  |  |  | EMB | MGIT: 5 ug/mL LJ: 2 ug/mL | WHO_current | 16 | 30 |  | 15 | 2 | 1 | 28 |
|  |  |  |  |  |  |  |  |  |  |  | PZA | MGIT: 100 ug/mL | WHO_current | 7 | 9 |  | 6 | 2 | 1 | 7 |
|  |  |  |  |  |  |  |  |  |  |  | FLQ | OFX:  MGIT: 2 ug/mL LJ: 4 ug/mL LFX: MGIT: 1 ug/mL MFX: MGIT: 1 ug/mL | WHO_current | 5 | 13 |  | 5 | 0 | 0 | 13 |
|  |  |  |  |  |  |  |  |  |  |  | SM | MGIT: 1 ug/mL LJ: 4 ug/mL | WHO_current | 16 | 8 |  | 16 | 0 | 0 | 8 |
|  |  |  |  |  |  |  |  |  |  |  | AMK | MGIT: 1 ug/mL LJ: 30 ug/mL | WHO_current | 5 | 8 |  | 4 | 0 | 1 | 8 |
|  |  |  |  |  |  |  |  |  |  |  | ETO | MGIT: 5 ug/mL LJ: 40 ug/mL | WHO_current | 6 | 10 |  | 5 | 7 | 1 | 3 |
|  |  |  |  |  |  |  |  |  |  |  | PAS | MGIT: 4 ug/mL LJ: 0.5 ug/mL | WHO_past | 3 | 14 |  | 1 | 3 | 2 | 11 |
| Abbreviations: AMK, amikacin; AMG, aminoglycosides; BDQ, bedaquiline; CPM, capreomycin; CFX, ciprofloxacin; CFZ, clofazimine; DCS, D-cycloserine; CS, cycloserine; DLM, delamanid; EMB, ethambutol; ETO, ethionamide; FLQ, fluoroquinolones; GFX, gatifloxacin; INH, isoniazid; KM, kanamycin; LFX, levofloxacin; LZD, linezolid; MFX, moxifloxacin; OFX, ofloxacin; PAS, para-aminosalicylic acid; PZA, pyrazinamide; PTO, prothionamide; RMP, rifampicin; SM, streptomycin; WGS, Whole genome sequencing; DST, drug sensitivity testing; MycoTB, Sensititre MYCOTB MIC plate; LJ, Löwenstein-Jensen; 7H10/7H11, Middlebrook 7H10/7H11; MGIT 960, BACTEC™ MGIT 960 system; MDR-TB, multidrug-resistant Tuberculosis; XDR-TB, extensively drug-resistant Tuberculosis; WHO, World Health Organization; R, resistance; S, sensitive; TP, true-positive; FP, false-positive; TN, true-negative; FN, false-negative; NA, Not available. | | | | | | | | | | | | | | | | | | | | |
