# Supplementary material for: Diagnostic yield of nine user-friendly bioinformatics tools for predicting Mycobacterium tuberculosis drug resistance: A systematic review and network meta-analysis
Source: PLOS Glob Public Health. 2025 Apr 21;5(4):e0004465. doi: 10.1371/journal.pgph.0004465 (PMC12011222; doi:10.1371/journal.pgph.0004465)
Supplement: S2 Table — (DOCX) [file pgph.0004465.s011.docx]

| No. | Author, year | Size | MDR/XDR | Method | Drugs | Category | R | S | Tools | TP | FP | FN | TN |
| --- | --- | --- | --- | --- | --- | --- | --- | --- | --- | --- | --- | --- | --- |
| 1 | Phelan, 2016 | 10 | 10 | MGIT 960 | AMK | WHO_current | 7 | 3 | Mykrobe | 4 | 0 | 3 | 3 |
| 1 | Phelan, 2016 | 10 | 10 | MGIT 960 | AMK | WHO_current | 7 | 3 | TBProfiler | 7 | 0 | 0 | 3 |
| 1 | Phelan, 2016 | 10 | 10 | MGIT 960 | CPM | WHO_current | 6 | 4 | Mykrobe | 4 | 0 | 2 | 4 |
| 1 | Phelan, 2016 | 10 | 10 | MGIT 960 | CPM | WHO_current | 6 | 4 | TBProfiler | 6 | 0 | 0 | 4 |
| 1 | Phelan, 2016 | 10 | 10 | MGIT 960 | EMB | WHO_current | 9 | 1 | Mykrobe | 7 | 0 | 2 | 1 |
| 1 | Phelan, 2016 | 10 | 10 | MGIT 960 | EMB | WHO_current | 9 | 1 | TBProfiler | 9 | 0 | 0 | 1 |
| 1 | Phelan, 2016 | 10 | 10 | MGIT 960 | ETO | WHO_current | 10 | 0 | Mykrobe | 8 | 0 | 2 | 0 |
| 1 | Phelan, 2016 | 10 | 10 | MGIT 960 | ETO | WHO_current | 10 | 0 | TBProfiler | 10 | 0 | 0 | 0 |
| 1 | Phelan, 2016 | 10 | 10 | MGIT 960 | INH | WHO_current | 10 | 0 | Mykrobe | 9 | 0 | 1 | 0 |
| 1 | Phelan, 2016 | 10 | 10 | MGIT 960 | INH | WHO_current | 10 | 0 | TBProfiler | 10 | 0 | 0 | 0 |
| 1 | Phelan, 2016 | 10 | 10 | MGIT 960 | LZD | WHO_current | 0 | 10 | Mykrobe | 0 | 0 | 0 | 10 |
| 1 | Phelan, 2016 | 10 | 10 | MGIT 960 | LZD | WHO_current | 0 | 10 | TBProfiler | 0 | 0 | 0 | 10 |
| 1 | Phelan, 2016 | 10 | 10 | MGIT 960 | MFX | WHO_current | 7 | 3 | Mykrobe | 7 | 0 | 0 | 3 |
| 1 | Phelan, 2016 | 10 | 10 | MGIT 960 | MFX | WHO_current | 7 | 3 | TBProfiler | 7 | 0 | 0 | 3 |
| 1 | Phelan, 2016 | 10 | 10 | MGIT 960 | OFX | WHO_current | 7 | 3 | Mykrobe | 7 | 0 | 0 | 3 |
| 1 | Phelan, 2016 | 10 | 10 | MGIT 960 | OFX | WHO_current | 7 | 3 | TBProfiler | 7 | 0 | 0 | 3 |
| 1 | Phelan, 2016 | 10 | 10 | MGIT 960 | PAS | WHO_past | 2 | 8 | Mykrobe | 2 | 0 | 0 | 8 |
| 1 | Phelan, 2016 | 10 | 10 | MGIT 960 | PAS | WHO_past | 2 | 8 | TBProfiler | 0 | 0 | 2 | 8 |
| 1 | Phelan, 2016 | 10 | 10 | MGIT 960 | PZA | WHO_current | 9 | 1 | Mykrobe | 9 | 0 | 0 | 1 |
| 1 | Phelan, 2016 | 10 | 10 | MGIT 960 | PZA | WHO_current | 9 | 1 | TBProfiler | 7 | 0 | 2 | 1 |
| 1 | Phelan, 2016 | 10 | 10 | MGIT 960 | RFB | WHO_current | 10 | 0 | Mykrobe | 10 | 0 | 0 | 0 |
| 1 | Phelan, 2016 | 10 | 10 | MGIT 960 | RFB | WHO_current | 10 | 0 | TBProfiler | 10 | 0 | 0 | 0 |
| 1 | Phelan, 2016 | 10 | 10 | MGIT 960 | RMP | WHO_current | 10 | 0 | Mykrobe | 10 | 0 | 0 | 0 |
| 1 | Phelan, 2016 | 10 | 10 | MGIT 960 | RMP | WHO_current | 10 | 0 | TBProfiler | 10 | 0 | 0 | 0 |
| 1 | Phelan, 2016 | 10 | 10 | MGIT 960 | SM | WHO_current | 9 | 1 | Mykrobe | 5 | 0 | 4 | 1 |
| 1 | Phelan, 2016 | 10 | 10 | MGIT 960 | SM | WHO_current | 9 | 1 | TBProfiler | 9 | 0 | 0 | 1 |
| 2 | Schleusener, 2017 | 91 | NA | BACTEC 460 | EMB | WHO_past | 14 | 77 | CASTB | 8 | 0 | 6 | 77 |
| 2 | Schleusener, 2017 | 91 | NA | BACTEC 460 | EMB | WHO_past | 14 | 77 | KvarQ | 7 | 1 | 7 | 76 |
| 2 | Schleusener, 2017 | 91 | NA | BACTEC 460 | EMB | WHO_past | 14 | 77 | Mykrobe | 7 | 1 | 7 | 76 |
| 2 | Schleusener, 2017 | 91 | NA | BACTEC 460 | EMB | WHO_past | 14 | 77 | PhyResSE | 12 | 2 | 2 | 75 |
| 2 | Schleusener, 2017 | 91 | NA | BACTEC 460 | EMB | WHO_past | 14 | 77 | TBProfiler | 12 | 3 | 2 | 74 |
| 2 | Schleusener, 2017 | 91 | NA | BACTEC 460 | INH | WHO_past | 29 | 62 | CASTB | 24 | 1 | 5 | 61 |
| 2 | Schleusener, 2017 | 91 | NA | BACTEC 460 | INH | WHO_past | 29 | 62 | KvarQ | 23 | 1 | 6 | 61 |
| 2 | Schleusener, 2017 | 91 | NA | BACTEC 460 | INH | WHO_past | 29 | 62 | Mykrobe | 23 | 3 | 6 | 59 |
| 2 | Schleusener, 2017 | 91 | NA | BACTEC 460 | INH | WHO_past | 29 | 62 | PhyResSE | 27 | 2 | 2 | 60 |
| 2 | Schleusener, 2017 | 91 | NA | BACTEC 460 | INH | WHO_past | 29 | 62 | TBProfiler | 26 | 10 | 3 | 52 |
| 2 | Schleusener, 2017 | 91 | NA | BACTEC 460 | PZA | WHO_past | 9 | 82 | CASTB | 4 | 1 | 5 | 81 |
| 2 | Schleusener, 2017 | 91 | NA | BACTEC 460 | PZA | WHO_past | 9 | 82 | KvarQ | 2 | 3 | 7 | 79 |
| 2 | Schleusener, 2017 | 91 | NA | BACTEC 460 | PZA | WHO_past | 9 | 82 | Mykrobe | - | - | - | - |
| 2 | Schleusener, 2017 | 91 | NA | BACTEC 460 | PZA | WHO_past | 9 | 82 | PhyResSE | 6 | 3 | 3 | 79 |
| 2 | Schleusener, 2017 | 91 | NA | BACTEC 460 | PZA | WHO_past | 9 | 82 | TBProfiler | 4 | 3 | 5 | 79 |
| 2 | Schleusener, 2017 | 91 | NA | BACTEC 460 | RMP | WHO_past | 14 | 77 | CASTB | 14 | 3 | 0 | 74 |
| 2 | Schleusener, 2017 | 91 | NA | BACTEC 460 | RMP | WHO_past | 14 | 77 | KvarQ | 13 | 4 | 1 | 73 |
| 2 | Schleusener, 2017 | 91 | NA | BACTEC 460 | RMP | WHO_past | 14 | 77 | Mykrobe | 14 | 5 | 0 | 72 |
| 2 | Schleusener, 2017 | 91 | NA | BACTEC 460 | RMP | WHO_past | 14 | 77 | PhyResSE | 14 | 5 | 0 | 72 |
| 2 | Schleusener, 2017 | 91 | NA | BACTEC 460 | RMP | WHO_past | 14 | 77 | TBProfiler | 14 | 5 | 0 | 72 |
| 2 | Schleusener, 2017 | 91 | NA | BACTEC 460 | SM | WHO_past | 37 | 54 | CASTB | 11 | 0 | 26 | 54 |
| 2 | Schleusener, 2017 | 91 | NA | BACTEC 460 | SM | WHO_past | 37 | 54 | KvarQ | 21 | 0 | 16 | 54 |
| 2 | Schleusener, 2017 | 91 | NA | BACTEC 460 | SM | WHO_past | 37 | 54 | Mykrobe | 21 | 0 | 16 | 54 |
| 2 | Schleusener, 2017 | 91 | NA | BACTEC 460 | SM | WHO_past | 37 | 54 | PhyResSE | 31 | 1 | 6 | 53 |
| 2 | Schleusener, 2017 | 91 | NA | BACTEC 460 | SM | WHO_past | 37 | 54 | TBProfiler | 21 | 0 | 16 | 54 |
| 3 | Chatterjee, 2017 | 29 | 12 | MGIT 960 | EMB | WHO_past | 11 | 18 | Mykrobe | 6 | 0 | 5 | 18 |
| 3 | Chatterjee, 2017 | 29 | 12 | MGIT 960 | INH | WHO_current | 14 | 15 | Mykrobe | 13 | 0 | 1 | 15 |
| 3 | Chatterjee, 2017 | 29 | 12 | MGIT 960 | RMP | WHO_current | 13 | 16 | Mykrobe | 12 | 0 | 1 | 16 |
| 3 | Chatterjee, 2017 | 29 | 12 | MGIT 960 | SM | WHO_past | 11 | 18 | Mykrobe | 11 | 2 | 0 | 16 |
| 4 | Bouzouita, 2018 | 82 | 61 | MGIT 960 | PZA | WHO_current | 25 | 57 | PhyResSE | 23 | 0 | 2 | 57 |
| 5 | Macedo, 2018 | 54 | 54 | MGIT 960 | AMK | WHO_current | 11 | 43 | Mykrobe | 7 | 0 | 4 | 43 |
| 5 | Macedo, 2018 | 54 | 54 | MGIT 960 | AMK | WHO_current | 11 | 43 | PhyResSE | 7 | 0 | 4 | 43 |
| 5 | Macedo, 2018 | 54 | 54 | MGIT 960 | AMK | WHO_current | 11 | 43 | TBProfiler | 11 | 1 | 0 | 42 |
| 5 | Macedo, 2018 | 54 | 54 | MGIT 960 | AMK | WHO_current | 11 | 43 | TGS-TB | 7 | 1 | 4 | 42 |
| 5 | Macedo, 2018 | 54 | 54 | MGIT 960 | CPM | WHO_current | 7 | 47 | Mykrobe | 7 | 0 | 0 | 47 |
| 5 | Macedo, 2018 | 54 | 54 | MGIT 960 | CPM | WHO_current | 7 | 47 | PhyResSE | 7 | 0 | 0 | 47 |
| 5 | Macedo, 2018 | 54 | 54 | MGIT 960 | CPM | WHO_current | 7 | 47 | TBProfiler | 7 | 0 | 0 | 47 |
| 5 | Macedo, 2018 | 54 | 54 | MGIT 960 | CPM | WHO_current | 7 | 47 | TGS-TB | 7 | 0 | 0 | 47 |
| 5 | Macedo, 2018 | 54 | 54 | MGIT 960 | EMB | WHO_current | 32 | 22 | Mykrobe | 22 | 5 | 10 | 17 |
| 5 | Macedo, 2018 | 54 | 54 | MGIT 960 | EMB | WHO_current | 32 | 22 | PhyResSE | 30 | 7 | 2 | 15 |
| 5 | Macedo, 2018 | 54 | 54 | MGIT 960 | EMB | WHO_current | 32 | 22 | TBProfiler | 28 | 7 | 4 | 15 |
| 5 | Macedo, 2018 | 54 | 54 | MGIT 960 | EMB | WHO_current | 32 | 22 | TGS-TB | 28 | 6 | 4 | 16 |
| 5 | Macedo, 2018 | 54 | 54 | MGIT 960 | ETO | WHO_current | 34 | 20 | Mykrobe | 0 | 0 | 34 | 20 |
| 5 | Macedo, 2018 | 54 | 54 | MGIT 960 | ETO | WHO_current | 34 | 20 | PhyResSE | 27 | 0 | 7 | 20 |
| 5 | Macedo, 2018 | 54 | 54 | MGIT 960 | ETO | WHO_current | 34 | 20 | TBProfiler | 33 | 0 | 1 | 20 |
| 5 | Macedo, 2018 | 54 | 54 | MGIT 960 | ETO | WHO_current | 34 | 20 | TGS-TB | 33 | 0 | 1 | 20 |
| 5 | Macedo, 2018 | 54 | 54 | MGIT 960 | FLQ | WHO_undefined | 8 | 46 | Mykrobe | 8 | 0 | 0 | 46 |
| 5 | Macedo, 2018 | 54 | 54 | MGIT 960 | FLQ | WHO_undefined | 8 | 46 | PhyResSE | 8 | 0 | 0 | 46 |
| 5 | Macedo, 2018 | 54 | 54 | MGIT 960 | FLQ | WHO_undefined | 8 | 46 | TBProfiler | 8 | 0 | 0 | 46 |
| 5 | Macedo, 2018 | 54 | 54 | MGIT 960 | FLQ | WHO_undefined | 8 | 46 | TGS-TB | 8 | 0 | 0 | 46 |
| 5 | Macedo, 2018 | 54 | 54 | MGIT 960 | INH | WHO_current | 54 | 0 | Mykrobe | 52 | 0 | 2 | 0 |
| 5 | Macedo, 2018 | 54 | 54 | MGIT 960 | INH | WHO_current | 54 | 0 | PhyResSE | 52 | 0 | 2 | 0 |
| 5 | Macedo, 2018 | 54 | 54 | MGIT 960 | INH | WHO_current | 54 | 0 | TBProfiler | 54 | 0 | 0 | 0 |
| 5 | Macedo, 2018 | 54 | 54 | MGIT 960 | INH | WHO_current | 54 | 0 | TGS-TB | 53 | 0 | 1 | 0 |
| 5 | Macedo, 2018 | 54 | 54 | MGIT 960 | KM | WHO_current | 14 | 40 | Mykrobe | 7 | 0 | 7 | 40 |
| 5 | Macedo, 2018 | 54 | 54 | MGIT 960 | KM | WHO_current | 14 | 40 | PhyResSE | 12 | 0 | 2 | 40 |
| 5 | Macedo, 2018 | 54 | 54 | MGIT 960 | KM | WHO_current | 14 | 40 | TBProfiler | 14 | 0 | 0 | 40 |
| 5 | Macedo, 2018 | 54 | 54 | MGIT 960 | KM | WHO_current | 14 | 40 | TGS-TB | 14 | 0 | 0 | 40 |
| 5 | Macedo, 2018 | 54 | 54 | MGIT 960 | LZD | WHO_current | 0 | 54 | Mykrobe | 0 | 0 | 0 | 54 |
| 5 | Macedo, 2018 | 54 | 54 | MGIT 960 | LZD | WHO_current | 0 | 54 | PhyResSE | 0 | 0 | 0 | 54 |
| 5 | Macedo, 2018 | 54 | 54 | MGIT 960 | LZD | WHO_current | 0 | 54 | TBProfiler | 0 | 0 | 0 | 54 |
| 5 | Macedo, 2018 | 54 | 54 | MGIT 960 | LZD | WHO_current | 0 | 54 | TGS-TB | 0 | 0 | 0 | 54 |
| 5 | Macedo, 2018 | 54 | 54 | MGIT 960 | PAS | WHO_past | 1 | 53 | Mykrobe | 1 | 0 | 0 | 53 |
| 5 | Macedo, 2018 | 54 | 54 | MGIT 960 | PAS | WHO_past | 1 | 53 | PhyResSE | 1 | 0 | 0 | 53 |
| 5 | Macedo, 2018 | 54 | 54 | MGIT 960 | PAS | WHO_past | 1 | 53 | TBProfiler | 1 | 0 | 0 | 53 |
| 5 | Macedo, 2018 | 54 | 54 | MGIT 960 | PAS | WHO_past | 1 | 53 | TGS-TB | 1 | 0 | 0 | 53 |
| 5 | Macedo, 2018 | 54 | 54 | MGIT 960 | PZA | WHO_current | 31 | 23 | Mykrobe | 0 | 0 | 31 | 23 |
| 5 | Macedo, 2018 | 54 | 54 | MGIT 960 | PZA | WHO_current | 31 | 23 | PhyResSE | 20 | 2 | 11 | 21 |
| 5 | Macedo, 2018 | 54 | 54 | MGIT 960 | PZA | WHO_current | 31 | 23 | TBProfiler | 22 | 2 | 9 | 21 |
| 5 | Macedo, 2018 | 54 | 54 | MGIT 960 | PZA | WHO_current | 31 | 23 | TGS-TB | 29 | 2 | 2 | 21 |
| 5 | Macedo, 2018 | 54 | 54 | MGIT 960 | RMP | WHO_current | 54 | 0 | Mykrobe | 54 | 0 | 0 | 0 |
| 5 | Macedo, 2018 | 54 | 54 | MGIT 960 | RMP | WHO_current | 54 | 0 | PhyResSE | 54 | 0 | 0 | 0 |
| 5 | Macedo, 2018 | 54 | 54 | MGIT 960 | RMP | WHO_current | 54 | 0 | TBProfiler | 53 | 0 | 1 | 0 |
| 5 | Macedo, 2018 | 54 | 54 | MGIT 960 | RMP | WHO_current | 54 | 0 | TGS-TB | 52 | 0 | 2 | 0 |
| 5 | Macedo, 2018 | 54 | 54 | MGIT 960 | SM | WHO_current | 46 | 8 | Mykrobe | 31 | 0 | 15 | 8 |
| 5 | Macedo, 2018 | 54 | 54 | MGIT 960 | SM | WHO_current | 46 | 8 | PhyResSE | 33 | 0 | 13 | 8 |
| 5 | Macedo, 2018 | 54 | 54 | MGIT 960 | SM | WHO_current | 46 | 8 | TBProfiler | 45 | 4 | 1 | 4 |
| 5 | Macedo, 2018 | 54 | 54 | MGIT 960 | SM | WHO_current | 46 | 8 | TGS-TB | 31 | 1 | 9 | 7 |
| 6 | Feliciano, 2018 | 29 | NA | MGIT 960 | EMB | WHO_current | 7 | 22 | TBProfiler | 7 | 4 | 0 | 18 |
| 6 | Feliciano, 2018 | 29 | NA | MGIT 960 | INH | WHO_current | 23 | 6 | TBProfiler | 21 | 0 | 2 | 6 |
| 6 | Feliciano, 2018 | 29 | NA | MGIT 960 | RMP | WHO_current | 16 | 13 | TBProfiler | 14 | 1 | 2 | 12 |
| 6 | Feliciano, 2018 | 29 | NA | MGIT 960 | SM | WHO_current | 14 | 15 | TBProfiler | 12 | 1 | 2 | 14 |
| 7 | Faksri, 2019 | 266 | 207 | 7H10 | AMK | WHO_undefined | 29 | 175 | PhyResSE | 20 | 9 | 0 | 175 |
| 7 | Faksri, 2019 | 266 | 207 | 7H10 | AMK | WHO_undefined | 29 | 175 | TBProfiler | 20 | 9 | 4 | 171 |
| 7 | Faksri, 2019 | 266 | 207 | 7H10 | EMB | WHO_current | 121 | 108 | PhyResSE | 106 | 15 | 20 | 88 |
| 7 | Faksri, 2019 | 266 | 207 | 7H10 | EMB | WHO_current | 121 | 108 | TBProfiler | 112 | 9 | 20 | 88 |
| 7 | Faksri, 2019 | 266 | 207 | 7H10 | ETO | WHO_current | 49 | 155 | PhyResSE | 0 | 49 | 1 | 154 |
| 7 | Faksri, 2019 | 266 | 207 | 7H10 | ETO | WHO_current | 49 | 155 | TBProfiler | 19 | 30 | 8 | 147 |
| 7 | Faksri, 2019 | 266 | 207 | 7H10 | GFX | WHO_past | 20 | 184 | PhyResSE | 19 | 1 | 86 | 98 |
| 7 | Faksri, 2019 | 266 | 207 | 7H10 | GFX | WHO_past | 20 | 184 | TBProfiler | 19 | 1 | 89 | 95 |
| 7 | Faksri, 2019 | 266 | 207 | 7H10 | INH | WHO_current | 204 | 57 | PhyResSE | 194 | 10 | 0 | 57 |
| 7 | Faksri, 2019 | 266 | 207 | 7H10 | INH | WHO_current | 204 | 57 | TBProfiler | 197 | 7 | 8 | 49 |
| 7 | Faksri, 2019 | 266 | 207 | 7H10 | KM | WHO_past | 34 | 170 | PhyResSE | 1 | 33 | 6 | 164 |
| 7 | Faksri, 2019 | 266 | 207 | 7H10 | KM | WHO_past | 34 | 170 | TBProfiler | 22 | 12 | 7 | 163 |
| 7 | Faksri, 2019 | 266 | 207 | 7H10 | LFX | WHO_current | 66 | 135 | PhyResSE | 60 | 6 | 41 | 94 |
| 7 | Faksri, 2019 | 266 | 207 | 7H10 | LFX | WHO_current | 66 | 135 | TBProfiler | 60 | 6 | 45 | 90 |
| 7 | Faksri, 2019 | 266 | 207 | 7H10 | MFX | WHO_past | 60 | 141 | PhyResSE | 50 | 10 | 51 | 90 |
| 7 | Faksri, 2019 | 266 | 207 | 7H10 | MFX | WHO_past | 60 | 141 | TBProfiler | 55 | 5 | 50 | 91 |
| 7 | Faksri, 2019 | 266 | 207 | 7H10 | OFX | WHO_current | 111 | 96 | PhyResSE | 99 | 12 | 5 | 91 |
| 7 | Faksri, 2019 | 266 | 207 | 7H10 | OFX | WHO_current | 111 | 96 | TBProfiler | 98 | 13 | 10 | 86 |
| 7 | Faksri, 2019 | 266 | 207 | 7H10 | RMP | WHO_current | 202 | 60 | PhyResSE | 183 | 19 | 0 | 60 |
| 7 | Faksri, 2019 | 266 | 207 | 7H10 | RMP | WHO_current | 202 | 60 | TBProfiler | 185 | 17 | 0 | 60 |
| 7 | Faksri, 2019 | 266 | 207 | 7H10 | SM | WHO_current | 130 | 132 | PhyResSE | 116 | 14 | 7 | 125 |
| 7 | Faksri, 2019 | 266 | 207 | 7H10 | SM | WHO_current | 130 | 132 | TBProfiler | 124 | 6 | 13 | 119 |
| 8 | Beek, 2019 | 211 | 8 | MGIT 960 | EMB | WHO_current | 1 | 210 | KvarQ | 0 | 3 | 1 | 206 |
| 8 | Beek, 2019 | 211 | 8 | MGIT 960 | EMB | WHO_current | 1 | 210 | Mykrobe | 0 | 3 | 1 | 207 |
| 8 | Beek, 2019 | 211 | 8 | MGIT 960 | EMB | WHO_current | 1 | 210 | PhyResSE | 0 | 5 | 1 | 205 |
| 8 | Beek, 2019 | 211 | 8 | MGIT 960 | EMB | WHO_current | 1 | 210 | TBProfiler | 1 | 7 | 0 | 202 |
| 8 | Beek, 2019 | 211 | 8 | MGIT 960 | EMB | WHO_current | 1 | 210 | TGS-TB | 1 | 6 | 0 | 203 |
| 8 | Beek, 2019 | 211 | 8 | MGIT 960 | INH | WHO_current | 16 | 195 | KvarQ | 12 | 0 | 4 | 195 |
| 8 | Beek, 2019 | 211 | 8 | MGIT 960 | INH | WHO_current | 16 | 195 | Mykrobe | 12 | 0 | 4 | 195 |
| 8 | Beek, 2019 | 211 | 8 | MGIT 960 | INH | WHO_current | 16 | 195 | PhyResSE | 13 | 0 | 3 | 195 |
| 8 | Beek, 2019 | 211 | 8 | MGIT 960 | INH | WHO_current | 16 | 195 | TBProfiler | 13 | 7 | 3 | 188 |
| 8 | Beek, 2019 | 211 | 8 | MGIT 960 | INH | WHO_current | 16 | 195 | TGS-TB | 12 | 30 | 4 | 165 |
| 8 | Beek, 2019 | 211 | 8 | MGIT 960 | PZA | WHO_current | 5 | 206 | KvarQ | 3 | 2 | 1 | 204 |
| 8 | Beek, 2019 | 211 | 8 | MGIT 960 | PZA | WHO_current | 5 | 206 | Mykrobe | - | - | - | - |
| 8 | Beek, 2019 | 211 | 8 | MGIT 960 | PZA | WHO_current | 5 | 206 | PhyResSE | 2 | 1 | 3 | 205 |
| 8 | Beek, 2019 | 211 | 8 | MGIT 960 | PZA | WHO_current | 5 | 206 | TBProfiler | 3 | 0 | 1 | 206 |
| 8 | Beek, 2019 | 211 | 8 | MGIT 960 | PZA | WHO_current | 5 | 206 | TGS-TB | 3 | 0 | 1 | 206 |
| 8 | Beek, 2019 | 211 | 8 | MGIT 960 | RMP | WHO_current | 8 | 203 | KvarQ | 8 | 0 | 0 | 203 |
| 8 | Beek, 2019 | 211 | 8 | MGIT 960 | RMP | WHO_current | 8 | 203 | Mykrobe | 8 | 4 | 0 | 199 |
| 8 | Beek, 2019 | 211 | 8 | MGIT 960 | RMP | WHO_current | 8 | 203 | PhyResSE | 8 | 0 | 0 | 203 |
| 8 | Beek, 2019 | 211 | 8 | MGIT 960 | RMP | WHO_current | 8 | 203 | TBProfiler | 8 | 0 | 0 | 203 |
| 8 | Beek, 2019 | 211 | 8 | MGIT 960 | RMP | WHO_current | 8 | 203 | TGS-TB | 8 | 0 | 0 | 203 |
| 8 | Beek, 2019 | 211 | 8 | MGIT 960 | SM | WHO_current | 12 | 199 | KvarQ | 11 | 0 | 1 | 199 |
| 8 | Beek, 2019 | 211 | 8 | MGIT 960 | SM | WHO_current | 12 | 199 | Mykrobe | 11 | 0 | 1 | 199 |
| 8 | Beek, 2019 | 211 | 8 | MGIT 960 | SM | WHO_current | 12 | 199 | PhyResSE | 11 | 0 | 1 | 199 |
| 8 | Beek, 2019 | 211 | 8 | MGIT 960 | SM | WHO_current | 12 | 199 | TBProfiler | 11 | 0 | 1 | 199 |
| 8 | Beek, 2019 | 211 | 8 | MGIT 960 | SM | WHO_current | 12 | 199 | TGS-TB | 11 | 8 | 1 | 191 |
| 9 | Iwamoto, 2019 | 191 | 165 | MGIT 960 | PZA | WHO_current | 108 | 83 | CASTB | 34 | 1 | 74 | 82 |
| 9 | Iwamoto, 2019 | 191 | 165 | MGIT 960 | PZA | WHO_current | 108 | 83 | PhyResSE | 53 | 1 | 55 | 82 |
| 9 | Iwamoto, 2019 | 191 | 165 | MGIT 960 | PZA | WHO_current | 108 | 83 | TBProfiler | 65 | 0 | 43 | 83 |
| 9 | Iwamoto, 2019 | 191 | 165 | MGIT 960 | PZA | WHO_current | 108 | 83 | TGS-TB | 105 | 1 | 3 | 82 |
| 10 | Guimarães, 2021 | 71 | 16 | LJ | EMB | WHO_current | 8 | 63 | CASTB | 5 | 5 | 3 | 58 |
| 10 | Guimarães, 2021 | 71 | 16 | LJ | EMB | WHO_current | 8 | 63 | KvarQ | 7 | 2 | 1 | 61 |
| 10 | Guimarães, 2021 | 71 | 16 | LJ | EMB | WHO_current | 8 | 63 | MTBseq | 7 | 5 | 1 | 58 |
| 10 | Guimarães, 2021 | 71 | 16 | LJ | EMB | WHO_current | 8 | 63 | Mykrobe | 7 | 7 | 1 | 56 |
| 10 | Guimarães, 2021 | 71 | 16 | LJ | EMB | WHO_current | 8 | 63 | PhyResSE | 8 | 4 | 0 | 59 |
| 10 | Guimarães, 2021 | 71 | 16 | LJ | EMB | WHO_current | 8 | 63 | TBProfiler | 8 | 6 | 0 | 57 |
| 10 | Guimarães, 2021 | 71 | 16 | LJ | INH | WHO_current | 30 | 41 | CASTB | 25 | 3 | 5 | 38 |
| 10 | Guimarães, 2021 | 71 | 16 | LJ | INH | WHO_current | 30 | 41 | KvarQ | 26 | 1 | 4 | 40 |
| 10 | Guimarães, 2021 | 71 | 16 | LJ | INH | WHO_current | 30 | 41 | MTBseq | 28 | 10 | 2 | 31 |
| 10 | Guimarães, 2021 | 71 | 16 | LJ | INH | WHO_current | 30 | 41 | Mykrobe | 27 | 2 | 3 | 39 |
| 10 | Guimarães, 2021 | 71 | 16 | LJ | INH | WHO_current | 30 | 41 | PhyResSE | 20 | 0 | 10 | 41 |
| 10 | Guimarães, 2021 | 71 | 16 | LJ | INH | WHO_current | 30 | 41 | TBProfiler | 26 | 1 | 4 | 40 |
| 10 | Guimarães, 2021 | 71 | 16 | LJ | PZA | WHO_past | 4 | 65 | CASTB | 2 | 4 | 2 | 61 |
| 10 | Guimarães, 2021 | 71 | 16 | LJ | PZA | WHO_past | 4 | 65 | KvarQ | 1 | 4 | 5 | 61 |
| 10 | Guimarães, 2021 | 71 | 16 | LJ | PZA | WHO_past | 4 | 65 | MTBseq | 2 | 5 | 4 | 60 |
| 10 | Guimarães, 2021 | 71 | 16 | LJ | PZA | WHO_past | 4 | 65 | Mykrobe | 4 | 5 | 2 | 60 |
| 10 | Guimarães, 2021 | 71 | 16 | LJ | PZA | WHO_past | 4 | 65 | PhyResSE | 2 | 5 | 4 | 60 |
| 10 | Guimarães, 2021 | 71 | 16 | LJ | PZA | WHO_past | 4 | 65 | TBProfiler | 3 | 2 | 3 | 63 |
| 10 | Guimarães, 2021 | 71 | 16 | LJ | RMP | WHO_current | 18 | 53 | CASTB | 13 | 4 | 5 | 49 |
| 10 | Guimarães, 2021 | 71 | 16 | LJ | RMP | WHO_current | 18 | 53 | KvarQ | 17 | 5 | 1 | 48 |
| 10 | Guimarães, 2021 | 71 | 16 | LJ | RMP | WHO_current | 18 | 53 | MTBseq | 15 | 5 | 3 | 48 |
| 10 | Guimarães, 2021 | 71 | 16 | LJ | RMP | WHO_current | 18 | 53 | Mykrobe | 17 | 6 | 1 | 47 |
| 10 | Guimarães, 2021 | 71 | 16 | LJ | RMP | WHO_current | 18 | 53 | PhyResSE | 1 | 0 | 17 | 53 |
| 10 | Guimarães, 2021 | 71 | 16 | LJ | RMP | WHO_current | 18 | 53 | TBProfiler | 17 | 5 | 1 | 48 |
| 10 | Guimarães, 2021 | 71 | 16 | LJ | SM | WHO_current | 8 | 63 | CASTB | 5 | 36 | 3 | 27 |
| 10 | Guimarães, 2021 | 71 | 16 | LJ | SM | WHO_current | 8 | 63 | KvarQ | 6 | 1 | 2 | 62 |
| 10 | Guimarães, 2021 | 71 | 16 | LJ | SM | WHO_current | 8 | 63 | MTBseq | 6 | 2 | 2 | 61 |
| 10 | Guimarães, 2021 | 71 | 16 | LJ | SM | WHO_current | 8 | 63 | Mykrobe | 6 | 1 | 2 | 62 |
| 10 | Guimarães, 2021 | 71 | 16 | LJ | SM | WHO_current | 8 | 63 | PhyResSE | 5 | 2 | 3 | 61 |
| 10 | Guimarães, 2021 | 71 | 16 | LJ | SM | WHO_current | 8 | 63 | TBProfiler | 6 | 1 | 2 | 62 |
| 11.1 | Nonghanphithak, 2020 | 60 | 59 | 7H10 | AMK | WHO_undefined | 23 | 37 | TBProfiler | 17 | 0 | 6 | 37 |
| 11.1 | Nonghanphithak, 2020 | 60 | 59 | 7H10 | EMB | WHO_current | 38 | 19 | TBProfiler | 35 | 4 | 3 | 15 |
| 11.1 | Nonghanphithak, 2020 | 60 | 59 | 7H10 | ETO | WHO_current | 25 | 35 | TBProfiler | 23 | 6 | 2 | 29 |
| 11.1 | Nonghanphithak, 2020 | 60 | 59 | 7H10 | INH | WHO_current | 59 | 1 | TBProfiler | 56 | 0 | 3 | 1 |
| 11.1 | Nonghanphithak, 2020 | 60 | 59 | 7H10 | KM | WHO_past | 27 | 32 | TBProfiler | 19 | 0 | 8 | 32 |
| 11.1 | Nonghanphithak, 2020 | 60 | 59 | 7H10 | MFX | WHO_past | 17 | 41 | TBProfiler | 15 | 11 | 2 | 30 |
| 11.1 | Nonghanphithak, 2020 | 60 | 59 | 7H10 | OFX | WHO_current | 31 | 29 | TBProfiler | 28 | 0 | 3 | 29 |
| 11.1 | Nonghanphithak, 2020 | 60 | 59 | 7H10 | PAS | WHO_past | 31 | 29 | TBProfiler | 22 | 1 | 9 | 28 |
| 11.1 | Nonghanphithak, 2020 | 60 | 59 | 7H10 | RMP | WHO_current | 60 | 0 | TBProfiler | 57 | 0 | 3 | 0 |
| 11.1 | Nonghanphithak, 2020 | 60 | 59 | 7H10 | SM | WHO_current | 36 | 24 | TBProfiler | 34 | 9 | 2 | 15 |
| 12 | Wu, 2020 | 306 | 254 | MGIT 960 | AMK | WHO_current | 36 | 270 | TBProfiler | 27 | 0 | 9 | 270 |
| 12 | Wu, 2020 | 306 | 254 | MGIT 960 | EMB | WHO_current | 118 | 188 | TBProfiler | 115 | 67 | 3 | 121 |
| 12 | Wu, 2020 | 306 | 254 | MGIT 960 | INH | WHO_current | 256 | 50 | TBProfiler | 242 | 4 | 14 | 46 |
| 12 | Wu, 2020 | 306 | 254 | MGIT 960 | OFX | WHO_current | 129 | 177 | TBProfiler | 120 | 2 | 9 | 175 |
| 12 | Wu, 2020 | 306 | 254 | MGIT 960 | RMP | WHO_current | 276 | 30 | TBProfiler | 268 | 0 | 8 | 30 |
| 12 | Wu, 2020 | 306 | 254 | MGIT 960 | SM | WHO_current | 210 | 96 | TBProfiler | 204 | 4 | 6 | 92 |
| 13 | Genestet, 2020 | 274 | 4 | MGIT 960 | EMB | WHO_current | 1 | 273 | PhyResSE | 1 | 4 | 0 | 269 |
| 13 | Genestet, 2020 | 274 | 4 | MGIT 960 | INH | WHO_current | 21 | 253 | PhyResSE | 21 | 0 | 0 | 253 |
| 13 | Genestet, 2020 | 274 | 4 | MGIT 960 | PZA | WHO_current | 16 | 258 | PhyResSE | 15 | 0 | 1 | 258 |
| 13 | Genestet, 2020 | 274 | 4 | MGIT 960 | RMP | WHO_current | 6 | 268 | PhyResSE | 6 | 1 | 0 | 267 |
| 14 | Kim, 2022 | 37 | 14 | LJ | AMG (AMK, KM, CPM) | WHO_current | 1 | 36 | CASTB | 1 | 0 | 0 | 36 |
| 14 | Kim, 2022 | 37 | 14 | LJ | AMG (AMK, KM, CPM) | WHO_current | 1 | 36 | Mykrobe | 1 | 0 | 0 | 36 |
| 14 | Kim, 2022 | 37 | 14 | LJ | AMG (AMK, KM, CPM) | WHO_current | 1 | 36 | PhyResSE | 1 | 0 | 0 | 36 |
| 14 | Kim, 2022 | 37 | 14 | LJ | AMG (AMK, KM, CPM) | WHO_current | 1 | 36 | TBProfiler | 1 | 0 | 0 | 36 |
| 14 | Kim, 2022 | 37 | 14 | LJ | AMG (AMK, KM, CPM) | WHO_current | 1 | 36 | TGS-TB | 1 | 0 | 0 | 36 |
| 14 | Kim, 2022 | 37 | 14 | LJ | CS | WHO_past | 0 | 37 | CASTB | 0 | 0 | 0 | 37 |
| 14 | Kim, 2022 | 37 | 14 | LJ | CS | WHO_past | 0 | 37 | Mykrobe | 0 | 0 | 0 | 37 |
| 14 | Kim, 2022 | 37 | 14 | LJ | CS | WHO_past | 0 | 37 | PhyResSE | 0 | 0 | 0 | 37 |
| 14 | Kim, 2022 | 37 | 14 | LJ | CS | WHO_past | 0 | 37 | TBProfiler | 0 | 0 | 0 | 37 |
| 14 | Kim, 2022 | 37 | 14 | LJ | CS | WHO_past | 0 | 37 | TGS-TB | 0 | 0 | 0 | 37 |
| 14 | Kim, 2022 | 37 | 14 | LJ | EMB | WHO_current | 4 | 33 | CASTB | 3 | 5 | 1 | 28 |
| 14 | Kim, 2022 | 37 | 14 | LJ | EMB | WHO_current | 4 | 33 | Mykrobe | 3 | 4 | 1 | 29 |
| 14 | Kim, 2022 | 37 | 14 | LJ | EMB | WHO_current | 4 | 33 | PhyResSE | 3 | 4 | 1 | 29 |
| 14 | Kim, 2022 | 37 | 14 | LJ | EMB | WHO_current | 4 | 33 | TBProfiler | 3 | 5 | 1 | 28 |
| 14 | Kim, 2022 | 37 | 14 | LJ | EMB | WHO_current | 4 | 33 | TGS-TB | 3 | 6 | 1 | 27 |
| 14 | Kim, 2022 | 37 | 14 | LJ | ETO | WHO_current | 3 | 34 | CASTB | 0 | 0 | 3 | 34 |
| 14 | Kim, 2022 | 37 | 14 | LJ | ETO | WHO_current | 3 | 34 | Mykrobe | 0 | 0 | 3 | 34 |
| 14 | Kim, 2022 | 37 | 14 | LJ | ETO | WHO_current | 3 | 34 | PhyResSE | 2 | 9 | 1 | 25 |
| 14 | Kim, 2022 | 37 | 14 | LJ | ETO | WHO_current | 3 | 34 | TBProfiler | 2 | 13 | 1 | 21 |
| 14 | Kim, 2022 | 37 | 14 | LJ | ETO | WHO_current | 3 | 34 | TGS-TB | 2 | 14 | 1 | 20 |
| 14 | Kim, 2022 | 37 | 14 | LJ | FLQ (LFX, MFX, OFX) | WHO_past | 5 | 32 | CASTB | 2 | 0 | 3 | 32 |
| 14 | Kim, 2022 | 37 | 14 | LJ | FLQ (LFX, MFX, OFX) | WHO_past | 5 | 32 | Mykrobe | 3 | 0 | 2 | 32 |
| 14 | Kim, 2022 | 37 | 14 | LJ | FLQ (LFX, MFX, OFX) | WHO_past | 5 | 32 | PhyResSE | 3 | 0 | 2 | 32 |
| 14 | Kim, 2022 | 37 | 14 | LJ | FLQ (LFX, MFX, OFX) | WHO_past | 5 | 32 | TBProfiler | 3 | 1 | 2 | 31 |
| 14 | Kim, 2022 | 37 | 14 | LJ | FLQ (LFX, MFX, OFX) | WHO_past | 5 | 32 | TGS-TB | 4 | 2 | 1 | 30 |
| 14 | Kim, 2022 | 37 | 14 | LJ | INH | WHO_current | 17 | 20 | CASTB | 11 | 13 | 6 | 7 |
| 14 | Kim, 2022 | 37 | 14 | LJ | INH | WHO_current | 17 | 20 | Mykrobe | 11 | 15 | 6 | 5 |
| 14 | Kim, 2022 | 37 | 14 | LJ | INH | WHO_current | 17 | 20 | PhyResSE | 11 | 15 | 6 | 5 |
| 14 | Kim, 2022 | 37 | 14 | LJ | INH | WHO_current | 17 | 20 | TBProfiler | 12 | 15 | 5 | 5 |
| 14 | Kim, 2022 | 37 | 14 | LJ | INH | WHO_current | 17 | 20 | TGS-TB | 12 | 15 | 5 | 5 |
| 14 | Kim, 2022 | 37 | 14 | LJ | PAS | WHO_past | 0 | 37 | CASTB | 0 | 0 | 0 | 37 |
| 14 | Kim, 2022 | 37 | 14 | LJ | PAS | WHO_past | 0 | 37 | Mykrobe | 0 | 0 | 0 | 37 |
| 14 | Kim, 2022 | 37 | 14 | LJ | PAS | WHO_past | 0 | 37 | PhyResSE | 0 | 0 | 0 | 37 |
| 14 | Kim, 2022 | 37 | 14 | LJ | PAS | WHO_past | 0 | 37 | TBProfiler | 0 | 2 | 0 | 35 |
| 14 | Kim, 2022 | 37 | 14 | LJ | PAS | WHO_past | 0 | 37 | TGS-TB | 0 | 0 | 0 | 37 |
| 14 | Kim, 2022 | 37 | 14 | Pyrazinamidase test | PZA | WHO_past | 0 | 37 | CASTB | 0 | 4 | 0 | 33 |
| 14 | Kim, 2022 | 37 | 14 | Pyrazinamidase test | PZA | WHO_past | 0 | 37 | Mykrobe | 0 | 5 | 0 | 32 |
| 14 | Kim, 2022 | 37 | 14 | Pyrazinamidase test | PZA | WHO_past | 0 | 37 | PhyResSE | 0 | 11 | 0 | 26 |
| 14 | Kim, 2022 | 37 | 14 | Pyrazinamidase test | PZA | WHO_past | 0 | 37 | TBProfiler | 0 | 6 | 0 | 31 |
| 14 | Kim, 2022 | 37 | 14 | Pyrazinamidase test | PZA | WHO_past | 0 | 37 | TGS-TB | 0 | 3 | 0 | 34 |
| 14 | Kim, 2022 | 37 | 14 | LJ | RMP | WHO_current | 10 | 27 | CASTB | 9 | 5 | 1 | 22 |
| 14 | Kim, 2022 | 37 | 14 | LJ | RMP | WHO_current | 10 | 27 | Mykrobe | 8 | 2 | 2 | 25 |
| 14 | Kim, 2022 | 37 | 14 | LJ | RMP | WHO_current | 10 | 27 | PhyResSE | 8 | 5 | 2 | 22 |
| 14 | Kim, 2022 | 37 | 14 | LJ | RMP | WHO_current | 10 | 27 | TBProfiler | 8 | 5 | 2 | 22 |
| 14 | Kim, 2022 | 37 | 14 | LJ | RMP | WHO_current | 10 | 27 | TGS-TB | 8 | 5 | 2 | 22 |
| 14 | Kim, 2022 | 37 | 14 | LJ | SM | WHO_past | 11 | 26 | CASTB | 7 | 0 | 4 | 26 |
| 14 | Kim, 2022 | 37 | 14 | LJ | SM | WHO_past | 11 | 26 | Mykrobe | 8 | 0 | 3 | 26 |
| 14 | Kim, 2022 | 37 | 14 | LJ | SM | WHO_past | 11 | 26 | PhyResSE | 8 | 0 | 3 | 26 |
| 14 | Kim, 2022 | 37 | 14 | LJ | SM | WHO_past | 11 | 26 | TBProfiler | 8 | 1 | 3 | 25 |
| 14 | Kim, 2022 | 37 | 14 | LJ | SM | WHO_past | 11 | 26 | TGS-TB | 8 | 0 | 3 | 26 |
| 15 | Che, 2022 | 59 | 49 | LJ | AMK | WHO_current | 8 | 51 | TBProfiler | 6 | 0 | 2 | 51 |
| 15 | Che, 2022 | 59 | 49 | LJ | CPM | WHO_current | 5 | 54 | TBProfiler | 4 | 2 | 1 | 52 |
| 15 | Che, 2022 | 59 | 49 | LJ | EMB | WHO_current | 20 | 39 | TBProfiler | 20 | 14 | 0 | 25 |
| 15 | Che, 2022 | 59 | 49 | LJ | INH | WHO_current | 49 | 10 | TBProfiler | 47 | 1 | 2 | 9 |
| 15 | Che, 2022 | 59 | 49 | LJ | LFX | WHO_past | 23 | 36 | TBProfiler | 23 | 1 | 0 | 35 |
| 15 | Che, 2022 | 59 | 49 | LJ | PAS | WHO_past | 10 | 39 | TBProfiler | 7 | 1 | 3 | 38 |
| 15 | Che, 2022 | 59 | 49 | LJ | PTO | WHO_current | 15 | 44 | TBProfiler | 14 | 4 | 1 | 40 |
| 15 | Che, 2022 | 59 | 49 | LJ | RMP | WHO_current | 59 | 0 | TBProfiler | 59 | 0 | 0 | 0 |
| 15 | Che, 2022 | 59 | 49 | LJ | SM | WHO_current | 38 | 21 | TBProfiler | 37 | 0 | 1 | 21 |
| 16 | Wu, 2022 | 182 | 59 | MycoTB | AMK | WHO_undefined | 6 | 176 | TBProfiler | 6 | 57 | 0 | 119 |
| 16 | Wu, 2022 | 182 | 59 | MycoTB | CS | WHO_undefined | 28 | 154 | TBProfiler | 0 | 0 | 28 | 154 |
| 16 | Wu, 2022 | 182 | 59 | MycoTB | EMB | WHO_undefined | 33 | 149 | TBProfiler | 33 | 19 | 0 | 130 |
| 16 | Wu, 2022 | 182 | 59 | MycoTB | ETO | WHO_undefined | 8 | 174 | TBProfiler | 5 | 20 | 3 | 154 |
| 16 | Wu, 2022 | 182 | 59 | MycoTB | INH | WHO_undefined | 78 | 104 | TBProfiler | 71 | 5 | 7 | 99 |
| 16 | Wu, 2022 | 182 | 59 | MycoTB | KM | WHO_undefined | 8 | 174 | TBProfiler | 8 | 57 | 0 | 117 |
| 16 | Wu, 2022 | 182 | 59 | MycoTB | MFX | WHO_undefined | 38 | 144 | TBProfiler | 33 | 6 | 5 | 138 |
| 16 | Wu, 2022 | 182 | 59 | MycoTB | OFX | WHO_undefined | 37 | 145 | TBProfiler | 32 | 7 | 5 | 138 |
| 16 | Wu, 2022 | 182 | 59 | MycoTB | PAS | WHO_undefined | 6 | 177 | TBProfiler | 2 | 3 | 4 | 174 |
| 16 | Wu, 2022 | 182 | 59 | MycoTB | RMP | WHO_undefined | 68 | 114 | TBProfiler | 66 | 11 | 2 | 103 |
| 16 | Wu, 2022 | 182 | 59 | MycoTB | SM | WHO_undefined | 59 | 123 | TBProfiler | 57 | 47 | 2 | 76 |
| 17.1 | Finci, 2022 | 677 | 605 | MGIT 960 | AMK | WHO_current | 36 | 636 | MTBseq | 34 | 10 | 2 | 626 |
| 17.1 | Finci, 2022 | 677 | 605 | MGIT 960 | INH | WHO_current | 635 | 39 | MTBseq | 626 | 3 | 9 | 36 |
| 17.1 | Finci, 2022 | 677 | 605 | MGIT 960 | KM | WHO_current | 186 | 486 | MTBseq | 176 | 23 | 10 | 463 |
| 17.1 | Finci, 2022 | 677 | 605 | MGIT 960 | LFX | WHO_past | 42 | 613 | MTBseq | 41 | 9 | 1 | 604 |
| 17.1 | Finci, 2022 | 677 | 605 | MGIT 960 | MFX | WHO_past | 70 | 604 | MTBseq | 59 | 7 | 11 | 597 |
| 17.1 | Finci, 2022 | 677 | 605 | MGIT 960 | RMP | WHO_current | 614 | 57 | MTBseq | 610 | 36 | 4 | 21 |
| 18.1 | Hall, 2023 | 132 | NA | LJ or 7H | AMK | WHO_current | 11 | 78 | Mykrobe | 10 | 2 | 1 | 76 |
| 18.1 | Hall, 2023 | 132 | NA | LJ or 7H | CPM | WHO_current | 1 | 51 | Mykrobe | 0 | 1 | 1 | 50 |
| 18.1 | Hall, 2023 | 132 | NA | LJ or 7H | EMB | WHO_current | 14 | 77 | Mykrobe | 10 | 14 | 4 | 63 |
| 18.1 | Hall, 2023 | 132 | NA | LJ or 7H | INH | WHO_current | 51 | 48 | Mykrobe | 42 | 3 | 9 | 45 |
| 18.1 | Hall, 2023 | 132 | NA | LJ or 7H | KM | WHO_current | 0 | 52 | Mykrobe | 0 | 1 | 0 | 51 |
| 18.1 | Hall, 2023 | 132 | NA | LJ or 7H | OFX | WHO_current | 10 | 77 | Mykrobe | 10 | 4 | 0 | 73 |
| 18.1 | Hall, 2023 | 132 | NA | LJ or 7H | RMP | WHO_current | 48 | 44 | Mykrobe | 42 | 1 | 6 | 43 |
| 18.1 | Hall, 2023 | 132 | NA | LJ or 7H | SM | WHO_current | 8 | 83 | Mykrobe | 4 | 11 | 4 | 72 |
| 18.2 | Hall, 2023 | 132 | NA | LJ or 7H | AMK | WHO_current | 11 | 78 | Mykrobe | 11 | 2 | 0 | 76 |
| 18.2 | Hall, 2023 | 132 | NA | LJ or 7H | CPM | WHO_current | 1 | 51 | Mykrobe | 0 | 1 | 1 | 50 |
| 18.2 | Hall, 2023 | 132 | NA | LJ or 7H | EMB | WHO_current | 14 | 77 | Mykrobe | 10 | 14 | 4 | 63 |
| 18.2 | Hall, 2023 | 132 | NA | LJ or 7H | INH | WHO_current | 51 | 48 | Mykrobe | 42 | 4 | 9 | 44 |
| 18.2 | Hall, 2023 | 132 | NA | LJ or 7H | KM | WHO_current | 0 | 52 | Mykrobe | 0 | 1 | 0 | 51 |
| 18.2 | Hall, 2023 | 132 | NA | LJ or 7H | OFX | WHO_current | 10 | 77 | Mykrobe | 10 | 4 | 0 | 73 |
| 18.2 | Hall, 2023 | 132 | NA | LJ or 7H | RMP | WHO_current | 48 | 44 | Mykrobe | 42 | 1 | 6 | 43 |
| 18.2 | Hall, 2023 | 132 | NA | LJ or 7H | SM | WHO_current | 8 | 83 | Mykrobe | 5 | 11 | 3 | 72 |
| 19 | Lee, 2023 | 57 | 38 | LJ | AMG (AMK, KM, CPM) | WHO_current | 9 | 48 | TBProfiler | 9 | 1 | 0 | 47 |
| 19 | Lee, 2023 | 57 | 38 | LJ | CS | WHO_past | 2 | 55 | TBProfiler | 0 | 1 | 2 | 54 |
| 19 | Lee, 2023 | 57 | 38 | LJ | EMB | WHO_current | 23 | 34 | TBProfiler | 22 | 7 | 1 | 27 |
| 19 | Lee, 2023 | 57 | 38 | LJ | ETO/PTO | WHO_current | 10 | 47 | TBProfiler | 10 | 8 | 0 | 39 |
| 19 | Lee, 2023 | 57 | 38 | LJ | FLQ (LFX, MFX, OFX) | WHO_past | 17 | 40 | TBProfiler | 17 | 1 | 0 | 39 |
| 19 | Lee, 2023 | 57 | 38 | LJ | INH | WHO_current | 38 | 19 | TBProfiler | 35 | 1 | 3 | 18 |
| 19 | Lee, 2023 | 57 | 38 | LJ | LZD | WHO_undefined | 1 | 56 | TBProfiler | 1 | 0 | 0 | 56 |
| 19 | Lee, 2023 | 57 | 38 | LJ | PAS | WHO_past | 9 | 48 | TBProfiler | 6 | 2 | 3 | 46 |
| 19 | Lee, 2023 | 57 | 38 | Pyrazinamidase test | PZA | WHO_past | 19 | 38 | TBProfiler | 15 | 3 | 4 | 35 |
| 19 | Lee, 2023 | 57 | 38 | LJ | RMP | WHO_current | 37 | 20 | TBProfiler | 36 | 0 | 1 | 20 |
| 19 | Lee, 2023 | 57 | 38 | LJ | SM | WHO_current | 17 | 40 | TBProfiler | 14 | 4 | 3 | 36 |
| 20 | Wang, 2023 | 202 | 202 | LJ | CPM | WHO_current | 16 | 186 | TBProfiler | 15 | 3 | 1 | 183 |
| 20 | Wang, 2023 | 202 | 202 | LJ | EMB | WHO_current | 92 | 110 | TBProfiler | 86 | 39 | 6 | 71 |
| 20 | Wang, 2023 | 202 | 202 | LJ | ETO | WHO_current | 23 | 179 | TBProfiler | 17 | 21 | 6 | 158 |
| 20 | Wang, 2023 | 202 | 202 | LJ | INH | WHO_current | 202 | 0 | TBProfiler | 191 | 0 | 11 | 0 |
| 20 | Wang, 2023 | 202 | 202 | LJ | KM | WHO_current | 15 | 187 | TBProfiler | 15 | 0 | 0 | 187 |
| 20 | Wang, 2023 | 202 | 202 | LJ | OFX | WHO_past | 99 | 103 | TBProfiler | 91 | 6 | 8 | 97 |
| 20 | Wang, 2023 | 202 | 202 | LJ | RMP | WHO_current | 202 | 0 | TBProfiler | 200 | 0 | 2 | 0 |
| 20 | Wang, 2023 | 202 | 202 | LJ | SM | WHO_current | 122 | 80 | TBProfiler | 98 | 2 | 24 | 78 |
| 21 | Xiao, 2023 | 200 | 136 | 7H11 | AMK | WHO_undefined | 8 | 192 | TGS-TB | 7 | 0 | 1 | 192 |
| 21 | Xiao, 2023 | 200 | 136 | 7H11 | CPM | WHO_undefined | 6 | 194 | TGS-TB | 5 | 3 | 1 | 191 |
| 21 | Xiao, 2023 | 200 | 136 | 7H10 | EMB | WHO_current | 77 | 123 | TGS-TB | 74 | 22 | 3 | 101 |
| 21 | Xiao, 2023 | 200 | 136 | 7H11 | ETO | WHO_current | 34 | 166 | TGS-TB | 33 | 31 | 1 | 135 |
| 21 | Xiao, 2023 | 200 | 136 | 7H10 | FLQ (MFX, OFX) | WHO_past | 22 | 178 | TGS-TB | 22 | 1 | 0 | 177 |
| 21 | Xiao, 2023 | 200 | 136 | 7H10 | INH | WHO_current | 136 | 64 | TGS-TB | 131 | 5 | 5 | 59 |
| 21 | Xiao, 2023 | 200 | 136 | 7H11 | KM | WHO_past | 11 | 189 | TGS-TB | 9 | 6 | 2 | 183 |
| 21 | Xiao, 2023 | 200 | 136 | 7H11 | PAS | WHO_past | 6 | 194 | TGS-TB | 6 | 1 | 0 | 193 |
| 21 | Xiao, 2023 | 200 | 136 | MGIT 960 | PZA | WHO_current | 40 | 160 | TGS-TB | 39 | 2 | 1 | 158 |
| 21 | Xiao, 2023 | 200 | 136 | 7H10 | RMP | WHO_current | 197 | 3 | TGS-TB | 196 | 3 | 1 | 0 |
| 21 | Xiao, 2023 | 200 | 136 | 7H10 | SM | WHO_current | 60 | 140 | TGS-TB | 58 | 22 | 2 | 118 |
| 22 | Morey-León, 2023 | 88 | 52 | MGIT 960 and/or LJ | AMK | WHO_current | 0 | 21 | KvarQ | 0 | 0 | 0 | 21 |
| 22 | Morey-León, 2023 | 88 | 52 | MGIT 960 and/or LJ | AMK | WHO_current | 0 | 21 | Mykrobe | 0 | 1 | 0 | 20 |
| 22 | Morey-León, 2023 | 88 | 52 | MGIT 960 and/or LJ | AMK | WHO_current | 0 | 21 | PhyResSE | 0 | 1 | 0 | 20 |
| 22 | Morey-León, 2023 | 88 | 52 | MGIT 960 and/or LJ | AMK | WHO_current | 0 | 21 | SAM-TB | 0 | 1 | 0 | 20 |
| 22 | Morey-León, 2023 | 88 | 52 | MGIT 960 and/or LJ | AMK | WHO_current | 0 | 21 | TBProfiler | 0 | 1 | 0 | 20 |
| 22 | Morey-León, 2023 | 88 | 52 | MGIT 960 and/or LJ | CPM | WHO_past | 0 | 21 | KvarQ | 0 | 0 | 0 | 21 |
| 22 | Morey-León, 2023 | 88 | 52 | MGIT 960 and/or LJ | CPM | WHO_past | 0 | 21 | Mykrobe | 0 | 1 | 0 | 20 |
| 22 | Morey-León, 2023 | 88 | 52 | MGIT 960 and/or LJ | CPM | WHO_past | 0 | 21 | PhyResSE | 0 | 1 | 0 | 20 |
| 22 | Morey-León, 2023 | 88 | 52 | MGIT 960 and/or LJ | CPM | WHO_past | 0 | 21 | SAM-TB | 0 | 1 | 0 | 20 |
| 22 | Morey-León, 2023 | 88 | 52 | MGIT 960 and/or LJ | CPM | WHO_past | 0 | 21 | TBProfiler | 0 | 1 | 0 | 20 |
| 22 | Morey-León, 2023 | 88 | 52 | MGIT 960 and/or LJ | EMB | WHO_current | 11 | 76 | KvarQ | 8 | 5 | 3 | 71 |
| 22 | Morey-León, 2023 | 88 | 52 | MGIT 960 and/or LJ | EMB | WHO_current | 11 | 76 | Mykrobe | 11 | 10 | 0 | 66 |
| 22 | Morey-León, 2023 | 88 | 52 | MGIT 960 and/or LJ | EMB | WHO_current | 11 | 76 | PhyResSE | 10 | 11 | 1 | 65 |
| 22 | Morey-León, 2023 | 88 | 52 | MGIT 960 and/or LJ | EMB | WHO_current | 11 | 76 | SAM-TB | 10 | 11 | 1 | 65 |
| 22 | Morey-León, 2023 | 88 | 52 | MGIT 960 and/or LJ | EMB | WHO_current | 11 | 76 | TBProfiler | 11 | 11 | 0 | 65 |
| 22 | Morey-León, 2023 | 88 | 52 | MGIT 960 and/or LJ | INH | WHO_current | 63 | 25 | KvarQ | 58 | 1 | 5 | 24 |
| 22 | Morey-León, 2023 | 88 | 52 | MGIT 960 and/or LJ | INH | WHO_current | 63 | 25 | Mykrobe | 60 | 1 | 3 | 24 |
| 22 | Morey-León, 2023 | 88 | 52 | MGIT 960 and/or LJ | INH | WHO_current | 63 | 25 | PhyResSE | 60 | 1 | 3 | 24 |
| 22 | Morey-León, 2023 | 88 | 52 | MGIT 960 and/or LJ | INH | WHO_current | 63 | 25 | SAM-TB | 60 | 1 | 3 | 24 |
| 22 | Morey-León, 2023 | 88 | 52 | MGIT 960 and/or LJ | INH | WHO_current | 63 | 25 | TBProfiler | 59 | 1 | 4 | 24 |
| 22 | Morey-León, 2023 | 88 | 52 | MGIT 960 and/or LJ | KM | WHO_current | 0 | 45 | KvarQ | 0 | 0 | 0 | 45 |
| 22 | Morey-León, 2023 | 88 | 52 | MGIT 960 and/or LJ | KM | WHO_current | 0 | 45 | Mykrobe | 0 | 2 | 0 | 43 |
| 22 | Morey-León, 2023 | 88 | 52 | MGIT 960 and/or LJ | KM | WHO_current | 0 | 45 | PhyResSE | 0 | 2 | 0 | 43 |
| 22 | Morey-León, 2023 | 88 | 52 | MGIT 960 and/or LJ | KM | WHO_current | 0 | 45 | SAM-TB | 0 | 0 | 0 | 45 |
| 22 | Morey-León, 2023 | 88 | 52 | MGIT 960 and/or LJ | KM | WHO_current | 0 | 45 | TBProfiler | 0 | 2 | 0 | 43 |
| 22 | Morey-León, 2023 | 88 | 52 | MGIT 960 and/or LJ | LFX | WHO_past | 7 | 39 | KvarQ | 3 | 0 | 4 | 39 |
| 22 | Morey-León, 2023 | 88 | 52 | MGIT 960 and/or LJ | LFX | WHO_past | 7 | 39 | Mykrobe | 3 | 1 | 4 | 38 |
| 22 | Morey-León, 2023 | 88 | 52 | MGIT 960 and/or LJ | LFX | WHO_past | 7 | 39 | PhyResSE | 3 | 1 | 4 | 38 |
| 22 | Morey-León, 2023 | 88 | 52 | MGIT 960 and/or LJ | LFX | WHO_past | 7 | 39 | SAM-TB | 5 | 1 | 2 | 38 |
| 22 | Morey-León, 2023 | 88 | 52 | MGIT 960 and/or LJ | LFX | WHO_past | 7 | 39 | TBProfiler | 3 | 1 | 4 | 38 |
| 22 | Morey-León, 2023 | 88 | 52 | MGIT 960 and/or LJ | MFX | WHO_past | 2 | 19 | KvarQ | 1 | 1 | 1 | 18 |
| 22 | Morey-León, 2023 | 88 | 52 | MGIT 960 and/or LJ | MFX | WHO_past | 2 | 19 | Mykrobe | 1 | 2 | 1 | 17 |
| 22 | Morey-León, 2023 | 88 | 52 | MGIT 960 and/or LJ | MFX | WHO_past | 2 | 19 | PhyResSE | 1 | 2 | 1 | 17 |
| 22 | Morey-León, 2023 | 88 | 52 | MGIT 960 and/or LJ | MFX | WHO_past | 2 | 19 | SAM-TB | 1 | 4 | 1 | 15 |
| 22 | Morey-León, 2023 | 88 | 52 | MGIT 960 and/or LJ | MFX | WHO_past | 2 | 19 | TBProfiler | 1 | 2 | 1 | 17 |
| 22 | Morey-León, 2023 | 88 | 52 | Pyrazinamidase test | PZA | WHO_past | 12 | 52 | KvarQ | 8 | 6 | 4 | 46 |
| 22 | Morey-León, 2023 | 88 | 52 | Pyrazinamidase test | PZA | WHO_past | 12 | 52 | Mykrobe | 10 | 3 | 2 | 49 |
| 22 | Morey-León, 2023 | 88 | 52 | Pyrazinamidase test | PZA | WHO_past | 12 | 52 | PhyResSE | 9 | 7 | 3 | 45 |
| 22 | Morey-León, 2023 | 88 | 52 | Pyrazinamidase test | PZA | WHO_past | 12 | 52 | SAM-TB | 12 | 8 | 0 | 44 |
| 22 | Morey-León, 2023 | 88 | 52 | Pyrazinamidase test | PZA | WHO_past | 12 | 52 | TBProfiler | 10 | 7 | 2 | 45 |
| 22 | Morey-León, 2023 | 88 | 52 | MGIT 960 and/or LJ | RMP | WHO_current | 66 | 22 | KvarQ | 58 | 0 | 8 | 22 |
| 22 | Morey-León, 2023 | 88 | 52 | MGIT 960 and/or LJ | RMP | WHO_current | 66 | 22 | Mykrobe | 63 | 0 | 3 | 22 |
| 22 | Morey-León, 2023 | 88 | 52 | MGIT 960 and/or LJ | RMP | WHO_current | 66 | 22 | PhyResSE | 64 | 0 | 2 | 22 |
| 22 | Morey-León, 2023 | 88 | 52 | MGIT 960 and/or LJ | RMP | WHO_current | 66 | 22 | SAM-TB | 64 | 0 | 2 | 22 |
| 22 | Morey-León, 2023 | 88 | 52 | MGIT 960 and/or LJ | RMP | WHO_current | 66 | 22 | TBProfiler | 66 | 3 | 0 | 19 |
| 22 | Morey-León, 2023 | 88 | 52 | MGIT 960 and/or LJ | SM | WHO_current | 16 | 5 | KvarQ | 9 | 0 | 7 | 5 |
| 22 | Morey-León, 2023 | 88 | 52 | MGIT 960 and/or LJ | SM | WHO_current | 16 | 5 | Mykrobe | 9 | 0 | 7 | 5 |
| 22 | Morey-León, 2023 | 88 | 52 | MGIT 960 and/or LJ | SM | WHO_current | 16 | 5 | PhyResSE | 11 | 0 | 5 | 5 |
| 22 | Morey-León, 2023 | 88 | 52 | MGIT 960 and/or LJ | SM | WHO_current | 16 | 5 | SAM-TB | 12 | 0 | 4 | 5 |
| 22 | Morey-León, 2023 | 88 | 52 | MGIT 960 and/or LJ | SM | WHO_current | 16 | 5 | TBProfiler | 11 | 0 | 5 | 5 |
| 23 | Billard-Pomares, 2022 | 227 | 4 | MGIT 960 | EMB | WHO_current | 1 | 226 | TBProfiler | 1 | 2 | 0 | 224 |
| 23 | Billard-Pomares, 2022 | 227 | 4 | MGIT 960 | INH | WHO_current | 18 | 209 | TBProfiler | 18 | 0 | 0 | 209 |
| 23 | Billard-Pomares, 2022 | 227 | 4 | MGIT 960 | PZA | WHO_current | 12 | 215 | TBProfiler | 8 | 0 | 4 | 215 |
| 23 | Billard-Pomares, 2022 | 227 | 4 | MGIT 960 | RMP | WHO_current | 5 | 222 | TBProfiler | 5 | 0 | 0 | 222 |
| 24 | Quagliaro, 2023 | 227 | 4 | MGIT 960 | EMB | WHO_current | 1 | 226 | Mykrobe | 1 | 1 | 0 | 225 |
| 24 | Quagliaro, 2023 | 227 | 4 | MGIT 960 | EMB | WHO_current | 1 | 226 | PhyResSE | 1 | 2 | 0 | 224 |
| 24 | Quagliaro, 2023 | 227 | 4 | MGIT 960 | INH | WHO_current | 18 | 209 | Mykrobe | 15 | 0 | 3 | 209 |
| 24 | Quagliaro, 2023 | 227 | 4 | MGIT 960 | INH | WHO_current | 18 | 209 | PhyResSE | 15 | 0 | 3 | 209 |
| 24 | Quagliaro, 2023 | 227 | 4 | MGIT 960 | PZA | WHO_current | 12 | 215 | Mykrobe | 8 | 0 | 4 | 215 |
| 24 | Quagliaro, 2023 | 227 | 4 | MGIT 960 | PZA | WHO_current | 12 | 215 | PhyResSE | 8 | 3 | 4 | 212 |
| 24 | Quagliaro, 2023 | 227 | 4 | MGIT 960 | RMP | WHO_current | 5 | 222 | Mykrobe | 4 | 2 | 1 | 220 |
| 24 | Quagliaro, 2023 | 227 | 4 | MGIT 960 | RMP | WHO_current | 5 | 222 | PhyResSE | 5 | 0 | 0 | 222 |
| 25 | Daniyarov, 2023 | 10 | 10 | MGIT 960 | AMK | WHO_current | 2 | 8 | CASTB | 2 | 0 | 0 | 0 |
| 25 | Daniyarov, 2023 | 10 | 10 | MGIT 960 | AMK | WHO_current | 2 | 8 | Mykrobe | 2 | 0 | 0 | 8 |
| 25 | Daniyarov, 2023 | 10 | 10 | MGIT 960 | AMK | WHO_current | 2 | 8 | TBProfiler | 2 | 0 | 0 | 0 |
| 25 | Daniyarov, 2023 | 10 | 10 | MGIT 960 | CPM | WHO_current | 3 | 7 | CASTB | - | - | - | - |
| 25 | Daniyarov, 2023 | 10 | 10 | MGIT 960 | CPM | WHO_current | 3 | 7 | Mykrobe | 2 | 0 | 1 | 7 |
| 25 | Daniyarov, 2023 | 10 | 10 | MGIT 960 | CPM | WHO_current | 3 | 7 | TBProfiler | 2 | 0 | 0 | 0 |
| 25 | Daniyarov, 2023 | 10 | 10 | MGIT 960 | EMB | WHO_current | 4 | 6 | CASTB | 4 | 5 | 0 | 0 |
| 25 | Daniyarov, 2023 | 10 | 10 | MGIT 960 | EMB | WHO_current | 4 | 6 | Mykrobe | 4 | 5 | 0 | 1 |
| 25 | Daniyarov, 2023 | 10 | 10 | MGIT 960 | EMB | WHO_current | 4 | 6 | TBProfiler | 4 | 6 | 0 | 0 |
| 25 | Daniyarov, 2023 | 10 | 10 | MGIT 960 | ETO | WHO_current | 4 | 6 | CASTB | - | - | - | - |
| 25 | Daniyarov, 2023 | 10 | 10 | MGIT 960 | ETO | WHO_current | 4 | 6 | Mykrobe | - | - | - | - |
| 25 | Daniyarov, 2023 | 10 | 10 | MGIT 960 | ETO | WHO_current | 4 | 6 | TBProfiler | 1 | 2 | 0 | 0 |
| 25 | Daniyarov, 2023 | 10 | 10 | MGIT 960 | INH | WHO_current | 10 | 0 | CASTB | 10 | 0 | 0 | 0 |
| 25 | Daniyarov, 2023 | 10 | 10 | MGIT 960 | INH | WHO_current | 10 | 0 | Mykrobe | 10 | 0 | 0 | 0 |
| 25 | Daniyarov, 2023 | 10 | 10 | MGIT 960 | INH | WHO_current | 10 | 0 | TBProfiler | 10 | 0 | 0 | 0 |
| 25 | Daniyarov, 2023 | 10 | 10 | MGIT 960 | KM | WHO_current | 3 | 7 | CASTB | - | - | - | - |
| 25 | Daniyarov, 2023 | 10 | 10 | MGIT 960 | KM | WHO_current | 3 | 7 | Mykrobe | 2 | 1 | 1 | 6 |
| 25 | Daniyarov, 2023 | 10 | 10 | MGIT 960 | KM | WHO_current | 3 | 7 | TBProfiler | 3 | 2 | 0 | 0 |
| 25 | Daniyarov, 2023 | 10 | 10 | MGIT 960 | LFX | WHO_past | 8 | 2 | CASTB | - | - | - | - |
| 25 | Daniyarov, 2023 | 10 | 10 | MGIT 960 | LFX | WHO_past | 8 | 2 | Mykrobe | - | - | - | - |
| 25 | Daniyarov, 2023 | 10 | 10 | MGIT 960 | LFX | WHO_past | 8 | 2 | TBProfiler | 8 | 2 | 0 | 0 |
| 25 | Daniyarov, 2023 | 10 | 10 | MGIT 960 | MFX | WHO_current | 10 | 0 | CASTB | - | - | - | - |
| 25 | Daniyarov, 2023 | 10 | 10 | MGIT 960 | MFX | WHO_current | 10 | 0 | Mykrobe | 10 | 0 | 0 | 0 |
| 25 | Daniyarov, 2023 | 10 | 10 | MGIT 960 | MFX | WHO_current | 10 | 0 | TBProfiler | 10 | 0 | 0 | 0 |
| 25 | Daniyarov, 2023 | 10 | 10 | MGIT 960 | PZA | WHO_current | 9 | 1 | CASTB | - | - | - | - |
| 25 | Daniyarov, 2023 | 10 | 10 | MGIT 960 | PZA | WHO_current | 9 | 1 | Mykrobe | 7 | 0 | 2 | 1 |
| 25 | Daniyarov, 2023 | 10 | 10 | MGIT 960 | PZA | WHO_current | 9 | 1 | TBProfiler | - | - | - | - |
| 25 | Daniyarov, 2023 | 10 | 10 | MGIT 960 | RMP | WHO_current | 10 | 0 | CASTB | 9 | 0 | 0 | 0 |
| 25 | Daniyarov, 2023 | 10 | 10 | MGIT 960 | RMP | WHO_current | 10 | 0 | Mykrobe | 10 | 0 | 0 | 0 |
| 25 | Daniyarov, 2023 | 10 | 10 | MGIT 960 | RMP | WHO_current | 10 | 0 | TBProfiler | 10 | 0 | 0 | 0 |
| 25 | Daniyarov, 2023 | 10 | 10 | MGIT 960 | SM | WHO_current | 3 | 7 | CASTB | 3 | 7 | 0 | 0 |
| 25 | Daniyarov, 2023 | 10 | 10 | MGIT 960 | SM | WHO_current | 3 | 7 | Mykrobe | 3 | 7 | 0 | 0 |
| 25 | Daniyarov, 2023 | 10 | 10 | MGIT 960 | SM | WHO_current | 3 | 7 | TBProfiler | 3 | 7 | 0 | 0 |
| 26 | Lim, 2023 | 3808 | 58 | MGIT | EMB | NA | 14 | 2984 | TBProfiler | 13 | 38 | 1 | 2946 |
| 26 | Lim, 2023 | 3808 | 58 | MGIT | INH | NA | 213 | 2781 | TBProfiler | 202 | 11 | 11 | 2770 |
| 26 | Lim, 2023 | 3808 | 58 | MGIT | PZA | NA | 46 | 2092 | TBProfiler | 30 | 2 | 16 | 2090 |
| 26 | Lim, 2023 | 3808 | 58 | MGIT | RMP | NA | 62 | 2934 | TBProfiler | 61 | 12 | 1 | 2922 |
| 27 | Shaw, 2023 | 38 | 3 | MGIT and solid agar | EMB | NA | 6 | 32 | TBProfiler | 5 | 0 | 1 | 32 |
| 27 | Shaw, 2023 | 38 | 3 | MGIT and solid agar | INH | NA | 8 | 30 | TBProfiler | 7 | 1 | 1 | 29 |
| 27 | Shaw, 2023 | 38 | 3 | MGIT and solid agar | PZA | NA | 1 | 37 | TBProfiler | 1 | 0 | 0 | 37 |
| 27 | Shaw, 2023 | 38 | 3 | MGIT and solid agar | RMP | NA | 1 | 37 | TBProfiler | 1 | 0 | 0 | 37 |
| 27 | Shaw, 2023 | 38 | 3 | MGIT and solid agar | SM | NA | 5 | 33 | TBProfiler | 5 | 0 | 0 | 33 |
| 28 | Cloutier Charette, 2024 | 72 | 32 | LJ | AMK | NA | 0 | 36 | Mykrobe | 0 | 1 | 0 | 35 |
| 28 | Cloutier Charette, 2024 | 72 | 32 | LJ | CPM | NA | 1 | 35 | Mykrobe | 0 | 1 | 1 | 34 |
| 28 | Cloutier Charette, 2024 | 72 | 32 | LJ | EMB | NA | 12 | 60 | Mykrobe | 9 | 11 | 3 | 49 |
| 28 | Cloutier Charette, 2024 | 72 | 32 | LJ | FLQ | NA | 0 | 36 | Mykrobe | 0 | 0 | 0 | 36 |
| 28 | Cloutier Charette, 2024 | 72 | 32 | LJ | INH | NA | 34 | 38 | Mykrobe | 30 | 1 | 4 | 37 |
| 28 | Cloutier Charette, 2024 | 72 | 32 | LJ | KM | NA | 0 | 36 | Mykrobe | 0 | 1 | 0 | 35 |
| 28 | Cloutier Charette, 2024 | 72 | 32 | LJ | RMP |  | 33 | 39 | Mykrobe | 31 | 0 | 2 | 39 |
| 28 | Cloutier Charette, 2024 | 72 | 32 | LJ | SM | NA | 5 | 65 | Mykrobe | 4 | 10 | 1 | 55 |
| 29 | He, 2024 | 110 | 100 | LJ | AMK | WHO_current | 9 | 101 | GenTB | 9 | 2 | 0 | 99 |
| 29 | He, 2024 | 110 | 100 | LJ | AMK | WHO_current | 7 | 103 | Mykrobe | 7 | 4 | 0 | 99 |
| 29 | He, 2024 | 110 | 100 | LJ | AMK | WHO_current | 9 | 101 | PhyResSE | 9 | 2 | 0 | 99 |
| 29 | He, 2024 | 110 | 100 | LJ | AMK | WHO_current | 9 | 101 | SAM-TB | 9 | 2 | 0 | 99 |
| 29 | He, 2024 | 110 | 100 | LJ | AMK | WHO_current | 9 | 101 | TBProfiler | 9 | 2 | 0 | 99 |
| 29 | He, 2024 | 110 | 100 | LJ | CPM | WHO_current | 11 | 99 | GenTB | 8 | 1 | 3 | 98 |
| 29 | He, 2024 | 110 | 100 | LJ | CPM | WHO_current | 7 | 103 | Mykrobe | 7 | 2 | 0 | 101 |
| 29 | He, 2024 | 110 | 100 | LJ | CPM | WHO_current | 9 | 101 | PhyResSE | 8 | 1 | 1 | 100 |
| 29 | He, 2024 | 110 | 100 | LJ | CPM | WHO_current | 9 | 101 | SAM-TB | 8 | 1 | 1 | 100 |
| 29 | He, 2024 | 110 | 100 | LJ | CPM | WHO_current | 9 | 101 | TBProfiler | 8 | 1 | 1 | 100 |
| 29 | He, 2024 | 110 | 100 | LJ | EMB | WHO_current | 49 | 61 | GenTB | 39 | 19 | 10 | 42 |
| 29 | He, 2024 | 110 | 100 | LJ | EMB | WHO_current | 73 | 37 | Mykrobe | 55 | 3 | 18 | 34 |
| 29 | He, 2024 | 110 | 100 | LJ | EMB | WHO_current | 64 | 46 | PhyResSE | 49 | 9 | 15 | 37 |
| 29 | He, 2024 | 110 | 100 | LJ | EMB | WHO_current | 71 | 39 | SAM-TB | 53 | 5 | 18 | 34 |
| 29 | He, 2024 | 110 | 100 | LJ | EMB | WHO_current | 75 | 35 | TBProfiler | 56 | 2 | 19 | 33 |
| 29 | He, 2024 | 110 | 100 | LJ | INH | WHO_current | 82 | 28 | GenTB | 82 | 19 | 0 | 9 |
| 29 | He, 2024 | 110 | 100 | LJ | INH | WHO_current | 94 | 16 | Mykrobe | 94 | 7 | 0 | 9 |
| 29 | He, 2024 | 110 | 100 | LJ | INH | WHO_current | 88 | 22 | PhyResSE | 88 | 13 | 0 | 9 |
| 29 | He, 2024 | 110 | 100 | LJ | INH | WHO_current | 90 | 20 | SAM-TB | 90 | 11 | 0 | 9 |
| 29 | He, 2024 | 110 | 100 | LJ | INH | WHO_current | 95 | 15 | TBProfiler | 95 | 6 | 0 | 9 |
| 29 | He, 2024 | 110 | 100 | LJ | KM | WHO_current | 11 | 99 | GenTB | 9 | 2 | 2 | 97 |
| 29 | He, 2024 | 110 | 100 | LJ | KM | WHO_current | 8 | 102 | Mykrobe | 7 | 4 | 1 | 98 |
| 29 | He, 2024 | 110 | 100 | LJ | KM | WHO_current | 10 | 100 | PhyResSE | 9 | 2 | 1 | 98 |
| 29 | He, 2024 | 110 | 100 | LJ | KM | WHO_current | 9 | 101 | SAM-TB | 9 | 2 | 0 | 99 |
| 29 | He, 2024 | 110 | 100 | LJ | KM | WHO_current | 10 | 100 | TBProfiler | 9 | 2 | 1 | 98 |
| 29 | He, 2024 | 110 | 100 | LJ | PAS | WHO_past | / | / | GenTB | / | / | / | / |
| 29 | He, 2024 | 110 | 100 | LJ | PAS | WHO_past | / | / | Mykrobe | / | / | / | / |
| 29 | He, 2024 | 110 | 100 | LJ | PAS | WHO_past | / | / | PhyResSE | / | / | / | / |
| 29 | He, 2024 | 110 | 100 | LJ | PAS | WHO_past | 6 | 104 | SAM-TB | 5 | 2 | 1 | 102 |
| 29 | He, 2024 | 110 | 100 | LJ | PAS | WHO_past | 8 | 102 | TBProfiler | 7 | 0 | 1 | 102 |
| 29 | He, 2024 | 110 | 100 | MGIT 960 | PTO | WHO_current | 1 | 109 | GenTB | 0 | 23 | 1 | 86 |
| 29 | He, 2024 | 110 | 100 | MGIT 960 | PTO | WHO_current | 30 | 80 | Mykrobe | 14 | 9 | 16 | 71 |
| 29 | He, 2024 | 110 | 100 | MGIT 960 | PTO | WHO_current | / | / | PhyResSE | / | / | / | / |
| 29 | He, 2024 | 110 | 100 | MGIT 960 | PTO | WHO_current | 1 | 109 | SAM-TB | 1 | 22 | 0 | 87 |
| 29 | He, 2024 | 110 | 100 | MGIT 960 | PTO | WHO_current | 14 | 96 | TBProfiler | 7 | 16 | 7 | 80 |
| 29 | He, 2024 | 110 | 100 | MGIT 960 | PZA | WHO_current | 22 | 88 | GenTB | 21 | 44 | 1 | 44 |
| 29 | He, 2024 | 110 | 100 | MGIT 960 | PZA | WHO_current | 45 | 65 | Mykrobe | 43 | 22 | 2 | 43 |
| 29 | He, 2024 | 110 | 100 | MGIT 960 | PZA | WHO_current | 18 | 92 | PhyResSE | 16 | 49 | 2 | 43 |
| 29 | He, 2024 | 110 | 100 | MGIT 960 | PZA | WHO_current | 46 | 64 | SAM-TB | 42 | 23 | 4 | 41 |
| 29 | He, 2024 | 110 | 100 | MGIT 960 | PZA | WHO_current | 59 | 51 | TBProfiler | 55 | 10 | 4 | 41 |
| 29 | He, 2024 | 110 | 100 | LJ | RMP | WHO_current | 85 | 25 | GenTB | 85 | 15 | 0 | 10 |
| 29 | He, 2024 | 110 | 100 | LJ | RMP | WHO_current | 97 | 13 | Mykrobe | 97 | 3 | 0 | 10 |
| 29 | He, 2024 | 110 | 100 | LJ | RMP | WHO_current | 92 | 18 | PhyResSE | 92 | 8 | 0 | 10 |
| 29 | He, 2024 | 110 | 100 | LJ | RMP | WHO_current | 97 | 13 | SAM-TB | 97 | 3 | 0 | 10 |
| 29 | He, 2024 | 110 | 100 | LJ | RMP | WHO_current | 97 | 13 | TBProfiler | 97 | 3 | 0 | 10 |
| 29 | He, 2024 | 110 | 100 | LJ | SM | WHO_current | 61 | 49 | GenTB | 61 | 8 | 0 | 41 |
| 29 | He, 2024 | 110 | 100 | LJ | SM | WHO_current | 61 | 49 | Mykrobe | 61 | 8 | 0 | 41 |
| 29 | He, 2024 | 110 | 100 | LJ | SM | WHO_current | 53 | 57 | PhyResSE | 53 | 16 | 0 | 41 |
| 29 | He, 2024 | 110 | 100 | LJ | SM | WHO_current | 65 | 45 | SAM-TB | 64 | 5 | 1 | 40 |
| 29 | He, 2024 | 110 | 100 | LJ | SM | WHO_current | 65 | 45 | TBProfiler | 64 | 5 | 1 | 40 |
| 30 | Liu, 2024 | 297 | 297 | 7H10 | AMK | WHO_past | 12 | 285 | TBProfiler | 12 | 3 | 0 | 282 |
| 30 | Liu, 2024 | 297 | 297 | 7H10 | CPM | WHO_past | 13 | 284 | TBProfiler | 12 | 1 | 1 | 283 |
| 30 | Liu, 2024 | 297 | 297 | 7H10 | EMB | WHO_current | 148 | 149 | TBProfiler | 139 | 37 | 9 | 112 |
| 30 | Liu, 2024 | 297 | 297 | 7H10 | ETO | WHO_current | 93 | 204 | TBProfiler | 83 | 48 | 10 | 156 |
| 30 | Liu, 2024 | 297 | 297 | 7H10 | FLQ (MFX, LFX) | WHO_current | 35 | 262 | TBProfiler | 34 | 0 | 1 | 262 |
| 30 | Liu, 2024 | 297 | 297 | 7H10 | INH | WHO_current | 297 | 0 | TBProfiler | 295 | 0 | 2 | 0 |
| 30 | Liu, 2024 | 297 | 297 | 7H10 | KM | WHO_past | 20 | 277 | TBProfiler | 18 | 4 | 2 | 273 |
| 30 | Liu, 2024 | 297 | 297 | 7H10 | PAS | WHO_past | 12 | 285 | TBProfiler | 10 | 13 | 2 | 272 |
| 30 | Liu, 2024 | 297 | 297 | MGIT 960 | PZA | WHO_current | 84 | 213 | TBProfiler | 82 | 6 | 2 | 207 |
| 30 | Liu, 2024 | 297 | 297 | 7H10 | RMP | WHO_current | 297 | 0 | TBProfiler | 297 | 0 | 0 | 0 |
| 30 | Liu, 2024 | 297 | 297 | 7H10 | SM | WHO_current | 137 | 160 | TBProfiler | 119 | 23 | 18 | 137 |
| 31 | Rukmana, 2024a | 60 | 7 | MGIT 960 | BDQ | NA | 3 | 57 | Mykrobe | 3 | 0 | 0 | 57 |
| 32 | Rukmana, 2024b | 34 | NA | MGIT 960 | PZA | WHO_current | 8 | 26 | GenTB | 7 | 1 | 1 | 25 |
| 32 | Rukmana, 2024b | 34 | NA | MGIT 960 | PZA | WHO_current | 8 | 26 | Mykrobe | 6 | 1 | 2 | 25 |
| 33 | Sadovska, 2024 | 46 | NA | MGIT 960 and/or LJ | AMK | WHO_current | 5 | 8 | TBProfiler | 4 | 0 | 1 | 8 |
| 33 | Sadovska, 2024 | 46 | NA | MGIT 960 and/or LJ | EMB | WHO_current | 16 | 30 | TBProfiler | 15 | 2 | 1 | 28 |
| 33 | Sadovska, 2024 | 46 | NA | MGIT 960 and/or LJ | ETO | WHO_current | 6 | 10 | TBProfiler | 5 | 7 | 1 | 3 |
| 33 | Sadovska, 2024 | 46 | NA | MGIT 960 and/or LJ | FLQ (OFX) | WHO_current | 5 | 13 | TBProfiler | 5 | 0 | 0 | 13 |
| 33 | Sadovska, 2024 | 46 | NA | MGIT 960 and/or LJ | INH | WHO_current | 23 | 23 | TBProfiler | 23 | 1 | 0 | 22 |
| 33 | Sadovska, 2024 | 46 | NA | MGIT 960 and/or LJ | PAS | WHO_past | 3 | 14 | TBProfiler | 1 | 3 | 2 | 11 |
| 33 | Sadovska, 2024 | 46 | NA | MGIT 960 and/or LJ | PZA | WHO_current | 7 | 9 | TBProfiler | 6 | 2 | 1 | 7 |
| 33 | Sadovska, 2024 | 46 | NA | MGIT 960 and/or LJ | RMP | WHO_current | 18 | 28 | TBProfiler | 17 | 1 | 1 | 27 |
| 33 | Sadovska, 2024 | 46 | NA | MGIT 960 and/or LJ | SM | WHO_current | 16 | 8 | TBProfiler | 16 | 0 | 0 | 8 |
