# Supplementary material for: Diagnostic yield of nine user-friendly bioinformatics tools for predicting Mycobacterium tuberculosis drug resistance: A systematic review and network meta-analysis
Source: PLOS Glob Public Health. 2025 Apr 21;5(4):e0004465. doi: 10.1371/journal.pgph.0004465 (PMC12011222; doi:10.1371/journal.pgph.0004465)
Supplement: S3 Table — (DOCX) [file pgph.0004465.s012.docx]

| Table 3. Comparison of features among bioinformatics tools | | | | | | | | | | | |
| --- | --- | --- | --- | --- | --- | --- | --- | --- | --- | --- | --- |
|  | Features | KvarQ | PhyResSE | TGS-TB | CASTB | Mykrobe | TBProfiler | MTBseq | SAM-TB | GenTB | Resistance Sniffer |
| Availability and requirements | Year | 2014 | 2015 | 2015 | 2015 | 2015, 2019 | 2015, 2019 | 2018 | 2022 | 2021 | 2019 |
|  | Latest version, year | v0.12.2, 2014 | v.1.0, 2015 | NA | NA | v.0.13.0, 2023 | v.5.0.1, 2023 | v.1.1.0, 2023 | NA | NA | NA |
|  | Project home page | https://kvarq.readthedocs.io/en/latest/ | https://bioinf.fz-borstel.de/mchips/phyresse/ | https://gph.niid.go.jp/tgs-tb/ | http://castb.ri.ncgm.go.jp/CASTB | https://www.mykrobe.com/ | https://tbdr.lshtm.ac.uk/ | https://github.com/ngs-fzb/MTBseq_source | https://samtb.uni-medica.com/index | https://gentb.hms.harvard.edu/ | http://resistance-sniffer.bi.up.ac.za/clader.php?clade=Mycobacterium_tuberculosis |
|  | Organization | Swiss Tropical and Public Health Institute | Research Center Borstel, Leibniz Lung Center, Germany | National Institute of Infectious Diseases, Japan | National Center for Global Health and Medicine, Japan | European Bioinformatics Institute | London School of Hygiene & Tropical Medicine | Research Center Borstel, Germany | Fudan University, China | Harvard University | University of Pretoria, South Africa |
|  | Programming Language | Python/C | Perl | Python/C | NA | Python 2 or 3 | Python | Perl | NA | Python 2 and 3, R, Perl | Python 2.7 (2.5) |
|  | Operating system(s) | Platform independent | Windows, Linux, MacOsX | Platform independent | Platform independent | Windows, Linux, MacOsX | Linux, MacOsX | Linux | Platform independent | Platform independent | Platform independent |
| Workfolw | Data sources | Illumina | Illumina or Ion Torrent | Illumina | Illumina | Illumina or Oxford Nanopore | Illumina or Oxford Nanopore | Illumina | Illumina | Illumina | Illumina |
|  | Input format | FastQ | FastQ | FastQ | FastQ, FastA | FastQ | FastQ | FastQ | FastQ | FastQ | FastA, FastQ |
|  | Quality Control | No | Yes, FastQC | No | No | No | Yes, Trimmomatic | No | Yes, FastQC, Cutadapt | Yes, fastQ Validator, fastp | No |
|  | Pipeline | Custom scripts | BWA-MEM, FastQC, Qualimap, SAMtools, GATK | Custom scripts, NUCmer | Custom scripts | SAMtools | Trimmomatic, BWA/bowtie2,GATK, Minimap2, BCFtools, Delly | BWA-MEM, SAMTools, PICARD-tools, GATK | FastQC, Cutadapt, BWA-MEM, SAMtools, Picard | Fastp, minimap2, Pilon, Variant annotation, Prediction matrix | Custom scripts |
|  | Outputs | Text file in json format | Html | NA | NA | Html | Json, txt, pdf and html formats | Speciﬁc report ﬁle | Html, csv | Heatmap and barplot | Bar plot |
|  | Resistance | Yes, SNP | Yes, SNP/indel | Yes, SNP | Yes, SNP | Yes, SNP/indel | Yes, SNP/indel | Yes, SNP | Yes, SNP and indel | Yes, SNP/indel | Yes, SNP |
|  | Lineage | Yes | Yes | Yes | Yes | Yes | Yes | Yes | Yes | Yes | Yes |
|  | Phylogenetic | Yes | Yes | Yes | Yes | No | Yes | Yes | Yes | Yes | Yes |
|  | NTM | No | No | No | No | Yes | No | No | Yes | No | No |
|  | User interfaces | Commandline or local computer | Webserver | Webserver | Webserver | Commandline or local computer | Commandline or Webserver | Commandline | Webserver | Commandline or Webserver | Webserver |
| Characteristics | Batch upload | Yes | Yes | Yes | No | No | Yes | Yes | Yes | Yes | Yes |
|  | Run-time (median) | 2 min | 15 min | 2 min | 10 min | 3 min | 5 min | NA | 30-150 min | 35 min | Several seconds to a few minutes |
|  | Allow inspection and  modification | Yes | No | Yes | No | Yes | Yes | Yes | Yes | No | No |
|  | Drugs | INH, RMP, EMB, SM, AMK, KM, FLQ | INH, RMP, EMB, SM, PZA, LFX, MFX, GFX, AMK, KM, CPM, ETO | INH, RMP, EMB, SM, PZA, KM, FLQ | INH, RMP, EMB, SM, PZA, CFX | INH, RMP, EMB, SM, PZA, LFX, MFX, GFX, AMK, KM, CPM | INH, RMP, EMB, SM, PZA, AMK, KM, CPM, FLQ, ETO, PAS, CS, LZD, BDQ, CFZ, DLM | INH, RMP, EMB, SM, PZA, AMK, KM, CPM, FLQ, ETO, PAS, CS, LZD, BDQ, CFZ, DLM | INH, RMP, EMB, SM, PZA, ETO, AMK, KM, CPM, OFX/LFX, MFX, PAS, CS, LZD, BDQ, CFZ, DLM | INH, RMP, EMB, SM, PZA, MFX, LFX, CFX, OFX, KM, CPM, ETO, PAS | INH, RMP, EM, SM, PZA, FLQ, PSA, CS, AMK, CPM, KM |
|  | Unique features | User-friendly, directly extracts relevant information from fastq files | Rigorous preprocessing and QC | User-friendly | User-friendly | Identify species of mixed infections | Resistance mutation library | Having a full automated analysis pipeline | Performs MTBC genetic relationship analysis and NTM species identification | Two potential predictors, GenTB-RF and GenTB-WDNN | User-friendly |
| Abbreviations: AMK, amikacin; AMG, aminoglycosides; BDQ, bedaquiline; CPM, capreomycin; CFX, ciprofloxacin; CFZ, clofazimine; DCS, D-cycloserine; CS, cycloserine; DLM, delamanid; EMB, ethambutol; ETO, ethionamide; FLQ, fluoroquinolones; GFX, gatifloxacin; INH, isoniazid; KM, kanamycin; LFX, levofloxacin; LZD, linezolid; MFX, moxifloxacin; OFX, ofloxacin; PAS, para-aminosalicylic acid; PZA, pyrazinamide; PTO, prothionamide; RMP, rifampicin; SM, streptomycin; SNP, single-nucleotide polymorphism; NA, Not available. | | | | | | | | | | | |
