# Supplementary material for: Diagnostic yield of nine user-friendly bioinformatics tools for predicting Mycobacterium tuberculosis drug resistance: A systematic review and network meta-analysis
Source: PLOS Glob Public Health. 2025 Apr 21;5(4):e0004465. doi: 10.1371/journal.pgph.0004465 (PMC12011222; doi:10.1371/journal.pgph.0004465)
Supplement: S4 Table — (DOCX) [file pgph.0004465.s013.docx]

| Table 4. Methodological quality assessment of included studies based on QUADAS-2 | | | | | | | | | | | | | | | | | | |
| --- | --- | --- | --- | --- | --- | --- | --- | --- | --- | --- | --- | --- | --- | --- | --- | --- | --- | --- |
|  |  | Patient Selection | | | | | Index Test | | | | Reference Standard | | | | Flow and Timing | | | |
| NO. |  | Risk of Bias | | | | Concerns regarding applicability | Risk of Bias | | | Concerns regarding applicability | Risk of Bias | | | Concerns regarding applicability |  | Risk of Bias | | |
|  | Studies | Was a consecutive or random sample of patients enrolled? | Was a case-control design avoided? | Did the study avoid inappropriate exclusions? | Could the selection of patients have introduced bias? | Are there concerns that the included patients and setting do not match the review question? | Were the index test results interpreted without knowledge of the results of the reference standard? | If a threshold was used, was it pre-specified? | Could the conduct or interpretation of the index test have introduced bias? | Are there concerns that the index test, its conduct, or interpretation differ from the review question? | Is the reference standards likely to correctly classify the target condition? | Were the reference standard results interpreted without knowledge of the results of the index tests? | Could the reference standard, its conduct, or its interpretation have introduced bias? | Are there concerns that the target condition as defined by the reference standard does not match the question? | Was there an appropriate interval between index test and reference standard? | Did all patients receive the same reference standard? | Were all patients included in the analysis? | Could the patient flow have introduced bias? |
| 1 | Phelan, 2016 | Unclear | NO | NO | High risk | High concern | NO | Yes | Unclear risk | Low concern | Yes | Yes | Low risk | Low concern | Yes | Yes | Yes | Low risk |
| 2 | Schleusener, 2017 | Unclear | NO | NO | High risk | Unclear concern | NO | Yes | Unclear risk | Low concern | Yes | Yes | Low risk | Low concern | Yes | Yes | Yes | Low risk |
| 3 | Chatterjee, 2017 | Yes | Yes | Yes | Low risk | Low concern | NO | Yes | Unclear risk | Low concern | Yes | Yes | Low risk | Low concern | Yes | Yes | Yes | Low risk |
| 4 | Bouzouita, 2018 | Unclear | NO | NO | High risk | High concern | NO | Yes | Unclear risk | Low concern | Yes | Yes | Low risk | Low concern | Yes | Yes | Yes | Low risk |
| 5 | Macedo, 2018 | Unclear | NO | NO | High risk | High concern | NO | Yes | Unclear risk | Low concern | Yes | Yes | Low risk | Low concern | Yes | Yes | Yes | Low risk |
| 6 | Feliciano, 2018 | Unclear | NO | NO | High risk | Unclear concern | NO | Yes | Unclear risk | Low concern | Yes | Yes | Low risk | Low concern | Yes | Yes | Yes | Low risk |
| 7 | Faksri, 2019 | Unclear | NO | NO | High risk | Low concern | NO | Yes | Unclear risk | Low concern | Yes | Yes | Low risk | Low concern | Yes | Yes | NO | Low risk |
| 8 | Beek, 2019 | NO | NO | NO | High risk | High concern | NO | Yes | Unclear risk | Low concern | Yes | Yes | Low risk | Low concern | Yes | Yes | Yes | Low risk |
| 9 | Iwamoto, 2019 | Unclear | NO | NO | High risk | High concern | NO | Yes | Unclear risk | Low concern | Yes | Yes | Low risk | Low concern | Yes | Yes | Yes | Low risk |
| 10 | Guimarães, 2021 | Unclear | Unclear | Unclear | Unclear risk | Unclear concern | NO | Yes | Unclear risk | Low concern | Yes | Yes | Low risk | Low concern | Yes | Yes | Yes | Low risk |
| 11 | Nonghanphithak, 2020 | NO | NO | NO | High risk | High concern | NO | Yes | Unclear risk | Low concern | Yes | Yes | Low risk | Low concern | Yes | Yes | Yes | Low risk |
| 12 | Wu, 2020 | Unclear | NO | NO | High risk | Low concern | NO | Yes | Unclear risk | Low concern | Yes | Yes | Low risk | Low concern | Yes | Yes | Yes | Low risk |
| 13 | Genestet, 2020 | Yes | Yes | Yes | Low risk | Low concern | NO | Yes | Unclear risk | Low concern | Yes | Yes | Low risk | Low concern | Yes | Yes | Yes | Low risk |
| 14 | Kim, 2022 | Unclear | NO | NO | High risk | High concern | NO | Yes | Unclear risk | Low concern | Yes | Yes | Low risk | Low concern | Yes | Yes | Yes | Low risk |
| 15 | Che, 2022 | Unclear | NO | NO | High risk | High concern | NO | Yes | Unclear risk | Low concern | Yes | Yes | Low risk | Low concern | Yes | Yes | Yes | Low risk |
| 16 | Wu, 2022 | Unclear | Unclear | Unclear | Unclear risk | Unclear concern | NO | Yes | Unclear risk | Low concern | Yes | Yes | Low risk | Low concern | Yes | Yes | Yes | Low risk |
| 17 | Finci, 2022 | Unclear | NO | NO | High risk | Low concern | NO | Yes | Unclear risk | Low concern | Yes | Yes | Low risk | Low concern | Yes | Yes | Yes | Low risk |
| 18 | Hall, 2023 | Unclear | NO | NO | High risk | High concern | NO | Yes | Unclear risk | Low concern | Yes | Yes | Low risk | Low concern | Yes | NO | NO | High risk |
| 19 | Lee, 2023 | Unclear | Unclear | Unclear | Unclear risk | High concern | NO | Yes | Unclear risk | Low concern | Yes | Yes | Low risk | Low concern | Yes | Yes | Yes | Low risk |
| 20 | Wang, 2023 | Unclear | NO | NO | High risk | High concern | NO | Yes | Unclear risk | Low concern | Yes | Yes | Low risk | Low concern | Yes | Yes | Yes | Low risk |
| 21 | Xiao, 2023 | Unclear | NO | NO | High risk | High concern | NO | Yes | Unclear risk | Low concern | Yes | Yes | Low risk | Low concern | Yes | Yes | Yes | Low risk |
| 22 | Morey-León, 2023 | Unclear | NO | NO | High risk | High concern | NO | Yes | Unclear risk | Low concern | Yes | Yes | Low risk | Low concern | Yes | NO | NO | High risk |
| 23 | Billard-Pomares, 2022 | Yes | Yes | Yes | Low risk | Low concern | NO | Yes | Unclear risk | Low concern | Yes | Yes | Low risk | Low concern | Yes | Yes | Yes | Low risk |
| 24 | Quagliaro, 2023 | Yes | Yes | Yes | Low risk | Low concern | NO | Yes | Unclear risk | Low concern | Yes | Yes | Low risk | Low concern | Yes | Yes | Yes | Low risk |
| 25 | Daniyarov, 2023 | Unclear | NO | NO | High risk | High concern | NO | Yes | Unclear risk | Low concern | Yes | Yes | Low risk | Low concern | Yes | Yes | Yes | Low risk |
| 26 | Lim, 2023 | Yes | Yes | Yes | Low risk | Low concern | NO | Yes | Unclear risk | Low concern | Yes | Yes | Low risk | Low concern | Yes | Yes | NO | Low risk |
| 27 | Shaw, 2023 | Unclear | NO | NO | High risk | High concern | NO | Yes | Unclear risk | Low concern | Yes | Yes | Low risk | Low concern | Yes | NO | Yes | Low risk |
| 28 | Cloutier Charette, 2024 | Unclear | Yes | Yes | High risk | Low concern | NO | Yes | Unclear risk | Low concern | Yes | Yes | Low risk | Low concern | Yes | Yes | NO | Low risk |
| 29 | He, 2024 | Yes | NO | NO | High risk | High concern | NO | Yes | Unclear risk | Low concern | Yes | Yes | Low risk | Low concern | Yes | Yes | NO | Low risk |
| 30 | Liu, 2024 | Unclear | NO | NO | High risk | Low concern | NO | Yes | Unclear risk | Low concern | Yes | Yes | Low risk | Low concern | Yes | Yes | Yes | Low risk |
| 31 | Rukmana, 2024a | Unclear | NO | NO | High risk | High concern | NO | Yes | Unclear risk | Low concern | Yes | Yes | Low risk | Low concern | Yes | Yes | Yes | Low risk |
| 32 | Rukmana, 2024b | Unclear | NO | NO | High risk | High concern | NO | Yes | Unclear risk | Low concern | Yes | Yes | Low risk | Low concern | Yes | Yes | Yes | Low risk |
| 33 | Sadovska, 2024 | Unclear | NO | Yes | High risk | High concern | NO | Yes | Unclear risk | Low concern | Yes | Yes | Low risk | Low concern | Yes | NO | NO | High risk |
