# Supplementary material for: Diagnostic yield of nine user-friendly bioinformatics tools for predicting Mycobacterium tuberculosis drug resistance: A systematic review and network meta-analysis
Source: PLOS Glob Public Health. 2025 Apr 21;5(4):e0004465. doi: 10.1371/journal.pgph.0004465 (PMC12011222; doi:10.1371/journal.pgph.0004465)
Supplement: S5 Table — (DOCX) [file pgph.0004465.s014.docx]

| Table 5. Relative sensitivity and specificity of PhyResSE, Mykrobe, and TBProfiler stratified by culture-based pDSTs. | | | | | | | |
| --- | --- | --- | --- | --- | --- | --- | --- |
| Categories | Studies (MDR/XDR; sizes) | Pooled sensitivity (95% CI) | Pooled specificity ( 95% CI) | Relative sensitivity  (95% CI) | P value | Relative specificity  (95% CI) | P value |
| **PhyResSE** |  |  |  |  |  |  |  |
| Pyrazinamide |  |  |  |  |  |  |  |
| WHO_current | 7 (396; 1149) | 0.86 (0.58-0.97) | 0.98 (0.93-1.00) | 1 | 0.66 | 1 | 0.47 |
| WHO_past | 3 (68; 250) | 0.76 (0.25-0.97) | 0.93 (0.61-0.99) | 0.88 (0.50-1.55) |  | 0.95 (0.82-1.10) |  |
| **Mykrobe** |  |  |  |  |  |  |  |
| Streptomycin |  |  |  |  |  |  |  |
| WHO_current | 9 (250; 818) | 0.81(0.59-0.93) | 0.96(0.66-1.00) | 1 | 0.94 | 1 | 0.46 |
| WHO_past | 3 (26; 157) | 0.82(0.42-0.97) | 1.00(0.69-1.00) | 1.01(0.70-1.48) |  | 1.04(0.94-1.14) |  |
| **TBProfiler** |  |  |  |  |  |  |  |
| Kanamycin |  |  |  |  |  |  |  |
| WHO_current | 6 (418; 470) | 0.98 (0.88-1.00) | 0.99 (0.87-1.00) | 1 | 0.00 | 1 | 0.66 |
| WHO_past/undefined | 4 (622; 805) | 0.81 (0.69-0.89) | 0.97 (0.64-1.00) | 0.82 (0.73-0.94) |  | 0.98 (0.89-1.07) |  |
| Ofloxacin |  |  |  |  |  |  |  |
| WHO_current | 5 (530; 688) | 0.92 (0.88-0.95) | 0.97 (0.91-0.99) |  | 0.56 |  | 0.69 |
| WHO_past/undefined | 4 (313; 478) | 0.91 (0.85-0.94) | 0.96 (0.90-0.99) | 0.98 (0.92-1.04) |  | 0.99 (0.94-1.04) |  |
| Pyrazinamide |  |  |  |  |  |  |  |
| WHO_current | 8 (638; 1146) | 0.83 (0.69-0.91) | 0.98 (0.93-1.00) | 1 | 0.31 | 1 | 0.42 |
| WHO_past | 4 (106; 307) | 0.68 (0.41-0.87) | 0.95 (0.77-0.99) | 0.82 (0.56-1.20) |  | 0.96 (0.88-1.06) |  |
| Streptomycin |  |  |  |  |  |  |  |
| WHO_current | 15 (1318; 1819) | 0.93(0.88-0.97) | 0.94(0.82-0.98) | 1 | 0.24 | 1 | 0.82 |
| WHO_past/undefined | 4 (111; 367) | 0.83(0.60-0.94) | 0.95(0.67-1.00) | 0.88(0.72-1.09) |  | 1.02(0.89-1.16) |  |
| Abbreviations: MDR, multidrug-resistant; XDR, extensively drug-resistant. | | | | | | | |
