# Supplementary material for: Diagnostic yield of nine user-friendly bioinformatics tools for predicting Mycobacterium tuberculosis drug resistance: A systematic review and network meta-analysis
Source: PLOS Glob Public Health. 2025 Apr 21;5(4):e0004465. doi: 10.1371/journal.pgph.0004465 (PMC12011222; doi:10.1371/journal.pgph.0004465)
Supplement: S6 Table — (DOCX) [file pgph.0004465.s015.docx]

| Table 6. Network meta-analysis of bioinformatics tools for predicting drug resistance to first- and second-line anti-TB drugs. | | | | | | | | | | |
| --- | --- | --- | --- | --- | --- | --- | --- | --- | --- | --- |
| Relative sensitivity | | | | | | | | | | Drugs |
| Relative speciﬁcity | *TBProfiler* | 0.98 (0.90-1.08) | 0.95 (0.88-1.02) | 1.21 (0.98-1.72) | 0.94 (0.85-1.05) | 1.00 (0.90-1.17) | 1.28 (0.97-1.96) | 1.02 (0.90-1.25) | 1.15 (0.92-1.83) | Isoniazid |
|  | 1.05 (0.99-1.11) | *Mykrobe* | 0.99 (0.92-1.05) | 1.24 (1.00-1.75) | 0.96 (0.87-1.08) | 1.02 (0.92-1.19) | 1.31 (1.00-2.02) | 1.05 (0.92-1.30) | 1.18 (0.96-1.86) |  |
|  | 1.04 (0.99-1.10) | 1.00 (0.94-1.06) | *PhyResSE* | **1.27 (1.02-1.78)** | 0.98 (0.90-1.10) | 1.04 (0.95-1.22) | **1.33 (1.01-2.05)** | 1.07 (0.95-1.30) | 1.20 (0.97-1.89) |  |
|  | 1.03 (0.97-1.12) | 0.99 (0.92-1.08) | 0.99 (0.92-1.08) | *TGS-TB* | 0.78 (0.52-0.97) | 0.83 (0.58-1.04) | 1.07 (0.69-1.69) | 0.85 (0.59-1.12) | 0.96 (0.65-1.54) |  |
|  | 1.05 (0.99-1.14) | 1.01 (0.95-1.10) | 1.02 (0.95-1.10) | 1.03 (0.94-1.13) | *KvarQ* | 1.06 (0.94-1.25) | 1.36 (1.04-2.11) | 1.09 (0.94-1.34) | 1.22 (0.97-1.92) |  |
|  | 1.04 (0.98-1.14) | 1.01 (0.94-1.09) | 1.01 (0.94-1.09) | 1.02 (0.93-1.12) | 0.99 (0.91-1.09) | *CASTB* | 1.29 (0.96-1.99) | 1.03 (0.86-1.28) | 1.16 (0.91-1.81) |  |
|  | 0.97 (0.93-1.04) | 0.93 (0.88-1.00) | 0.94 (0.88-1.00) | 0.95 (0.87-1.02) | 0.92 (0.85-0.99) | 0.93 (0.85-1.00) | *MTBseq* | 0.83 (0.50-1.13) | 0.93 (0.56-1.49) |  |
|  | 1.01 (0.94-1.12) | 0.97 (0.90-1.08) | 0.97 (0.90-1.08) | 0.98 (0.88-1.11) | 0.96 (0.87-1.07) | 0.96 (0.87-1.08) | 1.04 (0.95-1.16) | *SAM-TB* | 1.13 (0.86-1.71) |  |
|  | 0.99 (0.92-1.37) | 0.96 (0.87-1.32) | 0.96 (0.87-1.34) | 0.97 (0.85-1.31) | 0.95 (0.84-1.29) | 0.95 (0.84-1.30) | 1.03 (0.92-1.42) | 0.99 (0.85-1.33) | *GenTB* |  |
|  | *TBProfiler* | 1.01 (0.95-1.10) | 0.99 (0.92-1.05) | 1.20 (0.94-2.02) | 1.00 (0.90-1.18) | 1.02 (0.93-1.23) | 1.18 (0.94-1.84) | 1.00 (0.88-1.26) | 1.25 (0.95-2.30) | Rifampicin |
|  | 1.04 (0.99-1.10) | *Mykrobe* | 0.99 (0.91-1.04) | 1.18 (0.92-2.00) | 0.99 (0.87-1.17) | 1.01 (0.91-1.20) | 1.17 (0.92-1.81) | 0.99 (0.86-1.24) | 1.24 (0.94-2.26) |  |
|  | **1.08 (1.01-1.24)** | 1.05 (0.94-1.19) | *PhyResSE* | 1.22 (0.96-2.09) | 1.02 (0.92-1.21) | 1.04 (0.95-1.23) | 1.21 (0.96-1.90) | 1.02 (0.90-1.28) | 1.28 (0.98-2.37) |  |
|  | 1.09 (0.97-1.41) | 1.05 (0.76-1.37) | 1.00 (0.85-1.28) | *TGS-TB* | 0.89 (0.57-1.10) | 0.95 (0.72-1.13) | 1.10 (0.76-1.68) | 0.93 (0.66-1.16) | 1.15 (0.75-2.04) |  |
|  | 1.13 (0.98-1.48) | 1.09 (0.92-1.42) | 1.04 (0.86-1.38) | 1.04 (0.79-1.41) | *KvarQ* | 1.03 (0.91-1.22) | 1.18 (0.92-1.81) | 1.00 (0.83-1.27) | 1.25 (0.93-2.26) |  |
|  | 1.12 (0.99-1.47) | 1.09 (0.93-1.41) | 1.03 (0.86-1.34) | 1.04 (0.79-1.36) | 1.01 (0.74-1.32) | *CASTB* | 1.15 (0.89-1.78) | 0.97 (0.78-1.21) | 1.22 (0.89-2.19) |  |
|  | 1.07 (0.96-1.40) | 1.04 (0.91-1.37) | 0.99 (0.84-1.30) | 0.99 (0.74-1.30) | 0.97 (0.74-1.28) | 0.97 (0.75-1.25) | *MTBseq* | 0.87 (0.54-1.13) | 1.08 (0.63-1.83) |  |
|  | 1.06 (0.95-1.39) | 1.02 (0.89-1.35) | 0.97 (0.82-1.28) | 0.97 (0.74-1.29) | 0.96 (0.73-1.26) | 0.96 (0.73-1.29) | 0.99 (0.70-1.31) | *SAM-TB* | 1.27 (0.94-2.22) |  |
|  | 1.11 (0.93-2.06) | 1.08 (0.87-1.97) | 1.02 (0.81-1.88) | 1.02 (0.72-1.93) | 1.00 (0.71-1.84) | 1.01 (0.71-1.83) | 1.04 (0.71-1.93) | 1.06 (0.74-2.00) | *GenTB* |  |
|  | *TBProfiler* | 0.97 (0.90-1.03) | 1.00 (0.94-1.06) | 1.02 (0.94-1.13) | 0.92 (0.86-0.98) | 0.98 (0.90-1.08) | 0.98 (0.88-1.15) | 1.01 (0.92-1.13) | **1.30 (1.07-1.65)** | Ethambutol |
|  | **1.26 (1.11-1.44)** | *Mykrobe* | 1.03 (0.97-1.10) | 1.06 (0.97-1.17) | 0.95 (0.88-1.02) | 1.01 (0.93-1.12) | 1.02 (0.91-1.19) | 1.04 (0.95-1.17) | **1.34 (1.11-1.70)** |  |
|  | 1.08 (0.99-1.20) | **0.86 (0.73-0.99)** | *PhyResSE* | 1.03 (0.94-1.14) | **0.92 (0.86-0.99)** | 0.98 (0.90-1.08) | 0.98 (0.87-1.15) | 1.01 (0.92-1.13) | **1.30 (1.08-1.64)** |  |
|  | 1.03 (0.94-1.20) | **0.83 (0.70-0.98)** | 0.96 (0.84-1.14) | *TGS-TB* | **0.90 (0.81-0.99)** | 0.96 (0.86-1.08) | 0.97 (0.84-1.15) | 0.99 (0.87-1.14) | **1.28 (1.03-1.63)** |  |
|  | **1.44 (1.10-2.09)** | 1.17 (0.86-1.69) | **1.35 (1.03-1.91)** | **1.41 (1.05-2.02)** | *KvarQ* | 1.07 (0.97-1.19) | 1.07 (0.95-1.25) | 1.10 (1.00-1.23) | **1.41 (1.16-1.80)** |  |
|  | **1.36 (1.07-1.90)** | 1.09 (0.83-1.53) | 1.27 (0.97-1.77) | 1.32 (1.00-1.85) | 0.99 (0.64-1.42) | *CASTB* | 1.00 (0.87-1.17) | 1.03 (0.90-1.18) | **1.33 (1.08-1.71)** |  |
|  | 1.14 (0.91-1.87) | 0.92 (0.69-1.50) | 1.07 (0.83-1.80) | 1.11 (0.84-1.82) | 0.83 (0.54-1.41) | 0.85 (0.54-1.40) | *MTBseq* | 1.03 (0.87-1.21) | **1.33 (1.05-1.73)** |  |
|  | 1.15 (1.00-1.47) | 0.92 (0.77-1.19) | 1.08 (0.92-1.37) | 1.12 (0.91-1.45) | 0.83 (0.56-1.17) | 0.85 (0.56-1.17) | 1.02 (0.55-1.43) | *SAM-TB* | **1.30 (1.07-1.63)** |  |
|  | 1.12 (0.96-1.47) | 0.90 (0.74-1.19) | 1.05 (0.89-1.38) | 1.09 (0.89-1.42) | 0.81 (0.54-1.15) | 0.84 (0.57-1.17) | 1.01 (0.53-1.43) | 0.98 (0.76-1.28) | *GenTB* |  |
|  | *TBProfiler* | **1.02 (0.94-1.14)** | **1.06 (1.01-1.11)** | 1.00 (0.95-1.06) | 1.04 (0.99-1.11) | 1.01 (0.96-1.06) | 1.05 (0.97-1.17) | 1.03 (0.99-1.11) | **1.07 (1.01-1.17)** | Pyrazinamide |
|  | 1.08 (0.81-1.46) | *Mykrobe* | **1.05 (1.01-1.11)** | 0.99 (0.94-1.06) | 1.03 (0.98-1.10) | 1.00 (0.96-1.06) | 1.04 (0.97-1.16) | 1.03 (0.98-1.10) | **1.06 (1.01-1.16)** |  |
|  | 1.08 (0.86-1.37) | 1.00 (0.72-1.36) | *PhyResSE* | 0.95 (0.89-1.01) | 0.98 (0.93-1.05) | 0.95 (0.90-1.00) | 0.99 (0.91-1.11) | 0.98 (0.93-1.04) | 1.01 (0.96-1.10) |  |
|  | 0.76 (0.56-0.98) | **0.76 (0.61-0.94)** | 1.00 (1.00-1.00) | *TGS-TB* | 1.04 (0.98-1.12) | 1.01 (0.94-1.07) | 1.05 (0.96-1.18) | 1.04 (0.97-1.13) | 1.07 (1.00-1.19) |  |
|  | **1.71 (1.01-3.22)** | 1.60 (0.90-3.04) | 1.61 (0.94-2.98) | **2.09 (1.21-3.98)** | *KvarQ* | 0.97 (0.89-1.03) | 1.01 (0.92-1.12) | 1.00 (0.93-1.07) | 1.03 (0.96-1.14) |  |
|  | **1.76 (1.01-3.46)** | 1.65 (0.89-3.24) | 1.67 (0.94-3.25) | **2.18 (1.26-4.19)** | 1.11 (0.45-2.37) | *CASTB* | 1.04 (0.96-1.16) | 1.03 (0.97-1.12) | 1.07 (1.00-1.16) |  |
|  | 2.42 (0.80-10.52) | 2.26 (0.71-9.84) | 2.28 (0.72-9.83) | 2.97 (0.98-12.63) | 1.51 (0.36-6.28) | 1.50 (0.33-5.74) | *MTBseq* | 0.99 (0.88-1.09) | 1.02 (0.90-1.14) |  |
|  | 0.89 (0.69-1.29) | 0.83 (0.59-1.22) | 0.84 (0.62-1.25) | 1.09 (0.84-1.60) | **0.55 (0.25-0.94)** | 0.56 (0.23-1.00) | 0.60 (0.09-1.18) | *SAM-TB* | 1.04 (0.97-1.13) |  |
|  | 0.91 (0.69-1.36) | 0.84 (0.60-1.30) | 0.86 (0.62-1.31) | 1.12 (0.85-1.70) | 0.56 (0.26-1.00) | 0.57 (0.23-1.01) | 0.60 (0.09-1.28) | 1.03 (0.65-1.59) | *GenTB* |  |
|  | *TBProfiler* | 0.98 (0.87-1.09) | 0.98 (0.87-1.10) | 1.13 (0.90-1.57) | 0.92 (0.80-1.13) | 1.43 (0.97-2.34) | 1.10 (0.83-1.91) | 0.99 (0.81-1.42) | 1.04 (0.83-1.57) | Streptomycin |
|  | **1.13 (1.03-1.32)** | *Mykrobe* | 0.99 (0.83-1.16) | 1.18 (0.92-1.66) | 0.95 (0.81-1.17) | 1.48 (1.00-2.46) | 1.15 (0.86-2.00) | 1.03 (0.82-1.49) | 1.08 (0.85-1.66) |  |
|  | 1.06 (0.98-1.20) | 0.93 (0.80-1.06) | *PhyResSE* | 1.18 (0.92-1.63) | 0.95 (0.79-1.16) | 1.46 (0.98-2.33) | 1.12 (0.82-1.98) | 1.01 (0.80-1.43) | 1.06 (0.82-1.60) |  |
|  | 1.10 (0.96-1.33) | 0.97 (0.82-1.17) | 1.05 (0.88-1.26) | *TGS-TB* | 0.81 (0.56-1.05) | 1.23 (0.72-2.05) | 0.95 (0.56-1.76) | 0.86 (0.55-1.30) | 0.90 (0.57-1.44) |  |
|  | 1.12 (0.99-1.35) | 0.99 (0.85-1.19) | 1.07 (0.92-1.28) | 1.03 (0.82-1.28) | *KvarQ* | **1.56 (1.03-2.60)** | 1.20 (0.86-2.01) | 1.09 (0.82-1.59) | 1.14 (0.85-1.75) |  |
|  | **1.31 (1.07-1.70)** | 1.16 (0.94-1.51) | 1.25 (1.02-1.62) | 1.19 (0.94-1.57) | 1.19 (0.93-1.53) | *CASTB* | 0.80 (0.43-1.43) | 0.73 (0.41-1.21) | 0.76 (0.40-1.33) |  |
|  | 1.18 (0.91-1.93) | 1.04 (0.78-1.70) | 1.12 (0.85-1.86) | 1.08 (0.77-1.84) | 1.06 (0.77-1.74) | 0.91 (0.61-1.48) | *MTBseq* | 0.96 (0.54-1.45) | 1.01 (0.58-1.59) |  |
|  | 1.03 (0.91-1.29) | 0.91 (0.78-1.14) | 0.98 (0.85-1.22) | 0.94 (0.76-1.20) | 0.93 (0.75-1.16) | 0.79 (0.58-1.04) | 0.91 (0.51-1.25) | *SAM-TB* | 1.07 (0.74-1.63) |  |
|  | 0.94 (0.85-1.22) | 0.83 (0.71-1.07) | 0.89 (0.78-1.16) | 0.86 (0.70-1.12) | 0.85 (0.69-1.11) | **0.73 (0.52-0.98)** | 0.84 (0.48-1.17) | 0.91 (0.72-1.19) | *GenTB* |  |
|  | *TBProfiler* | 0.94 (0.82-1.10) | 0.94 (0.82-1.08) | 0.98 (0.83-1.29) | 1.03 (0.81-1.86) | 1.59 (0.93-4.16) | 1.24 (0.83-2.89) | 1.02 (0.84-1.49) | 1.08 (0.82-1.93) | Amikacin |
|  | 1.27 (0.93-1.89) | *Mykrobe* | 1.00 (0.88-1.16) | 1.06 (0.90-1.40) | 1.09 (0.87-1.99) | 1.71 (1.00-4.46) | 1.33 (0.91-3.10) | 1.09 (0.90-1.58) | 1.16 (0.90-2.09) |  |
|  | 1.18 (0.94-1.68) | 0.98 (0.68-1.47) | *PhyResSE* | 1.05 (0.88-1.39) | 1.09 (0.84-1.99) | 1.70 (0.96-4.50) | 1.32 (0.87-3.10) | 1.08 (0.88-1.60) | 1.14 (0.86-2.04) |  |
|  | 1.20 (0.93-1.79) | 1.02 (0.69-1.56) | 1.06 (0.72-1.59) | *TGS-TB* | 1.06 (0.74-1.96) | 1.59 (0.75-4.18) | 1.25 (0.73-3.05) | 1.03 (0.70-1.53) | 1.11 (0.73-2.06) |  |
|  | 11506.45 (0.84-56922.77) | 9358.14 (0.62-40597.92) | 9820.18 (0.62-46351.81) | 10222.74 (0.59-52037.60) | *KvarQ* | 1.64 (0.78-4.37) | 1.28 (0.62-3.00) | 1.06 (0.64-1.54) | 1.11 (0.61-1.91) |  |
|  | 1.16 (0.83-2.89) | 0.98 (0.60-2.32) | 1.04 (0.64-2.53) | 0.99 (0.55-2.30) | 0.62 (0.00-1.99) | *CASTB* | 0.89 (0.27-2.19) | 0.74 (0.25-1.24) | 0.77 (0.26-1.48) |  |
|  | 1.21 (0.85-2.62) | 1.03 (0.62-2.21) | 1.06 (0.65-2.32) | 1.01 (0.54-2.27) | 0.70 (0.00-1.99) | 1.18 (0.48-2.59) | *MTBseq* | 0.90 (0.32-1.42) | 0.95 (0.35-1.79) |  |
|  | 1.09 (0.82-2.17) | 0.92 (0.59-1.79) | 0.96 (0.59-1.99) | 0.94 (0.53-1.88) | 0.61 (0.00-1.66) | 1.05 (0.43-2.22) | 1.00 (0.43-2.02) | *SAM-TB* | 1.06 (0.67-1.78) |  |
|  | 1.13 (0.81-2.54) | 0.94 (0.59-2.05) | 0.98 (0.59-2.16) | 0.94 (0.50-2.15) | 0.66 (0.00-1.92) | 1.08 (0.40-2.45) | 1.02 (0.39-2.29) | 1.07 (0.44-2.35) | *GenTB* |  |
|  | *TBProfiler* | 0.92 (0.76-1.08) | 0.99 (0.80-1.47) | 0.98 (0.74-1.46) | 1.01 (0.80-1.43) | 1.02 (0.78-1.66) | 0.94 (0.72-1.40) | 1.46 (0.68-3.69) |  | Capreomycin |
|  | 1.32 (0.93-1.98) | *Mykrobe* | 1.07 (0.89-1.55) | 1.08 (0.86-1.64) | 1.11 (0.93-1.57) | 1.12 (0.93-1.82) | 1.04 (0.84-1.58) | 1.63 (0.85-4.11) |  |  |
|  | 1.17 (0.78-2.35) | 0.92 (0.45-1.85) | *PhyResSE* | 1.03 (0.75-1.52) | 1.05 (0.81-1.48) | 1.07 (0.80-1.66) | 0.99 (0.70-1.47) | 1.52 (0.68-3.99) |  |  |
|  | 1.15 (0.72-2.32) | 0.93 (0.48-1.93) | 1.05 (0.48-2.18) | *TGS-TB* | 1.05 (0.68-1.54) | 1.05 (0.58-1.73) | 1.00 (0.63-1.54) | 1.48 (0.56-3.60) |  |  |
|  | 1.32 (0.79-3.07) | 1.05 (0.53-2.35) | 1.17 (0.55-2.66) | 1.25 (0.47-3.05) | *SAM-TB* | 1.04 (0.76-1.66) | 0.95 (0.60-1.40) | 1.46 (0.64-3.65) |  |  |
|  | 1.83 (0.99-3.71) | 1.36 (0.60-2.48) | 1.61 (0.84-3.27) | 1.83 (0.74-3.61) | 1.61 (0.71-3.57) | *GenTB* | 0.95 (0.53-1.46) | 1.43 (0.47-3.61) |  |  |
|  | 1588.01 (0.71-1302.79) | 1258.96 (0.50-988.62) | 1356.96 (0.47-1239.76) | 1424.35 (0.49-1165.65) | 1432.23 (0.38-994.53) | 1083.74 (0.28-781.60) | *KvarQ* | 1.59 (0.70-3.85) |  |  |
|  | 9.55 (0.69-67.29) | 6.45 (0.49-42.66) | 7.00 (0.47-45.98) | 4.68 (0.47-25.15) | 6.23 (0.38-42.17) | 4.02 (0.27-23.96) | 10.30 (0.00-78.50) | *CASTB* |  |  |
|  | *TBProfiler* | 0.85 (0.63-1.21) | 1.00 (0.71-1.51) | 0.72 (0.53-1.00) | **0.71 (0.52-0.91)** |  |  |  |  | Ethionamide |
|  | 2.35 (0.97-5.91) | *PhyResSE* | 1.20 (0.86-1.85) | 0.87 (0.60-1.27) | 0.84 (0.56-1.10) |  |  |  |  |  |
|  | 1.02 (0.71-1.61) | **0.52 (0.18-0.99)** | *TGS-TB* | 0.74 (0.42-1.11) | 0.73 (0.43-1.00) |  |  |  |  |  |
|  | 2677522.70 (1.09-11506.83) | 1164411.49 (0.39-6183.78) | 3073038.96 (1.08-10884.13) | *Mykrobe* | 0.99 (0.66-1.25) |  |  |  |  |  |
|  | **7.38 (1.36-25.40)** | 4.33 (0.40-15.62) | **7.63 (1.27-26.30)** | 1.94 (0.00-10.26) | *CASTB* |  |  |  |  |  |
|  | *TBProfiler* | 0.96 (0.81-1.15) | 0.97 (0.85-1.14) | 1.00 (0.79-1.43) | 0.92 (0.74-1.22) | 1.21 (0.76-3.22) | 1.43 (0.80-4.00) | 0.94 (0.80-1.14) | 1.06 (0.79-1.88) | Kanamycin |
|  | 1.36 (0.92-2.36) | *Mykrobe* | 1.02 (0.88-1.21) | 1.04 (0.83-1.52) | 0.96 (0.78-1.26) | 1.27 (0.81-3.33) | 1.49 (0.82-4.35) | 0.98 (0.83-1.19) | 1.11 (0.84-1.97) |  |
|  | 1.56 (1.01-2.91) | 1.09 (0.54-2.14) | *PhyResSE* | 1.03 (0.80-1.52) | 0.94 (0.68-1.25) | 1.25 (0.78-3.27) | 1.47 (0.81-4.16) | 0.97 (0.80-1.17) | 1.09 (0.78-1.86) |  |
|  | 1.07 (0.78-1.75) | 0.72 (0.33-1.30) | 0.71 (0.33-1.26) | *TGS-TB* | 0.95 (0.63-1.31) | 1.25 (0.70-3.08) | 1.48 (0.72-4.34) | 0.98 (0.66-1.26) | 1.10 (0.64-1.96) |  |
|  | 9291.62 (0.79-845.44) | 5936.05 (0.37-565.56) | 6120.61 (0.37-483.66) | 9546.23 (0.65-759.72) | *KvarQ* | 1.35 (0.83-3.54) | 1.55 (0.72-4.03) | 1.04 (0.79-1.30) | 1.17 (0.76-2.05) |  |
|  | 1.88 (0.77-7.63) | 1.24 (0.33-4.94) | 1.24 (0.37-5.21) | 1.80 (0.61-6.97) | 1.06 (0.00-4.23) | *CASTB* | 1.35 (0.35-4.05) | 0.91 (0.33-1.25) | 1.00 (0.30-1.89) |  |
|  | 1.36 (0.81-3.29) | 0.88 (0.33-2.33) | 0.90 (0.34-2.37) | 1.30 (0.62-3.27) | 0.84 (0.00-2.65) | 1.08 (0.13-2.81) | *MTBseq* | 0.83 (0.27-1.20) | 0.92 (0.30-1.72) |  |
|  | 1.08 (0.74-2.24) | 0.73 (0.31-1.54) | 0.69 (0.30-1.42) | 1.03 (0.54-2.11) | 0.67 (0.00-1.81) | 0.87 (0.12-2.05) | 0.90 (0.27-2.02) | *SAM-TB* | 1.12 (0.81-1.85) |  |
|  | 1.79 (0.88-4.50) | 1.11 (0.45-2.97) | 1.13 (0.44-2.93) | 1.75 (0.73-4.41) | 1.11 (0.00-3.60) | 1.46 (0.17-4.28) | 1.50 (0.34-4.26) | 1.78 (0.65-4.58) | *GenTB* |  |
|  | *TBProfiler* | 0.99 (0.68-1.40) | 0.96 (0.62-1.60) | 1.03 (0.57-2.30) | 0.88 (0.54-1.59) | 1.10 (0.71-2.23) | 1.45 (0.79-3.22) | 0.88 (0.54-1.72) |  | Levofloxacin |
|  | 1.19 (0.86-1.98) | *PhyResSE* | 0.98 (0.62-1.53) | 1.09 (0.56-2.97) | 0.90 (0.52-1.58) | 1.11 (0.69-2.32) | 1.49 (0.79-3.43) | 0.91 (0.55-1.77) |  |  |
|  | 1.22 (0.85-2.50) | 1.10 (0.69-1.95) | *Mykrobe* | 1.16 (0.55-3.04) | 0.94 (0.53-1.73) | 1.14 (0.73-1.99) | 1.54 (0.83-3.59) | 0.95 (0.51-1.86) |  |  |
|  | 1.13 (0.66-2.58) | 1.00 (0.48-2.25) | 0.96 (0.34-2.27) | *MTBseq* | 0.97 (0.39-1.79) | 1.25 (0.47-2.85) | 1.66 (0.52-4.00) | 0.98 (0.38-2.00) |  |  |
|  | 1.40 (0.83-3.84) | 1.23 (0.67-3.25) | 1.14 (0.44-2.70) | 1.42 (0.41-4.24) | *KvarQ* | 1.35 (0.80-2.59) | 1.80 (0.83-4.26) | 1.11 (0.57-2.17) |  |  |
|  | 1.16 (0.75-2.67) | 1.01 (0.58-2.28) | 0.94 (0.40-1.92) | 1.16 (0.42-2.74) | 0.89 (0.23-1.94) | *SAM-TB* | 1.43 (0.59-3.48) | 0.86 (0.40-1.77) |  |  |
|  | 1.13 (0.72-2.39) | 0.97 (0.49-2.01) | 0.91 (0.38-1.77) | 1.14 (0.46-2.47) | 0.86 (0.22-1.81) | 1.03 (0.39-2.10) | *TGS-TB* | 0.68 (0.24-1.35) |  |  |
|  | 1.84 (0.88-4.82) | 1.53 (0.74-3.96) | 1.48 (0.55-3.32) | 1.92 (0.51-4.85) | 1.52 (0.39-3.72) | 1.77 (0.52-4.09) | 1.74 (0.68-4.09) | *CASTB* |  |  |
|  | *TBProfiler* | 0.96 (0.73-1.31) | 1.04 (0.83-1.51) | 1.13 (0.83-1.69) | 1.19 (0.77-2.65) | 1.00 (0.80-1.55) | 1.30 (0.85-2.97) | 0.93 (0.71-1.32) |  | Moxifloxacin |
|  | 1.09 (0.84-1.58) | *Mykrobe* | 1.05 (0.78-1.52) | 1.17 (0.84-1.78) | 1.23 (0.76-2.76) | 1.05 (0.79-1.65) | 1.35 (0.87-3.21) | 0.97 (0.71-1.41) |  |  |
|  | 1.16 (0.94-1.69) | 1.09 (0.76-1.62) | *PhyResSE* | 1.12 (0.78-1.70) | 1.18 (0.71-2.65) | 0.98 (0.73-1.49) | 1.27 (0.79-2.93) | 0.92 (0.64-1.34) |  |  |
|  | 1.02 (0.76-1.53) | 0.92 (0.59-1.41) | 0.88 (0.49-1.37) | *TGS-TB* | 1.09 (0.56-2.47) | 0.91 (0.53-1.54) | 1.21 (0.62-2.94) | 0.83 (0.51-1.25) |  |  |
|  | 1.58 (0.81-4.67) | 1.50 (0.69-4.30) | 1.43 (0.67-4.19) | 1.64 (0.80-4.81) | *MTBseq* | 0.99 (0.41-1.61) | 1.26 (0.47-2.98) | 0.91 (0.38-1.46) |  |  |
|  | 1.65 (0.78-4.59) | 1.55 (0.73-4.50) | 1.48 (0.70-3.85) | 1.73 (0.84-4.90) | 1.30 (0.28-4.12) | *KvarQ* | 1.29 (0.66-2.71) | 0.96 (0.56-1.40) |  |  |
|  | 2.37 (0.85-6.59) | 2.17 (0.73-5.90) | 1.99 (0.67-5.63) | 2.45 (0.83-6.86) | 1.94 (0.32-5.58) | 1.46 (0.25-4.41) | *SAM-TB* | 0.83 (0.39-1.26) |  |  |
|  | 1.76 (0.94-4.77) | 1.64 (0.84-4.56) | 1.55 (0.80-4.28) | 1.83 (0.95-4.95) | 1.43 (0.35-4.00) | 1.36 (0.25-3.75) | 1.34 (0.30-3.88) | *CASTB* |  |  |
|  | *TBProfiler* | 1.00 (0.88-1.18) | 1.01 (0.90-1.20) | 1.04 (0.92-1.38) | 0.99 (0.85-1.38) |  |  |  |  | Ofloxacin |
|  | 1.00 (0.83-1.30) | *Mykrobe* | 1.02 (0.86-1.24) | 1.04 (0.87-1.40) | 0.99 (0.80-1.38) |  |  |  |  |  |
|  | 1.08 (0.91-1.57) | 1.10 (0.84-1.61) | *PhyResSE* | 1.04 (0.85-1.36) | 0.99 (0.76-1.40) |  |  |  |  |  |
|  | 0.95 (0.80-1.23) | 0.97 (0.75-1.26) | 0.91 (0.60-1.16) | *TGS-TB* | 0.96 (0.64-1.32) |  |  |  |  |  |
|  | 2.06 (0.93-5.99) | 2.15 (0.90-6.15) | 1.99 (0.85-5.29) | 2.14 (0.97-6.08) | *CASTB* |  |  |  |  |  |
|  | *TBProfiler* | 0.96 (0.87-1.02) | 0.95 (0.82-1.03) | 0.96 (0.84-1.09) | 0.97 (0.81-1.22) | 1.15 (0.89-2.25) |  |  |  | Para-aminosalicylic acid |
|  | 0.71 (0.46-1.02) | *TGS-TB* | 1.00 (0.91-1.08) | 1.01 (0.91-1.16) | 1.02 (0.90-1.27) | 1.22 (0.98-2.50) |  |  |  |  |
|  | 0.70 (0.39-1.24) | 1.07 (0.59-1.85) | *Mykrobe* | 1.01 (0.91-1.18) | 1.03 (0.89-1.31) | 1.21 (0.86-2.49) |  |  |  |  |
|  | 1.65 (0.48-5.83) | 2.46 (0.68-8.51) | 2.48 (0.58-8.19) | *PhyResSE* | 1.02 (0.86-1.30) | 1.22 (0.97-2.45) |  |  |  |  |
|  | 153903.00 (0.48-8300.78) | 229736.50 (0.70-13440.57) | 179850.60 (0.63-12093.99) | 175461.50 (0.24-12487.79) | *SAM-TB* | 1.18 (0.85-2.35) |  |  |  |  |
|  | 1.06 (0.56-2.78) | 1.62 (0.82-4.46) | 1.58 (0.74-4.28) | 1.42 (0.25-3.93) | 0.91 (0.00-3.22) | *CASTB* |  |  |  |  |
|  | *TBProfiler* | 1.04 (0.59-2.10) | 1.22 (0.68-2.36) | 1.22 (0.67-2.55) |  |  |  |  |  | Prothionamide |
|  | 1.75 (0.63-6.58) | *Mykrobe* | 1.25 (0.57-2.62) | 1.26 (0.54-2.79) |  |  |  |  |  |  |
|  | 1461.85 (0.75-758.06) | 1242.80 (0.40-695.00) | *SAM-TB* | 1.07 (0.45-2.25) |  |  |  |  |  |  |
|  | 1.20 (0.46-3.18) | 0.94 (0.18-2.91) | 0.56 (0.00-2.26) | *GenTB* |  |  |  |  |  |  |
| The upper triangle gives relative sensitivity (95% CI) and the comparisons should be read from top to bottom. The lower triangle gives relative speciﬁcity (95% CI) and the comparisons should be read from left to right. Bold values indicate statistically significant differences. | | | | | | | | | | |
