# Supplementary material for: Diagnostic yield of nine user-friendly bioinformatics tools for predicting Mycobacterium tuberculosis drug resistance: A systematic review and network meta-analysis
Source: PLOS Glob Public Health. 2025 Apr 21;5(4):e0004465. doi: 10.1371/journal.pgph.0004465 (PMC12011222; doi:10.1371/journal.pgph.0004465)
Supplement: Attachment — Submitted filename: Response to Reviewers.docx [file pgph.0004465.s017.docx]

Dear Prof. Faksri,

We sincerely appreciate the time and effort that you and the reviewers have dedicated to providing valuable feedback on our manuscript, titled "Diagnostic yield of eight user-friendly bioinformatics tools for predicting Mycobacterium tuberculosis drug resistance: a systematic review and network meta-analysis" (MS# PGPH-D-24-02151). We have carefully considered the reviewers' comments and have revised our manuscript accordingly.

We are pleased to submit the revised version of our manuscript, which includes the following:

1. A marked-up version of the manuscript with all changes highlighted.

2. A clean version of the revised manuscript without tracked changes.

3. A detailed rebuttal letter that addresses each of the reviewers' comments individually.

We hope that the revisions satisfy the journal's standards, and we remain open to any further suggestions or feedback that you may have.

Thank you again for your kind consideration, and we look forward to your response.

Yours sincerely,

Mao-Shui Wang

Shandong Public Health Clinical Center

Shandong University

Journal Requirements:

1. As required by our policy on Data Availability, please ensure your manuscript or supplementary information includes the following:

A numbered table of all studies identified in the literature search, including those that were excluded from the analyses.

Responses:

Thank you for your kind suggestion. A total of 1,325 records were identified through database searching (Figure 1). Of these, 680 records were removed either due to duplication (n=491) or for other reasons (n=189): non-English articles (n=12), book chapters (n=28), conference abstracts (n=46), reviews (n=88), correspondence or notes (n=15). The remaining 645 articles were assessed for their titles and abstracts by two reviewers (CYL and HY) independently. During this independent screening, duplicates were set aside for further assessment, and any discrepancies were discussed with a third reviewer (GCP). After screening the title and abstract, 553 articles were excluded, and 92 full-text articles were assessed for eligibility. Ultimately, 33 articles were included in the final analysis.

A detailed account of the selection process, including the studies that were included and excluded, is provided in the Supplementary Materials (File 4).

For every excluded study, the table should list the reason(s) for exclusion.

Responses:

The reasons for excluding specific articles from the analysis are fully detailed in the Supplementary Materials (File 4).

If any of the included studies are unpublished, include a link (URL) to the primary source or detailed information about how the content can be accessed.

Responses:

Thank you for your comment. All of the studies included in our analysis are published and peer reviewed. Therefore, there are no unpublished studies requiring additional access information or links to primary sources.

A table of all data extracted from the primary research sources for the systematic review and/or meta-analysis. The table must include the following information for each study:

Name of data extractors and date of data extraction

Responses:

Thank you for your comment. We have provided a table with all the data extracted from the primary research sources for the systematic review and/or meta-analysis (Supplementary Table 1). Regarding the requested information about data extractors and the date of data extraction, this work was performed by CYL and HY (as noted in Line 104) over the course of approximately one month, from February to March 2024. We apologize for not having specific dates available, as data extraction was conducted continuously throughout this period.

During the revision, a second literature search was performed, and relevant data (date) for the literature review and data collection were gathered. However, to maintain consistency and avoid potential confusion for the readers, this information was not included in the revision.

Confirmation that the study was eligible to be included in the review.

Responses:

Thank you for your valuable comments. Key information related to the PICO items has been compiled and is available in the Supplementary Materials (File 4).

All data extracted from each study for the reported systematic review and/or meta-analysis that would be needed to replicate your analyses.

Responses:

All data extracted from each study for the reported systematic review and/or meta-analysis, including the details required for replication of our analyses, are provided in Supplementary Table 1. This table contains key characteristics of the included studies and all data points used in the analyses. Additionally, Supplementary Table 2 presents the data, which can be imported directly into statistical software for analyses.

If data or supporting information were obtained from another source (e.g. correspondence with the author of the original research article), please provide the source of data and dates on which the data/information were obtained by your research group.

Responses:

Thank you for your comment. All data used in our systematic review and meta-analysis were collected directly from the published papers, as stated above, with no additional unpublished data obtained from the author teams.

If applicable for your analysis, a table showing the completed risk of bias and quality/certainty assessments for each study or outcome.  Please ensure this is provided for each domain or parameter assessed. For example, if you used the Cochrane risk-of-bias tool for randomized trials, provide answers to each of the signalling questions for each study. If you used GRADE to assess certainty of evidence, provide judgements about each of the quality of evidence factor. This should be provided for each outcome.

Responses:

The results of the risk of bias and quality/certainty assessments for each study, with detailed information for each assessed domain or parameter, are presented in Supplementary Figure 1 and Supplementary Table 4. This table includes comprehensive evaluations using the Quality Assessment of Diagnostic Accuracy Studies (QUADAS-2) tool, which was applied to assess risk of bias and concerns regarding applicability.

An explanation of how missing data were handled.

Responses:

Thank you for this comment. First, the outcome data, including true positive/negative and false positive/negative events, were complete. If any such data were missing, it could not be synthesized. Second, we have specified that studies with insufficient data were excluded from further analysis (Figure 1). For subgroups, if relevant data were unavailable, the corresponding studies were excluded from the subgroup analyses.

This information can be included in the main text, supplementary information, or relevant data repository. Please note that providing these underlying data is a requirement for publication in this journal, and if these data are not provided your manuscript might be rejected.

Responses:

We have addressed these concerns as outlined above. The required underlying data have been included in the supplementary information to ensure compliance with the journal's publication requirements. If you have any further questions, please do not hesitate to let us know, and we will be happy to make additional revisions.

Additional Editor Comments (if provided):

[Note: HTML markup is below. Please do not edit.]

Reviewers' comments:

Reviewer's Responses to Questions

**Comments to the Author**

1. Does this manuscript meet PLOS Global Public Health’s [publication criteria](https://track.editorialmanager.com/CL0/https:%2F%2Fjournals.plos.org%2Fglobalpublichealth%2Fs%2Fcriteria-for-publication/1/010f0192d2e73583-02426c27-0ef1-4ccb-a5c1-23f79213701d-000000/Wv7UImCKQl_mfwup6LSE5ig-jNc5nOkU3PXWFoGgPp0=182)? Is the manuscript technically sound, and do the data support the conclusions? The manuscript must describe methodologically and ethically rigorous research with conclusions that are appropriately drawn based on the data presented.

Reviewer #1: Partly

Reviewer #2: Partly

2. Has the statistical analysis been performed appropriately and rigorously?

Reviewer #1: No

Reviewer #2: I don't know

3. Have the authors made all data underlying the findings in their manuscript fully available (please refer to the Data Availability Statement at the start of the manuscript PDF file)?

The [PLOS Data policy](https://track.editorialmanager.com/CL0/https:%2F%2Fjournals.plos.org%2Fglobalpublichealth%2Fs%2Fdata-availability/1/010f0192d2e73583-02426c27-0ef1-4ccb-a5c1-23f79213701d-000000/dDHPdCZfFdL0Mm1jiF6-GvHZh9ZPH4gEhzgRmT66Bn0=182) requires authors to make all data underlying the findings described in their manuscript fully available without restriction, with rare exception. The data should be provided as part of the manuscript or its supporting information, or deposited to a public repository. For example, in addition to summary statistics, the data points behind means, medians and variance measures should be available. If there are restrictions on publicly sharing data—e.g. participant privacy or use of data from a third party—those must be specified.

Reviewer #1: No

Reviewer #2: No

4. Is the manuscript presented in an intelligible fashion and written in standard English?

PLOS Global Public Health does not copyedit accepted manuscripts, so the language in submitted articles must be clear, correct, and unambiguous. Any typographical or grammatical errors should be corrected at revision, so please note any specific errors here.

Reviewer #1: Yes

Reviewer #2: Yes

Responses:

Thank you for your valuable feedback on our manuscript and for evaluating its alignment with PLOS Global Public Health’s publication criteria. We appreciate your acknowledgment of the strengths in our work, as well as your constructive comments indicating areas for improvement.

We understand the importance of ensuring that our research meets the highest methodological standards, particularly in the detailed statistical analysis and data presentation, and that our conclusions are well-supported by the results presented. We acknowledge the need to enhance the statistical analysis and improve data availability, and have carefully tried to address these concerns to strengthen the manuscript's rigor and clarity (see specific comments below).

Thank you once more. Your feedback is invaluable, and we greatly appreciate your time and thoughtful evaluation of the manuscript.

5. Review Comments to the Author
Please use the space provided to explain your answers to the questions above. You may also include additional comments for the author, including concerns about dual publication, research ethics, or publication ethics. (Please upload your review as an attachment if it exceeds 20,000 characters)

Reviewer #1: Authors has reviewed literature and compared various bioinformatics tools for predicting Mycobacterium tuberculosis drug resistance, with TBProfiler showing the best diagnostic accuracy for most anti-TB drugs. TGS-TB performed exceptionally for pyrazinamide and certain second-line drugs.

Responses:

Thank you for your thoughtful review. Many revisions have been made based on your suggestions, and we have provided detailed explanations to address your concerns below. If you have any further questions or additional feedback, please feel free to reach out, and we will be happy to revise further if needed.

There are a few queries and concerns as listed below:

1. What are the criteria for selecting the antibiotics list included in the study? We noticed that newer drugs like Bedaquiline, Delamanid, and Pretomanid, which are essential for treating rifampicin-resistant TB, were excluded from the analysis. Bedaquiline is a core drug in Rr-TB treatment regimens, and both Delamanid and Pretomanid have shown great potential in shortening MDR- and XDR-TB therapies and improving patient outcomes. Can author clarify the reasons for this exclusion?

Please refer: <https://www.sciencedirect.com/science/article/pii/S1198743X2100464X#bib3> and <https://www.ncbi.nlm.nih.gov/pmc/articles/PMC7825472/>

1. Mutations in atpE gene, encoding subunit C of the ATP synthase, can prevent BDQ from binding to the C subunit, thus resulting in BDQ resistance <https://www.ncbi.nlm.nih.gov/pmc/articles/PMC9976417/>

2. synonymous mutation, T960C, in the fgd1 gene was uniformly found in DLM-resistant mtb <https://www.ncbi.nlm.nih.gov/pmc/articles/PMC11170162/>

3. Pretomanid and delamanid are pro-drugs that share the same activation pathway, the products of ddn, fgd1, fbiA-D. Loss-of-function and certain other mutations in any of these 6 genes have been associated with high delamanid/pretomanid resistance in M. tuberculosis <https://www.ncbi.nlm.nih.gov/pmc/articles/PMC11069242/>

Responses:

Thank you very much for your insightful suggestion. In our analysis, we excluded catalog-based approaches where a single causative mutation directly suggests resistance (e.g., the World Health Organization catalog), as these do not involve a complex algorithm (Lines 98-101). This was the primary reason for excluding such papers from our study.

However, we appreciate your recommendation and have carefully reviewed the manuscript you suggested. Your feedback has been highly inspiring, and we plan to conduct a systematic review of bedaquiline (BDQ) in the future, focusing on the relationship between specific mutations and resistance levels. For instance, mutations in the *atpE* gene are typically linked with medium to high levels of resistance, whereas mutations associated with efflux pump (such as *Rv0678* and *pep*Q ) generally correspond to low to medium resistance levels (Shaw ES, et al. Bedaquiline: What Might the Future Hold? Lancet Microbe. 2024 Jul 26:100909).

A summary of these findings would be valuable, potentially supporting the use of high-dose BDQ. For instance, a review on this topic for isoniazid resistance has previously been published (Rivière E et al. Identifying Isoniazid Resistance Markers to Guide Inclusion of High-Dose Isoniazid in Tuberculosis Treatment Regimens. Clin Microbiol Infect. 2020 Oct;26(10):1332-1337).

As drug and data selection for our study depends on the scope of each included study, we were limited by the lack of novel or repurposed drugs used in the included studies. Possibly due to the absence of a gold standard for assessing drug susceptibility or unclear resistance mechanisms (BDQ: Köser CU, et al. A composite reference standard is needed for bedaquiline antimicrobial susceptibility testing for Mycobacterium tuberculosis complex. Eur Respir J. 2024 Jul 11;64(1):2400391; Shaw ES, et al. Bedaquiline: What Might the Future Hold? Lancet Microbe. 2024 Jul 26:100909).

Once again, thank you for your helpful comments. They have significantly contributed to shaping our future research directions.

2. How does the performance of other bioinformatics tools (such as TGS-TB, MTBseq, KvarQ, CASTB and SAM-TB missing in S3 Table and S3 Figure) compare in terms of sensitivity and specificity for predicting drug resistance in tuberculosis, particularly when benchmarked against WHO recommendations and phenotypic drug susceptibility testing (pDST)?

Responses:

The Supplementary Table 5 and Supplementary Figure 2 were designed to compare WHO recommendations (current vs. past versions) for phenotypic drug susceptibility testing (pDST), highlighting the critical role of pDST approaches. In this comparison, studies were included if their DST methods matched (solid or liquid culture medium), ensuring that subgroup analyses maintain consistent baseline characteristics.

The lack of comparison data for tools such as TGS-TB, MTBseq, KvarQ, CASTB, and SAM-TB is primarily due to either limited available data or significant variations in DST approaches across studies. Recognizing this limitation, we are currently undertaking an individual patient data meta-analysis in another study in which we are collecting genomic sequences and DST results for analysis of the various bioinformatics tools. This approach aims to reduce inter-study bias and achieve a high level of statistical efficacy. While this is a substantial project and is expected to be completed by the end of 2025, we believe it will lead to robust and valuable conclusions. We hope you potentially could be available for review of our future work. Thank you very much again.

3. How were the bioinformatics tools or their performance selected for comparison in this study with respect to their Superiority Index (SI) in table 1, and what specific criteria/methods were used to determine their ranking and predictive accuracy for TB drug resistance?

Responses:

Thank you for your important question. In our study, the bioinformatics tools were selected for comparison based on their relevance and established use in predicting TB drug resistance.

During the literature search, we faced challenges in designing accurate search strategies, particularly in differentiating catalog-based approaches from bioinformatics tools used for resistance prediction. Many studies using catalog-based approaches were included in the final list, and were difficult to exclude. Therefore, prior to the study, we conducted a comprehensive search for relevant tools using Google and PubMed. All identified bioinformatics tools were subsequently included in our search strategy.

We presented the SIs in Tables 3 and 4 to provide a comparative measure of each tool’s performance. The SI was calculated using a Bayesian network meta-analysis framework, and each tool's predictive accuracy for TB drug resistance was assessed across multiple performance metrics, including sensitivity and specificity.

The ranking of interventions is an attractive feature of network meta-analysis. Currently, it is still challenging to rank competing diagnostic tests. Some researchers consider diagnostic odds ratio (DOR) as an indicator of ranking of competing diagnostic tests (Glas AS, et al. The diagnostic odds ratio: a single indicator of test performance. J Clin Epidemiol. 2003 Nov;56(11):1129-35); however, the measure is not easy to interpret and use in clinical practice (Deeks JJ. Systematic reviews of evaluations of diagnostic and screening tests. In: Egger M, Smith GD, Altman DG, editors. Systematic reviews in health care: meta-analysis in context. London, UK: BMJ Publishing Group, 2001. pp 248–282). As an alternative, the SI introduced by Deutsch et al (Deutsch R, et al. Quantifying relative superiority among many binary-valued diagnostic tests in the presence of a gold standard. J Data Sci 2009;7:161–77), gives more weight to tests that perform well on both measures and less weight on those that perform poorly on both or excel in one but poorly in the other (Nyaga VN, et al. ANOVA model for network meta-analysis of diagnostic test accuracy data. Stat Methods Med Res. 2018 Jun;27(6):1766-1784). The SI ranges from 0 to infinity. In our current study, the SI for each tool was derived based on its probability of being the highest-performing tool in terms of predictive accuracy. This ranking approach and metric allowed us to objectively compare the relative effectiveness of each tool. Furthermore, no additional comparisons between SIs have been developed at this time. We believe SI provides a robust measure of each tool's performance, allowing for an effective comparison of the tools’ ability to predict TB drug resistance.

Thank you again. We hope our response has clarified your concerns. If you have any further questions, please feel free to leave a comment.

4. In page no. 9, line no. 152, What factors contributed to the high risk of bias in sample and patient selection across the majority of studies, and how might the unblinded MTB resistance profiling impact the applicability of the findings? Additionally, what measures were taken to ensure low bias risk in the index and reference test domains, as well as in the flow and timing domain?

Responses:

The methodological quality assessment of 33 studies using the QUADAS-2 tool highlighted significant concerns in patient selection, with the majority of studies (Supplementary Table 4) exhibiting a high risk of bias. This was primarily attributed to non-randomized sampling, inappropriate exclusions, and the use of case-control designs, practices that could introduce potential biases affecting the reliability of findings.

Thank you for your insightful comment regarding the impact of unblinded MTB (*Mycobacterium tuberculosis*) resistance profiling on the applicability of our findings. The lack of blinding in resistance profiling can introduce potential biases, such as observer bias, which may affect the accuracy and reliability of diagnostic test results. This, in turn, could influence the overall conclusions of our meta-analysis by potentially overestimating the effectiveness of the diagnostic tools evaluated. This bias as a disadvantage has been noted in the limitation section. Moving forward, we recommend that future research in this area incorporate blinding wherever feasible to minimize bias and enhance the validity and applicability of the results. By doing so, we can ultimately aid clinicians and policymakers in making informed decisions.

Additionally, we did not implement any specific measures regarding the index and reference test domains. Instead, we assessed the risk of bias and presented the corresponding results to help readers interpret our findings. These identified risks were considered solely for assessment purposes and did not serve as grounds for excluding studies from the review.

5. How did the Bayesian approach differ from traditional meta-analytic methods in assessing the diagnostic accuracy of the bioinformatics tools? In the abstract, the authors mention that the data were collected and pooled using random-effects meta-analysis and Bayesian network meta-analysis. However, the implications of the random-effects meta-analysis are not discussed in the manuscript as well as supplementary.

Responses:

Thank you for your important point. The differences between the Bayesian approach and traditional meta-analytic methods are as follows:

Traditional meta-analyses, specifically random-effects meta-analysis, was utilized to assess pooled performance metrics such as sensitivity, specificity, and AUC (See Tables 1 and 2, Supplementary Figures 2 and 3). This method was employed to describe the performance of individual bioinformatics tools. In contrast, Bayesian network meta-analysis (NMA) was designed to evaluate comparative measures, including superiority index (SI), relative sensitivity, and relative specificity (refer to Tables 3, 4, and Supplementary Table 6). This approach enabled direct comparisons between the performance of the different bioinformatics tools. Of importance, the random-effects meta-analysis (traditional approach) and Bayesian network meta-analysis were conducted sequentially, not in parallel.

Therefore, the Bayesian network meta-analysis was specifically used for calculating relative sensitivity, relative specificity, and SI to rank the tools. All other measures, such as pooled sensitivity, specificity, AUC, and meta-regression analyses, were performed using the traditional random-effects approach. Additionally, we have included a detailed description of the statistical methods in the Supplementary Material (File 3), which outlines the full workflow using Streptomycin as an example. This additional documentation may clarify the methodology and sequencing of analyses.

We acknowledge that the implications of the random-effects meta-analysis were not extensively discussed in the manuscript. Our primary focus was on comparing the performance of the bioinformatics tools and explaining the differences between them, as we believe this discussion holds greater clinical significance and enhances the applicability of these tools. However, we recognize the importance of addressing the random-effects meta-analysis more explicitly. To avoid redundancy, we have tried to elaborate on this in the discussion of performance differences.

6. How were the heterogeneity (Differences between studies) and inconsistency (Differences in Results between tools) of studies accounted for in the Bayesian network meta-analysis?

What implications do the results of the Bayesian network meta-analysis have for clinical practice and the management of TB drug resistance?

As mentioned in the previous question, why did the author not discuss about random-effects meta-analysis anywhere in the manuscript, which is known to address heterogeneity and inconsistency?

Responses:

Thank you for highlighting that we needed this in the presentation of the data.

1. Consistency in the SI and NMA: The principle of the SI assumes consistency as a fundamental premise of its statistical method (Stat Methods Med Res. 2018 Jun;27(6):1766-1784). Incoherence or inconsistency in NMA arises when direct and indirect evidence for the same comparison differ substantially. Since the arm-based model implicitly assumes consistency, methods designed to detect and quantify inconsistency in contrast-based models are not directly applicable to arm-based models.

2. Systematic Comparisons and Mitigation of Bias: Under the ANOVA-based Bayesian NMA model, each diagnostic method is systematically compared within the framework, which helps mitigate study-level biases. From our perspective, inconsistency or incoherence can be seen as a form of heterogeneity between studies, often caused by missing data or the influence of outlying studies (Stat Methods Med Res. 2018 Jun;27(6):1766-1784). In the model, the effects of individual studies on pooled estimates are captured by study-level effects. Additionally, the model hypothetically allows any two diagnostic tools to be directly compared within a study, reducing the impact of inconsistency (Stat Methods Med Res. 2018 Jun;27(6):1766-1784).

That said, identifying influential observations within the Bayesian framework remains computationally challenging and is an active area of research. To date, there is no widely accepted method for specifically identifying heterogeneity and inconsistency in the context of network meta-analyses. Even in high-impact publications (e.g., Lancet Glob Health. 2017 Aug;5(8):e760-e771), discussions of heterogeneity are typically limited to pairwise comparisons and rarely extend to NMA. This highlights the ongoing need for methodological advancements in this area.

3. Sequential Approach and Random-Effects Meta-Analysis: In our study, we adopted a sequential approach, performing traditional random-effects meta-analysis before conducting the Bayesian NMA. The random-effects meta-analysis provided pooled estimates and accounted for heterogeneity among individual tools. While the Bayesian NMA focused on ranking tools and comparing their performance, the random-effects meta-analysis offered a robust foundation for understanding between-study variability.

We acknowledge that heterogeneity and inconsistency are complex issues, particularly in the Bayesian NMA of diagnostic studies. These challenges remain an area of ongoing methodological development. We also recognize the importance of addressing these concerns more explicitly in future analyses. Your thoughtful feedback has provided valuable insights, and we sincerely appreciate any further suggestions you may have to enhance our understanding and improve our study.

7. In the study by Xiao et al., they used whole-genome sequencing (WGS) to predict drug resistance in Mycobacterium tuberculosis and found high accuracy for several antibiotics, including isoniazid and rifampicin. They mentioned that the Total Genotyping Solution for TB (TGS-TB) worked well for many drugs. In contrast, the review focused mainly on one antibiotic (pyrazinamide) and reported high effectiveness for it, along with good performance for other drugs like kanamycin and moxifloxacin. Why does the study show that TGS-TB is effective for many antibiotics, while the review only highlights one antibiotic? What could explain this difference in focus? (Please refer: <https://www.nature.com/articles/s41598-023-29652-3>).

Responses:

First, we used the study by Xiao et al. as a reference to explain why TGS-TB demonstrates such excellent performance in predicting pyrazinamide (PZA) resistance compared to other tools. The paper attributes this superior performance to TGS-TB's enhanced ability to detect insertions and deletions, which is particularly crucial for PZA resistance. Unlike other drugs, there is currently no conclusive drug susceptibility testing (DST) approach for PZA, making tools like TGS-TB especially valuable and warranting further discussion in our study.

Additionally, our study is a meta-analysis aiming to provide a pooled estimate of the effectiveness across multiple studies. While TGS-TB demonstrated strong performance in the Xiao et al. study, this does not necessarily mean the tool will yield the same level of effectiveness when data are pooled across various studies. Our focus on PZA in the review aims to provide a balanced analysis based on the available evidence, recognizing that pooled estimates may differ from the findings of individual studies.

However, I agree with your point. If more data become available, updating the meta-analysis could be highly beneficial in strengthening and refining our current findings, especially since the pooled estimates are based on data from individual studies.

8. Why did the author omit the ARIBA (Antimicrobial Resistance Identification By Assembly) tool, which demonstrates better specificity and lower major error compared to KvarQ, MTBseq, Mykrobe, and TB-profiler? Please see Table 5 in this publication for reference: <https://www.ncbi.nlm.nih.gov/pmc/articles/PMC7004237/>.

Responses:

Thank you for your comment. We have reviewed the paper and assessed its eligibility. However, since the results were based on a secondary analysis of data from existing databases, it met our exclusion criteria. Therefore, we excluded this study from our final analysis.

In our study, we aim to consider the variability introduced by different sequencing platforms across sites. To address this, we included only studies with original data, avoiding the limitations of secondary analyses, where the same sequences may be reused across different studies.

Regarding ARIBA, we included it in our search strategy and repeated the literature selection process. The result is shown in Figure 1. ARIBA is a tool that efficiently and accurately identifies antimicrobial resistance genes and variants from paired sequencing reads using a combined mapping/alignment and targeted local assembly approach. However, it is not specifically designed for TB, unlike other investigated tools that are more suitable for this purpose. Although there are many similar tools with comparable functionality, including additional (non-TB specific) tools would complicate the analysis considerably. Nevertheless, we acknowledge this as a limitation of the study.

9. The raw data and codes were missing, and the methodology section was poorly written and lacked clarity in details. To ensure the reproducibility of the results in terms of statistical and scientific performance, it is essential for the authors to provide this information.

Responses:

Thank you for your honest feedback. The methods section has been revised thoroughly, trying to enhance clarity. To illustrate the analytical process, we have included an example using streptomycin, along with the corresponding raw data and analysis code. These details are provided in the Supplementary Materials (Files 3 and Supplementary Table 2) to ensure transparency and reproducibility.

Reviewer #2: Introduction
This is a review of the article “Diagnostic yield of eight user-friendly bioinformatics tools for predicting Mycobacterium tuberculosis drug resistance: a systematic review and network meta-analysis” by Ya-Li Chen et al, submitted for publication to PLOS Global Public Health. The authors report an assessment of the diagnostic yield of 8 bioinformatics tools for Mycobacterium tuberculosis drug resistance prediction through a systematic review of published studies. Their comparison, based on several statistical methods, indicate TBProfiler is the best method overall but suggest other tools are better for the prediction of the resistance to certain drugs.

Merits
Although the authors were not the first to do a comparative assessment of bioinformatics tools for the prediction of Mycobacterium tuberculosis drug resistance (see for example reference 13 in the paper), they are responsible for the most comprehensive comparison, both in terms of the number of programs they evaluated, the search sources they employed and the amount of data they considered. Their search strategy, explained in Supplementary File 1, is simple yet effective and allowed the authors to build a comprehensive dataset. Although the data was already published, they were also the first to scrape the data and offer it in the form of written tables, which is a much-appreciated first step towards their availability in public databases.

The authors declared that this systematic review and network meta-analysis are compliant with suggested reporting guidelines. I found it to be compliant with most items in the PRISMA checklist [1]. I can also confirm that this protocol is registered in PROSPERO as stated in the text. The authors defined eligibility criteria that are specific and which I consider to be appropriate for the analysis. They also specifically report the risk of bias in their analysis which gives credibility to their protocol.

The methods are well laid out and the statistical analysis seems to be performed equally well, including in the case of subgroup analyses.

Responses:

Thank you for your time and thoughtful review of our manuscript. We appreciate your expertise and comments, especially regarding the strengths of our methodology and findings. We aimed to provide a comprehensive assessment of these bioinformatics tools, and your feedback affirms the value of our work in contributing to the field of TB drug resistance prediction. Thank you once again.

Critique

My main and very important concern with this work is that the data is not readily available and that the statistical analysis is not reproducible. I encourage the authors to publish both the data (if possible, or at least a protocol to extract the data from the original work) and the computational analysis pipeline in an open repository for others to check the results and confirm ain conclusions. The latter would be facilitative if the outdoors chose to conduct the statistical analysis in an open package like R instead of Stata. The openness is also needed given the advancement of bioinformatics tools, as this approach would allow anyone to extend the comparison and assess the performance as new methods become available.

Responses:

Thank you for pointing this out. To increase transparency, we have now included the original data with the resubmission, allowing for replication or our analyses. Additionally, we have added a detailed description of the analysis process using streptomycin as an example. This includes the original data and the R language syntax used for the analysis, which can be found in the Supplementary Materials (Files 4). These revisions, to our knowledge, adhere to the journal’s open policy and transparency requirements, facilitating readers’ understanding of our data and methods.

Our statistical analyses were conducted using both Stata and R. Specifically, pooled sensitivities, specificities, AUCs, and meta-regression analyses were performed using Stata, while relative sensitivities, specificities, and summary indices (SIs) were estimated using R. Detailed descriptions of this methodology are included in the statistical analysis section of the manuscript, as well as in the Supplementary Materials.

We appreciate the suggestion to use open-source tools, such as R, to enhance accessibility and reproducibility. While we recognize the advantages of R – and will keep this in mind moving forward - Stata offers analytic tools highly suitable for this study. Its user-friendly interface and optimized routines provide efficient and reliable workflows, particularly for complex tasks such as pooled sensitivity, specificity, AUC, and meta-regression analyses. However, certain aspects of our study, such as the NMA, could easily be conducted using R. Researchers familiar with performing NMAs in R will likely also have experience with performing traditional meta-analyses in R, allowing for replication of our methods. Due to the extensive work of our analyses, and our experience and familiarity with Stata, we have not re-performed all analyses in only one of the programs. We believe our methodology could be replicated in R for a majority of the analyses.

To improve broader accessibility and reproducibility, we have provided a detailed documentation of the entire analytical process (using Streptomycin as an example), along with raw data and R syntax, in the Supplementary Materials (Files 4).

Since the search strategy is relatively simple and crucial for the data collected, readers may want to know which other strategies the authors considered. Likewise, since the studies considered were independently evaluated by two reviewers, it is not transparent how they finally decided on any discrepancies.

Responses:

Thank you for your valuable suggestion. Prior to conducting the database search, we carried out an informal systematic review of the currently available bioinformatics tools. As a result, the search strategy was intentionally kept simple but effective for capturing the relevant data. We appreciate your feedback, and if any further clarification is needed, we will be happy to make additional revisions.

A total of 1,325 records were identified through database searches (Figure 1). Of these, 680 records were removed due to duplication (n=491) or for other reasons (n=189), including non-English articles (n=12), book chapters (n=28), conference abstracts (n=46), reviews (n=88), and correspondence or notes (n=15). The remaining 645 articles were independently assessed for their titles and abstracts by two reviewers (CYL and HY). During this process, duplicates were set aside for further review, and any discrepancies were resolved through discussion with a third reviewer (GCP). As a result, 553 articles were excluded in this phase. Subsequently, 92 full-text articles were assessed for eligibility, leading to the inclusion of 33 articles for analysis. A detailed account of the selection process, including documentation of both included and excluded studies, is available in the Supplementary Materials (File 4). Regarding data collection, we have provided a table containing all data extracted from the primary research sources for the systematic review and/or meta-analysis in Supplementary Tables 1 and 2.

Discussion
The authors have presented a very comprehensive analysis that promises to be useful for the entire community of researchers. However, I could not find the underlying data or statistical analysis needed to reproduce the findings, therefore I should raise caution in the credibility of this meta-analysis. I expect the authors to be capable of providing these and resubmitting the text for publication. If the analysis proves to be well-done, the conclusions presented by the authors will be supported by clear results. The manuscript is generally well written, with a correct use of English grammar and vocabulary.

Responses:

Thank you for your important comment. We have now included the original data, along with a comprehensive description of the analysis process (using Streptomycin as an example), including syntax and detailed results, during the resubmission.

Together with the comments in the previous sections, please note below some suggestions that may help to improve the manuscript:

I strongly suggest the authors include a warning around line 246, suggesting that using these bioinformatics tools without previous experience is problematic and might confound the researcher, no matter how well they perform and how friendly they are.

Response:

Thank you for your important suggestion. We have included a warning to address the potential challenges for clinicians, researchers, or technicians who may lack prior experience with these bioinformatics tools (Line 274-276).

While SAM-TB performs best for the prediction of the performance of some drugs in the Bayesian network meta-analysis, it is not considered in the resulting comparative analysis. The authors should explain better and earlier in the manuscript why they decided to leave this program out. Moreover, they should figure out if they still want to include this method or not; perhaps they require an additional criterium that would take program out of the analysis.

Responses:

Thank you for your comment. Due to the limited and highly heterogeneous data derived from only two studies, the confidence interval around the estimates of SAM-TB's absolute sensitivity and specificity is wide, resulting in a high degree of uncertainty. Consequently, it is challenging to draw reliable conclusions regarding its performance. Therefore, while SAM-TB exhibited a good SI for certain drugs, caution is warranted when interpreting these findings, and further research is needed to assess its reliability and effectiveness. Additionally, tools like GenTB and MTBseq, which were only evaluated in single studies, were also excluded from the conclusions due to the same lack of sufficient data, posing a significant risk of bias. We have now added an explanation in the manuscript’s result section (Line 231-233).

Our inclusion and exclusion criteria did not specify a minimum number of studies for a tool to be considered for our analyses. As a result, we chose to present the corresponding data for these tools for readers’ reference, even though its inclusion in preliminary evaluations may appear ambiguous. We believe this approach provides readers with useful insights into the performance of each tool despite the limitations behind these tools’ data. We acknowledge that excluding these tools from the final analysis represents a limitation of our study, and this has been clearly stated in the discussion (Line 312-315).

Given the activity in the field, it is not surprising to find 19 more search results in PubMed only as of October 25, 2024, using the authors' search strategy. Therefore, I would recommend the authors to update their results and, provided the papers and the data satisfy the eligibility criteria, incorporate the extra data in their analysis, which should be straightforward if the analysis is reproducible
Responses:

Thank you for your thoughtful suggestion. We have now updated the search for relevant articles, initially covering publications up to November 6, 2023, and subsequently extending the search to October 25, 2024. The electronic search strategy used for this updated search is detailed in Supplementary File 2. Additionally, we have performed a thorough examination of 86 full-text articles, which are documented in Supplementary File 3. We appreciate your recommendation to incorporate additional data that meet the eligibility criteria, as it aligns with our commitment to ensuring the robustness and reproducibility of our analysis.

Although the report is largely compliant with the PRISMA-NMA checklist [1], I suggest the authors particularly check if the abstract includes funding information and registration number.
Introduction. In line 62, "(...) drug resistance (i.e., isoniazid (...)”, I suggest replacing 'i.e.' with 'e.g.' as not all first-line drugs are mentioned there.

Responses:

Thank you for this suggestion. We have replaced "i.e.," with "e.g.," in line 69 to accommodate this.

Figure 2. I think the data would be better presented as a table to encourage others to extract it. Regardless, the title and data in the columns need a better alignment. Also, it would be useful to explain again in this and other figure's captions what is the meaning of abbreviations like "M/XDR-TB".

Responses:

Thank you for your helpful feedback. We have included a table in the Supplementary Materials (File 3) to facilitate extraction by others. Additionally, we have adjusted the alignment of the title and data in the columns for improved clarity, and have also included explanations for each abbreviations in tables and figures.

Supplementary Figure 3. For clear identification, if possible, name the method and antibiotic used at the top of each panel instead of in the caption

Responses:

Thank you for the suggestion. We have revised Supplementary Figures 2, 3, 4, and 5 by adding the method and antibiotic name at the top of each panel for clearer identification, as recommended.

Table 1. For a clearer analysis, please separate first- and second-order drugs with a horizontal line or in two separate tables. Also, please indicate in the caption why are some values stressed in bold.

Response:

Thank you for your valuable suggestion. We have addressed this by separating the results into the following tables for greater clarity:

Table 1. Diagnostic accuracy of WGS analysis tools in predicting resistance to all first-line anti-TB drugs.

Table 2. Diagnostic accuracy of WGS analysis tools in predicting resistance to any second-line anti-TB drugs.

Table 3. Bayesian network meta-analysis of the diagnostic accuracy of bioinformatics tools for predicting resistance to all first-line anti-TB drugs.

Table 4. Bayesian network meta-analysis of the diagnostic accuracy of bioinformatics tools for predicting resistance to any second-line anti-TB drug.

Additionally, we have clarified in the table captions that the values highlighted in bold indicate statistically significant differences in relative sensitivity and relative specificity in the Bayesian NMA. Thank you for helping improve the clarity and readability of our results.

Results > Bayesian network meta-analysis. Lines 202 to 205 need reviewing, because for example, the pooled sensitivity and specificity of the bioinformatics tools are also listed in Table 1.

Response:

Thank you for pointing this out. We have updated the Results section. This concern has been addressed in Lines 219-221.

Results > Bayesian network meta-analysis. The Bayesian network meta-analysis described between lines 207 to 213 seems to be missing the resistance prediction to levofloxacin. I suggest the authors add a the analysis here.

Response:

Thank you. We have revised it and updated the Results section.

Results > Bayesian network meta-analysis. Add 'respectively' nearly at the end of line 210.

Response:

We have updated the Results section accordingly, and the error has been corrected in Line 220.

Bibliography

<https://www.prisma-statement.org/nma>

Response:

Thank you for your comment. We have already cited the PRISMA guideline in our manuscript (Line 85). Please let us know if there is anything specific you feel needs further clarification regarding this reference.

6. PLOS authors have the option to publish the peer review history of their article ([what does this mean?](https://track.editorialmanager.com/CL0/https:%2F%2Fjournals.plos.org%2Fglobalpublichealth%2Fs%2Feditorial-and-peer-review-process%23loc-peer-review-history/1/010f0192d2e73583-02426c27-0ef1-4ccb-a5c1-23f79213701d-000000/mSMoLlUF3eO3gGhpYjoOzr241HYpmqrveQGbVEzM42Y=182)). If published, this will include your full peer review and any attached files.
**Do you want your identity to be public for this peer review?** If you choose “no”, your identity will remain anonymous but your review may still be made public.
For information about this choice, including consent withdrawal, please see our [Privacy Policy](https://track.editorialmanager.com/CL0/https:%2F%2Fwww.plos.org%2Fprivacy-policy/1/010f0192d2e73583-02426c27-0ef1-4ccb-a5c1-23f79213701d-000000/xeB2_nC9lfFO3ZEjNBCW5OnKytQCtixAF06s162Onp8=182).

Reviewer #1: **Yes:** Anshu Bhardwaj

Reviewer #2: **Yes:** Nicolas Palopoli

Response:

Again, we would like to extend our sincere thanks to Prof. Anshu Bhardwaj and Prof. Nicolas Palopoli for their valuable time, expertise, and thoughtful feedback on our manuscript. The constructive comments and insights have been instrumental in improving the quality of our work. The feedback has also inspired us and may lead to future research projects. We also greatly appreciate the willingness to share your identities in the peer review process. Thank you once again for your expertise, time, and support.
